# Genome-wide detection of copy number variation in Chinese indigenous sheep using an ovine high-density 600 K SNP array

**DOI:** 10.1038/s41598-017-00847-9

**Published:** 2017-04-19

**Authors:** Qing Ma, Xuexue Liu, Jianfei Pan, Lina Ma, Yuehui Ma, Xiaohong He, Qianjun Zhao, Yabin Pu, Yingkang Li, Lin Jiang

**Affiliations:** 1 Institute of Animal Science, Ningxia Academy of Agriculture and Forestry Sciences, Yinchuan, Ningxia 75002 China; 2 Institute of Animal Science, Chinese Academy of Agricultural Sciences (CAAS), No. 2 Yuanmingyuan West Road, Beijing, 100193 China; 3 CAAS-ILRI Joint Laboratory on Livestock and Forage Genetic Resources, Institute of Animal Science, Chinese Academy of Agricultural Sciences (CAAS), No. 2 Yuanmingyuan West Road, Beijing, 100193 China

## Abstract

Copy number variants (CNVs) represent a form of genomic structural variation underlying phenotypic diversity. In this study, we used the Illumina *Ovine* SNP 600 K BeadChip array for genome-wide detection of CNVs in 48 Chinese Tan sheep. A total of 1,296 CNV regions (CNVRs), ranging from 1.2 kb to 2.3 Mb in length, were detected, representing approximately 4.7% of the entire ovine genome (Oar_v3.1). We combined our findings with five existing CNVR reports to generate a composite genome-wide dataset of 4,321 CNVRs, which revealed 556 (43%) novel CNVRs. Subsequently, ten novel CNVRs were randomly chosen for further quantitative real-time PCR (qPCR) confirmation, and eight were successfully validated. Gene functional enrichment revealed that these CNVRs cluster into Gene Ontology (GO) categories of homeobox and embryonic skeletal system morphogenesis. One CNVR overlapping with the homeobox transcription factor *DLX3* and previously shown to be associated with curly hair in sheep was identified as the candidate CNV for the special curly fleece phenotype in Tan sheep. We constructed a Chinese indigenous sheep genomic CNV map based on the Illumina *Ovine* SNP 600 K BeadChip array, providing an important addition to published sheep CNVs, which will be helpful for future investigations of the genomic structural variations underlying traits of interest in sheep.

## Introduction

Copy number variations (CNVs), which represent a type of genomic structural variation, are DNA segments ranging in size from 1 kilobase (kb) to several megabases (Mbs) in which duplication or deletion events have occurred^1^. Previous studies have shown that CNVs influencing genes or gene regions are associated with important phenotypic traits in livestock. For example, duplications of the *KIT* gene constitute the *Dominant white* locus in pigs^2, 3^. In chicken, the pea-comb phenotype is caused by a CNV in intron 1 of the *SOX5* gene^4^, and the late feathering locus comprises a partial duplication of the *PRLR* and *SPEF2* genes^5^. A 1.6-kb deletion in *TBX3* disrupts the asymmetric hair pigmentation that underlies Dun camouflage coloring in horses^6^. As one of the first domesticated animals, sheep (*Ovis aries*) have played an important role in human society^7^. Although increasing attention has been paid at identifying ovine CNVs^8–13^, the total number of CNVs, particularly in Chinese indigenous sheep, has been limited. None of the previously described ovine CNVs except for the agouti duplication, which affects the *ASIP* locus in sheep and contributes to coat color variability, have been determined to have a direct effect on a sheep trait^14, 15^. Therefore, more efforts are needed to identify CNVs, one of the most important types of genomic variation, in the sheep genome.

In recent years, advances in high-throughput genome scanning technologies, especially SNP arrays, DNA hybridization on array platforms, and next-generation sequencing methods, have allowed the identification of genome-wide structural variants that, due to their small size, are undetectable using microscopic techniques^16^. Compared to the other two technological platforms, SNP array is more cost-effective, allowing users to increase the number of samples on a limited budget and, as a result, achieve a more desirable performance in large-scale CNV detections, particularly at the genome-wide scale^17^. SNP arrays are also advantageous due to their high signal-to-noise ratios and the use of B-allele frequency, which facilitates the interpretation of results^18^. Furthermore, less sample per experiment is required for SNP arrays compared to that required for array-based comparative genomic hybridization (aCGH)^19^. However, the main bias of SNP arrays on CNV detection is the low SNP coverage of the genomic regions that often harbor CNVs^20^. This bias may be minimized to some extent by increasing the genome coverage using commercial high-density SNP arrays. Therefore, the detection of CNVs by high-density SNP arrays has become in increasingly common and performed successfully in various species.

Tan sheep, one of the most important sheep breeds indigenous to China, are reared in Ningxia Province and are renowned for their production of high-quality pelts and long-term adaptation to the dry, cold and windy climate of northwestern China. The famous lamb pelts from Tan sheep are the result of long-term artificial selection and thus exhibit a lustrous white curly fleece that disappears gradually with age. Earlier studies have focused on various candidate genes to investigate the genetic mechanism of the distinct curly fleece trait (e.g., polymorphisms in the *KRT1.2* (*keratin 1.2*)^21^ and *KAP1.3* (*keratin associated protein 1.3*)^22^ genes, which are related to wool curvature). The latest skin transcriptome profiling of Tan sheep at ages 1 and 48 months determined that the keratin genes (including *KRT25*, *KRT5*, *KRT7*, and *KRT14*) and their associated pathways, which were previously shown to be associated with hair/fleece development and function, are expressed differentially between 1 and 48 months of age^23^. However, the genetic components behind the white curly fleece phenomenon and the adaptation to harsh environments in Tan sheep are still unclear.

The primary aim of this study was to conduct a genome-wide survey of sheep CNV regions (CNVRs) using the Illumina *Ovine* SNP 600 K BeadChip array and to explore the roles of the potentially specific CNVs in Chinese Tan sheep. First, a reliable algorithm was used to assay 48 animals to obtain highly convincing CNVs. Second, the identified CNVRs were compared with five existing CNVR reports in sheep and qPCR was conducted to validate a subset of the novel CNVRs. Finally, the potentially breed-specific CNVRs were determined and the functional relevance of the CNVR-harboring genes was further analyzed using Gene Ontology enrichment and the QTL database (http://www.animalgenome.org/cgi-bin/QTLdb/OA/browse).

## Results

### Genome-wide detection of CNVs and CNVRs in Chinese Tan sheep

**Figure 1:**
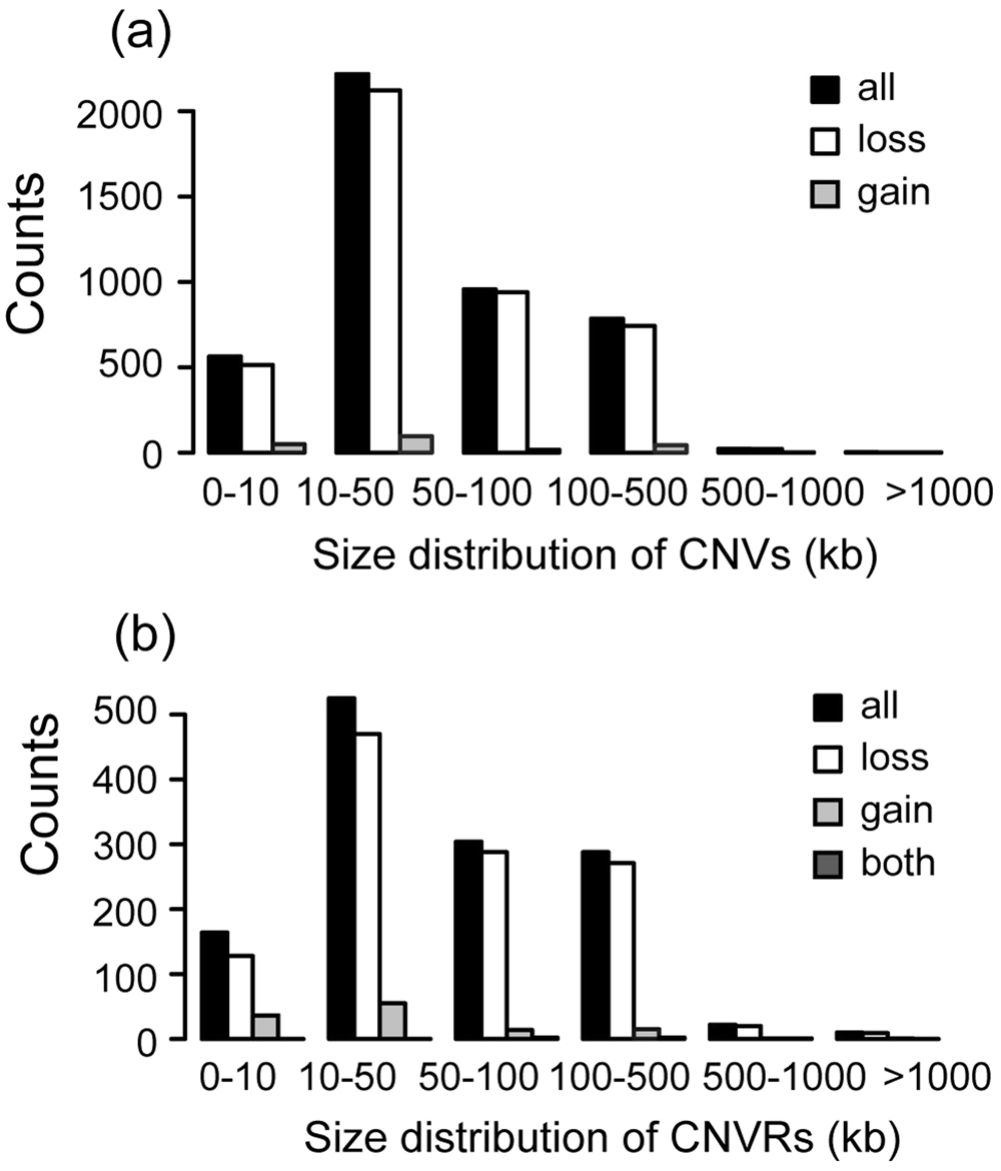
Length distribution of CNVs (**a**) and CNVRs (**b**) in Tan sheep.

After employing a stringent CNV calling pipeline, we identified 5,190 autosomal CNVs (4,985 losses and 205 gains) in 48 Chinese Tan sheep (Additional file: Table S1). In this study, we defined “loss” and “gain” as deletions and insertions relative to the normal di-allele copy number in the ovine genome. The average length of the CNVs was 64.1 kb, and the median length was 47.1 kb. We found that approximately 48.5% of the CNVs range from 10 kb to 50 kb, 21.7% of CNVs range from 50 kb to 100 kb in size, with 12.2% being small fragment CNVs (<10 kb) (Fig. 1a). We found the number of CNVs in each individual to vary from 140 to 560. After aggregating the overlapping CNVs, we obtained 1,296 autosomal CNVRs representing 121.8 Mb (4.7%) of the entire ovine genome (Additional file: Table S2). The average CNVR size is 92.7 kb, ranging from 1.2 kb to 2.3 Mb. CNVRs ranging in size from 10 kb to 500 kb represent the majority (86.2%), whereas CNVRs larger than 1 Mb were rarely observed (0.8%) (Fig. 1b).

**Figure 2:**
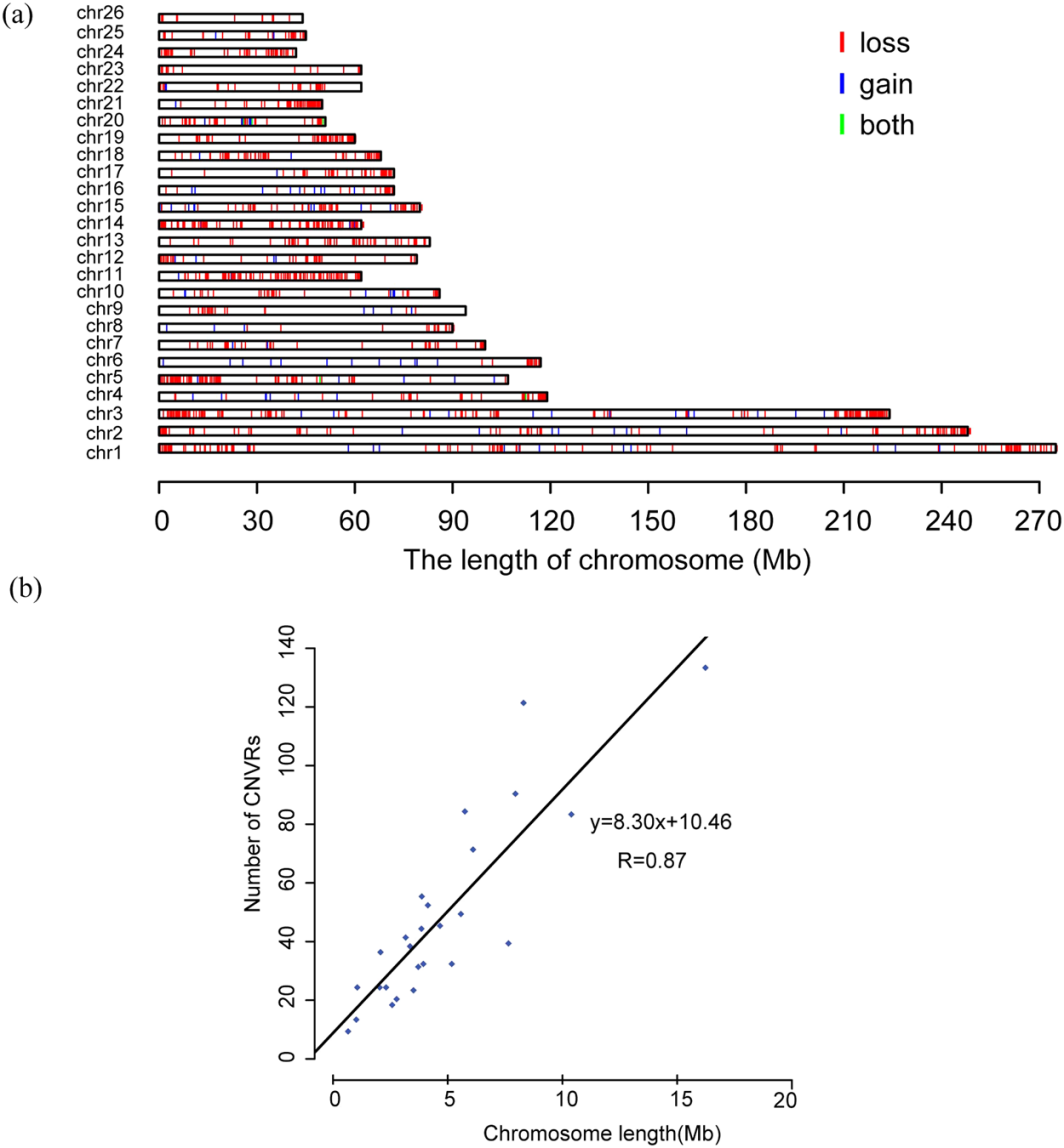
Genome-wide distribution of autosomal CNVRs in Tan sheep. (**a**) A map of CNVRs in the sheep genome; blue, green and red represent gain, loss and both (gain and loss), respectively. (**b**) Correlation between CNVR counts and chromosome length.

We generated a genome-wide map of CNVRs in Tan sheep (Fig. 2a). The chromosomal proportion covered by CNVRs varies between chromosomes, ranging from 1.5% on OAR26 to 18.2% on OAR24 (Table 1). The number of chromosomal CNVRs ranges from 15 to 135 on OAR23 and OAR3, and the chromosome length and the number of CNVRs show a strong positive linear relationship (Fig. 2b, R^2^ = 0.87). The average distance between the CNVRs on each chromosome ranges from 467.5 kb on OAR6 to 544.9 kb on OAR11. The closest CNVRs are 4.5 kb apart on OAR3, whereas the largest inter-CNV distance is 34.4 Mb on OAR23.

**Table 1:** The distribution of CNVRs in the ovine autosomes.

Chr	CNVR counts	Total CNVR length (kb)	Average size (kb)	Chromosome coverage (%)	Gene counts	Genic CNVR counts	Intergenic CNVR counts
1	123	8300378	67482.75	3.0%	280	96	27
2	92	7947660	83670.05	3.2%	232	75	17
3	135	16229424	113500.4	7.2%	530	111	24
4	46	3850077	81935.06	3.2%	120	36	10
5	73	6103018	83602.99	5.7%	334	64	9
6	25	3505082	140203.3	3.0%	89	18	7
7	38	2065043	54343.24	2.1%	102	33	5
8	20	2576134	128806.7	2.8%	37	14	6
9	22	2770076	125912.6	2.9%	101	13	9
10	33	3714767	109261.8	4.3%	76	22	11
11	85	10386612	122195.4	16.7%	478	82	3
12	34	5175925	143813	6.6%	189	31	3
13	40	3354010	69875.21	4.0%	143	39	1
14	86	5745649	66044.67	9.2%	280	73	13
15	57	3869565	66721.45	4.8%	142	46	11
16	26	2030652	75223.78	2.8%	54	13	13
17	34	3936480	109361.9	5.5%	195	30	4
18	51	5569999	109215.7	8.1%	194	43	8
19	54	4130712	76494.67	6.8%	162	42	12
20	43	3166193	73632.4	6.2%	88	31	12
21	47	4662020	99191.91	9.3%	229	39	8
22	26	2311189	88891.88	4.6%	60	15	11
23	15	1008958	67263.87	1.6%	20	11	4
24	41	7643393	186424.2	18.2%	356	39	2
25	26	1055495	37756.86	2.3%	32	21	5
26	11	656429	59675.36	1.5%	15	9	2

Remarkably, compared with the number of gain events, we observed almost ten times more loss events and a longer loss length, with the number of regions with CNV losses and gains being 1,173 and 119, respectively. Both types were found in only five regions. For each CNVR, the relative frequency of animals with an overlapping CNV ranged from 2.1% to 37.5% (Additional file: Table S2). In addition, 553 CNVRs were present in only one animal, whereas most CNVRs (57.3%) were identified in two or more samples.

### Validation of the identified CNVRs

**Figure 3:**
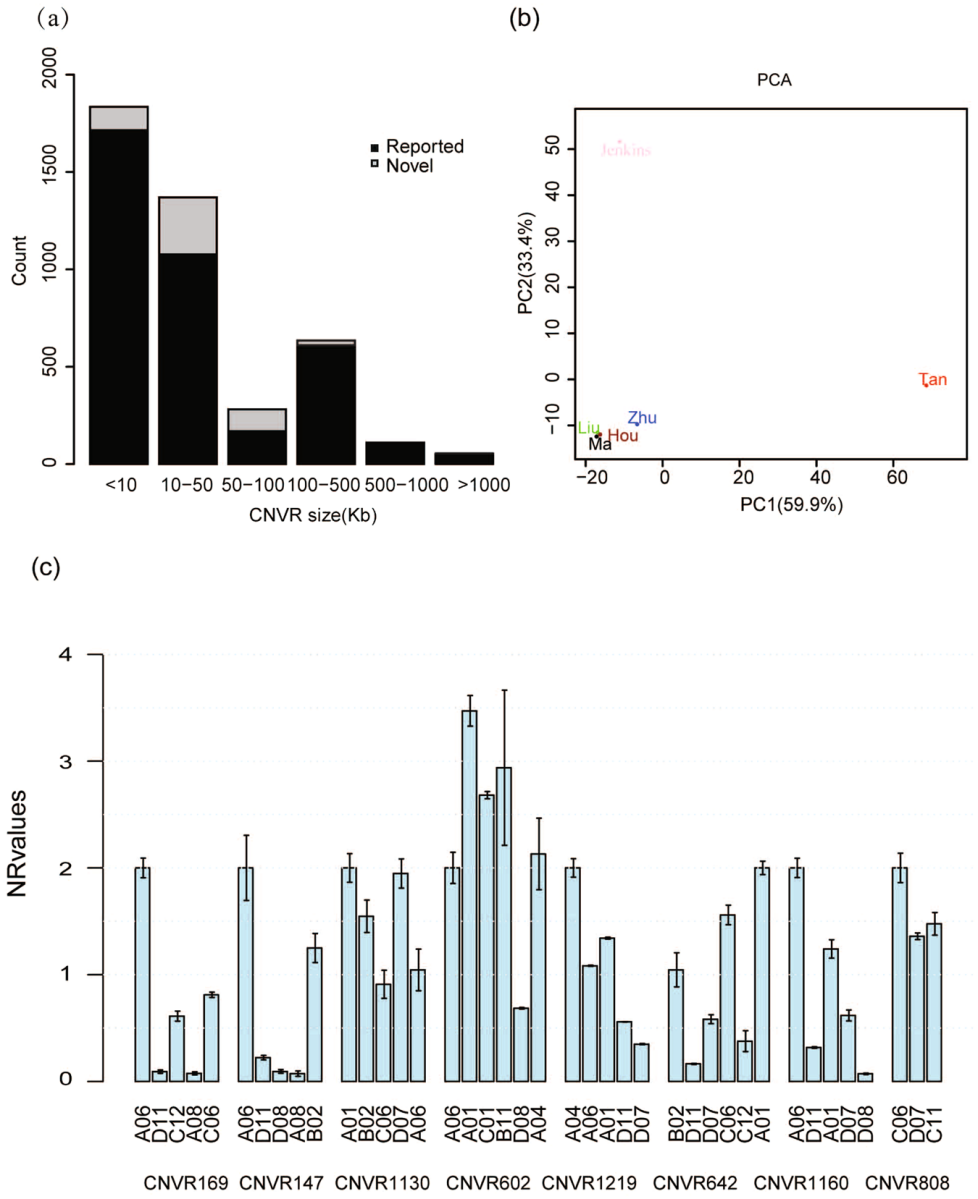
A summary of CNVRs identified in the previous study and in the current study. (**a**) Number of known and novel CNVRs identified in the present study; gray and black boxes indicate known and novel CNVRs, respectively. (**b**) PCA plot of different studies. (**c**) qPCR validation of eight selected CNVRs.

Based on five previous reports using various platforms to detect CNVs in different breeds of sheep, a total of 4,321 CNVRs (Additional file: Table S3) were obtained^9–13^. When comparing the novel CNVRs to the previously identified CNVRs, we found 740 (57.1%) overlapping regions (Fig. 3a). Interestingly, the majority of the previously identified CNVRs detected in our study were large CNVRs (>100 kb) (Fig. 3a). The total length of the novel CNVRs corresponded to 20.7 Mb of the genomic area. There were 340, 340, 10, 15, and 187 CNVRs that overlapped with those described by Zhu *et al*.^9^, Jenkins *et al*.^13^, Ma *et al*.^11^, Hou *et al*.^12^, and Liu *et al*.^10^, respectively (Table 2). To investigate the differences in distribution patterns between the five studies, we performed principle component analysis (PCA) based on the composite CNVR dataset (Fig. 3b). PCA showed that PC1 distinguished our study from others and that PC2 distinguished our study from Jenkins’ study; the other four studies clustered together (Fig. 3b). The hierarchical clustering results showed the same tendency (Additional file: Figure S1).

**Table 2:** Comparison of our study with five recent ovine CNV reports using various platforms.

	This study	Jenkins 2016	Zhu 2016	Hou 2015	Ma 2015	Liu 2013
**platform**	SNP600	aCGH	SNP600	aCGH	SNP50	SNP50
**sample/breed**	48	30	120	5	160	100
**CNVR count**	1296	3488	490	51	111	238
**CNVR range**	1 Kb–2.3 Mb	1 Kb–3.6 Mb	100 Kb–805 Kb	52 Kb–21.1 Mb	14 Kb–567 Kb	14 Kb–1.3 Mb
**CNVR gain**	119	n.a.	93	23	99	13
**CNVR loss**	1173	n.a.	390	21	12	219
**CNVR both**	5	n.a.	7	7	0	6
**Median size**	46 Kb	8 Kb	133 Kb	117 Kb	101 Kb	187 Kb
**Mean size**	93 Kb	19 Kb	165 Kb	89 Kb	124 Kb	254 Kb
**Genome coverage**	4.7%	2.7%	3.3%	0.6%	4.8%	2.3%
**overlapping with this study**	n.a.	340	340	15	10	187
**CNVRs overlapped within genes**	81%	59%	81.06–90.90%	100%	n.a.	53.36%

As many as 556 (42.9%) CNVRs identified in our study are novel (Fig. 3a). To verify the accuracy of our prediction of the novel CNVRs, quantitative real-time PCR (qPCR) was used to validate ten randomly selected CNVRs from our study (i.e., CNVRs #16, 147, 169, 602, 642, 660, 808, 1130, 1160, and 1219) (Fig. 3c). These CNVRs represent three predicted statuses (losses, gains and both) for CNVRs, with frequencies ranging from low to high. Eight (80%) of the selected CNVRs were successfully confirmed. As shown in Fig. 3c, a normalized ratio (NR) of approximately 2 indicates a normal status (no CNV); an NR of approximately 1 or 0 indicates one or two copies deleted, and an NR of approximately 3 or above indicates one or more copies gained. Each CNVR had a reference sample, in which a CNV was not detected, and a reference gene, which also did not contain a CNV. The correlation between our CNV prediction and PCR validation was highly significant (P = 8.92E-12) (Additional file: Figure S2). Details of the primers and results are listed in the supporting information (Additional file: Table S4).

The reference genome used in this study, Oar_v3.1, has 21,585 gaps larger than 1 kb^16^. As stated earlier, we identified 5,190 CNVs, which accounted for 4.7% of the entire ovine genome. Among these, 463 (8.9%) overlapped with 1,255 gaps, indicating that the majority (90%) of the CNVs that we identified are located within the gap-free genomic region. Among the 463 CNVs that overlap with gaps, 140 (>30%) were confirmed by other studies. Of the eight randomly selected CNVs for qPCR validation, four overlapped with gaps and were verified by qPCR, suggesting that these gap-overlapping CNVs are also high-confidence CNVs.

### Functional annotation of the identified CNVRs

**Figure 4:**
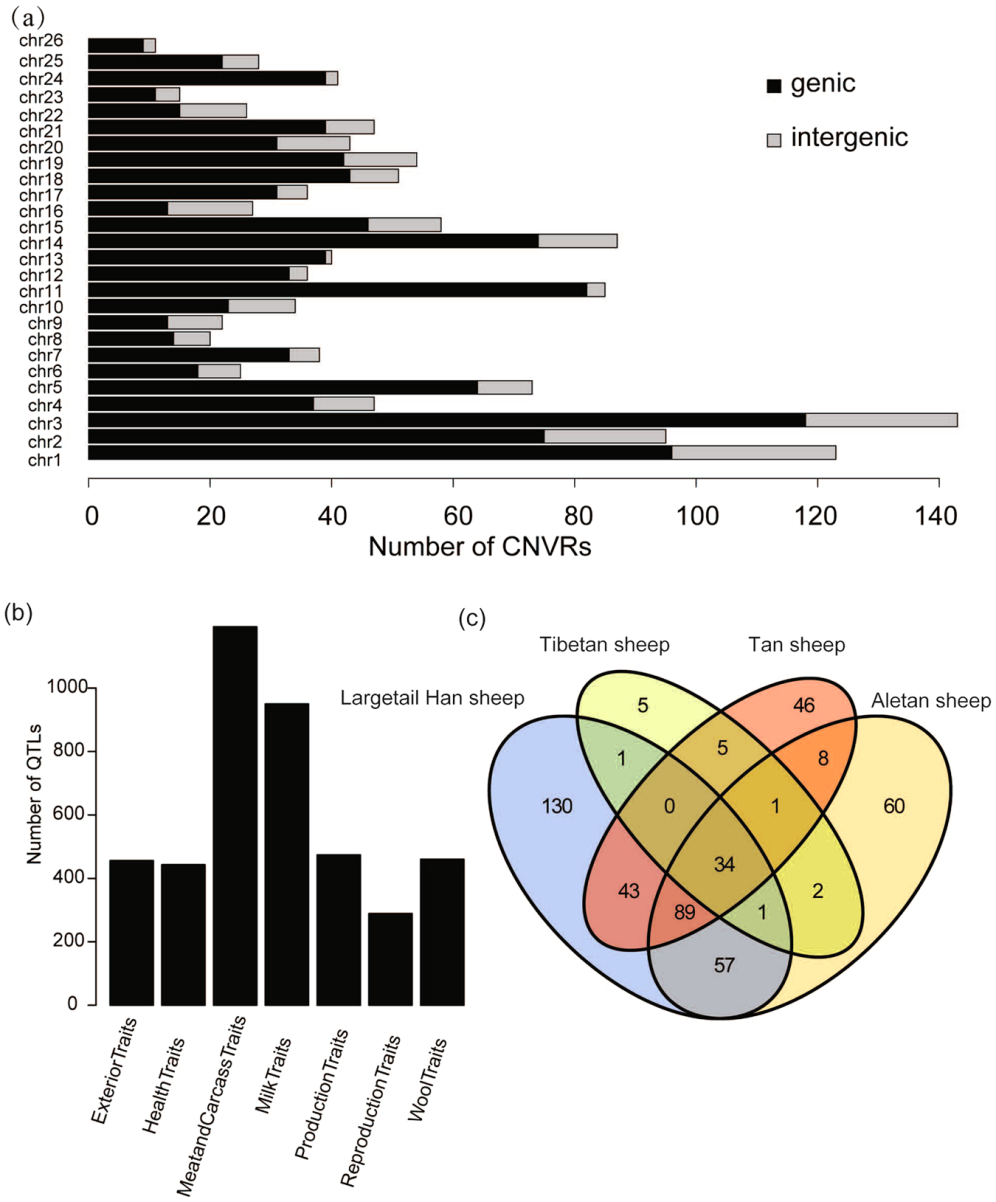
CNVR-harboring genes. (**a**) Number of CNVRs located within the genic and intergenic regions; black and gray indicate the genic and intergenic CNVRs, respectively. (**b**) Number of genes located in different QTL categories. (**c**) Venn diagram of CNVR numbers in four different Chinese indigenous sheep breeds.

The BioMart system in ENSEMBL (http://www.biomart.org/) was used to retrieve the gene content in the 1,296 CNVRs (1173 losses, 119 gains and 5 both). The 81% of our CNVRs overlap with 4,541 genomic genes (Additional file: Table S2), which are mostly located inside CNVRs (Fig. 4a). Among these genes, 89.9% are protein-coding genes, 3.5% microRNAs (miRNAs), 3.2% lncRNAs, and others pseudogenes, processed pseudogenes, small nucleolar (snoRNA) genes, and miscRNAs.

Interestingly, we found that these CNVR-harboring genes are significantly enriched for lipid metabolism (P = 0.001) and GTPase activity (P = 4.63E-07). Kyoto Encyclopedia of Genes and Genomes (KEGG) analysis showed that the Notch signaling pathway, the MAPK signaling pathway and the VEGF signaling pathway to be significantly enriched (Additional file: Table S5). Furthermore, we found genes associated with lipid metabolism, including *PPARA* (*peroxisome proliferator-activated receptor-α*), *RXRA* (*retinoic X receptor A*), *KLF11* (*Kruppel-like factor 11*), *PPP1CA* (*phosphoprotein phosphatase 1 catalytic subunit A*), and *PDGFA* (*platelet-derived growth factor alpha*), that were previously reported to overlap with CNVs in fat-tailed sheep^9^. We found a total of 1,094 CNVRs (84.4%) that overlap with at least one base with QTLs from the sheep Animal QTLdb (Fig. 4b and Additional file: Table S5). Most cases proved to be CNVRs residing in a QTL region.

### Potentially specific CNVRs in Tan sheep

Using the same 600 K ovine BeadChip platform, Zhu *et al*.^9^ identified 371, 370 and 66 CNVRs in large-tailed Han sheep, Altay and Tibetan sheep, respectively, which allowed us to identify the potentially breed-specific CNVRs for Tan sheep. According to the method described by Zhu *et al*.^9^, we filtered out CNVs smaller than 100 kb and obtained 303 CNVRs. The Venn diagram of the four different indigenous Chinese sheep breeds showed 46, 60, 130, and 5 potentially specific CNVRs in Tan sheep, Altay sheep, large-tailed Han sheep, and Tibetan sheep, respectively, which were sampled by both Zhu *et al*.^9^ and in our studies (Fig. 4c). GO analysis of genes overlapping with the 46 potentially specific CNVRs in Tan sheep showed significant enrichment of the homeobox (P = 9.6E-06), embryonic skeletal system morphogenesis (GO:0048704, P = 2.15E-08) and anterior/posterior pattern specification (GO:0009952, P = 2.5E-07) categories (Table 3). Interestingly, *DLX3*/*DLX4* (*homeobox protein DLX-3/DLX-4*), two well-known genes associated with hair development, belong to the most significantly enriched GO category of homeobox. Moreover, *HOXD12* (*homeobox protein Hox-D12*) and *TBX6* (*T-box transcription factor 6*), as well as *HOXB3* (*homeobox protein Hox-B3*), which is involved in embryonic skeletal system morphogenesis, overlap with the CNVRs potentially specific to Tan sheep.

**Table 3:** The enrichment of Go terms for the specific CNVRs in Tan sheep.

Category	Term	Count	PValue
UP_KEYWORDS	Homeobox	11	9.60E-06
UP_KEYWORDS	Developmental protein	19	1.32E-04
UP_SEQ_FEATURE	short sequence motif:Antp-type hexapeptide	7	9.90E-09
UP_SEQ_FEATURE	DNA-binding region:Homeobox	11	1.01E-06
UP_SEQ_FEATURE	domain:Chromo	3	0.006223
UP_SEQ_FEATURE	domain:FAD-binding FR-type	3	0.007754
GOTERM_BP_DIRECT	GO:0048704~embryonic skeletal system morphogenesis	8	2.15E-08
GOTERM_BP_DIRECT	GO:0009952~anterior/posterior pattern specification	9	2.50E-07
GOTERM_BP_DIRECT	GO:0016925~protein sumoylation	6	0.001729
GOTERM_BP_DIRECT	GO:0050665~hydrogen peroxide biosynthetic process	3	0.001969
GOTERM_BP_DIRECT	GO:0043687~post-translational protein modification	10	0.008192
GOTERM_BP_DIRECT	GO:0060324~face development	3	0.009843
GOTERM_CC_DIRECT	GO:0035102~PRC1 complex	4	8.30E-05
GOTERM_CC_DIRECT	GO:0031519~PcG protein complex	4	9.09E-04
GOTERM_MF_DIRECT	GO:0035064~methylated histone binding	5	4.97E-04
GOTERM_MF_DIRECT	GO:0003700~transcription factor activity, sequence-specific DNA binding	15	0.0034
GOTERM_MF_DIRECT	GO:0043565~sequence-specific DNA binding	9	0.008613
GOTERM_MF_DIRECT	GO:0019899~enzyme binding	8	0.009435
KEGG_PATHWAY	hsa01200:Carbon metabolism	5	0.007425
KEGG_PATHWAY	hsa04151:PI3K-Akt signaling pathway	8	0.009275

## Discussion

Using the high-density 600 K SNP arrays, this study identified more than 5,000 autosomal CNVs in 48 animals and grouped them into 1,296 CNVRs in the sheep genome, thereby providing a genome-wide view of CNVs in the Chinese Tan sheep genome. The number of identified CNVRs is larger than that reported by two existing ovine 50 K SNP array-based CNV studies, in which 111 and 238 CNVRs were detected (Table 2). This difference is not surprising, as the current study used a high-density SNP array containing more than 600 K probes, whereas Ma *et al*.^11^ and Liu *et al*.^10^ used low-density SNP arrays with 50 K probes. Thus, more than 10 times better resolution was achieved by this study than the earlier two SNP array studies, which also resulted in the smaller mean/median length of CNVRs (Table 2). In fact, a significant improvement in CNVR detection in sheep has been previously achieved by increasing the aCGH probe-spacing resolution from 385 K to 2.1 million probes^13^. In addition to probe-spacing resolution in the genome, the applied filtering process can also affect CNV detection. This was reflected by the comparison of the current study to a previous 600 K SNP array-based CNV study, in which Zhu *et al*.^9^ filtered out the small CNVs (less than 100 kb in size) for CNV detection. As a result, Zhu *et al*.^9^ identified half as many CNVRs as the present study, and the median size of the CNVRs they detected was twice as large as that of our study. As expected, when we applied the same filtration process used by Zhu *et al*.^9^, a CNVR overlap of nearly 60% was reached, which is much higher compared to that found in the previous four studies (Table 2).

The 1,296 autosomal CNVRs reported in this study account for approximately 4.7% of the entire ovine genome. This estimate is similar to the range reported in horses^18^, pigs^19^, cattle^24^, and humans^25^ (0.8% to 5.0%). The estimate is still higher, even when compared with a comprehensive CGH array-based CNV study that covered 2.7% of the ovine genome^13^. This difference could be due to the underestimation of sheep CNVs by Jenkins *et al*.^13^, in which a cattle reference genome was used for the probe design and, thus, sheep CNVs in regions that were deleted in or of low homology with the reference genome were likely ignored. The difference could also be due to the overestimation by the CNV calling algorithm, PennCNV, in our study. The current SNP array-based detection of CNVs remains prone to false positives and shows low concordance between multiple calling algorithms^26^. Although PennCNV software is widely used for Illumina SNP arrays^27^, particularly for high-density SNP arrays^9^, a certain proportion of false positives exists in our findings. SNP arrays may also miss CNVs because their SNP chip coverage shows inherent bias against the genomic regions harboring CNVs^20^. For example, segmental duplications (SDs), one of the catalysts and hotspots for CNV formation, are often affected by low probe density due to the difficulties of array design^28^. Furthermore, common CNVs may cause SNPs to be rejected when the SNPs deviate from Mendelian inheritance and the Hardy-Weinberg equilibrium^20^.

Of the 1,296 identified CNVRs, more losses than gains were observed. This imbalance was also observed in reports by Zhu *et al*.^9^ and Liu *et al*.^10^ (Table 2) and is commonly reported in the literature^29^. This large disparity between deletions and duplications can be biased by the high sensitivity to loss events of the CNV calling algorithm and the lack of a Chinese sheep population during the SNP array design. If this bias can be avoided and the high ratio between loss and gain in Chinese native sheep breeds is still found, then this high ratio could be due to genomic differences between foreign breeds and Chinese breeds. Therefore, new strategies (for example, sequencing-based CNV detection) and large Chinese sheep populations are needed to investigate the high loss/gain ratio in future studies.

Notably, 57.1% of the CNVRs detected in this study can be confirmed by other published studies^9–13^ and 80% of the randomly selected novel CNVRs were further confirmed by qPCR, indicating the accuracy of our CNV detection using a high-density SNP chip in the sheep genome. Currently, three algorithms for CNV detection based on SNP arrays have been developed, which are available in different programs, including PennCNV^30^, cnvPartition (http://www.illumina.com/documents/products/technotes/technote_cnv_algorithms.pdf) and QuantiSNP^31^. According to a previous comprehensive assessment of multiple CNV calling algorithms for array-based detection, the concordance of these algorithms is low^26^. The common set of CNVs detected by multiple algorithms may avoid bias to some extent but may also miss many false negatives, whereas the union set may contain a large number of false positives. This makes it difficult to determine the appropriate number of CNVs, as summarized by Winchester *et al*.^20^. According to the number of citations in PubMed, PennCNV software is currently the most widely used for Illumina chips (PennCNV: 955 citations; QuantiSNP: 432 citations)^27^, particularly for high-density SNP data^9^. Compared to other algorithms such as CNVPartition and QuantiSNP, PennCNV is more reliable for assessing the number of copies when using Illumina high-density arrays because it incorporates the allelic intensity ratio at each SNP marker and the total signal intensity, the allele frequency of SNPs, the distance between neighboring SNPs, and the GC content to overcome biases^32^. In this study, we used high-density SNP arrays and stricter filtering criteria (SD of LRR < 0.30 and BAF = 0.01) to reduce the rate of false-positive results, resulting in a qPCR confirmation percentage of 80%. We also found a good correlation between our CNV prediction and qPCR validation (P = 9.0E-12; R = 0.7105; Figure S2). The discrepancy between PennCNV and qPCR validation could represent false negatives in qPCR amplification due to the ambiguous boundaries of CNVs. The uncertain boundaries may lead to placing the qPCR primers outside the actual CNVs, whereas the potential impacts of SNPs/small indels may affect the specific binding of primers to the CNV region for some individuals^17^ or could be false positives in the CNV detection by PennCNV in our study.

More than 80% of the CNVRs identified in this article span 1,094 QTLs (Table S6) belonging to seven categories: meat and carcass traits, milk traits, production traits, exterior traits, reproduction traits, health traits, and wool traits. Tan sheep is one of the most important sheep breeds in China because the pelts have special curly fleece after birth, but the animal gradually loses this phenotype with age^23^. To identify Tan sheep-specific CNVRs underlying this unique phenotype, the current study was compared with that by Zhu *et al*.^9^ and 46 potentially breed-specific CNVRs in Tan sheep were obtained (Table S7). GO analysis of the CNVR-harboring genes showed the most significant enrichment in homeobox proteins (P = 9.6E-06), which have been previously shown to play key roles during fetal development in humans^33^. Among the homeobox transcription factors, *DLX3* is essential for hair morphogenesis, differentiation and cycling programs^34^. The mutation of this gene is associated with tricho-dento-osseous syndrome, which is characterized by curly and kinky hair at infancy that later straightens^35^. Interestingly, this is consistent with the unique phenotype in Tan sheep (i.e., curly fleece after birth that disappears with age). Previous studies have shown that SNPs in the 3′UTR and promoter regions of *DLX3* have a significant effect on wool curvature in Chinese Merino sheep^36–38^. Therefore, this candidate gene is worthy of validation for its functional relevance to the special curly fleece in Tan sheep. In addition to the unique trait of curly fleece, Tan sheep exhibits the common fat-tail phenotype, as do other sheep indigenous to China, for adaptation to the dry (average annual precipitation <400 mm) and cold (average annual temperature is 4 °C) climate. Thus, by comparison with the previous CNV study of the two typical Chinese fat-tail breed^9^, we found that the same set of CNVR-harboring genes involved in lipid metabolism and the same GO category of lipid metabolism were significantly enriched (P = 0.001), indicating that the identified CNVRs are also likely associated with the fat-tail phenotype in Tan sheep.

## Materials and Methods

### Sample preparation

Blood samples were randomly collected from 48 unrelated Tan sheep (6 rams and 42 ewes) from multiple flocks in Ningxia province. Each sheep was carefully confirmed to match the phenotypic characteristics of the Tan sheep breed. Genomic DNA was extracted from blood using the Promega Wizard Genomic DNA Purification Kit (Promega, Madison, Wisconsin, USA) according to the standard protocol provided by the manufacturer. A NanoDrop 2000 was used to measure the purity and concentration of the genomic DNA.

Procurement of peripheral blood was performed according to the guidelines for the care and use of experimental animals established by the ethics committees of the Ministry of Agriculture of People’s Republic of China. All of the animal experiments were approved by the Chinese Academy of Agricultural Sciences (CAAS) (Beijing, China).

### Genotyping and quality control

According to the manufacturer’s protocols (Illumina, San Diego, California, USA), all genomic DNA samples from 48 sheep were genotyped using the Illumina *Ovine* SNP600K BeadChip array, which contains oligo probes for 685,734 SNPs, with the majority (80%) equally spanning the ovine genome, 10% reported in the literature as functional variants, 7% overlapping with the Illumina *Ovine* SNP50K array, and 3% accessible using a genotyping-by-sequencing protocol^39^. The raw data were extracted using GenomeStudio (Illumina) and strict quality control was used for SNP filtering to increase the accuracy of the CNV detection. First, we removed individuals in which the call rate was <90%. Second, we discarded SNPs with a >10% missing genotype and a minor allele frequency (MAF) <0.05. Third, we removed SNPs that severely deviated from Hardy-Weinberg equilibrium (multiple test-adjusted P < 10^−5^) within each population. In addition, identical-by-descent (IBD) was conducted using Plink software. Finally, 495,786 autosomal SNPs were subjected to the subsequent CNV detection and analysis. The X and Y chromosomes were excluded^40^.

### Genome-wide detection of CNVs and CNVRs

CNVs were detected using a hidden Markov Model (PennCNV, http://www.openbioinformatics.org/penncnv/), which allows for the detection of CNVs based on Illumina or Affymetrix SNP chip data. Illumina GenomeStudio software can export the signal intensity data of the log R ratio of R (LRR) and B allele frequency (BAF) for each SNP. The population frequency of B allele (PFB) file was calculated based on the average BAF of each marker in the population. The PennCNV algorithm^30^ was only applied to autosomes (command: -lastchr 26) to identify individual-based CNVs. To increase the confidence of the detected CNVs, quality control was performed by employing standard exclusions of the LRR (standard deviation of LRR) <0.3, a BAF drift <0.01 and a waviness factor <0.05. We classified the status of these CNV into two categories: “loss” (CNV containing a deletion) and “gain” (CNV containing a duplication).

The CNVRs were determined by aggregating the overlapping CNVs (with at least 1-bp of overlap) that were identified across all of the samples, according to previously reported methods^41, 42^. We removed the CNVRs that were less than 1 kb, as CNVs were defined as fragments ranging from 1 kb to several Mbs and having a variable copy number in comparison to with reference genome^1^. To further support the PCA results, a Hierarchical clustering analysis^43^ for all published studies was performed according to their CNVR distribution.

### Functional enrichment analysis of CNVR-harboring genes

BioMart (http://www.biomart.org/) in the Ensembl database was employed to identify genes located within or partially overlapping with the identified CNVRs. CNVRs that overlapped with the gene’s coding region by at least 1 bp were used to calculate the proportion of CNVR overlapping genes. Functional annotation was performed in DAVID (http://david.abcc.ncifcrf.gov/) for GO terms and KEGG pathway analyses. Because the sheep genome annotation is limited, the ovine Ensembl gene IDs were converted into orthologous human Ensembl gene IDs for the functional enrichment analysis. Furthermore, these CNVRs were mapped to the sheep QTLs from the Animal QTL database (http://www.animalgenome.org/cgi-bin/QTLdb/OA/browse).

### Comparison with previous studies

To evaluate the reliability of the CNVRs detected, we compared our results to five existing studies of sheep CNVs detected using various platforms and involving different breeds^9–13^. The CNVRs were compared according to our previous paper^17^. To compare the CNVRs among sheep breeds with different types of tails under the same condition (including the same platforms, analysis methods and filtering parameters), we removed all CNVs less than 100 kb according to the technique described by Zhu *et al*.^9^.

### qPCR validation of CNVRs

We performed qPCR analysis on ten random selected genomic regions harboring CNVs identified in this study on an ABI7500 (Applied Biosystems by Life Technologies, Darmstadt, Germany) sequence detection system. The primers (Additional file: Table S4) were designed using Primer Premier 6 software (Premier Company) and were based on NCBI reference sequences. The genomic DNAs of the same individual used in the Illumina Chip genotyping were used for the experimental validation. The two normal copies of *DGAT2* in the ovine genome were used as reference genes according to our own study and a previous study^10^. Four samples in which a normal copy number was identified in the target regions were used as reference samples. PCR experiments were conducted using Power SYBR Green PCR Reagent Kit (Applied Biosystems). The qPCR conditions were as follows: 95 °C for 3 min, followed by 40 cycles of 95 °C for 15 s and 60 °C for 60 s. Three replications were performed for each sample. Fold changes were determined using a standard 2^−ΔΔCT^ method that compares the ΔC_T_ value of a reference sample with the sample of interest for the ΔΔC_T_ calculation and compares the C_T_ (cycle threshold) values of a reference gene to the gene of interest for the ΔC_T_ calculation. Fold changes were normalized to a diploid number for a better comparison of copy number in all qPCR plots.

## Electronic supplementary material

dataset1

## References

[1] Sebat J (2004). Large-scale copy number polymorphism in the human genome. Science.

[2] Pielberg G, Olsson C, Syvänen AC, Andersson L (2002). Unexpectedly high allelic diversity at the KIT locus causing dominant white color in the domestic pig. Genetics.

[3] Fontanesi L (2010). Genetic heterogeneity and selection signature at the KIT gene in pigs showing different coat colours and patterns. Anim. Genet.

[4] Wright, D. *et al*. Copy number variation in intron 1 of SOX5 causes the Pea-comb phenotype in chickens. *PLoS Genet*. **5**, doi:10.1371/journal.pgen.1000512 (2009). 10.1371/journal.pgen.1000512 PMC2685452 19521496

[5] Elferink, M.G., Vallée, A.A., Jungerius, A.P., Crooijmans, R.P. & Groenen, M.A.M. Partial duplication of the PRLR and SPEF2 genes at the late feathering locus in chicken. *BMC Genomics* **9**, doi:10.1186/1471-2164-9-391 (2008). 10.1186/1471-2164-9-391 PMC2542384 18713476

[6] Imsland F (2016). Regulatory mutations in TBX3 disrupt asymmetric hair pigmentation that underlies Dun camouflage color in horses. Nat. Genet..

[7] Lv FH (2015). Mitogenomic meta-analysis identifies two phases of migration in the history of eastern Eurasian sheep. Mol. Biol. Evol..

[8] Fontanesi L (2011). A first comparative map of copy number variations in the sheep genome. Genomics.

[9] Zhu, C. *et al*. Genome-wide detection of CNVs in Chinese indigenous sheep with different types of tails using ovine high-density 600 K SNP arrays. *Sci. Rep*. **6**, doi:10.1038/srep27822 (2016). 10.1038/srep27822 PMC4901276 27282145

[10] Liu, J. *et al*. Analysis of copy number variations in the sheep genome using 50 K SNP BeadChip array. *BMC Genomics* **14**, doi:10.1186/1471-2164-14-229 (2013). 10.1186/1471-2164-14-229 PMC3626776 23565757

[11] Ma Y, Zhang Q, Lu Z, Zhao X, Zhang Y (2015). Analysis of copy number variations by SNP50 BeadChip array in Chinese sheep. Genomics.

[12] Hou CL (2015). Genome-wide analysis of copy number variations in Chinese sheep using array comparative genomic hybridization. Small Ruminant Res.

[13] Jenkins, G.M. *et al*. Copy number variants in the sheep genome detected using multiple approaches. *BMC Genomics* **17**, doi:10.1186/s12864-016-2754-7 (2016). 10.1186/s12864-016-2754-7 PMC4898393 27277319

[14] Norris BJ, Whan VA (2008). A gene duplication affecting expression of the ovine ASIP gene is responsible for white and black sheep. Genome Res..

[15] Fontanesi L, Dall’Olio S, Beretti F, Portolano B, Russo V (2011). Coat colours in the Massese sheep breed are associated with mutations in the agouti signalling protein (ASIP) and melanocortin 1 receptor (MC1R) genes. Animal.

[16] Jiang Y (2014). The sheep genome illuminates biology of the rumen and lipid metabolism. Science.

[17] Dong K (2015). Copy number variation detection using SNP genotyping arrays in three Chinese pig breeds. Anim. Genet..

[18] Kader A (2016). Identification of copy number variations in three Chinese horse breeds using 70 K single nucleotide polymorphism BeadChip array. Anim. Genet..

[19] Wang, J. *et al*. A genome-wide detection of copy number variations using SNP genotyping arrays in swine. *BMC Genomics* **13**, doi:10.1186/1471-2164-13-273 (2012). 10.1186/1471-2164-13-273 PMC3464621 22726314

[20] Winchester L, Yau C, Ragoussis J (2009). Comparing CNV detection methods for SNP arrays. Brief. Funct. Genomic. Proteomic.

[21] Rui Z (2010). Correlation between KRT1. 2 gene and properties of lamb fur qualities of Tan sheep in Ningxia. J. Agric. Sci..

[22] Lijuan Y (2010). Correlation between KAP1. 3 gene and fur quality characteristics in Ningxia Tan sheep. Journal of Ningxia University (Natural Science Edition).

[23] Kang, X. *et al*. Transcriptome profile at different physiological stages reveals potential mode for curly fleece in Chinese tan sheep. *PLoS One* **8**, doi:10.1371/journal.pone.0071763 (2013). 10.1371/journal.pone.0071763 PMC3753335 23990983

[24] Hou, Y. *et al*. Genomic characteristics of cattle copy number variations. *BMC Genomics* **12**, doi:10.1186/1471-2164-12-127 (2011). 10.1186/1471-2164-12-127 PMC3053260 21345189

[25] McCarroll SA (2008). Integrated detection and population-genetic analysis of SNPs and copy number variation. Nat. Genet..

[26] Pinto D (2011). Comprehensive assessment of array-based platforms and calling algorithms for detection of copy number variants. Nat. Biotechnol..

[27] Mace A (2016). New quality measure for SNP array based CNV detection. Bioinformatics.

[28] Xu L, Hou Y, Bickhart D, Song J, Liu G (2013). Comparative analysis of CNV calling algorithms: literature survey and a case study using bovine high-density SNP data. Microarrays.

[29] Doan, R. *et al*. Whole-genome sequencing and genetic variant analysis of a quarter horse mare. *BMC Genomics* **13**, doi:10.1186/1471-2164-13-78 (2012). 10.1186/1471-2164-13-78 PMC3309927 22340285

[30] Wang K (2007). PennCNV: an integrated hidden Markov model designed for high-resolution copy number variation detection in whole-genome SNP genotyping data. Genome Res..

[31] Colella S (2007). QuantiSNP: an Objective Bayes Hidden-Markov Model to detect and accurately map copy number variation using SNP genotyping data. Nucleic Acids Res..

[32] G. M (2011). Assessment of copy number variation using the Illumina Infinium 1 M SNP-array: a comparison of methodological approaches in the Spanish Bladder Cancer/EPICURO study. Human Mutat..

[33] Mavrogiannis LA (2001). Haploinsufficiency of the human homeobox gene ALX4 causes skull ossification defects. Nat. Genet..

[34] Hwang J, Mehrani T, Millar SE, Morasso MI (2008). Dlx3 is a crucial regulator of hair follicle differentiation and cycling. Development.

[35] Al-Batayneh, O.B. Tricho-dento-osseous syndrome: diagnosis and dental management. *Int. J. Dent*. **2012**, doi:10.1155/2012/514692 (2012). 10.1155/2012/514692 PMC3434396 22969805

[36] Pei W (2013). Promoter characterization of sheep Dlx3 gene and association of promoter polymorphisms with wool quality traits in Chinese Merino. Scientia Agricultura Sinica.

[37] Rong EG (2012). Polymorphism in 3′UTR of DLX3 gene and its association with wool quality traits in Chinese merino sheep. Chinese Journal of Animal and Veterinary Sciences.

[38] Rong, E. *et al*. Functional characterization of a single nucleotide polymorphism in the 3′untranslated region of sheep DLX3 gene. *PLoS One* **10**, doi:10.1371/journal.pone.0137135 (2015). 10.1371/journal.pone.0137135 PMC4558038 26332462

[39] Anderson, R. Development of a high density (600 K) Illumina Ovine SNP Chip and its use to fine map the yellow fat locus. In *Plant and Animal Genome XXII Conference* (Plant and Animal Genome, 2014).

[40] Raudsepp T, Chowdhary BP (2008). The horse pseudoautosomal region (PAR): characterization and comparison with the human, chimp and mouse PARs. Cytogenet. Genome Res..

[41] Feuk L, Carson AR, Scherer SW (2006). Structural variation in the human genome. Nat. Rev. Genet..

[42] Redon R (2006). Global variation in copy number in the human genome. Nature.

[43] Xie, J. *et al*. Identification of copy number variations in Xiang and Kele pigs. *PLoS One* **11**, doi:10.1371/journal.pone.0148565 (2016). 10.1371/journal.pone.0148565 PMC4740446 26840413

