# Supplementary material for: Genome-wide detection of copy number variation in Chinese indigenous sheep using an ovine high-density 600 K SNP array
Source: Sci Rep. 2017 Apr 19;7:912. doi: 10.1038/s41598-017-00847-9 (PMC5430420; doi:10.1038/s41598-017-00847-9)
Supplement: Supplementary file 1 — dataset1 [file 41598_2017_847_MOESM1_ESM.docx]

**Genome-wide detection of copy number variation in Chinese indigenous sheep using an ovine high-density 600K SNP array**

Qing Ma^1^*, Xuexue Liu^2,3^*, JianfeiPan^2,3^, Lina Ma^1^, Yuehui Ma^2,3^，Xiaohong He^2,3^, Qianjun Zhao^2,3^, Yabin Pu^2,3^, Yingkang Li^1^& Lin Jiang^2,3^

^1^Institute of Animal Science, Ningxia Academy of Agriculture and Forestry Sciences, Yinchuan, Ningxia 75002, China. ^2^Institute of Animal Science, Chinese Academy of Agricultural Sciences (CAAS), No.2 Yuanmingyuan West Road, Beijing 100193, China. ^3^CAAS-ILRI Joint Laboratory on Livestock and Forage Genetic Resources, Institute of Animal Science, Chinese Academy of Agricultural Sciences (CAAS), No.2 Yuanmingyuan West Road, Beijing 100193, China.

*These authors contributed equally to this work.

Correspondence and requests for materials should be addressed to Y.L(email: nxnkycx@163.com) or L.J. (email: [jianglin@caas.cn](mailto:jianglin@caas.cn)) .

**Supporting information**

Additional supporting information may be found in the online version of this article.

**Table S1** CNVs identified in Tan sheep

**Table S2** CNVR distribution in Tan sheep

**Table S3** Merged CNVRs from five studies

**Table S4** qPCR primers used for validation

**Table S5** CNVRs that overlap with QTLs

**Table S6** Functional enrichment analysis of CNVR-overlapping genes

**Table S7** Specific CNVRs in Tan sheep

**Figure S1** Hierarchical clustering of CNVs detected in five existing studies

**Figure S2** Correlation between PennCNV prediction and qPCR validation


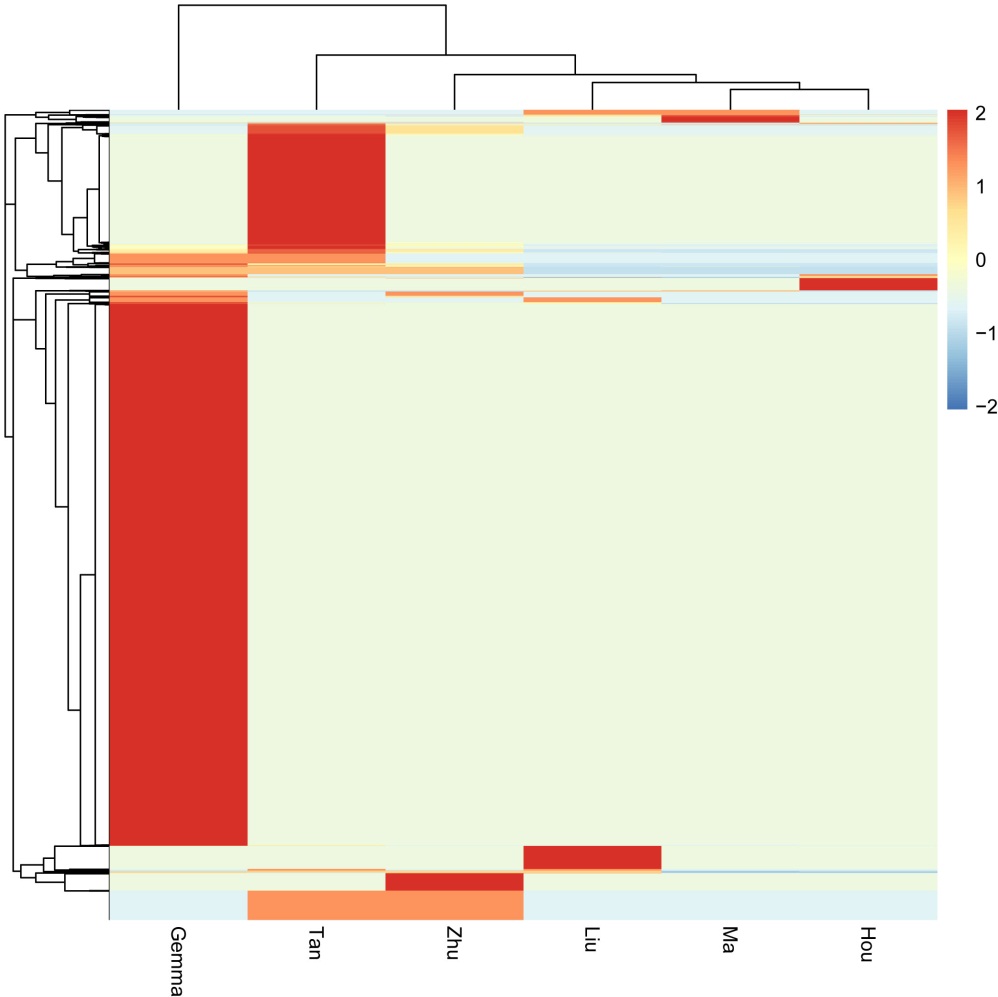

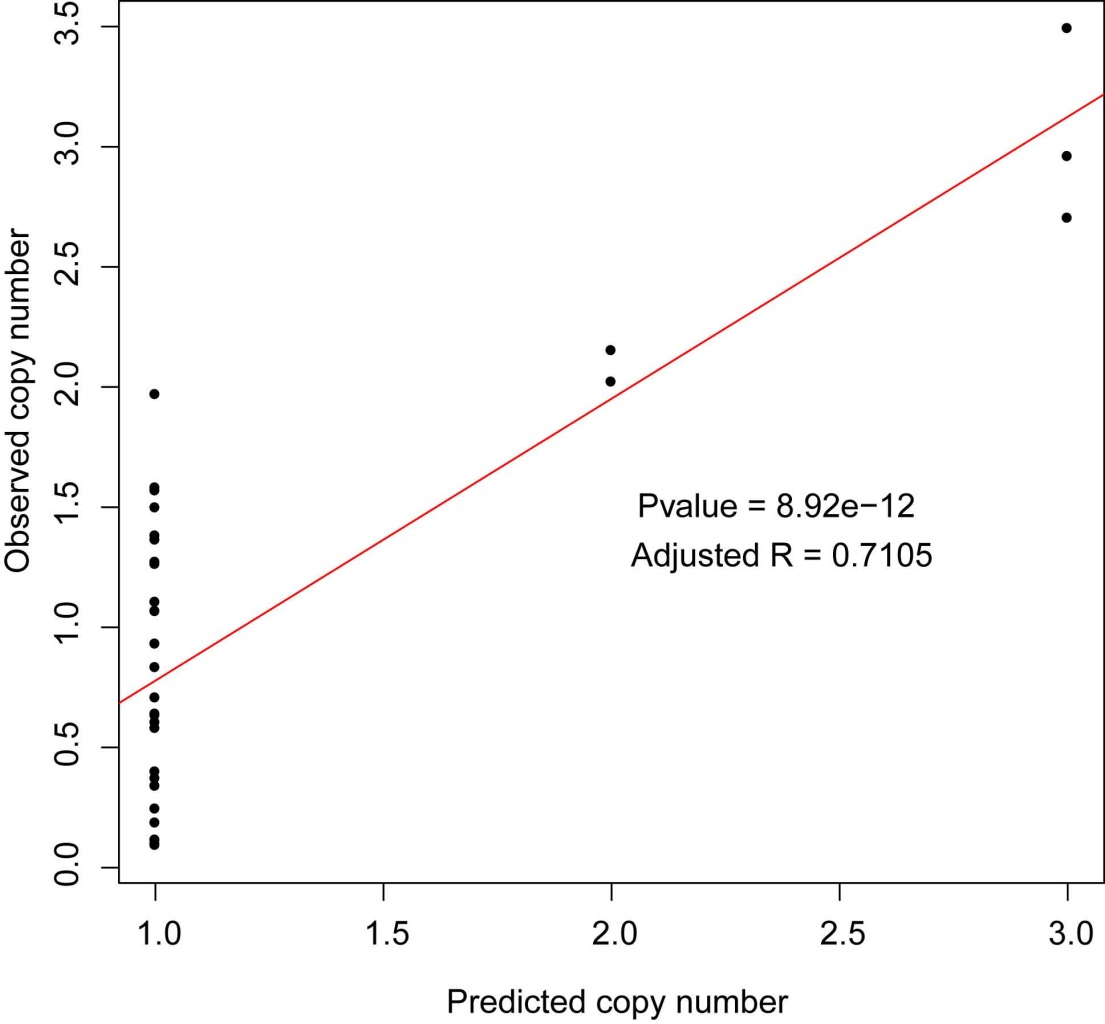


| **Table S1 CNV in Tan sheep** | | | | | |  |
| --- | --- | --- | --- | --- | --- | --- |
| Chr | Start | End | Numsnp | Length | startSNP | endSNP |
| 1 | 52854 | 310089 | 41 | 257236 | s64199.1 | oar3_OAR1_310089 |
| 1 | 62311 | 64468 | 4 | 2158 | oar3_OAR1_62311 | oar3_OAR1_64468 |
| 1 | 62311 | 76686 | 5 | 14376 | oar3_OAR1_62311 | oar3_OAR1_76686 |
| 1 | 62311 | 183304 | 23 | 120994 | oar3_OAR1_62311 | oar3_OAR1_183304 |
| 1 | 104800 | 250825 | 25 | 146026 | oar3_OAR1_104800 | oar3_OAR1_250825 |
| 1 | 104800 | 183304 | 16 | 78505 | oar3_OAR1_104800 | oar3_OAR1_183304 |
| 1 | 150205 | 179610 | 8 | 29406 | oar3_OAR1_150205 | oar3_OAR1_179610 |
| 1 | 150205 | 250825 | 18 | 100621 | oar3_OAR1_150205 | oar3_OAR1_250825 |
| 1 | 150205 | 250825 | 18 | 100621 | oar3_OAR1_150205 | oar3_OAR1_250825 |
| 1 | 201366 | 259883 | 9 | 58518 | oar3_OAR1_201366 | oar3_OAR1_259883 |
| 1 | 201366 | 310089 | 13 | 108724 | oar3_OAR1_201366 | oar3_OAR1_310089 |
| 1 | 250825 | 327204 | 9 | 76380 | oar3_OAR1_250825 | oar3_OAR1_327204 |
| 1 | 277602 | 695986 | 66 | 418385 | oar3_OAR1_277602 | oar3_OAR1_695986 |
| 1 | 277602 | 434493 | 32 | 156892 | oar3_OAR1_277602 | oar3_OAR1_434493 |
| 1 | 351221 | 373938 | 9 | 22718 | oar3_OAR1_351221 | oar3_OAR1_373938 |
| 1 | 401196 | 419642 | 7 | 18447 | OAR1_88143.1 | oar3_OAR1_419642 |
| 1 | 463433 | 501662 | 9 | 38230 | oar3_OAR1_463433 | oar3_OAR1_501662 |
| 1 | 463433 | 630221 | 27 | 166789 | oar3_OAR1_463433 | oar3_OAR1_630221 |
| 1 | 499014 | 560503 | 11 | 61490 | oar3_OAR1_499014 | oar3_OAR1_560503 |
| 1 | 533127 | 695986 | 20 | 162860 | oar3_OAR1_533127 | oar3_OAR1_695986 |
| 1 | 533127 | 630221 | 15 | 97095 | oar3_OAR1_533127 | oar3_OAR1_630221 |
| 1 | 589909 | 643628 | 10 | 53720 | oar3_OAR1_589909 | s34880.1 |
| 1 | 594318 | 630221 | 6 | 35904 | oar3_OAR1_594318 | oar3_OAR1_630221 |
| 1 | 754781 | 784057 | 7 | 29277 | oar3_OAR1_754781 | oar3_OAR1_784057 |
| 1 | 754781 | 852009 | 26 | 97229 | oar3_OAR1_754781 | oar3_OAR1_852009 |
| 1 | 754781 | 784057 | 7 | 29277 | oar3_OAR1_754781 | oar3_OAR1_784057 |
| 1 | 796379 | 935222 | 21 | 138844 | oar3_OAR1_796379 | oar3_OAR1_935222 |
| 1 | 810365 | 973690 | 26 | 163326 | oar3_OAR1_810365 | oar3_OAR1_973690 |
| 1 | 827883 | 990789 | 23 | 162907 | oar3_OAR1_827883 | oar3_OAR1_990789 |
| 1 | 832887 | 1144284 | 49 | 311398 | oar3_OAR1_832887 | oar3_OAR1_1144284 |
| 1 | 832887 | 935222 | 12 | 102336 | oar3_OAR1_832887 | oar3_OAR1_935222 |
| 1 | 840636 | 915659 | 9 | 75024 | oar3_OAR1_840636 | oar3_OAR1_915659 |
| 1 | 846954 | 852009 | 4 | 5056 | oar3_OAR1_846954 | oar3_OAR1_852009 |
| 1 | 912507 | 1056650 | 23 | 144144 | OAR1_537224_X.1 | oar3_OAR1_1056650 |
| 1 | 912507 | 935222 | 5 | 22716 | OAR1_537224_X.1 | oar3_OAR1_935222 |
| 1 | 966208 | 990789 | 7 | 24582 | oar3_OAR1_966208 | oar3_OAR1_990789 |
| 1 | 966208 | 976511 | 5 | 10304 | oar3_OAR1_966208 | oar3_OAR1_976511 |
| 1 | 1037519 | 1155400 | 26 | 117882 | oar3_OAR1_1037519 | s48804.1 |
| 1 | 1037519 | 1246467 | 39 | 208949 | oar3_OAR1_1037519 | oar3_OAR1_1246467 |
| 1 | 1039681 | 1246467 | 37 | 206787 | oar3_OAR1_1039681 | oar3_OAR1_1246467 |
| 1 | 1039681 | 1155400 | 24 | 115720 | oar3_OAR1_1039681 | s48804.1 |
| 1 | 1089734 | 1160510 | 19 | 70777 | oar3_OAR1_1089734 | oar3_OAR1_1160510 |
| 1 | 1089734 | 1180783 | 22 | 91050 | oar3_OAR1_1089734 | oar3_OAR1_1180783 |
| 1 | 1089734 | 1160510 | 19 | 70777 | oar3_OAR1_1089734 | oar3_OAR1_1160510 |
| 1 | 1123474 | 1246467 | 25 | 122994 | oar3_OAR1_1123474 | oar3_OAR1_1246467 |
| 1 | 1126017 | 1155400 | 11 | 29384 | oar3_OAR1_1126017 | s48804.1 |
| 1 | 1136626 | 1160510 | 7 | 23885 | oar3_OAR1_1136626 | oar3_OAR1_1160510 |
| 1 | 1550996 | 1563059 | 6 | 12064 | oar3_OAR1_1550996 | oar3_OAR1_1563059 |
| 1 | 1605666 | 1719331 | 24 | 113666 | oar3_OAR1_1605666 | oar3_OAR1_1719331 |
| 1 | 1618342 | 1719331 | 23 | 100990 | s38369.1 | oar3_OAR1_1719331 |
| 1 | 1628054 | 1666862 | 10 | 38809 | oar3_OAR1_1628054 | oar3_OAR1_1666862 |
| 1 | 1843965 | 1892471 | 14 | 48507 | oar3_OAR1_1843965 | oar3_OAR1_1892471 |
| 1 | 1857520 | 1877325 | 6 | 19806 | oar3_OAR1_1857520 | oar3_OAR1_1877325 |
| 1 | 2011394 | 2126668 | 25 | 115275 | oar3_OAR1_2011394 | oar3_OAR1_2126668 |
| 1 | 2036743 | 2047345 | 5 | 10603 | oar3_OAR1_2036743 | oar3_OAR1_2047345 |
| 1 | 2073681 | 2111220 | 10 | 37540 | oar3_OAR1_2073681 | oar3_OAR1_2111220 |
| 1 | 2073681 | 2126668 | 12 | 52988 | oar3_OAR1_2073681 | oar3_OAR1_2126668 |
| 1 | 2138737 | 2164128 | 10 | 25392 | oar3_OAR1_2138737 | oar3_OAR1_2164128 |
| 1 | 2146727 | 2165777 | 12 | 19051 | oar3_OAR1_2146727 | oar3_OAR1_2165777 |
| 1 | 2146727 | 2204741 | 21 | 58015 | oar3_OAR1_2146727 | oar3_OAR1_2204741 |
| 1 | 2186197 | 2207484 | 7 | 21288 | oar3_OAR1_2186197 | oar3_OAR1_2207484 |
| 1 | 2203962 | 2229206 | 13 | 25245 | s09433.1 | oar3_OAR1_2229206 |
| 1 | 2211654 | 2285769 | 23 | 74116 | oar3_OAR1_2211654 | oar3_OAR1_2285769 |
| 1 | 2211654 | 2225145 | 6 | 13492 | oar3_OAR1_2211654 | oar3_OAR1_2225145 |
| 1 | 2211654 | 2229206 | 10 | 17553 | oar3_OAR1_2211654 | oar3_OAR1_2229206 |
| 1 | 2221877 | 2346207 | 44 | 124331 | oar3_OAR1_2221877 | oar3_OAR1_2346207 |
| 1 | 2221877 | 2291703 | 23 | 69827 | oar3_OAR1_2221877 | oar3_OAR1_2291703 |
| 1 | 2221877 | 2229206 | 8 | 7330 | oar3_OAR1_2221877 | oar3_OAR1_2229206 |
| 1 | 2291703 | 2339635 | 19 | 47933 | oar3_OAR1_2291703 | oar3_OAR1_2339635 |
| 1 | 2316546 | 2339635 | 14 | 23090 | oar3_OAR1_2316546 | oar3_OAR1_2339635 |
| 1 | 2563995 | 2808944 | 54 | 244950 | oar3_OAR1_2563995 | s63837.1 |
| 1 | 2590671 | 2635118 | 10 | 44448 | oar3_OAR1_2590671 | oar3_OAR1_2635118 |
| 1 | 2605394 | 2735832 | 34 | 130439 | oar3_OAR1_2605394 | oar3_OAR1_2735832 |
| 1 | 2906205 | 2974590 | 18 | 68386 | s32107.1 | oar3_OAR1_2974590 |
| 1 | 2914442 | 2963230 | 12 | 48789 | oar3_OAR1_2914442 | oar3_OAR1_2963230 |
| 1 | 2919118 | 2974590 | 13 | 55473 | oar3_OAR1_2919118 | oar3_OAR1_2974590 |
| 1 | 2922702 | 2974590 | 11 | 51889 | oar3_OAR1_2922702 | oar3_OAR1_2974590 |
| 1 | 2937953 | 2995306 | 16 | 57354 | oar3_OAR1_2937953 | oar3_OAR1_2995306 |
| 1 | 2940195 | 2972533 | 7 | 32339 | oar3_OAR1_2940195 | oar3_OAR1_2972533 |
| 1 | 2960877 | 2974590 | 4 | 13714 | s16836.1 | oar3_OAR1_2974590 |
| 1 | 3114537 | 3127656 | 7 | 13120 | oar3_OAR1_3114537 | oar3_OAR1_3127656 |
| 1 | 3114537 | 3132186 | 9 | 17650 | oar3_OAR1_3114537 | s50787.1 |
| 1 | 3114537 | 3132186 | 9 | 17650 | oar3_OAR1_3114537 | s50787.1 |
| 1 | 3119384 | 3132186 | 8 | 12803 | oar3_OAR1_3119384 | s50787.1 |
| 1 | 3397235 | 3480553 | 17 | 83319 | oar3_OAR1_3397235 | oar3_OAR1_3480553 |
| 1 | 3844565 | 3868153 | 7 | 23589 | oar3_OAR1_3844565 | oar3_OAR1_3868153 |
| 1 | 3844565 | 3868153 | 7 | 23589 | oar3_OAR1_3844565 | oar3_OAR1_3868153 |
| 1 | 7534383 | 7541563 | 5 | 7181 | oar3_OAR1_7534383 | oar3_OAR1_7541563 |
| 1 | 7534383 | 7541563 | 5 | 7181 | oar3_OAR1_7534383 | oar3_OAR1_7541563 |
| 1 | 8067587 | 8087858 | 6 | 20272 | oar3_OAR1_8067587 | oar3_OAR1_8087858 |
| 1 | 8078843 | 8087858 | 4 | 9016 | oar3_OAR1_8078843 | oar3_OAR1_8087858 |
| 1 | 8078843 | 8087858 | 4 | 9016 | oar3_OAR1_8078843 | oar3_OAR1_8087858 |
| 1 | 10758793 | 10784179 | 9 | 25387 | oar3_OAR1_10758793 | oar3_OAR1_10784179 |
| 1 | 10758793 | 10775824 | 8 | 17032 | oar3_OAR1_10758793 | s62349.1 |
| 1 | 10764262 | 10775824 | 7 | 11563 | oar3_OAR1_10764262 | s62349.1 |
| 1 | 10764262 | 10784179 | 8 | 19918 | oar3_OAR1_10764262 | oar3_OAR1_10784179 |
| 1 | 10764262 | 10830834 | 14 | 66573 | oar3_OAR1_10764262 | oar3_OAR1_10830834 |
| 1 | 10764262 | 10784179 | 8 | 19918 | oar3_OAR1_10764262 | oar3_OAR1_10784179 |
| 1 | 10929980 | 10969407 | 10 | 39428 | oar3_OAR1_10929980 | oar3_OAR1_10969407 |
| 1 | 10929980 | 10981670 | 12 | 51691 | oar3_OAR1_10929980 | oar3_OAR1_10981670 |
| 1 | 10940829 | 10981670 | 10 | 40842 | oar3_OAR1_10940829 | oar3_OAR1_10981670 |
| 1 | 11105139 | 11111670 | 5 | 6532 | oar3_OAR1_11105139 | oar3_OAR1_11111670 |
| 1 | 12522110 | 12564454 | 13 | 42345 | oar3_OAR1_12522110 | oar3_OAR1_12564454 |
| 1 | 13898519 | 13949686 | 12 | 51168 | oar3_OAR1_13898519 | oar3_OAR1_13949686 |
| 1 | 13928793 | 13949686 | 7 | 20894 | oar3_OAR1_13928793 | oar3_OAR1_13949686 |
| 1 | 13928793 | 13945330 | 6 | 16538 | oar3_OAR1_13928793 | oar3_OAR1_13945330 |
| 1 | 14044540 | 14099180 | 17 | 54641 | oar3_OAR1_14044540 | OAR1_13970060.1 |
| 1 | 14165119 | 14189722 | 5 | 24604 | oar3_OAR1_14165119 | oar3_OAR1_14189722 |
| 1 | 15654925 | 15736001 | 18 | 81077 | oar3_OAR1_15654925 | oar3_OAR1_15736001 |
| 1 | 15724022 | 15736001 | 6 | 11980 | oar3_OAR1_15724022 | oar3_OAR1_15736001 |
| 1 | 15724022 | 15736001 | 6 | 11980 | oar3_OAR1_15724022 | oar3_OAR1_15736001 |
| 1 | 15728652 | 15737754 | 6 | 9103 | oar3_OAR1_15728652 | oar3_OAR1_15737754 |
| 1 | 17860628 | 17895291 | 14 | 34664 | oar3_OAR1_17860628 | oar3_OAR1_17895291 |
| 1 | 17860628 | 17898250 | 16 | 37623 | oar3_OAR1_17860628 | oar3_OAR1_17898250 |
| 1 | 17862637 | 17895291 | 13 | 32655 | oar3_OAR1_17862637 | oar3_OAR1_17895291 |
| 1 | 17862637 | 17895291 | 13 | 32655 | oar3_OAR1_17862637 | oar3_OAR1_17895291 |
| 1 | 17867080 | 17884650 | 10 | 17571 | oar3_OAR1_17867080 | oar3_OAR1_17884650 |
| 1 | 17869182 | 17895291 | 11 | 26110 | oar3_OAR1_17869182 | oar3_OAR1_17895291 |
| 1 | 17982237 | 18055873 | 23 | 73637 | oar3_OAR1_17982237 | oar3_OAR1_18055873 |
| 1 | 17982237 | 18018485 | 14 | 36249 | oar3_OAR1_17982237 | oar3_OAR1_18018485 |
| 1 | 17982799 | 18018485 | 12 | 35687 | oar3_OAR1_17982799 | oar3_OAR1_18018485 |
| 1 | 17995766 | 18055873 | 18 | 60108 | oar3_OAR1_17995766 | oar3_OAR1_18055873 |
| 1 | 18016184 | 18018485 | 5 | 2302 | oar3_OAR1_18016184 | oar3_OAR1_18018485 |
| 1 | 18041155 | 18055873 | 7 | 14719 | oar3_OAR1_18041155 | oar3_OAR1_18055873 |
| 1 | 18349667 | 18389672 | 15 | 40006 | oar3_OAR1_18349667 | oar3_OAR1_18389672 |
| 1 | 19214192 | 19273693 | 16 | 59502 | s68238.1 | oar3_OAR1_19273693 |
| 1 | 19229668 | 19273693 | 13 | 44026 | oar3_OAR1_19229668 | oar3_OAR1_19273693 |
| 1 | 19240294 | 19285241 | 15 | 44948 | oar3_OAR1_19240294 | oar3_OAR1_19285241 |
| 1 | 19252556 | 19268772 | 8 | 16217 | oar3_OAR1_19252556 | oar3_OAR1_19268772 |
| 1 | 19252556 | 19273693 | 9 | 21138 | oar3_OAR1_19252556 | oar3_OAR1_19273693 |
| 1 | 20373749 | 20391284 | 4 | 17536 | oar3_OAR1_20373749 | oar3_OAR1_20391284 |
| 1 | 20763894 | 20803868 | 11 | 39975 | oar3_OAR1_20763894 | oar3_OAR1_20803868 |
| 1 | 20848290 | 20915457 | 16 | 67168 | oar3_OAR1_20848290 | oar3_OAR1_20915457 |
| 1 | 20860757 | 20915457 | 12 | 54701 | s31459.1 | oar3_OAR1_20915457 |
| 1 | 22223962 | 22263272 | 13 | 39311 | oar3_OAR1_22223962 | oar3_OAR1_22263272 |
| 1 | 22253882 | 22263272 | 5 | 9391 | oar3_OAR1_22253882 | oar3_OAR1_22263272 |
| 1 | 22257502 | 22263272 | 4 | 5771 | oar3_OAR1_22257502 | oar3_OAR1_22263272 |
| 1 | 22529742 | 22544815 | 7 | 15074 | s75089.1 | oar3_OAR1_22544815 |
| 1 | 22529742 | 22547501 | 8 | 17760 | s75089.1 | oar3_OAR1_22547501 |
| 1 | 22889133 | 22895020 | 4 | 5888 | oar3_OAR1_22889133 | OAR1_22985927.1 |
| 1 | 27085903 | 27120829 | 6 | 34927 | oar3_OAR1_27085903 | oar3_OAR1_27120829 |
| 1 | 27114387 | 27149809 | 7 | 35423 | oar3_OAR1_27114387 | oar3_OAR1_27149809 |
| 1 | 27333681 | 27369275 | 9 | 35595 | s02259.1 | oar3_OAR1_27369275 |
| 1 | 27544008 | 27606495 | 16 | 62488 | oar3_OAR1_27544008 | oar3_OAR1_27606495 |
| 1 | 27544008 | 27591289 | 12 | 47282 | oar3_OAR1_27544008 | oar3_OAR1_27591289 |
| 1 | 27544008 | 27591289 | 12 | 47282 | oar3_OAR1_27544008 | oar3_OAR1_27591289 |
| 1 | 27568168 | 27591289 | 7 | 23122 | oar3_OAR1_27568168 | oar3_OAR1_27591289 |
| 1 | 27775891 | 27954808 | 18 | 178918 | oar3_OAR1_27775891 | oar3_OAR1_27954808 |
| 1 | 27925957 | 27954808 | 11 | 28852 | oar3_OAR1_27925957 | oar3_OAR1_27954808 |
| 1 | 28947828 | 29013932 | 20 | 66105 | oar3_OAR1_28947828 | oar3_OAR1_29013932 |
| 1 | 28972525 | 29008912 | 11 | 36388 | oar3_OAR1_28972525 | oar3_OAR1_29008912 |
| 1 | 29118266 | 29148388 | 13 | 30123 | oar3_OAR1_29118266 | oar3_OAR1_29148388 |
| 1 | 57994785 | 58061776 | 13 | 66992 | oar3_OAR1_57994785 | oar3_OAR1_58061776 |
| 1 | 65666227 | 65687573 | 6 | 21347 | oar3_OAR1_65666227 | oar3_OAR1_65687573 |
| 1 | 65693790 | 65721810 | 4 | 28021 | oar3_OAR1_65693790 | oar3_OAR1_65721810 |
| 1 | 67503987 | 67536295 | 10 | 32309 | oar3_OAR1_67503987 | oar3_OAR1_67536295 |
| 1 | 81731065 | 81871731 | 24 | 140667 | oar3_OAR1_81731065 | oar3_OAR1_81871731 |
| 1 | 85752680 | 85753906 | 4 | 1227 | oar3_OAR1_85752680 | oar3_OAR1_85753906 |
| 1 | 85886747 | 85921445 | 14 | 34699 | oar3_OAR1_85886747 | oar3_OAR1_85921445 |
| 1 | 88490072 | 88543147 | 14 | 53076 | oar3_OAR1_88490072 | oar3_OAR1_88543147 |
| 1 | 89244808 | 89261022 | 6 | 16215 | oar3_OAR1_89244808 | oar3_OAR1_89261022 |
| 1 | 91573814 | 91599161 | 10 | 25348 | s67307.1 | oar3_OAR1_91599161 |
| 1 | 95998052 | 96011223 | 6 | 13172 | oar3_OAR1_95998052 | oar3_OAR1_96011223 |
| 1 | 1.01E+08 | 1.01E+08 | 17 | 38962 | oar3_OAR1_101315383 | oar3_OAR1_101354344 |
| 1 | 1.02E+08 | 1.02E+08 | 9 | 11793 | oar3_OAR1_102286053 | oar3_OAR1_102297845 |
| 1 | 1.03E+08 | 1.03E+08 | 7 | 28775 | oar3_OAR1_102758169 | oar3_OAR1_102786943 |
| 1 | 1.04E+08 | 1.04E+08 | 16 | 67118 | oar3_OAR1_103658300 | oar3_OAR1_103725417 |
| 1 | 1.04E+08 | 1.04E+08 | 21 | 79001 | oar3_OAR1_103744512 | oar3_OAR1_103823512 |
| 1 | 1.04E+08 | 1.04E+08 | 5 | 6345 | oar3_OAR1_103750215 | s03716.1 |
| 1 | 1.04E+08 | 1.04E+08 | 9 | 53909 | oar3_OAR1_103862621 | oar3_OAR1_103916529 |
| 1 | 1.05E+08 | 1.05E+08 | 16 | 46292 | oar3_OAR1_104514334 | oar3_OAR1_104560625 |
| 1 | 1.05E+08 | 1.05E+08 | 6 | 7828 | oar3_OAR1_104552798 | oar3_OAR1_104560625 |
| 1 | 1.05E+08 | 1.05E+08 | 6 | 7828 | oar3_OAR1_104552798 | oar3_OAR1_104560625 |
| 1 | 1.05E+08 | 1.05E+08 | 30 | 110234 | oar3_OAR1_104720393 | oar3_OAR1_104830626 |
| 1 | 1.05E+08 | 1.05E+08 | 4 | 8432 | oar3_OAR1_104822195 | oar3_OAR1_104830626 |
| 1 | 1.05E+08 | 1.05E+08 | 6 | 17924 | oar3_OAR1_104929178 | oar3_OAR1_104947101 |
| 1 | 1.05E+08 | 1.05E+08 | 6 | 17924 | oar3_OAR1_104929178 | oar3_OAR1_104947101 |
| 1 | 1.05E+08 | 1.05E+08 | 6 | 17924 | oar3_OAR1_104929178 | oar3_OAR1_104947101 |
| 1 | 1.05E+08 | 1.05E+08 | 4 | 3006 | oar3_OAR1_104932901 | oar3_OAR1_104935906 |
| 1 | 1.05E+08 | 1.05E+08 | 5 | 14201 | oar3_OAR1_104932901 | oar3_OAR1_104947101 |
| 1 | 1.05E+08 | 1.05E+08 | 17 | 25765 | oar3_OAR1_105186368 | oar3_OAR1_105212132 |
| 1 | 1.05E+08 | 1.05E+08 | 10 | 13907 | OAR1_112952978.1 | oar3_OAR1_105212132 |
| 1 | 1.05E+08 | 1.05E+08 | 10 | 22436 | oar3_OAR1_105269404 | s45548.1 |
| 1 | 1.05E+08 | 1.05E+08 | 3 | 2081 | oar3_OAR1_105269404 | oar3_OAR1_105271484 |
| 1 | 1.06E+08 | 1.06E+08 | 3 | 2649 | oar3_OAR1_105855065 | oar3_OAR1_105857713 |
| 1 | 1.09E+08 | 1.1E+08 | 14 | 53383 | oar3_OAR1_109447836 | oar3_OAR1_109501218 |
| 1 | 1.1E+08 | 1.1E+08 | 7 | 10727 | oar3_OAR1_109692980 | s35704.1 |
| 1 | 1.1E+08 | 1.1E+08 | 7 | 35041 | oar3_OAR1_110380227 | oar3_OAR1_110415267 |
| 1 | 1.1E+08 | 1.1E+08 | 10 | 51905 | oar3_OAR1_110380227 | oar3_OAR1_110432131 |
| 1 | 1.1E+08 | 1.1E+08 | 8 | 35825 | oar3_OAR1_110386844 | oar3_OAR1_110422668 |
| 1 | 1.11E+08 | 1.11E+08 | 10 | 27355 | oar3_OAR1_110637772 | oar3_OAR1_110665126 |
| 1 | 1.11E+08 | 1.11E+08 | 4 | 11782 | oar3_OAR1_110637772 | oar3_OAR1_110649553 |
| 1 | 1.11E+08 | 1.11E+08 | 9 | 27107 | oar3_OAR1_110646842 | oar3_OAR1_110673948 |
| 1 | 1.11E+08 | 1.11E+08 | 20 | 92014 | oar3_OAR1_110646842 | oar3_OAR1_110738855 |
| 1 | 1.17E+08 | 1.17E+08 | 3 | 1977 | oar3_OAR1_116632173 | oar3_OAR1_116634149 |
| 1 | 1.21E+08 | 1.21E+08 | 17 | 39331 | oar3_OAR1_121395112 | oar3_OAR1_121434442 |
| 1 | 1.25E+08 | 1.25E+08 | 12 | 17924 | oar3_OAR1_124967241 | oar3_OAR1_124985164 |
| 1 | 1.25E+08 | 1.25E+08 | 9 | 6981 | oar3_OAR1_124978184 | oar3_OAR1_124985164 |
| 1 | 1.25E+08 | 1.25E+08 | 9 | 6981 | oar3_OAR1_124978184 | oar3_OAR1_124985164 |
| 1 | 1.3E+08 | 1.3E+08 | 4 | 1180 | oar3_OAR1_129828346 | oar3_OAR1_129829525 |
| 1 | 1.3E+08 | 1.3E+08 | 4 | 1180 | oar3_OAR1_129828346 | oar3_OAR1_129829525 |
| 1 | 1.39E+08 | 1.39E+08 | 5 | 11340 | oar3_OAR1_138926787 | oar3_OAR1_138938126 |
| 1 | 1.42E+08 | 1.42E+08 | 4 | 3250 | oar3_OAR1_142435110 | oar3_OAR1_142438359 |
| 1 | 1.45E+08 | 1.45E+08 | 5 | 6989 | OAR1_156188829.1 | oar3_OAR1_144705979 |
| 1 | 1.47E+08 | 1.47E+08 | 8 | 27956 | oar3_OAR1_146729481 | oar3_OAR1_146757436 |
| 1 | 1.49E+08 | 1.5E+08 | 4 | 11452 | oar3_OAR1_149497936 | oar3_OAR1_149509387 |
| 1 | 1.5E+08 | 1.5E+08 | 3 | 4241 | oar3_OAR1_149537506 | oar3_OAR1_149541746 |
| 1 | 1.51E+08 | 1.51E+08 | 7 | 30729 | oar3_OAR1_150696884 | oar3_OAR1_150727612 |
| 1 | 1.57E+08 | 1.58E+08 | 15 | 68371 | oar3_OAR1_157444442 | oar3_OAR1_157512812 |
| 1 | 1.89E+08 | 1.89E+08 | 7 | 23542 | oar3_OAR1_188865426 | oar3_OAR1_188888967 |
| 1 | 1.89E+08 | 1.89E+08 | 6 | 6003 | oar3_OAR1_189415966 | oar3_OAR1_189421968 |
| 1 | 1.89E+08 | 1.89E+08 | 4 | 1608 | oar3_OAR1_189420361 | oar3_OAR1_189421968 |
| 1 | 1.89E+08 | 1.89E+08 | 4 | 1608 | oar3_OAR1_189420361 | oar3_OAR1_189421968 |
| 1 | 1.9E+08 | 1.9E+08 | 6 | 22272 | oar3_OAR1_189687683 | oar3_OAR1_189709954 |
| 1 | 1.91E+08 | 1.91E+08 | 11 | 28491 | oar3_OAR1_190587322 | oar3_OAR1_190615812 |
| 1 | 1.91E+08 | 1.91E+08 | 8 | 13887 | oar3_OAR1_191176468 | oar3_OAR1_191190354 |
| 1 | 2.01E+08 | 2.01E+08 | 39 | 186620 | oar3_OAR1_201018287 | oar3_OAR1_201204906 |
| 1 | 2.01E+08 | 2.01E+08 | 39 | 186620 | oar3_OAR1_201018287 | oar3_OAR1_201204906 |
| 1 | 2.01E+08 | 2.01E+08 | 17 | 74872 | OAR1_216941527.1 | oar3_OAR1_201204906 |
| 1 | 2.02E+08 | 2.02E+08 | 6 | 19664 | oar3_OAR1_201510811 | oar3_OAR1_201530474 |
| 1 | 2.19E+08 | 2.19E+08 | 4 | 7871 | oar3_OAR1_219114289 | oar3_OAR1_219122159 |
| 1 | 2.2E+08 | 2.2E+08 | 14 | 31253 | oar3_OAR1_220379509 | oar3_OAR1_220410761 |
| 1 | 2.26E+08 | 2.26E+08 | 5 | 2403 | oar3_OAR1_225840966 | oar3_OAR1_225843368 |
| 1 | 2.26E+08 | 2.26E+08 | 5 | 2403 | oar3_OAR1_225840966 | oar3_OAR1_225843368 |
| 1 | 2.39E+08 | 2.39E+08 | 6 | 26322 | s70663.1 | oar3_OAR1_239023141 |
| 1 | 2.39E+08 | 2.39E+08 | 7 | 21777 | oar3_OAR1_239345177 | oar3_OAR1_239366953 |
| 1 | 2.44E+08 | 2.44E+08 | 5 | 7611 | oar3_OAR1_243942526 | oar3_OAR1_243950136 |
| 1 | 2.44E+08 | 2.44E+08 | 5 | 7611 | oar3_OAR1_243942526 | oar3_OAR1_243950136 |
| 1 | 2.51E+08 | 2.51E+08 | 5 | 20059 | oar3_OAR1_251391746 | oar3_OAR1_251411804 |
| 1 | 2.52E+08 | 2.52E+08 | 13 | 39269 | oar3_OAR1_252261558 | oar3_OAR1_252300826 |
| 1 | 2.53E+08 | 2.53E+08 | 8 | 23486 | oar3_OAR1_253380501 | oar3_OAR1_253403986 |
| 1 | 2.54E+08 | 2.54E+08 | 17 | 38048 | oar3_OAR1_253519311 | oar3_OAR1_253557358 |
| 1 | 2.54E+08 | 2.54E+08 | 15 | 31775 | oar3_OAR1_253525584 | oar3_OAR1_253557358 |
| 1 | 2.58E+08 | 2.58E+08 | 3 | 4379 | oar3_OAR1_258383977 | s16506.1 |
| 1 | 2.6E+08 | 2.6E+08 | 20 | 59616 | oar3_OAR1_259687605 | oar3_OAR1_259747220 |
| 1 | 2.6E+08 | 2.6E+08 | 21 | 71667 | oar3_OAR1_259687605 | oar3_OAR1_259759271 |
| 1 | 2.6E+08 | 2.6E+08 | 7 | 38543 | oar3_OAR1_259815659 | oar3_OAR1_259854201 |
| 1 | 2.6E+08 | 2.6E+08 | 25 | 100895 | oar3_OAR1_260011540 | oar3_OAR1_260112434 |
| 1 | 2.6E+08 | 2.6E+08 | 25 | 92440 | oar3_OAR1_260078358 | oar3_OAR1_260170797 |
| 1 | 2.6E+08 | 2.6E+08 | 38 | 138358 | oar3_OAR1_260083531 | oar3_OAR1_260221888 |
| 1 | 2.6E+08 | 2.6E+08 | 4 | 4188 | oar3_OAR1_260108247 | oar3_OAR1_260112434 |
| 1 | 2.6E+08 | 2.6E+08 | 20 | 57864 | oar3_OAR1_260164025 | oar3_OAR1_260221888 |
| 1 | 2.6E+08 | 2.6E+08 | 17 | 44532 | oar3_OAR1_260446177 | oar3_OAR1_260490708 |
| 1 | 2.6E+08 | 2.6E+08 | 17 | 44532 | oar3_OAR1_260446177 | oar3_OAR1_260490708 |
| 1 | 2.61E+08 | 2.61E+08 | 4 | 5605 | oar3_OAR1_260684114 | oar3_OAR1_260689718 |
| 1 | 2.61E+08 | 2.61E+08 | 4 | 2689 | oar3_OAR1_261162335 | s44037.1 |
| 1 | 2.61E+08 | 2.61E+08 | 5 | 6913 | oar3_OAR1_261162335 | oar3_OAR1_261169247 |
| 1 | 2.61E+08 | 2.61E+08 | 5 | 6913 | oar3_OAR1_261162335 | oar3_OAR1_261169247 |
| 1 | 2.61E+08 | 2.61E+08 | 4 | 2689 | oar3_OAR1_261162335 | s44037.1 |
| 1 | 2.61E+08 | 2.61E+08 | 32 | 130434 | oar3_OAR1_261220816 | oar3_OAR1_261351249 |
| 1 | 2.61E+08 | 2.61E+08 | 19 | 68770 | oar3_OAR1_261238938 | oar3_OAR1_261307707 |
| 1 | 2.61E+08 | 2.61E+08 | 17 | 65968 | oar3_OAR1_261238938 | oar3_OAR1_261304905 |
| 1 | 2.61E+08 | 2.61E+08 | 4 | 1046 | oar3_OAR1_261303860 | oar3_OAR1_261304905 |
| 1 | 2.61E+08 | 2.62E+08 | 49 | 226494 | oar3_OAR1_261475082 | s24480.1 |
| 1 | 2.62E+08 | 2.62E+08 | 4 | 8063 | oar3_OAR1_261595573 | oar3_OAR1_261603635 |
| 1 | 2.62E+08 | 2.62E+08 | 19 | 105677 | oar3_OAR1_261863368 | oar3_OAR1_261969044 |
| 1 | 2.62E+08 | 2.62E+08 | 87 | 360206 | oar3_OAR1_261920163 | DU373830_532.1 |
| 1 | 2.62E+08 | 2.62E+08 | 18 | 49799 | oar3_OAR1_261942398 | oar3_OAR1_261992196 |
| 1 | 2.62E+08 | 2.62E+08 | 14 | 43994 | oar3_OAR1_261942398 | oar3_OAR1_261986391 |
| 1 | 2.62E+08 | 2.63E+08 | 74 | 326605 | oar3_OAR1_262197904 | oar3_OAR1_262524508 |
| 1 | 2.62E+08 | 2.62E+08 | 18 | 60528 | oar3_OAR1_262213109 | oar3_OAR1_262273636 |
| 1 | 2.62E+08 | 2.62E+08 | 11 | 27469 | oar3_OAR1_262219715 | oar3_OAR1_262247183 |
| 1 | 2.62E+08 | 2.62E+08 | 11 | 27469 | oar3_OAR1_262219715 | oar3_OAR1_262247183 |
| 1 | 2.62E+08 | 2.62E+08 | 17 | 53922 | oar3_OAR1_262219715 | oar3_OAR1_262273636 |
| 1 | 2.62E+08 | 2.62E+08 | 11 | 27469 | oar3_OAR1_262219715 | oar3_OAR1_262247183 |
| 1 | 2.62E+08 | 2.62E+08 | 18 | 66907 | oar3_OAR1_262386675 | oar3_OAR1_262453581 |
| 1 | 2.62E+08 | 2.63E+08 | 36 | 135590 | oar3_OAR1_262386675 | oar3_OAR1_262522264 |
| 1 | 2.62E+08 | 2.63E+08 | 29 | 117363 | oar3_OAR1_262395533 | s49200.1 |
| 1 | 2.62E+08 | 2.63E+08 | 25 | 97933 | oar3_OAR1_262407194 | oar3_OAR1_262505126 |
| 1 | 2.62E+08 | 2.63E+08 | 24 | 96174 | s71621.1 | oar3_OAR1_262505126 |
| 1 | 2.62E+08 | 2.63E+08 | 30 | 108768 | oar3_OAR1_262415741 | oar3_OAR1_262524508 |
| 1 | 2.62E+08 | 2.63E+08 | 20 | 79919 | oar3_OAR1_262425208 | oar3_OAR1_262505126 |
| 1 | 2.62E+08 | 2.63E+08 | 24 | 79196 | oar3_OAR1_262448570 | oar3_OAR1_262527765 |
| 1 | 2.63E+08 | 2.63E+08 | 14 | 34322 | oar3_OAR1_262537361 | oar3_OAR1_262571682 |
| 1 | 2.63E+08 | 2.63E+08 | 14 | 48546 | oar3_OAR1_262669374 | s18506.1 |
| 1 | 2.63E+08 | 2.63E+08 | 13 | 65499 | oar3_OAR1_262937799 | oar3_OAR1_263003297 |
| 1 | 2.63E+08 | 2.63E+08 | 9 | 40458 | oar3_OAR1_262937799 | oar3_OAR1_262978256 |
| 1 | 2.63E+08 | 2.63E+08 | 8 | 40106 | oar3_OAR1_262956968 | oar3_OAR1_262997073 |
| 1 | 2.63E+08 | 2.63E+08 | 3 | 854 | oar3_OAR1_262975317 | oar3_OAR1_262976170 |
| 1 | 2.63E+08 | 2.63E+08 | 3 | 854 | oar3_OAR1_262975317 | oar3_OAR1_262976170 |
| 1 | 2.63E+08 | 2.63E+08 | 53 | 243023 | oar3_OAR1_263047403 | oar3_OAR1_263290425 |
| 1 | 2.63E+08 | 2.63E+08 | 37 | 170097 | oar3_OAR1_263107746 | oar3_OAR1_263277842 |
| 1 | 2.63E+08 | 2.63E+08 | 35 | 158388 | oar3_OAR1_263107746 | oar3_OAR1_263266133 |
| 1 | 2.63E+08 | 2.63E+08 | 18 | 73906 | s01225.1 | oar3_OAR1_263227515 |
| 1 | 2.63E+08 | 2.63E+08 | 55 | 265172 | oar3_OAR1_263156665 | oar3_OAR1_263421836 |
| 1 | 2.63E+08 | 2.63E+08 | 13 | 33468 | oar3_OAR1_263199882 | oar3_OAR1_263233349 |
| 1 | 2.63E+08 | 2.63E+08 | 17 | 56171 | oar3_OAR1_263201199 | oar3_OAR1_263257369 |
| 1 | 2.63E+08 | 2.63E+08 | 23 | 89227 | oar3_OAR1_263201199 | oar3_OAR1_263290425 |
| 1 | 2.63E+08 | 2.63E+08 | 10 | 21929 | oar3_OAR1_263205587 | oar3_OAR1_263227515 |
| 1 | 2.63E+08 | 2.63E+08 | 6 | 15566 | oar3_OAR1_263205587 | oar3_OAR1_263221152 |
| 1 | 2.63E+08 | 2.63E+08 | 17 | 60547 | oar3_OAR1_263205587 | oar3_OAR1_263266133 |
| 1 | 2.63E+08 | 2.63E+08 | 31 | 148757 | oar3_OAR1_263331498 | oar3_OAR1_263480254 |
| 1 | 2.63E+08 | 2.63E+08 | 27 | 115316 | oar3_OAR1_263331498 | oar3_OAR1_263446813 |
| 1 | 2.63E+08 | 2.63E+08 | 27 | 115316 | oar3_OAR1_263331498 | oar3_OAR1_263446813 |
| 1 | 2.63E+08 | 2.63E+08 | 22 | 76607 | oar3_OAR1_263331498 | oar3_OAR1_263408104 |
| 1 | 2.63E+08 | 2.63E+08 | 18 | 68651 | oar3_OAR1_263339454 | oar3_OAR1_263408104 |
| 1 | 2.63E+08 | 2.63E+08 | 17 | 66471 | oar3_OAR1_263355366 | oar3_OAR1_263421836 |
| 1 | 2.63E+08 | 2.63E+08 | 17 | 78868 | oar3_OAR1_263367946 | oar3_OAR1_263446813 |
| 1 | 2.63E+08 | 2.63E+08 | 13 | 53891 | oar3_OAR1_263367946 | oar3_OAR1_263421836 |
| 1 | 2.63E+08 | 2.63E+08 | 10 | 50743 | oar3_OAR1_263396071 | oar3_OAR1_263446813 |
| 1 | 2.64E+08 | 2.64E+08 | 3 | 8946 | oar3_OAR1_263667096 | s64006.1 |
| 1 | 2.64E+08 | 2.64E+08 | 6 | 8705 | oar3_OAR1_263706554 | oar3_OAR1_263715258 |
| 1 | 2.64E+08 | 2.64E+08 | 21 | 61185 | oar3_OAR1_263770539 | OAR1_285314256.1 |
| 1 | 2.64E+08 | 2.64E+08 | 8 | 8330 | oar3_OAR1_263823394 | OAR1_285314256.1 |
| 1 | 2.64E+08 | 2.64E+08 | 8 | 8330 | oar3_OAR1_263823394 | OAR1_285314256.1 |
| 1 | 2.64E+08 | 2.64E+08 | 33 | 161283 | oar3_OAR1_263869952 | oar3_OAR1_264031234 |
| 1 | 2.64E+08 | 2.64E+08 | 36 | 185867 | oar3_OAR1_263869952 | oar3_OAR1_264055818 |
| 1 | 2.64E+08 | 2.64E+08 | 27 | 106251 | oar3_OAR1_263924984 | oar3_OAR1_264031234 |
| 1 | 2.64E+08 | 2.64E+08 | 21 | 65819 | oar3_OAR1_263965416 | oar3_OAR1_264031234 |
| 1 | 2.64E+08 | 2.64E+08 | 14 | 36422 | oar3_OAR1_263965416 | oar3_OAR1_264001837 |
| 1 | 2.64E+08 | 2.64E+08 | 14 | 36422 | oar3_OAR1_263965416 | oar3_OAR1_264001837 |
| 1 | 2.64E+08 | 2.64E+08 | 9 | 14430 | s53402.1 | oar3_OAR1_264001837 |
| 1 | 2.64E+08 | 2.64E+08 | 42 | 130957 | oar3_OAR1_264097034 | oar3_OAR1_264227990 |
| 1 | 2.64E+08 | 2.64E+08 | 5 | 21145 | oar3_OAR1_264097034 | oar3_OAR1_264118178 |
| 1 | 2.64E+08 | 2.64E+08 | 40 | 103966 | oar3_OAR1_264144511 | oar3_OAR1_264248476 |
| 1 | 2.64E+08 | 2.64E+08 | 13 | 32038 | oar3_OAR1_264216439 | oar3_OAR1_264248476 |
| 1 | 2.67E+08 | 2.67E+08 | 3 | 4323 | oar3_OAR1_266929402 | oar3_OAR1_266933724 |
| 1 | 2.67E+08 | 2.67E+08 | 5 | 19544 | oar3_OAR1_267057834 | oar3_OAR1_267077377 |
| 1 | 2.68E+08 | 2.68E+08 | 6 | 9894 | OAR1_290348891.1 | oar3_OAR1_268004276 |
| 1 | 2.69E+08 | 2.69E+08 | 19 | 69543 | oar3_OAR1_268709127 | oar3_OAR1_268778669 |
| 1 | 2.7E+08 | 2.7E+08 | 19 | 68986 | oar3_OAR1_270261569 | oar3_OAR1_270330554 |
| 1 | 2.7E+08 | 2.71E+08 | 7 | 30473 | oar3_OAR1_270483710 | oar3_OAR1_270514182 |
| 1 | 2.7E+08 | 2.71E+08 | 17 | 72949 | oar3_OAR1_270484744 | oar3_OAR1_270557692 |
| 1 | 2.7E+08 | 2.71E+08 | 4 | 19922 | oar3_OAR1_270494261 | oar3_OAR1_270514182 |
| 1 | 2.71E+08 | 2.71E+08 | 12 | 31139 | oar3_OAR1_271357001 | oar3_OAR1_271388139 |
| 1 | 2.72E+08 | 2.72E+08 | 28 | 155639 | oar3_OAR1_272118686 | oar3_OAR1_272274324 |
| 1 | 2.72E+08 | 2.72E+08 | 8 | 37846 | oar3_OAR1_272141098 | s29335.1 |
| 1 | 2.72E+08 | 2.72E+08 | 10 | 45332 | oar3_OAR1_272141098 | oar3_OAR1_272186429 |
| 1 | 2.72E+08 | 2.72E+08 | 8 | 37846 | oar3_OAR1_272141098 | s29335.1 |
| 1 | 2.72E+08 | 2.72E+08 | 10 | 45332 | oar3_OAR1_272141098 | oar3_OAR1_272186429 |
| 1 | 2.72E+08 | 2.72E+08 | 25 | 139523 | oar3_OAR1_272141098 | oar3_OAR1_272280620 |
| 1 | 2.72E+08 | 2.72E+08 | 5 | 21046 | oar3_OAR1_272141098 | oar3_OAR1_272162143 |
| 1 | 2.72E+08 | 2.72E+08 | 8 | 32518 | oar3_OAR1_272153912 | oar3_OAR1_272186429 |
| 1 | 2.72E+08 | 2.72E+08 | 8 | 32518 | oar3_OAR1_272153912 | oar3_OAR1_272186429 |
| 1 | 2.72E+08 | 2.72E+08 | 8 | 32518 | oar3_OAR1_272153912 | oar3_OAR1_272186429 |
| 1 | 2.72E+08 | 2.72E+08 | 8 | 32518 | oar3_OAR1_272153912 | oar3_OAR1_272186429 |
| 1 | 2.72E+08 | 2.72E+08 | 3 | 5710 | oar3_OAR1_272173234 | s29335.1 |
| 1 | 2.74E+08 | 2.74E+08 | 15 | 65754 | oar3_OAR1_273556585 | oar3_OAR1_273622338 |
| 1 | 2.75E+08 | 2.75E+08 | 8 | 14629 | oar3_OAR1_275297168 | oar3_OAR1_275311796 |
| 1 | 2.75E+08 | 2.75E+08 | 33 | 135080 | oar3_OAR1_275307529 | oar3_OAR1_275442608 |
| 1 | 2.75E+08 | 2.75E+08 | 23 | 95018 | oar3_OAR1_275347591 | oar3_OAR1_275442608 |
| 1 | 2.75E+08 | 2.75E+08 | 16 | 67788 | oar3_OAR1_275357346 | oar3_OAR1_275425133 |
| 2 | 157959 | 168924 | 5 | 10966 | oar3_OAR2_157959 | oar3_OAR2_168924 |
| 2 | 157959 | 301525 | 34 | 143567 | oar3_OAR2_157959 | oar3_OAR2_301525 |
| 2 | 157959 | 281346 | 30 | 123388 | oar3_OAR2_157959 | oar3_OAR2_281346 |
| 2 | 157959 | 177700 | 6 | 19742 | oar3_OAR2_157959 | oar3_OAR2_177700 |
| 2 | 221346 | 281346 | 21 | 60001 | oar3_OAR2_221346 | oar3_OAR2_281346 |
| 2 | 238610 | 240619 | 4 | 2010 | oar3_OAR2_238610 | oar3_OAR2_240619 |
| 2 | 238610 | 243903 | 5 | 5294 | oar3_OAR2_238610 | oar3_OAR2_243903 |
| 2 | 238610 | 240619 | 4 | 2010 | oar3_OAR2_238610 | oar3_OAR2_240619 |
| 2 | 238610 | 243903 | 5 | 5294 | oar3_OAR2_238610 | oar3_OAR2_243903 |
| 2 | 301525 | 592224 | 69 | 290700 | oar3_OAR2_301525 | oar3_OAR2_592224 |
| 2 | 454452 | 666318 | 56 | 211867 | oar3_OAR2_454452 | oar3_OAR2_666318 |
| 2 | 468502 | 750769 | 72 | 282268 | oar3_OAR2_468502 | oar3_OAR2_750769 |
| 2 | 468502 | 666318 | 51 | 197817 | oar3_OAR2_468502 | oar3_OAR2_666318 |
| 2 | 538815 | 545308 | 6 | 6494 | oar3_OAR2_538815 | oar3_OAR2_545308 |
| 2 | 541813 | 666318 | 36 | 124506 | oar3_OAR2_541813 | oar3_OAR2_666318 |
| 2 | 565916 | 610504 | 11 | 44589 | oar3_OAR2_565916 | oar3_OAR2_610504 |
| 2 | 610504 | 746203 | 36 | 135700 | oar3_OAR2_610504 | oar3_OAR2_746203 |
| 2 | 638312 | 656567 | 6 | 18256 | oar3_OAR2_638312 | oar3_OAR2_656567 |
| 2 | 679324 | 891246 | 49 | 211923 | s33481.1 | oar3_OAR2_891246 |
| 2 | 685645 | 770793 | 19 | 85149 | oar3_OAR2_685645 | oar3_OAR2_770793 |
| 2 | 711076 | 763743 | 12 | 52668 | oar3_OAR2_711076 | oar3_OAR2_763743 |
| 2 | 711076 | 750769 | 10 | 39694 | oar3_OAR2_711076 | oar3_OAR2_750769 |
| 2 | 711076 | 814115 | 23 | 103040 | oar3_OAR2_711076 | oar3_OAR2_814115 |
| 2 | 715526 | 746203 | 6 | 30678 | oar3_OAR2_715526 | oar3_OAR2_746203 |
| 2 | 737473 | 750769 | 4 | 13297 | oar3_OAR2_737473 | oar3_OAR2_750769 |
| 2 | 811330 | 814115 | 4 | 2786 | oar3_OAR2_811330 | oar3_OAR2_814115 |
| 2 | 811330 | 822884 | 7 | 11555 | oar3_OAR2_811330 | oar3_OAR2_822884 |
| 2 | 811330 | 865505 | 15 | 54176 | oar3_OAR2_811330 | oar3_OAR2_865505 |
| 2 | 811330 | 818610 | 5 | 7281 | oar3_OAR2_811330 | oar3_OAR2_818610 |
| 2 | 826798 | 915172 | 21 | 88375 | oar3_OAR2_826798 | oar3_OAR2_915172 |
| 2 | 826798 | 843923 | 5 | 17126 | oar3_OAR2_826798 | oar3_OAR2_843923 |
| 2 | 883204 | 915172 | 11 | 31969 | oar3_OAR2_883204 | oar3_OAR2_915172 |
| 2 | 883204 | 914854 | 10 | 31651 | oar3_OAR2_883204 | oar3_OAR2_914854 |
| 2 | 955809 | 1004540 | 13 | 48732 | oar3_OAR2_955809 | oar3_OAR2_1004540 |
| 2 | 1055883 | 1144845 | 19 | 88963 | oar3_OAR2_1055883 | oar3_OAR2_1144845 |
| 2 | 1112556 | 1150425 | 10 | 37870 | oar3_OAR2_1112556 | oar3_OAR2_1150425 |
| 2 | 1178140 | 1265940 | 28 | 87801 | oar3_OAR2_1178140 | oar3_OAR2_1265940 |
| 2 | 1206287 | 1239432 | 12 | 33146 | oar3_OAR2_1206287 | oar3_OAR2_1239432 |
| 2 | 1213258 | 1235011 | 7 | 21754 | oar3_OAR2_1213258 | oar3_OAR2_1235011 |
| 2 | 1378233 | 1475015 | 22 | 96783 | oar3_OAR2_1378233 | oar3_OAR2_1475015 |
| 2 | 1408222 | 1426911 | 7 | 18690 | oar3_OAR2_1408222 | s34805.1 |
| 2 | 1408222 | 1426911 | 7 | 18690 | oar3_OAR2_1408222 | s34805.1 |
| 2 | 1414233 | 1426911 | 6 | 12679 | oar3_OAR2_1414233 | s34805.1 |
| 2 | 1419284 | 1426911 | 4 | 7628 | oar3_OAR2_1419284 | s34805.1 |
| 2 | 1523260 | 1550658 | 11 | 27399 | oar3_OAR2_1523260 | oar3_OAR2_1550658 |
| 2 | 1690074 | 1717596 | 7 | 27523 | oar3_OAR2_1690074 | s34808.1 |
| 2 | 1690074 | 1717596 | 7 | 27523 | oar3_OAR2_1690074 | s34808.1 |
| 2 | 1732781 | 1792672 | 14 | 59892 | oar3_OAR2_1732781 | oar3_OAR2_1792672 |
| 2 | 1803652 | 1818104 | 7 | 14453 | oar3_OAR2_1803652 | oar3_OAR2_1818104 |
| 2 | 1811707 | 1844625 | 8 | 32919 | oar3_OAR2_1811707 | oar3_OAR2_1844625 |
| 2 | 1811707 | 1844625 | 8 | 32919 | oar3_OAR2_1811707 | oar3_OAR2_1844625 |
| 2 | 2162180 | 2216402 | 16 | 54223 | oar3_OAR2_2162180 | oar3_OAR2_2216402 |
| 2 | 2216402 | 2225592 | 4 | 9191 | oar3_OAR2_2216402 | s60414.1 |
| 2 | 3056356 | 3092819 | 10 | 36464 | oar3_OAR2_3056356 | oar3_OAR2_3092819 |
| 2 | 9324583 | 9348585 | 15 | 24003 | oar3_OAR2_9324583 | oar3_OAR2_9348585 |
| 2 | 9330164 | 9348585 | 14 | 18422 | oar3_OAR2_9330164 | oar3_OAR2_9348585 |
| 2 | 9330164 | 9348585 | 14 | 18422 | oar3_OAR2_9330164 | oar3_OAR2_9348585 |
| 2 | 9333424 | 9494709 | 45 | 161286 | oar3_OAR2_9333424 | oar3_OAR2_9494709 |
| 2 | 9391363 | 9434878 | 14 | 43516 | oar3_OAR2_9391363 | oar3_OAR2_9434878 |
| 2 | 9563949 | 9610640 | 21 | 46692 | oar3_OAR2_9563949 | oar3_OAR2_9610640 |
| 2 | 9563949 | 9578996 | 6 | 15048 | oar3_OAR2_9563949 | oar3_OAR2_9578996 |
| 2 | 9563949 | 9578996 | 6 | 15048 | oar3_OAR2_9563949 | oar3_OAR2_9578996 |
| 2 | 10257348 | 10279988 | 11 | 22641 | oar3_OAR2_10257348 | oar3_OAR2_10279988 |
| 2 | 10275109 | 10325810 | 18 | 50702 | oar3_OAR2_10275109 | oar3_OAR2_10325810 |
| 2 | 10275109 | 10298238 | 12 | 23130 | oar3_OAR2_10275109 | oar3_OAR2_10298238 |
| 2 | 10279112 | 10279988 | 6 | 877 | oar3_OAR2_10279112 | oar3_OAR2_10279988 |
| 2 | 10279112 | 10279988 | 6 | 877 | oar3_OAR2_10279112 | oar3_OAR2_10279988 |
| 2 | 10279112 | 10279988 | 6 | 877 | oar3_OAR2_10279112 | oar3_OAR2_10279988 |
| 2 | 10279112 | 10279988 | 6 | 877 | oar3_OAR2_10279112 | oar3_OAR2_10279988 |
| 2 | 10279112 | 10279988 | 6 | 877 | oar3_OAR2_10279112 | oar3_OAR2_10279988 |
| 2 | 10553921 | 10571760 | 5 | 17840 | oar3_OAR2_10553921 | oar3_OAR2_10571760 |
| 2 | 10553921 | 10571760 | 5 | 17840 | oar3_OAR2_10553921 | oar3_OAR2_10571760 |
| 2 | 13870959 | 13898175 | 10 | 27217 | oar3_OAR2_13870959 | oar3_OAR2_13898175 |
| 2 | 13883490 | 13898175 | 5 | 14686 | oar3_OAR2_13883490 | oar3_OAR2_13898175 |
| 2 | 23044762 | 23045955 | 3 | 1194 | oar3_OAR2_23044762 | oar3_OAR2_23045955 |
| 2 | 23044762 | 23045955 | 3 | 1194 | oar3_OAR2_23044762 | oar3_OAR2_23045955 |
| 2 | 23044762 | 23045955 | 3 | 1194 | oar3_OAR2_23044762 | oar3_OAR2_23045955 |
| 2 | 23044762 | 23045955 | 3 | 1194 | oar3_OAR2_23044762 | oar3_OAR2_23045955 |
| 2 | 23044762 | 23045955 | 3 | 1194 | oar3_OAR2_23044762 | oar3_OAR2_23045955 |
| 2 | 23044762 | 23045955 | 3 | 1194 | oar3_OAR2_23044762 | oar3_OAR2_23045955 |
| 2 | 23044762 | 23045955 | 3 | 1194 | oar3_OAR2_23044762 | oar3_OAR2_23045955 |
| 2 | 24308219 | 24352116 | 9 | 43898 | oar3_OAR2_24308219 | oar3_OAR2_24352116 |
| 2 | 24308219 | 24352116 | 9 | 43898 | oar3_OAR2_24308219 | oar3_OAR2_24352116 |
| 2 | 24308219 | 24428924 | 25 | 120706 | oar3_OAR2_24308219 | oar3_OAR2_24428924 |
| 2 | 24308219 | 24352116 | 9 | 43898 | oar3_OAR2_24308219 | oar3_OAR2_24352116 |
| 2 | 24406825 | 24435208 | 8 | 28384 | oar3_OAR2_24406825 | oar3_OAR2_24435208 |
| 2 | 27496648 | 27507513 | 4 | 10866 | oar3_OAR2_27496648 | oar3_OAR2_27507513 |
| 2 | 27503168 | 27549630 | 15 | 46463 | oar3_OAR2_27503168 | oar3_OAR2_27549630 |
| 2 | 27532789 | 27549630 | 9 | 16842 | oar3_OAR2_27532789 | oar3_OAR2_27549630 |
| 2 | 28083528 | 28152449 | 16 | 68922 | oar3_OAR2_28083528 | oar3_OAR2_28152449 |
| 2 | 28083528 | 28098208 | 9 | 14681 | oar3_OAR2_28083528 | oar3_OAR2_28098208 |
| 2 | 28083528 | 28098208 | 9 | 14681 | oar3_OAR2_28083528 | oar3_OAR2_28098208 |
| 2 | 28162293 | 28215589 | 15 | 53297 | oar3_OAR2_28162293 | oar3_OAR2_28215589 |
| 2 | 28162293 | 28236574 | 18 | 74282 | oar3_OAR2_28162293 | oar3_OAR2_28236574 |
| 2 | 28185023 | 28215589 | 8 | 30567 | oar3_OAR2_28185023 | oar3_OAR2_28215589 |
| 2 | 28185023 | 28409373 | 53 | 224351 | oar3_OAR2_28185023 | oar3_OAR2_28409373 |
| 2 | 28195591 | 28252533 | 15 | 56943 | oar3_OAR2_28195591 | s56017.1 |
| 2 | 28200541 | 28330201 | 29 | 129661 | oar3_OAR2_28200541 | oar3_OAR2_28330201 |
| 2 | 28295553 | 28388355 | 28 | 92803 | oar3_OAR2_28295553 | oar3_OAR2_28388355 |
| 2 | 42276176 | 42321990 | 13 | 45815 | s32184.1 | oar3_OAR2_42321990 |
| 2 | 42281710 | 42321990 | 12 | 40281 | oar3_OAR2_42281710 | oar3_OAR2_42321990 |
| 2 | 42744530 | 42830566 | 25 | 86037 | oar3_OAR2_42744530 | oar3_OAR2_42830566 |
| 2 | 42760779 | 42830566 | 21 | 69788 | oar3_OAR2_42760779 | oar3_OAR2_42830566 |
| 2 | 42781505 | 42822857 | 10 | 41353 | oar3_OAR2_42781505 | oar3_OAR2_42822857 |
| 2 | 42783841 | 42830566 | 13 | 46726 | oar3_OAR2_42783841 | oar3_OAR2_42830566 |
| 2 | 42783841 | 42830566 | 13 | 46726 | oar3_OAR2_42783841 | oar3_OAR2_42830566 |
| 2 | 42786287 | 42830566 | 12 | 44280 | s48762.1 | oar3_OAR2_42830566 |
| 2 | 42792215 | 42843174 | 12 | 50960 | oar3_OAR2_42792215 | oar3_OAR2_42843174 |
| 2 | 42792215 | 42830566 | 11 | 38352 | oar3_OAR2_42792215 | oar3_OAR2_42830566 |
| 2 | 42822857 | 42830566 | 5 | 7710 | oar3_OAR2_42822857 | oar3_OAR2_42830566 |
| 2 | 43147217 | 43261756 | 25 | 114540 | oar3_OAR2_43147217 | oar3_OAR2_43261756 |
| 2 | 43162230 | 43204586 | 7 | 42357 | oar3_OAR2_43162230 | oar3_OAR2_43204586 |
| 2 | 43162230 | 43261756 | 20 | 99527 | oar3_OAR2_43162230 | oar3_OAR2_43261756 |
| 2 | 43204586 | 43261756 | 14 | 57171 | oar3_OAR2_43204586 | oar3_OAR2_43261756 |
| 2 | 43214757 | 43261756 | 13 | 47000 | oar3_OAR2_43214757 | oar3_OAR2_43261756 |
| 2 | 43221935 | 43229856 | 5 | 7922 | oar3_OAR2_43221935 | oar3_OAR2_43229856 |
| 2 | 43302318 | 43307477 | 4 | 5160 | oar3_OAR2_43302318 | oar3_OAR2_43307477 |
| 2 | 43302318 | 43307477 | 4 | 5160 | oar3_OAR2_43302318 | oar3_OAR2_43307477 |
| 2 | 45276597 | 45326390 | 15 | 49794 | oar3_OAR2_45276597 | oar3_OAR2_45326390 |
| 2 | 45297048 | 45323700 | 9 | 26653 | oar3_OAR2_45297048 | oar3_OAR2_45323700 |
| 2 | 45297048 | 45329654 | 11 | 32607 | oar3_OAR2_45297048 | oar3_OAR2_45329654 |
| 2 | 45297048 | 45326390 | 10 | 29343 | oar3_OAR2_45297048 | oar3_OAR2_45326390 |
| 2 | 45297048 | 45303769 | 4 | 6722 | oar3_OAR2_45297048 | oar3_OAR2_45303769 |
| 2 | 45297048 | 45326390 | 10 | 29343 | oar3_OAR2_45297048 | oar3_OAR2_45326390 |
| 2 | 51510244 | 51551346 | 13 | 41103 | oar3_OAR2_51510244 | oar3_OAR2_51551346 |
| 2 | 51510244 | 51551346 | 13 | 41103 | oar3_OAR2_51510244 | oar3_OAR2_51551346 |
| 2 | 51517016 | 51536570 | 7 | 19555 | oar3_OAR2_51517016 | oar3_OAR2_51536570 |
| 2 | 52518107 | 52606254 | 25 | 88148 | oar3_OAR2_52518107 | oar3_OAR2_52606254 |
| 2 | 52601096 | 52608260 | 10 | 7165 | oar3_OAR2_52601096 | oar3_OAR2_52608260 |
| 2 | 52601096 | 52617622 | 11 | 16527 | oar3_OAR2_52601096 | oar3_OAR2_52617622 |
| 2 | 52602161 | 52608017 | 6 | 5857 | oar3_OAR2_52602161 | oar3_OAR2_52608017 |
| 2 | 59564867 | 59590037 | 21 | 25171 | oar3_OAR2_59564867 | oar3_OAR2_59590037 |
| 2 | 59571841 | 59588322 | 6 | 16482 | oar3_OAR2_59571841 | oar3_OAR2_59588322 |
| 2 | 59571841 | 59590037 | 7 | 18197 | oar3_OAR2_59571841 | oar3_OAR2_59590037 |
| 2 | 59586282 | 59588322 | 3 | 2041 | oar3_OAR2_59586282 | oar3_OAR2_59588322 |
| 2 | 74539093 | 74595750 | 10 | 56658 | oar3_OAR2_74539093 | oar3_OAR2_74595750 |
| 2 | 98185354 | 98185738 | 3 | 385 | oar3_OAR2_98185354 | oar3_OAR2_98185738 |
| 2 | 98185354 | 98185738 | 3 | 385 | oar3_OAR2_98185354 | oar3_OAR2_98185738 |
| 2 | 1.02E+08 | 1.02E+08 | 13 | 57022 | oar3_OAR2_101637138 | oar3_OAR2_101694159 |
| 2 | 1.03E+08 | 1.03E+08 | 19 | 22546 | oar3_OAR2_103354042 | oar3_OAR2_103376587 |
| 2 | 1.03E+08 | 1.03E+08 | 19 | 22546 | oar3_OAR2_103354042 | oar3_OAR2_103376587 |
| 2 | 1.03E+08 | 1.03E+08 | 14 | 13403 | oar3_OAR2_103359173 | oar3_OAR2_103372575 |
| 2 | 1.03E+08 | 1.03E+08 | 17 | 10148 | oar3_OAR2_103366440 | oar3_OAR2_103376587 |
| 2 | 1.03E+08 | 1.03E+08 | 12 | 3427 | oar3_OAR2_103366440 | oar3_OAR2_103369866 |
| 2 | 1.03E+08 | 1.03E+08 | 10 | 2522 | oar3_OAR2_103367345 | oar3_OAR2_103369866 |
| 2 | 1.04E+08 | 1.04E+08 | 5 | 12777 | oar3_OAR2_104462786 | oar3_OAR2_104475562 |
| 2 | 1.04E+08 | 1.04E+08 | 5 | 21782 | oar3_OAR2_104475518 | oar3_OAR2_104497299 |
| 2 | 1.11E+08 | 1.11E+08 | 24 | 110962 | oar3_OAR2_110685585 | oar3_OAR2_110796546 |
| 2 | 1.11E+08 | 1.11E+08 | 13 | 57468 | oar3_OAR2_110739079 | oar3_OAR2_110796546 |
| 2 | 1.11E+08 | 1.11E+08 | 5 | 27318 | oar3_OAR2_110769229 | oar3_OAR2_110796546 |
| 2 | 1.11E+08 | 1.11E+08 | 12 | 42269 | oar3_OAR2_110844205 | oar3_OAR2_110886473 |
| 2 | 1.11E+08 | 1.11E+08 | 37 | 178620 | oar3_OAR2_110869203 | oar3_OAR2_111047822 |
| 2 | 1.13E+08 | 1.13E+08 | 4 | 2724 | oar3_OAR2_112867780 | oar3_OAR2_112870503 |
| 2 | 1.13E+08 | 1.13E+08 | 14 | 118235 | oar3_OAR2_113265745 | oar3_OAR2_113383979 |
| 2 | 1.14E+08 | 1.14E+08 | 17 | 117188 | oar3_OAR2_114120134 | oar3_OAR2_114237321 |
| 2 | 1.16E+08 | 1.16E+08 | 40 | 204321 | oar3_OAR2_115903807 | OAR2_124098623.1 |
| 2 | 1.16E+08 | 1.16E+08 | 39 | 202434 | oar3_OAR2_115903807 | oar3_OAR2_116106240 |
| 2 | 1.16E+08 | 1.16E+08 | 6 | 10852 | oar3_OAR2_116065498 | oar3_OAR2_116076349 |
| 2 | 1.16E+08 | 1.16E+08 | 8 | 31483 | oar3_OAR2_116065498 | s51605.1 |
| 2 | 1.16E+08 | 1.16E+08 | 8 | 31483 | oar3_OAR2_116065498 | s51605.1 |
| 2 | 1.16E+08 | 1.16E+08 | 7 | 26678 | oar3_OAR2_116065498 | oar3_OAR2_116092175 |
| 2 | 1.16E+08 | 1.16E+08 | 11 | 31779 | oar3_OAR2_116076349 | OAR2_124098623.1 |
| 2 | 1.16E+08 | 1.16E+08 | 9 | 11148 | s51605.1 | OAR2_124098623.1 |
| 2 | 1.16E+08 | 1.16E+08 | 8 | 9261 | s51605.1 | oar3_OAR2_116106240 |
| 2 | 1.17E+08 | 1.17E+08 | 32 | 140529 | oar3_OAR2_116703428 | oar3_OAR2_116843956 |
| 2 | 1.17E+08 | 1.17E+08 | 17 | 72608 | oar3_OAR2_116715686 | oar3_OAR2_116788293 |
| 2 | 1.17E+08 | 1.17E+08 | 6 | 23458 | oar3_OAR2_116723399 | oar3_OAR2_116746856 |
| 2 | 1.17E+08 | 1.17E+08 | 18 | 61908 | oar3_OAR2_116731875 | oar3_OAR2_116793782 |
| 2 | 1.17E+08 | 1.17E+08 | 14 | 50318 | oar3_OAR2_116742691 | oar3_OAR2_116793008 |
| 2 | 1.17E+08 | 1.17E+08 | 14 | 53207 | oar3_OAR2_116931106 | oar3_OAR2_116984312 |
| 2 | 1.17E+08 | 1.17E+08 | 13 | 35507 | oar3_OAR2_116948806 | oar3_OAR2_116984312 |
| 2 | 1.17E+08 | 1.17E+08 | 13 | 35507 | oar3_OAR2_116948806 | oar3_OAR2_116984312 |
| 2 | 1.17E+08 | 1.17E+08 | 13 | 35507 | oar3_OAR2_116948806 | oar3_OAR2_116984312 |
| 2 | 1.17E+08 | 1.17E+08 | 11 | 56289 | oar3_OAR2_117083576 | oar3_OAR2_117139864 |
| 2 | 1.17E+08 | 1.17E+08 | 11 | 55837 | oar3_OAR2_117097356 | oar3_OAR2_117153192 |
| 2 | 1.17E+08 | 1.17E+08 | 25 | 118366 | oar3_OAR2_117203155 | oar3_OAR2_117321520 |
| 2 | 1.17E+08 | 1.17E+08 | 11 | 41148 | oar3_OAR2_117273438 | oar3_OAR2_117314585 |
| 2 | 1.17E+08 | 1.17E+08 | 11 | 41148 | oar3_OAR2_117273438 | oar3_OAR2_117314585 |
| 2 | 1.21E+08 | 1.21E+08 | 4 | 2429 | oar3_OAR2_120519478 | oar3_OAR2_120521906 |
| 2 | 1.22E+08 | 1.23E+08 | 9 | 23236 | oar3_OAR2_122493288 | oar3_OAR2_122516523 |
| 2 | 1.33E+08 | 1.33E+08 | 9 | 22741 | oar3_OAR2_132818788 | OAR2_141348394.1 |
| 2 | 1.33E+08 | 1.33E+08 | 17 | 72806 | oar3_OAR2_132840574 | oar3_OAR2_132913379 |
| 2 | 1.33E+08 | 1.33E+08 | 11 | 55669 | oar3_OAR2_132857711 | oar3_OAR2_132913379 |
| 2 | 1.33E+08 | 1.33E+08 | 10 | 44598 | s13618.1 | oar3_OAR2_132913379 |
| 2 | 1.33E+08 | 1.33E+08 | 6 | 20041 | oar3_OAR2_132893339 | oar3_OAR2_132913379 |
| 2 | 1.4E+08 | 1.4E+08 | 6 | 18365 | oar3_OAR2_139567358 | oar3_OAR2_139585722 |
| 2 | 1.43E+08 | 1.43E+08 | 12 | 40407 | oar3_OAR2_143368800 | oar3_OAR2_143409206 |
| 2 | 1.45E+08 | 1.45E+08 | 4 | 6864 | oar3_OAR2_144823894 | oar3_OAR2_144830757 |
| 2 | 1.47E+08 | 1.47E+08 | 7 | 23360 | OAR2_156184412.1 | oar3_OAR2_147233572 |
| 2 | 1.47E+08 | 1.47E+08 | 5 | 18535 | OAR2_156184412.1 | oar3_OAR2_147228747 |
| 2 | 1.54E+08 | 1.54E+08 | 4 | 2980 | oar3_OAR2_153577234 | oar3_OAR2_153580213 |
| 2 | 1.62E+08 | 1.62E+08 | 5 | 2937 | oar3_OAR2_161781578 | oar3_OAR2_161784514 |
| 2 | 1.85E+08 | 1.85E+08 | 4 | 9734 | oar3_OAR2_185475923 | oar3_OAR2_185485656 |
| 2 | 1.88E+08 | 1.88E+08 | 3 | 463 | oar3_OAR2_188203683 | OAR2_199620426.1 |
| 2 | 2.05E+08 | 2.05E+08 | 3 | 147 | oar3_OAR2_205186446 | oar3_OAR2_205186592 |
| 2 | 2.09E+08 | 2.09E+08 | 4 | 2180 | oar3_OAR2_209188914 | oar3_OAR2_209191093 |
| 2 | 2.11E+08 | 2.11E+08 | 5 | 15083 | oar3_OAR2_210881102 | oar3_OAR2_210896184 |
| 2 | 2.11E+08 | 2.11E+08 | 9 | 33092 | oar3_OAR2_210881102 | oar3_OAR2_210914193 |
| 2 | 2.19E+08 | 2.19E+08 | 27 | 145239 | oar3_OAR2_218874901 | oar3_OAR2_219020139 |
| 2 | 2.19E+08 | 2.19E+08 | 13 | 48418 | oar3_OAR2_218889476 | oar3_OAR2_218937893 |
| 2 | 2.19E+08 | 2.19E+08 | 13 | 48418 | oar3_OAR2_218889476 | oar3_OAR2_218937893 |
| 2 | 2.19E+08 | 2.19E+08 | 13 | 48418 | oar3_OAR2_218889476 | oar3_OAR2_218937893 |
| 2 | 2.19E+08 | 2.19E+08 | 13 | 48418 | oar3_OAR2_218889476 | oar3_OAR2_218937893 |
| 2 | 2.19E+08 | 2.19E+08 | 13 | 48418 | oar3_OAR2_218889476 | oar3_OAR2_218937893 |
| 2 | 2.19E+08 | 2.19E+08 | 13 | 48418 | oar3_OAR2_218889476 | oar3_OAR2_218937893 |
| 2 | 2.19E+08 | 2.19E+08 | 13 | 48418 | oar3_OAR2_218889476 | oar3_OAR2_218937893 |
| 2 | 2.19E+08 | 2.19E+08 | 24 | 128342 | oar3_OAR2_218926825 | oar3_OAR2_219055166 |
| 2 | 2.2E+08 | 2.2E+08 | 12 | 63978 | oar3_OAR2_219781182 | oar3_OAR2_219845159 |
| 2 | 2.2E+08 | 2.2E+08 | 8 | 10020 | oar3_OAR2_220088311 | oar3_OAR2_220098330 |
| 2 | 2.2E+08 | 2.2E+08 | 23 | 65013 | oar3_OAR2_220092910 | oar3_OAR2_220157922 |
| 2 | 2.2E+08 | 2.2E+08 | 26 | 87511 | oar3_OAR2_220144600 | oar3_OAR2_220232110 |
| 2 | 2.2E+08 | 2.2E+08 | 10 | 49504 | oar3_OAR2_220323194 | oar3_OAR2_220372697 |
| 2 | 2.2E+08 | 2.2E+08 | 8 | 31868 | oar3_OAR2_220340830 | oar3_OAR2_220372697 |
| 2 | 2.2E+08 | 2.21E+08 | 45 | 159569 | oar3_OAR2_220348463 | oar3_OAR2_220508031 |
| 2 | 2.2E+08 | 2.2E+08 | 14 | 46225 | s53222.1 | oar3_OAR2_220404888 |
| 2 | 2.2E+08 | 2.2E+08 | 8 | 22966 | oar3_OAR2_220372697 | oar3_OAR2_220395662 |
| 2 | 2.2E+08 | 2.2E+08 | 7 | 15414 | oar3_OAR2_220385504 | oar3_OAR2_220400917 |
| 2 | 2.2E+08 | 2.2E+08 | 7 | 15414 | oar3_OAR2_220385504 | oar3_OAR2_220400917 |
| 2 | 2.2E+08 | 2.2E+08 | 8 | 19385 | oar3_OAR2_220385504 | oar3_OAR2_220404888 |
| 2 | 2.28E+08 | 2.28E+08 | 9 | 49081 | oar3_OAR2_228082042 | oar3_OAR2_228131122 |
| 2 | 2.32E+08 | 2.32E+08 | 58 | 278176 | oar3_OAR2_232216629 | oar3_OAR2_232494804 |
| 2 | 2.32E+08 | 2.32E+08 | 14 | 45738 | oar3_OAR2_232234842 | oar3_OAR2_232280579 |
| 2 | 2.32E+08 | 2.33E+08 | 55 | 265997 | oar3_OAR2_232234842 | oar3_OAR2_232500838 |
| 2 | 2.32E+08 | 2.32E+08 | 15 | 53447 | oar3_OAR2_232234842 | oar3_OAR2_232288288 |
| 2 | 2.32E+08 | 2.32E+08 | 15 | 53447 | oar3_OAR2_232234842 | oar3_OAR2_232288288 |
| 2 | 2.32E+08 | 2.32E+08 | 14 | 45857 | oar3_OAR2_232242432 | oar3_OAR2_232288288 |
| 2 | 2.32E+08 | 2.32E+08 | 7 | 20631 | oar3_OAR2_232242432 | oar3_OAR2_232263062 |
| 2 | 2.32E+08 | 2.33E+08 | 52 | 243847 | oar3_OAR2_232256992 | oar3_OAR2_232500838 |
| 2 | 2.32E+08 | 2.32E+08 | 5 | 6071 | oar3_OAR2_232256992 | oar3_OAR2_232263062 |
| 2 | 2.32E+08 | 2.32E+08 | 4 | 2343 | oar3_OAR2_232260720 | oar3_OAR2_232263062 |
| 2 | 2.32E+08 | 2.32E+08 | 11 | 27569 | oar3_OAR2_232260720 | oar3_OAR2_232288288 |
| 2 | 2.32E+08 | 2.33E+08 | 24 | 127394 | oar3_OAR2_232373445 | oar3_OAR2_232500838 |
| 2 | 2.32E+08 | 2.32E+08 | 9 | 46350 | oar3_OAR2_232411218 | oar3_OAR2_232457567 |
| 2 | 2.33E+08 | 2.33E+08 | 12 | 53300 | oar3_OAR2_232622752 | oar3_OAR2_232676051 |
| 2 | 2.33E+08 | 2.33E+08 | 12 | 63568 | oar3_OAR2_233188958 | oar3_OAR2_233252525 |
| 2 | 2.33E+08 | 2.33E+08 | 7 | 26264 | oar3_OAR2_233188958 | oar3_OAR2_233215221 |
| 2 | 2.33E+08 | 2.33E+08 | 9 | 39517 | oar3_OAR2_233188958 | oar3_OAR2_233228474 |
| 2 | 2.33E+08 | 2.33E+08 | 7 | 26264 | oar3_OAR2_233188958 | oar3_OAR2_233215221 |
| 2 | 2.33E+08 | 2.33E+08 | 9 | 39517 | oar3_OAR2_233188958 | oar3_OAR2_233228474 |
| 2 | 2.33E+08 | 2.33E+08 | 8 | 33913 | oar3_OAR2_233194562 | oar3_OAR2_233228474 |
| 2 | 2.35E+08 | 2.35E+08 | 24 | 78163 | s10587.1 | oar3_OAR2_234838802 |
| 2 | 2.35E+08 | 2.35E+08 | 10 | 38945 | oar3_OAR2_234789719 | oar3_OAR2_234828663 |
| 2 | 2.35E+08 | 2.35E+08 | 11 | 44695 | oar3_OAR2_234789719 | oar3_OAR2_234834413 |
| 2 | 2.35E+08 | 2.35E+08 | 9 | 32820 | oar3_OAR2_234795844 | oar3_OAR2_234828663 |
| 2 | 2.35E+08 | 2.35E+08 | 5 | 8536 | s12918.1 | oar3_OAR2_234956379 |
| 2 | 2.37E+08 | 2.37E+08 | 9 | 32918 | oar3_OAR2_237290216 | oar3_OAR2_237323133 |
| 2 | 2.39E+08 | 2.39E+08 | 23 | 103885 | oar3_OAR2_238527580 | oar3_OAR2_238631464 |
| 2 | 2.39E+08 | 2.39E+08 | 4 | 8911 | oar3_OAR2_238539528 | oar3_OAR2_238548438 |
| 2 | 2.39E+08 | 2.39E+08 | 12 | 55445 | oar3_OAR2_238769740 | oar3_OAR2_238825184 |
| 2 | 2.39E+08 | 2.39E+08 | 22 | 108163 | oar3_OAR2_238795114 | oar3_OAR2_238903276 |
| 2 | 2.39E+08 | 2.39E+08 | 7 | 20049 | oar3_OAR2_238795114 | oar3_OAR2_238815162 |
| 2 | 2.39E+08 | 2.39E+08 | 13 | 56209 | oar3_OAR2_238802082 | oar3_OAR2_238858290 |
| 2 | 2.39E+08 | 2.39E+08 | 13 | 56209 | oar3_OAR2_238802082 | oar3_OAR2_238858290 |
| 2 | 2.39E+08 | 2.39E+08 | 19 | 70486 | oar3_OAR2_239079026 | OAR2_252561873.1 |
| 2 | 2.39E+08 | 2.39E+08 | 15 | 59958 | oar3_OAR2_239079026 | oar3_OAR2_239138983 |
| 2 | 2.4E+08 | 2.4E+08 | 25 | 85607 | oar3_OAR2_239634241 | oar3_OAR2_239719847 |
| 2 | 2.4E+08 | 2.4E+08 | 22 | 77890 | oar3_OAR2_239634241 | oar3_OAR2_239712130 |
| 2 | 2.4E+08 | 2.4E+08 | 19 | 58382 | s32680.1 | oar3_OAR2_239713498 |
| 2 | 2.4E+08 | 2.4E+08 | 9 | 25099 | oar3_OAR2_239694749 | oar3_OAR2_239719847 |
| 2 | 2.4E+08 | 2.4E+08 | 16 | 67506 | oar3_OAR2_240327561 | oar3_OAR2_240395066 |
| 2 | 2.4E+08 | 2.4E+08 | 9 | 37634 | oar3_OAR2_240357433 | oar3_OAR2_240395066 |
| 2 | 2.4E+08 | 2.4E+08 | 8 | 30934 | oar3_OAR2_240364133 | oar3_OAR2_240395066 |
| 2 | 2.4E+08 | 2.4E+08 | 8 | 30934 | oar3_OAR2_240364133 | oar3_OAR2_240395066 |
| 2 | 2.4E+08 | 2.4E+08 | 8 | 30934 | oar3_OAR2_240364133 | oar3_OAR2_240395066 |
| 2 | 2.4E+08 | 2.4E+08 | 5 | 7946 | oar3_OAR2_240387121 | oar3_OAR2_240395066 |
| 2 | 2.4E+08 | 2.4E+08 | 5 | 7946 | oar3_OAR2_240387121 | oar3_OAR2_240395066 |
| 2 | 2.41E+08 | 2.41E+08 | 27 | 101514 | oar3_OAR2_240735937 | oar3_OAR2_240837450 |
| 2 | 2.41E+08 | 2.41E+08 | 16 | 50325 | oar3_OAR2_240787126 | oar3_OAR2_240837450 |
| 2 | 2.41E+08 | 2.41E+08 | 35 | 180970 | oar3_OAR2_240805136 | oar3_OAR2_240986105 |
| 2 | 2.41E+08 | 2.41E+08 | 12 | 41696 | s00772.1 | oar3_OAR2_240986105 |
| 2 | 2.41E+08 | 2.41E+08 | 11 | 20526 | oar3_OAR2_241460328 | oar3_OAR2_241480853 |
| 2 | 2.41E+08 | 2.41E+08 | 4 | 2735 | oar3_OAR2_241478119 | oar3_OAR2_241480853 |
| 2 | 2.43E+08 | 2.43E+08 | 22 | 100793 | oar3_OAR2_242851610 | oar3_OAR2_242952402 |
| 2 | 2.43E+08 | 2.43E+08 | 20 | 86698 | oar3_OAR2_242865705 | oar3_OAR2_242952402 |
| 2 | 2.43E+08 | 2.43E+08 | 20 | 86698 | oar3_OAR2_242865705 | oar3_OAR2_242952402 |
| 2 | 2.43E+08 | 2.43E+08 | 12 | 40904 | oar3_OAR2_242865705 | oar3_OAR2_242906608 |
| 2 | 2.43E+08 | 2.43E+08 | 12 | 40904 | oar3_OAR2_242865705 | oar3_OAR2_242906608 |
| 2 | 2.43E+08 | 2.43E+08 | 5 | 10645 | oar3_OAR2_242865705 | oar3_OAR2_242876349 |
| 2 | 2.43E+08 | 2.43E+08 | 20 | 86698 | oar3_OAR2_242865705 | oar3_OAR2_242952402 |
| 2 | 2.43E+08 | 2.43E+08 | 6 | 20438 | oar3_OAR2_242931965 | oar3_OAR2_242952402 |
| 2 | 2.43E+08 | 2.43E+08 | 16 | 44036 | oar3_OAR2_243053568 | oar3_OAR2_243097603 |
| 2 | 2.43E+08 | 2.43E+08 | 45 | 135397 | oar3_OAR2_243053568 | oar3_OAR2_243188964 |
| 2 | 2.43E+08 | 2.43E+08 | 13 | 32069 | oar3_OAR2_243053568 | oar3_OAR2_243085636 |
| 2 | 2.43E+08 | 2.43E+08 | 25 | 80934 | oar3_OAR2_243081405 | oar3_OAR2_243162338 |
| 2 | 2.43E+08 | 2.43E+08 | 21 | 55728 | oar3_OAR2_243133237 | oar3_OAR2_243188964 |
| 2 | 2.43E+08 | 2.43E+08 | 12 | 33126 | oar3_OAR2_243133237 | oar3_OAR2_243166362 |
| 2 | 2.43E+08 | 2.43E+08 | 21 | 55728 | oar3_OAR2_243133237 | oar3_OAR2_243188964 |
| 2 | 2.43E+08 | 2.43E+08 | 6 | 9006 | oar3_OAR2_243179959 | oar3_OAR2_243188964 |
| 2 | 2.43E+08 | 2.43E+08 | 6 | 9006 | oar3_OAR2_243179959 | oar3_OAR2_243188964 |
| 2 | 2.43E+08 | 2.43E+08 | 6 | 9006 | oar3_OAR2_243179959 | oar3_OAR2_243188964 |
| 2 | 2.44E+08 | 2.44E+08 | 10 | 33648 | oar3_OAR2_243563568 | oar3_OAR2_243597215 |
| 2 | 2.44E+08 | 2.44E+08 | 11 | 39800 | oar3_OAR2_243563568 | oar3_OAR2_243603367 |
| 2 | 2.44E+08 | 2.44E+08 | 11 | 39800 | oar3_OAR2_243563568 | oar3_OAR2_243603367 |
| 2 | 2.44E+08 | 2.44E+08 | 9 | 26864 | oar3_OAR2_243576504 | oar3_OAR2_243603367 |
| 2 | 2.44E+08 | 2.44E+08 | 9 | 26864 | oar3_OAR2_243576504 | oar3_OAR2_243603367 |
| 2 | 2.44E+08 | 2.44E+08 | 16 | 62543 | oar3_OAR2_243747697 | oar3_OAR2_243810239 |
| 2 | 2.44E+08 | 2.44E+08 | 22 | 88606 | oar3_OAR2_243747697 | oar3_OAR2_243836302 |
| 2 | 2.44E+08 | 2.44E+08 | 20 | 74534 | oar3_OAR2_243763654 | oar3_OAR2_243838187 |
| 2 | 2.44E+08 | 2.44E+08 | 21 | 82416 | oar3_OAR2_243763654 | oar3_OAR2_243846069 |
| 2 | 2.44E+08 | 2.44E+08 | 23 | 84864 | oar3_OAR2_243766788 | oar3_OAR2_243851651 |
| 2 | 2.44E+08 | 2.44E+08 | 19 | 66538 | oar3_OAR2_243785114 | oar3_OAR2_243851651 |
| 2 | 2.44E+08 | 2.44E+08 | 18 | 64265 | oar3_OAR2_243787387 | oar3_OAR2_243851651 |
| 2 | 2.44E+08 | 2.44E+08 | 21 | 65175 | oar3_OAR2_243799555 | oar3_OAR2_243864729 |
| 2 | 2.44E+08 | 2.44E+08 | 17 | 52097 | oar3_OAR2_243799555 | oar3_OAR2_243851651 |
| 2 | 2.44E+08 | 2.44E+08 | 10 | 34076 | oar3_OAR2_243804112 | oar3_OAR2_243838187 |
| 2 | 2.44E+08 | 2.44E+08 | 10 | 34076 | oar3_OAR2_243804112 | oar3_OAR2_243838187 |
| 2 | 2.44E+08 | 2.44E+08 | 9 | 32191 | oar3_OAR2_243804112 | oar3_OAR2_243836302 |
| 2 | 2.44E+08 | 2.44E+08 | 8 | 25344 | oar3_OAR2_243804112 | oar3_OAR2_243829455 |
| 2 | 2.44E+08 | 2.44E+08 | 11 | 35275 | oar3_OAR2_243829455 | oar3_OAR2_243864729 |
| 2 | 2.44E+08 | 2.44E+08 | 5 | 11379 | oar3_OAR2_244055545 | oar3_OAR2_244066923 |
| 2 | 2.44E+08 | 2.44E+08 | 5 | 11379 | oar3_OAR2_244055545 | oar3_OAR2_244066923 |
| 2 | 2.44E+08 | 2.44E+08 | 5 | 11379 | oar3_OAR2_244055545 | oar3_OAR2_244066923 |
| 2 | 2.44E+08 | 2.44E+08 | 5 | 11379 | oar3_OAR2_244055545 | oar3_OAR2_244066923 |
| 2 | 2.44E+08 | 2.44E+08 | 8 | 31721 | oar3_OAR2_244055545 | oar3_OAR2_244087265 |
| 2 | 2.44E+08 | 2.44E+08 | 5 | 11379 | oar3_OAR2_244055545 | oar3_OAR2_244066923 |
| 2 | 2.45E+08 | 2.45E+08 | 10 | 32786 | oar3_OAR2_244802981 | oar3_OAR2_244835766 |
| 2 | 2.45E+08 | 2.45E+08 | 8 | 21926 | oar3_OAR2_244813841 | oar3_OAR2_244835766 |
| 2 | 2.45E+08 | 2.45E+08 | 6 | 12335 | oar3_OAR2_244823432 | oar3_OAR2_244835766 |
| 2 | 2.46E+08 | 2.46E+08 | 14 | 69350 | oar3_OAR2_245794805 | oar3_OAR2_245864154 |
| 2 | 2.46E+08 | 2.46E+08 | 13 | 50090 | oar3_OAR2_245819510 | oar3_OAR2_245869599 |
| 2 | 2.46E+08 | 2.46E+08 | 9 | 32701 | OAR2_260008602.1 | oar3_OAR2_245864154 |
| 2 | 2.46E+08 | 2.46E+08 | 8 | 30515 | OAR2_260008602.1 | oar3_OAR2_245861968 |
| 2 | 2.46E+08 | 2.46E+08 | 9 | 32701 | OAR2_260008602.1 | oar3_OAR2_245864154 |
| 2 | 2.46E+08 | 2.46E+08 | 9 | 32701 | OAR2_260008602.1 | oar3_OAR2_245864154 |
| 2 | 2.46E+08 | 2.46E+08 | 9 | 32701 | OAR2_260008602.1 | oar3_OAR2_245864154 |
| 2 | 2.46E+08 | 2.46E+08 | 18 | 72701 | oar3_OAR2_246016900 | oar3_OAR2_246089600 |
| 2 | 2.46E+08 | 2.46E+08 | 7 | 21008 | oar3_OAR2_246016900 | oar3_OAR2_246037907 |
| 2 | 2.46E+08 | 2.46E+08 | 7 | 21008 | oar3_OAR2_246016900 | oar3_OAR2_246037907 |
| 2 | 2.46E+08 | 2.46E+08 | 5 | 12038 | oar3_OAR2_246024393 | oar3_OAR2_246036430 |
| 2 | 2.46E+08 | 2.46E+08 | 18 | 58061 | oar3_OAR2_246105175 | oar3_OAR2_246163235 |
| 2 | 2.46E+08 | 2.46E+08 | 18 | 58061 | oar3_OAR2_246105175 | oar3_OAR2_246163235 |
| 2 | 2.46E+08 | 2.46E+08 | 18 | 58061 | oar3_OAR2_246105175 | oar3_OAR2_246163235 |
| 2 | 2.46E+08 | 2.46E+08 | 18 | 58061 | oar3_OAR2_246105175 | oar3_OAR2_246163235 |
| 2 | 2.46E+08 | 2.46E+08 | 12 | 43142 | oar3_OAR2_246120094 | oar3_OAR2_246163235 |
| 2 | 2.46E+08 | 2.46E+08 | 7 | 10888 | oar3_OAR2_246247797 | oar3_OAR2_246258684 |
| 2 | 2.46E+08 | 2.46E+08 | 10 | 15326 | oar3_OAR2_246247797 | oar3_OAR2_246263122 |
| 2 | 2.46E+08 | 2.46E+08 | 7 | 10888 | oar3_OAR2_246247797 | oar3_OAR2_246258684 |
| 2 | 2.46E+08 | 2.46E+08 | 4 | 6983 | oar3_OAR2_246251702 | oar3_OAR2_246258684 |
| 2 | 2.46E+08 | 2.46E+08 | 6 | 7339 | oar3_OAR2_246255784 | oar3_OAR2_246263122 |
| 2 | 2.46E+08 | 2.46E+08 | 10 | 27384 | oar3_OAR2_246401464 | oar3_OAR2_246428847 |
| 2 | 2.46E+08 | 2.46E+08 | 12 | 32097 | oar3_OAR2_246410022 | oar3_OAR2_246442118 |
| 2 | 2.46E+08 | 2.46E+08 | 17 | 45121 | oar3_OAR2_246410022 | oar3_OAR2_246455142 |
| 2 | 2.46E+08 | 2.46E+08 | 5 | 6445 | oar3_OAR2_246422403 | oar3_OAR2_246428847 |
| 2 | 2.46E+08 | 2.46E+08 | 8 | 19716 | oar3_OAR2_246422403 | oar3_OAR2_246442118 |
| 2 | 2.46E+08 | 2.46E+08 | 5 | 6445 | oar3_OAR2_246422403 | oar3_OAR2_246428847 |
| 2 | 2.47E+08 | 2.47E+08 | 25 | 115739 | oar3_OAR2_246775189 | oar3_OAR2_246890927 |
| 2 | 2.47E+08 | 2.47E+08 | 5 | 8563 | oar3_OAR2_246910446 | oar3_OAR2_246919008 |
| 2 | 2.47E+08 | 2.47E+08 | 13 | 36490 | oar3_OAR2_247004252 | oar3_OAR2_247040741 |
| 2 | 2.47E+08 | 2.47E+08 | 16 | 40467 | oar3_OAR2_247016432 | oar3_OAR2_247056898 |
| 2 | 2.47E+08 | 2.47E+08 | 6 | 5851 | oar3_OAR2_247016432 | oar3_OAR2_247022282 |
| 2 | 2.47E+08 | 2.47E+08 | 6 | 9187 | oar3_OAR2_247187981 | oar3_OAR2_247197167 |
| 2 | 2.47E+08 | 2.47E+08 | 13 | 22290 | oar3_OAR2_247330118 | oar3_OAR2_247352407 |
| 2 | 2.48E+08 | 2.48E+08 | 8 | 26052 | s58483.1 | oar3_OAR2_247833706 |
| 2 | 2.48E+08 | 2.48E+08 | 18 | 88352 | oar3_OAR2_247813400 | oar3_OAR2_247901751 |
| 2 | 2.48E+08 | 2.48E+08 | 12 | 57111 | oar3_OAR2_247813400 | oar3_OAR2_247870510 |
| 2 | 2.48E+08 | 2.48E+08 | 31 | 145435 | oar3_OAR2_247823905 | oar3_OAR2_247969339 |
| 2 | 2.48E+08 | 2.48E+08 | 45 | 198945 | oar3_OAR2_247823905 | oar3_OAR2_248022849 |
| 2 | 2.48E+08 | 2.48E+08 | 12 | 68046 | oar3_OAR2_247833706 | oar3_OAR2_247901751 |
| 2 | 2.48E+08 | 2.48E+08 | 15 | 53511 | oar3_OAR2_247969339 | oar3_OAR2_248022849 |
| 2 | 2.48E+08 | 2.48E+08 | 15 | 53511 | oar3_OAR2_247969339 | oar3_OAR2_248022849 |
| 2 | 2.48E+08 | 2.48E+08 | 22 | 61751 | oar3_OAR2_248022849 | oar3_OAR2_248084599 |
| 2 | 2.48E+08 | 2.48E+08 | 34 | 135983 | oar3_OAR2_248045390 | oar3_OAR2_248181372 |
| 2 | 2.48E+08 | 2.48E+08 | 25 | 85354 | oar3_OAR2_248066325 | oar3_OAR2_248151678 |
| 2 | 2.48E+08 | 2.48E+08 | 35 | 153627 | oar3_OAR2_248066750 | oar3_OAR2_248220376 |
| 2 | 2.48E+08 | 2.48E+08 | 22 | 79387 | oar3_OAR2_248072292 | oar3_OAR2_248151678 |
| 2 | 2.48E+08 | 2.48E+08 | 32 | 147146 | oar3_OAR2_248082625 | oar3_OAR2_248229770 |
| 2 | 2.48E+08 | 2.48E+08 | 11 | 48399 | oar3_OAR2_248181372 | oar3_OAR2_248229770 |
| 2 | 2.48E+08 | 2.48E+08 | 9 | 39005 | oar3_OAR2_248181372 | oar3_OAR2_248220376 |
| 2 | 2.48E+08 | 2.49E+08 | 20 | 130786 | oar3_OAR2_248370988 | oar3_OAR2_248501773 |
| 2 | 2.48E+08 | 2.49E+08 | 35 | 212192 | oar3_OAR2_248370988 | oar3_OAR2_248583179 |
| 2 | 2.48E+08 | 2.48E+08 | 5 | 28476 | oar3_OAR2_248402190 | oar3_OAR2_248430665 |
| 2 | 2.48E+08 | 2.48E+08 | 5 | 28476 | oar3_OAR2_248402190 | oar3_OAR2_248430665 |
| 2 | 2.48E+08 | 2.48E+08 | 5 | 28476 | oar3_OAR2_248402190 | oar3_OAR2_248430665 |
| 2 | 2.48E+08 | 2.48E+08 | 10 | 54408 | oar3_OAR2_248402190 | oar3_OAR2_248456597 |
| 2 | 2.48E+08 | 2.48E+08 | 5 | 28476 | oar3_OAR2_248402190 | oar3_OAR2_248430665 |
| 2 | 2.48E+08 | 2.48E+08 | 5 | 28476 | oar3_OAR2_248402190 | oar3_OAR2_248430665 |
| 2 | 2.48E+08 | 2.48E+08 | 4 | 19797 | oar3_OAR2_248402190 | oar3_OAR2_248421986 |
| 2 | 2.48E+08 | 2.49E+08 | 30 | 180990 | oar3_OAR2_248402190 | oar3_OAR2_248583179 |
| 2 | 2.49E+08 | 2.49E+08 | 39 | 277522 | oar3_OAR2_248678271 | oar3_OAR2_248955792 |
| 2 | 2.49E+08 | 2.49E+08 | 19 | 146113 | oar3_OAR2_248691771 | oar3_OAR2_248837883 |
| 2 | 2.49E+08 | 2.49E+08 | 14 | 96955 | oar3_OAR2_248740929 | oar3_OAR2_248837883 |
| 2 | 2.49E+08 | 2.49E+08 | 24 | 163795 | oar3_OAR2_248747094 | oar3_OAR2_248910888 |
| 2 | 2.49E+08 | 2.49E+08 | 19 | 130779 | oar3_OAR2_248780110 | oar3_OAR2_248910888 |
| 2 | 2.49E+08 | 2.49E+08 | 10 | 103196 | oar3_OAR2_248780110 | oar3_OAR2_248883305 |
| 2 | 2.49E+08 | 2.49E+08 | 8 | 57774 | oar3_OAR2_248780110 | oar3_OAR2_248837883 |
| 2 | 2.49E+08 | 2.49E+08 | 8 | 57774 | oar3_OAR2_248780110 | oar3_OAR2_248837883 |
| 2 | 2.49E+08 | 2.49E+08 | 19 | 130779 | oar3_OAR2_248780110 | oar3_OAR2_248910888 |
| 2 | 2.49E+08 | 2.49E+08 | 5 | 21001 | oar3_OAR2_248780110 | oar3_OAR2_248801110 |
| 2 | 2.49E+08 | 2.49E+08 | 13 | 90034 | oar3_OAR2_248820855 | oar3_OAR2_248910888 |
| 2 | 2.49E+08 | 2.49E+08 | 8 | 20285 | oar3_OAR2_248890604 | oar3_OAR2_248910888 |
| 2 | 2.49E+08 | 2.49E+08 | 8 | 20285 | oar3_OAR2_248890604 | oar3_OAR2_248910888 |
| 3 | 111156 | 236115 | 19 | 124960 | oar3_OAR3_111156 | oar3_OAR3_236115 |
| 3 | 111156 | 236115 | 19 | 124960 | oar3_OAR3_111156 | oar3_OAR3_236115 |
| 3 | 111156 | 153652 | 7 | 42497 | oar3_OAR3_111156 | oar3_OAR3_153652 |
| 3 | 111156 | 153652 | 7 | 42497 | oar3_OAR3_111156 | oar3_OAR3_153652 |
| 3 | 209682 | 287643 | 12 | 77962 | oar3_OAR3_209682 | oar3_OAR3_287643 |
| 3 | 209682 | 381958 | 26 | 172277 | oar3_OAR3_209682 | oar3_OAR3_381958 |
| 3 | 209682 | 740317 | 71 | 530636 | oar3_OAR3_209682 | oar3_OAR3_740317 |
| 3 | 209682 | 468884 | 37 | 259203 | oar3_OAR3_209682 | oar3_OAR3_468884 |
| 3 | 220814 | 375511 | 24 | 154698 | oar3_OAR3_220814 | oar3_OAR3_375511 |
| 3 | 258479 | 287643 | 5 | 29165 | oar3_OAR3_258479 | oar3_OAR3_287643 |
| 3 | 258479 | 468884 | 30 | 210406 | oar3_OAR3_258479 | oar3_OAR3_468884 |
| 3 | 258479 | 278972 | 4 | 20494 | oar3_OAR3_258479 | oar3_OAR3_278972 |
| 3 | 258479 | 287643 | 5 | 29165 | oar3_OAR3_258479 | oar3_OAR3_287643 |
| 3 | 258479 | 451946 | 28 | 193468 | oar3_OAR3_258479 | oar3_OAR3_451946 |
| 3 | 258479 | 451946 | 28 | 193468 | oar3_OAR3_258479 | oar3_OAR3_451946 |
| 3 | 258479 | 381958 | 19 | 123480 | oar3_OAR3_258479 | oar3_OAR3_381958 |
| 3 | 258479 | 441289 | 26 | 182811 | oar3_OAR3_258479 | oar3_OAR3_441289 |
| 3 | 258479 | 468884 | 30 | 210406 | oar3_OAR3_258479 | oar3_OAR3_468884 |
| 3 | 422167 | 543919 | 21 | 121753 | oar3_OAR3_422167 | oar3_OAR3_543919 |
| 3 | 543919 | 740317 | 24 | 196399 | oar3_OAR3_543919 | oar3_OAR3_740317 |
| 3 | 543919 | 935319 | 51 | 391401 | oar3_OAR3_543919 | oar3_OAR3_935319 |
| 3 | 583181 | 935319 | 48 | 352139 | oar3_OAR3_583181 | oar3_OAR3_935319 |
| 3 | 610356 | 740317 | 19 | 129962 | oar3_OAR3_610356 | oar3_OAR3_740317 |
| 3 | 610356 | 825364 | 26 | 215009 | oar3_OAR3_610356 | oar3_OAR3_825364 |
| 3 | 637192 | 703856 | 10 | 66665 | oar3_OAR3_637192 | oar3_OAR3_703856 |
| 3 | 637192 | 675050 | 4 | 37859 | oar3_OAR3_637192 | oar3_OAR3_675050 |
| 3 | 637192 | 792458 | 22 | 155267 | oar3_OAR3_637192 | oar3_OAR3_792458 |
| 3 | 637192 | 1530377 | 187 | 893186 | oar3_OAR3_637192 | oar3_OAR3_1530377 |
| 3 | 637192 | 675050 | 4 | 37859 | oar3_OAR3_637192 | oar3_OAR3_675050 |
| 3 | 675050 | 758988 | 17 | 83939 | oar3_OAR3_675050 | oar3_OAR3_758988 |
| 3 | 691553 | 700550 | 5 | 8998 | oar3_OAR3_691553 | oar3_OAR3_700550 |
| 3 | 738269 | 743283 | 4 | 5015 | oar3_OAR3_738269 | oar3_OAR3_743283 |
| 3 | 875107 | 935319 | 17 | 60213 | oar3_OAR3_875107 | oar3_OAR3_935319 |
| 3 | 886456 | 935319 | 14 | 48864 | oar3_OAR3_886456 | oar3_OAR3_935319 |
| 3 | 891897 | 935319 | 13 | 43423 | oar3_OAR3_891897 | oar3_OAR3_935319 |
| 3 | 891897 | 935319 | 13 | 43423 | oar3_OAR3_891897 | oar3_OAR3_935319 |
| 3 | 909644 | 935319 | 8 | 25676 | oar3_OAR3_909644 | oar3_OAR3_935319 |
| 3 | 909644 | 1062020 | 27 | 152377 | oar3_OAR3_909644 | oar3_OAR3_1062020 |
| 3 | 1000283 | 1062020 | 9 | 61738 | oar3_OAR3_1000283 | oar3_OAR3_1062020 |
| 3 | 1000283 | 1194831 | 41 | 194549 | oar3_OAR3_1000283 | oar3_OAR3_1194831 |
| 3 | 1021695 | 1132879 | 24 | 111185 | oar3_OAR3_1021695 | OAR3_1194797.1 |
| 3 | 1021695 | 1076557 | 10 | 54863 | oar3_OAR3_1021695 | oar3_OAR3_1076557 |
| 3 | 1021695 | 1243074 | 52 | 221380 | oar3_OAR3_1021695 | oar3_OAR3_1243074 |
| 3 | 1039605 | 1194831 | 38 | 155227 | oar3_OAR3_1039605 | oar3_OAR3_1194831 |
| 3 | 1042326 | 1082692 | 12 | 40367 | oar3_OAR3_1042326 | oar3_OAR3_1082692 |
| 3 | 1061428 | 1194831 | 34 | 133404 | oar3_OAR3_1061428 | oar3_OAR3_1194831 |
| 3 | 1076373 | 1132879 | 17 | 56507 | oar3_OAR3_1076373 | OAR3_1194797.1 |
| 3 | 1094166 | 1194831 | 25 | 100666 | oar3_OAR3_1094166 | oar3_OAR3_1194831 |
| 3 | 1131242 | 1206154 | 22 | 74913 | oar3_OAR3_1131242 | oar3_OAR3_1206154 |
| 3 | 1132479 | 1194831 | 17 | 62353 | oar3_OAR3_1132479 | oar3_OAR3_1194831 |
| 3 | 1132479 | 1194831 | 17 | 62353 | oar3_OAR3_1132479 | oar3_OAR3_1194831 |
| 3 | 1150620 | 1206154 | 16 | 55535 | oar3_OAR3_1150620 | oar3_OAR3_1206154 |
| 3 | 1150620 | 1206154 | 16 | 55535 | oar3_OAR3_1150620 | oar3_OAR3_1206154 |
| 3 | 1173729 | 1194831 | 8 | 21103 | oar3_OAR3_1173729 | oar3_OAR3_1194831 |
| 3 | 1183374 | 1204410 | 10 | 21037 | oar3_OAR3_1183374 | oar3_OAR3_1204410 |
| 3 | 1183374 | 1204410 | 10 | 21037 | oar3_OAR3_1183374 | oar3_OAR3_1204410 |
| 3 | 1234298 | 1403536 | 50 | 169239 | oar3_OAR3_1234298 | oar3_OAR3_1403536 |
| 3 | 1242546 | 1268624 | 14 | 26079 | oar3_OAR3_1242546 | oar3_OAR3_1268624 |
| 3 | 1242546 | 1423236 | 54 | 180691 | oar3_OAR3_1242546 | oar3_OAR3_1423236 |
| 3 | 1279453 | 1423236 | 40 | 143784 | oar3_OAR3_1279453 | oar3_OAR3_1423236 |
| 3 | 1311682 | 1430607 | 34 | 118926 | oar3_OAR3_1311682 | oar3_OAR3_1430607 |
| 3 | 1363448 | 1472558 | 31 | 109111 | oar3_OAR3_1363448 | oar3_OAR3_1472558 |
| 3 | 1363448 | 1404282 | 12 | 40835 | oar3_OAR3_1363448 | oar3_OAR3_1404282 |
| 3 | 1374662 | 1412008 | 14 | 37347 | oar3_OAR3_1374662 | oar3_OAR3_1412008 |
| 3 | 1374662 | 1386105 | 9 | 11444 | oar3_OAR3_1374662 | s73218.1 |
| 3 | 1374662 | 1469487 | 29 | 94826 | oar3_OAR3_1374662 | oar3_OAR3_1469487 |
| 3 | 1377814 | 1423236 | 14 | 45423 | oar3_OAR3_1377814 | oar3_OAR3_1423236 |
| 3 | 1380267 | 1430607 | 13 | 50341 | oar3_OAR3_1380267 | oar3_OAR3_1430607 |
| 3 | 1381308 | 1845573 | 79 | 464266 | oar3_OAR3_1381308 | oar3_OAR3_1845573 |
| 3 | 1443099 | 1462550 | 7 | 19452 | oar3_OAR3_1443099 | oar3_OAR3_1462550 |
| 3 | 1444163 | 1541007 | 23 | 96845 | oar3_OAR3_1444163 | oar3_OAR3_1541007 |
| 3 | 1456608 | 1472558 | 7 | 15951 | oar3_OAR3_1456608 | oar3_OAR3_1472558 |
| 3 | 1461932 | 1472558 | 6 | 10627 | oar3_OAR3_1461932 | oar3_OAR3_1472558 |
| 3 | 1461932 | 1608537 | 28 | 146606 | oar3_OAR3_1461932 | oar3_OAR3_1608537 |
| 3 | 1461932 | 1488553 | 8 | 26622 | oar3_OAR3_1461932 | oar3_OAR3_1488553 |
| 3 | 1462550 | 1581495 | 24 | 118946 | oar3_OAR3_1462550 | oar3_OAR3_1581495 |
| 3 | 1519413 | 1581495 | 12 | 62083 | oar3_OAR3_1519413 | oar3_OAR3_1581495 |
| 3 | 1519413 | 1581495 | 12 | 62083 | oar3_OAR3_1519413 | oar3_OAR3_1581495 |
| 3 | 1573779 | 1581495 | 4 | 7717 | oar3_OAR3_1573779 | oar3_OAR3_1581495 |
| 3 | 1621192 | 1845139 | 28 | 223948 | oar3_OAR3_1621192 | oar3_OAR3_1845139 |
| 3 | 1643897 | 1862454 | 25 | 218558 | oar3_OAR3_1643897 | oar3_OAR3_1862454 |
| 3 | 1643897 | 1840760 | 20 | 196864 | oar3_OAR3_1643897 | oar3_OAR3_1840760 |
| 3 | 1643897 | 1867569 | 26 | 223673 | oar3_OAR3_1643897 | oar3_OAR3_1867569 |
| 3 | 1643897 | 2114992 | 54 | 471096 | oar3_OAR3_1643897 | oar3_OAR3_2114992 |
| 3 | 1646553 | 1675552 | 6 | 29000 | oar3_OAR3_1646553 | oar3_OAR3_1675552 |
| 3 | 1646553 | 1678538 | 7 | 31986 | oar3_OAR3_1646553 | oar3_OAR3_1678538 |
| 3 | 1656073 | 1844155 | 19 | 188083 | oar3_OAR3_1656073 | oar3_OAR3_1844155 |
| 3 | 1656073 | 1845573 | 21 | 189501 | oar3_OAR3_1656073 | oar3_OAR3_1845573 |
| 3 | 1656073 | 1703872 | 10 | 47800 | oar3_OAR3_1656073 | oar3_OAR3_1703872 |
| 3 | 1656073 | 1903574 | 28 | 247502 | oar3_OAR3_1656073 | s60310.1 |
| 3 | 1656073 | 1853050 | 22 | 196978 | oar3_OAR3_1656073 | oar3_OAR3_1853050 |
| 3 | 1656073 | 1840760 | 18 | 184688 | oar3_OAR3_1656073 | oar3_OAR3_1840760 |
| 3 | 1660557 | 1695301 | 6 | 34745 | oar3_OAR3_1660557 | oar3_OAR3_1695301 |
| 3 | 1672660 | 1730909 | 10 | 58250 | oar3_OAR3_1672660 | oar3_OAR3_1730909 |
| 3 | 1672660 | 1845139 | 17 | 172480 | oar3_OAR3_1672660 | oar3_OAR3_1845139 |
| 3 | 1691284 | 1703872 | 4 | 12589 | oar3_OAR3_1691284 | oar3_OAR3_1703872 |
| 3 | 1703010 | 1730909 | 5 | 27900 | oar3_OAR3_1703010 | oar3_OAR3_1730909 |
| 3 | 1728466 | 1867569 | 14 | 139104 | oar3_OAR3_1728466 | oar3_OAR3_1867569 |
| 3 | 1728466 | 1840760 | 8 | 112295 | oar3_OAR3_1728466 | oar3_OAR3_1840760 |
| 3 | 1769826 | 1845573 | 8 | 75748 | oar3_OAR3_1769826 | oar3_OAR3_1845573 |
| 3 | 1941796 | 2071947 | 16 | 130152 | oar3_OAR3_1941796 | oar3_OAR3_2071947 |
| 3 | 1951716 | 2013140 | 7 | 61425 | oar3_OAR3_1951716 | oar3_OAR3_2013140 |
| 3 | 1951716 | 2013140 | 7 | 61425 | oar3_OAR3_1951716 | oar3_OAR3_2013140 |
| 3 | 1951716 | 2125715 | 22 | 174000 | oar3_OAR3_1951716 | oar3_OAR3_2125715 |
| 3 | 1951716 | 2211162 | 44 | 259447 | oar3_OAR3_1951716 | s08515.1 |
| 3 | 1951716 | 2013140 | 7 | 61425 | oar3_OAR3_1951716 | oar3_OAR3_2013140 |
| 3 | 1965718 | 2071947 | 13 | 106230 | oar3_OAR3_1965718 | oar3_OAR3_2071947 |
| 3 | 1965718 | 2013140 | 5 | 47423 | oar3_OAR3_1965718 | oar3_OAR3_2013140 |
| 3 | 1965718 | 2013140 | 5 | 47423 | oar3_OAR3_1965718 | oar3_OAR3_2013140 |
| 3 | 1987849 | 2185229 | 31 | 197381 | oar3_OAR3_1987849 | oar3_OAR3_2185229 |
| 3 | 1987849 | 2013140 | 4 | 25292 | oar3_OAR3_1987849 | oar3_OAR3_2013140 |
| 3 | 2053535 | 2144563 | 15 | 91029 | oar3_OAR3_2053535 | oar3_OAR3_2144563 |
| 3 | 2058608 | 2144563 | 13 | 85956 | oar3_OAR3_2058608 | oar3_OAR3_2144563 |
| 3 | 2180386 | 2276580 | 35 | 96195 | oar3_OAR3_2180386 | oar3_OAR3_2276580 |
| 3 | 2190891 | 2274343 | 30 | 83453 | oar3_OAR3_2190891 | oar3_OAR3_2274343 |
| 3 | 2195631 | 2334881 | 43 | 139251 | oar3_OAR3_2195631 | oar3_OAR3_2334881 |
| 3 | 2237297 | 2288321 | 17 | 51025 | oar3_OAR3_2237297 | oar3_OAR3_2288321 |
| 3 | 2254945 | 2288321 | 13 | 33377 | oar3_OAR3_2254945 | oar3_OAR3_2288321 |
| 3 | 2254945 | 2288321 | 13 | 33377 | oar3_OAR3_2254945 | oar3_OAR3_2288321 |
| 3 | 2254945 | 2288321 | 13 | 33377 | oar3_OAR3_2254945 | oar3_OAR3_2288321 |
| 3 | 2254945 | 2279119 | 10 | 24175 | oar3_OAR3_2254945 | oar3_OAR3_2279119 |
| 3 | 2254945 | 2276580 | 8 | 21636 | oar3_OAR3_2254945 | oar3_OAR3_2276580 |
| 3 | 2254945 | 2288321 | 13 | 33377 | oar3_OAR3_2254945 | oar3_OAR3_2288321 |
| 3 | 2258450 | 2274343 | 6 | 15894 | oar3_OAR3_2258450 | oar3_OAR3_2274343 |
| 3 | 2312875 | 2362094 | 19 | 49220 | oar3_OAR3_2312875 | oar3_OAR3_2362094 |
| 3 | 2334881 | 2374172 | 15 | 39292 | oar3_OAR3_2334881 | oar3_OAR3_2374172 |
| 3 | 2334881 | 2428124 | 22 | 93244 | oar3_OAR3_2334881 | oar3_OAR3_2428124 |
| 3 | 2359514 | 2454841 | 16 | 95328 | oar3_OAR3_2359514 | oar3_OAR3_2454841 |
| 3 | 2472161 | 2520927 | 14 | 48767 | oar3_OAR3_2472161 | oar3_OAR3_2520927 |
| 3 | 2472161 | 2569023 | 28 | 96863 | oar3_OAR3_2472161 | oar3_OAR3_2569023 |
| 3 | 2490631 | 2520927 | 10 | 30297 | oar3_OAR3_2490631 | oar3_OAR3_2520927 |
| 3 | 2490631 | 2527932 | 13 | 37302 | oar3_OAR3_2490631 | oar3_OAR3_2527932 |
| 3 | 2490631 | 2550083 | 18 | 59453 | oar3_OAR3_2490631 | oar3_OAR3_2550083 |
| 3 | 2689767 | 2946077 | 40 | 256311 | oar3_OAR3_2689767 | oar3_OAR3_2946077 |
| 3 | 2689767 | 2753924 | 14 | 64158 | oar3_OAR3_2689767 | s52389.1 |
| 3 | 2689767 | 2718699 | 8 | 28933 | oar3_OAR3_2689767 | oar3_OAR3_2718699 |
| 3 | 2691998 | 2768487 | 13 | 76490 | OAR3_2505765.1 | oar3_OAR3_2768487 |
| 3 | 2691998 | 2753924 | 12 | 61927 | OAR3_2505765.1 | s52389.1 |
| 3 | 2692064 | 2753924 | 11 | 61861 | oar3_OAR3_2692064 | s52389.1 |
| 3 | 2704871 | 2753924 | 9 | 49054 | oar3_OAR3_2704871 | s52389.1 |
| 3 | 2704871 | 2815489 | 13 | 110619 | oar3_OAR3_2704871 | s12435.1 |
| 3 | 2708567 | 2768487 | 9 | 59921 | oar3_OAR3_2708567 | oar3_OAR3_2768487 |
| 3 | 2718699 | 2808623 | 10 | 89925 | oar3_OAR3_2718699 | oar3_OAR3_2808623 |
| 3 | 2739875 | 2753924 | 3 | 14050 | oar3_OAR3_2739875 | s52389.1 |
| 3 | 2739875 | 2815489 | 7 | 75615 | oar3_OAR3_2739875 | s12435.1 |
| 3 | 2855071 | 2908941 | 6 | 53871 | oar3_OAR3_2855071 | oar3_OAR3_2908941 |
| 3 | 2855071 | 2945025 | 18 | 89955 | oar3_OAR3_2855071 | oar3_OAR3_2945025 |
| 3 | 2900358 | 3056545 | 33 | 156188 | oar3_OAR3_2900358 | oar3_OAR3_3056545 |
| 3 | 2917942 | 2937719 | 9 | 19778 | oar3_OAR3_2917942 | oar3_OAR3_2937719 |
| 3 | 2922815 | 2946077 | 10 | 23263 | oar3_OAR3_2922815 | oar3_OAR3_2946077 |
| 3 | 2922815 | 3056545 | 26 | 133731 | oar3_OAR3_2922815 | oar3_OAR3_3056545 |
| 3 | 2930228 | 3241497 | 60 | 311270 | oar3_OAR3_2930228 | oar3_OAR3_3241497 |
| 3 | 2945025 | 2994815 | 8 | 49791 | oar3_OAR3_2945025 | oar3_OAR3_2994815 |
| 3 | 2982182 | 2998911 | 5 | 16730 | oar3_OAR3_2982182 | oar3_OAR3_2998911 |
| 3 | 2982182 | 3039415 | 11 | 57234 | oar3_OAR3_2982182 | oar3_OAR3_3039415 |
| 3 | 2994815 | 2998911 | 4 | 4097 | oar3_OAR3_2994815 | oar3_OAR3_2998911 |
| 3 | 2996949 | 2998911 | 3 | 1963 | oar3_OAR3_2996949 | oar3_OAR3_2998911 |
| 3 | 2996949 | 3236287 | 46 | 239339 | oar3_OAR3_2996949 | oar3_OAR3_3236287 |
| 3 | 2996949 | 3056545 | 10 | 59597 | oar3_OAR3_2996949 | oar3_OAR3_3056545 |
| 3 | 3039415 | 3114287 | 19 | 74873 | oar3_OAR3_3039415 | oar3_OAR3_3114287 |
| 3 | 3114287 | 3209748 | 17 | 95462 | oar3_OAR3_3114287 | oar3_OAR3_3209748 |
| 3 | 3114287 | 3137442 | 6 | 23156 | oar3_OAR3_3114287 | oar3_OAR3_3137442 |
| 3 | 3114287 | 3276700 | 28 | 162414 | oar3_OAR3_3114287 | oar3_OAR3_3276700 |
| 3 | 3120310 | 3241497 | 20 | 121188 | oar3_OAR3_3120310 | oar3_OAR3_3241497 |
| 3 | 3123890 | 3164824 | 7 | 40935 | oar3_OAR3_3123890 | oar3_OAR3_3164824 |
| 3 | 3154371 | 3207602 | 10 | 53232 | oar3_OAR3_3154371 | oar3_OAR3_3207602 |
| 3 | 3307094 | 3533912 | 50 | 226819 | oar3_OAR3_3307094 | s47103.1 |
| 3 | 3307094 | 3330774 | 9 | 23681 | oar3_OAR3_3307094 | oar3_OAR3_3330774 |
| 3 | 3307094 | 3376406 | 17 | 69313 | oar3_OAR3_3307094 | oar3_OAR3_3376406 |
| 3 | 3309928 | 3341072 | 10 | 31145 | s00499.1 | s07270.1 |
| 3 | 3309928 | 3341072 | 10 | 31145 | s00499.1 | s07270.1 |
| 3 | 3309928 | 3376406 | 15 | 66479 | s00499.1 | oar3_OAR3_3376406 |
| 3 | 3309928 | 3376406 | 15 | 66479 | s00499.1 | oar3_OAR3_3376406 |
| 3 | 3330082 | 3490449 | 33 | 160368 | oar3_OAR3_3330082 | oar3_OAR3_3490449 |
| 3 | 3362012 | 3490449 | 27 | 128438 | oar3_OAR3_3362012 | oar3_OAR3_3490449 |
| 3 | 3421416 | 3523587 | 21 | 102172 | oar3_OAR3_3421416 | oar3_OAR3_3523587 |
| 3 | 3458153 | 3533912 | 17 | 75760 | oar3_OAR3_3458153 | s47103.1 |
| 3 | 3473658 | 3567661 | 21 | 94004 | OAR3_3313767.1 | oar3_OAR3_3567661 |
| 3 | 3490449 | 3533912 | 12 | 43464 | oar3_OAR3_3490449 | s47103.1 |
| 3 | 3490449 | 3533912 | 12 | 43464 | oar3_OAR3_3490449 | s47103.1 |
| 3 | 3490449 | 3533912 | 12 | 43464 | oar3_OAR3_3490449 | s47103.1 |
| 3 | 3561754 | 3602339 | 9 | 40586 | oar3_OAR3_3561754 | oar3_OAR3_3602339 |
| 3 | 3567661 | 3602339 | 8 | 34679 | oar3_OAR3_3567661 | oar3_OAR3_3602339 |
| 3 | 3689255 | 3740262 | 15 | 51008 | oar3_OAR3_3689255 | oar3_OAR3_3740262 |
| 3 | 3717536 | 3793164 | 19 | 75629 | oar3_OAR3_3717536 | OAR3_3767714.1 |
| 3 | 3717536 | 3764164 | 14 | 46629 | oar3_OAR3_3717536 | oar3_OAR3_3764164 |
| 3 | 3719011 | 3747824 | 11 | 28814 | s03864.1 | oar3_OAR3_3747824 |
| 3 | 3723920 | 3764164 | 11 | 40245 | oar3_OAR3_3723920 | oar3_OAR3_3764164 |
| 3 | 3723920 | 3747824 | 9 | 23905 | oar3_OAR3_3723920 | oar3_OAR3_3747824 |
| 3 | 3723920 | 3816302 | 23 | 92383 | oar3_OAR3_3723920 | oar3_OAR3_3816302 |
| 3 | 3723920 | 3816302 | 23 | 92383 | oar3_OAR3_3723920 | oar3_OAR3_3816302 |
| 3 | 3723920 | 3740262 | 7 | 16343 | oar3_OAR3_3723920 | oar3_OAR3_3740262 |
| 3 | 3740262 | 3747824 | 3 | 7563 | oar3_OAR3_3740262 | oar3_OAR3_3747824 |
| 3 | 3758453 | 3816302 | 14 | 57850 | oar3_OAR3_3758453 | oar3_OAR3_3816302 |
| 3 | 4181953 | 4219835 | 10 | 37883 | s70408.1 | oar3_OAR3_4219835 |
| 3 | 4201867 | 4208181 | 4 | 6315 | s14082.1 | oar3_OAR3_4208181 |
| 3 | 4538050 | 4543840 | 6 | 5791 | oar3_OAR3_4538050 | oar3_OAR3_4543840 |
| 3 | 4538050 | 4543840 | 6 | 5791 | oar3_OAR3_4538050 | oar3_OAR3_4543840 |
| 3 | 4542820 | 4543840 | 3 | 1021 | oar3_OAR3_4542820 | oar3_OAR3_4543840 |
| 3 | 5011133 | 5154509 | 41 | 143377 | oar3_OAR3_5011133 | oar3_OAR3_5154509 |
| 3 | 5067855 | 5154509 | 26 | 86655 | oar3_OAR3_5067855 | oar3_OAR3_5154509 |
| 3 | 5353244 | 5481147 | 32 | 127904 | oar3_OAR3_5353244 | oar3_OAR3_5481147 |
| 3 | 5554377 | 5560883 | 5 | 6507 | oar3_OAR3_5554377 | oar3_OAR3_5560883 |
| 3 | 5554377 | 5633973 | 30 | 79597 | oar3_OAR3_5554377 | oar3_OAR3_5633973 |
| 3 | 5554377 | 5662488 | 36 | 108112 | oar3_OAR3_5554377 | oar3_OAR3_5662488 |
| 3 | 5554377 | 5571089 | 9 | 16713 | oar3_OAR3_5554377 | oar3_OAR3_5571089 |
| 3 | 5596192 | 5633973 | 12 | 37782 | oar3_OAR3_5596192 | oar3_OAR3_5633973 |
| 3 | 5602018 | 5633973 | 10 | 31956 | oar3_OAR3_5602018 | oar3_OAR3_5633973 |
| 3 | 5628908 | 5633973 | 3 | 5066 | oar3_OAR3_5628908 | oar3_OAR3_5633973 |
| 3 | 5628908 | 5695709 | 26 | 66802 | oar3_OAR3_5628908 | s59028.1 |
| 3 | 5679091 | 5695709 | 10 | 16619 | oar3_OAR3_5679091 | s59028.1 |
| 3 | 5679091 | 5727502 | 13 | 48412 | oar3_OAR3_5679091 | oar3_OAR3_5727502 |
| 3 | 5889053 | 5940273 | 14 | 51221 | oar3_OAR3_5889053 | oar3_OAR3_5940273 |
| 3 | 5894299 | 5950571 | 17 | 56273 | oar3_OAR3_5894299 | oar3_OAR3_5950571 |
| 3 | 6071584 | 6139807 | 25 | 68224 | oar3_OAR3_6071584 | oar3_OAR3_6139807 |
| 3 | 6125879 | 6158784 | 18 | 32906 | oar3_OAR3_6125879 | oar3_OAR3_6158784 |
| 3 | 6188464 | 6218373 | 12 | 29910 | oar3_OAR3_6188464 | oar3_OAR3_6218373 |
| 3 | 6188464 | 6243676 | 16 | 55213 | oar3_OAR3_6188464 | s66434.1 |
| 3 | 6188464 | 6218373 | 12 | 29910 | oar3_OAR3_6188464 | oar3_OAR3_6218373 |
| 3 | 6214579 | 6243676 | 9 | 29098 | oar3_OAR3_6214579 | s66434.1 |
| 3 | 6217569 | 6243676 | 7 | 26108 | oar3_OAR3_6217569 | s66434.1 |
| 3 | 6345205 | 6381927 | 11 | 36723 | oar3_OAR3_6345205 | oar3_OAR3_6381927 |
| 3 | 6971384 | 6985751 | 6 | 14368 | oar3_OAR3_6971384 | oar3_OAR3_6985751 |
| 3 | 6971384 | 7031900 | 22 | 60517 | oar3_OAR3_6971384 | oar3_OAR3_7031900 |
| 3 | 7023652 | 7054120 | 12 | 30469 | s71470.1 | oar3_OAR3_7054120 |
| 3 | 7023652 | 7031900 | 8 | 8249 | s71470.1 | oar3_OAR3_7031900 |
| 3 | 7023652 | 7028446 | 7 | 4795 | s71470.1 | oar3_OAR3_7028446 |
| 3 | 7023652 | 7119366 | 21 | 95715 | s71470.1 | oar3_OAR3_7119366 |
| 3 | 7023652 | 7031900 | 8 | 8249 | s71470.1 | oar3_OAR3_7031900 |
| 3 | 7314242 | 7382352 | 13 | 68111 | oar3_OAR3_7314242 | oar3_OAR3_7382352 |
| 3 | 7314242 | 7393881 | 15 | 79640 | oar3_OAR3_7314242 | oar3_OAR3_7393881 |
| 3 | 7314242 | 7393881 | 15 | 79640 | oar3_OAR3_7314242 | oar3_OAR3_7393881 |
| 3 | 7452243 | 7483263 | 8 | 31021 | oar3_OAR3_7452243 | oar3_OAR3_7483263 |
| 3 | 7482628 | 7874053 | 73 | 391426 | oar3_OAR3_7482628 | oar3_OAR3_7874053 |
| 3 | 7516773 | 7537800 | 9 | 21028 | oar3_OAR3_7516773 | oar3_OAR3_7537800 |
| 3 | 7790658 | 7802848 | 3 | 12191 | oar3_OAR3_7790658 | oar3_OAR3_7802848 |
| 3 | 7981071 | 8008158 | 11 | 27088 | oar3_OAR3_7981071 | oar3_OAR3_8008158 |
| 3 | 7999886 | 8025839 | 12 | 25954 | oar3_OAR3_7999886 | oar3_OAR3_8025839 |
| 3 | 8042257 | 8066076 | 8 | 23820 | oar3_OAR3_8042257 | oar3_OAR3_8066076 |
| 3 | 8054971 | 8123731 | 19 | 68761 | oar3_OAR3_8054971 | oar3_OAR3_8123731 |
| 3 | 8062811 | 8097580 | 12 | 34770 | oar3_OAR3_8062811 | oar3_OAR3_8097580 |
| 3 | 8358071 | 8416746 | 8 | 58676 | oar3_OAR3_8358071 | oar3_OAR3_8416746 |
| 3 | 8925213 | 8966869 | 14 | 41657 | oar3_OAR3_8925213 | oar3_OAR3_8966869 |
| 3 | 8947077 | 8966869 | 9 | 19793 | oar3_OAR3_8947077 | oar3_OAR3_8966869 |
| 3 | 9348876 | 9434046 | 24 | 85171 | oar3_OAR3_9348876 | oar3_OAR3_9434046 |
| 3 | 9362095 | 9425907 | 17 | 63813 | oar3_OAR3_9362095 | oar3_OAR3_9425907 |
| 3 | 9403715 | 9434046 | 9 | 30332 | oar3_OAR3_9403715 | oar3_OAR3_9434046 |
| 3 | 9403715 | 9428422 | 7 | 24708 | oar3_OAR3_9403715 | s61491.1 |
| 3 | 10574201 | 10640801 | 17 | 66601 | oar3_OAR3_10574201 | oar3_OAR3_10640801 |
| 3 | 10574201 | 10640801 | 17 | 66601 | oar3_OAR3_10574201 | oar3_OAR3_10640801 |
| 3 | 10574201 | 10640801 | 17 | 66601 | oar3_OAR3_10574201 | oar3_OAR3_10640801 |
| 3 | 10574201 | 10610439 | 11 | 36239 | oar3_OAR3_10574201 | oar3_OAR3_10610439 |
| 3 | 10574201 | 10640801 | 17 | 66601 | oar3_OAR3_10574201 | oar3_OAR3_10640801 |
| 3 | 10574201 | 10595214 | 9 | 21014 | oar3_OAR3_10574201 | OAR3_11124571.1 |
| 3 | 11299226 | 11339179 | 8 | 39954 | oar3_OAR3_11299226 | oar3_OAR3_11339179 |
| 3 | 12297884 | 12362464 | 23 | 64581 | oar3_OAR3_12297884 | oar3_OAR3_12362464 |
| 3 | 12297884 | 12344437 | 16 | 46554 | oar3_OAR3_12297884 | oar3_OAR3_12344437 |
| 3 | 12306478 | 12324188 | 10 | 17711 | oar3_OAR3_12306478 | s38563.1 |
| 3 | 12306478 | 12342345 | 13 | 35868 | oar3_OAR3_12306478 | oar3_OAR3_12342345 |
| 3 | 12322302 | 12345633 | 11 | 23332 | oar3_OAR3_12322302 | oar3_OAR3_12345633 |
| 3 | 12838484 | 12881108 | 11 | 42625 | oar3_OAR3_12838484 | oar3_OAR3_12881108 |
| 3 | 13156550 | 13209741 | 12 | 53192 | oar3_OAR3_13156550 | oar3_OAR3_13209741 |
| 3 | 13532931 | 13543181 | 4 | 10251 | oar3_OAR3_13532931 | oar3_OAR3_13543181 |
| 3 | 13532931 | 13565592 | 11 | 32662 | oar3_OAR3_13532931 | oar3_OAR3_13565592 |
| 3 | 13994453 | 14061856 | 12 | 67404 | oar3_OAR3_13994453 | oar3_OAR3_14061856 |
| 3 | 17941886 | 18071046 | 29 | 129161 | oar3_OAR3_17941886 | oar3_OAR3_18071046 |
| 3 | 17947021 | 18009895 | 13 | 62875 | oar3_OAR3_17947021 | oar3_OAR3_18009895 |
| 3 | 18056783 | 18067059 | 3 | 10277 | oar3_OAR3_18056783 | oar3_OAR3_18067059 |
| 3 | 18545630 | 18545769 | 3 | 140 | oar3_OAR3_18545630 | oar3_OAR3_18545769 |
| 3 | 19105792 | 19133211 | 9 | 27420 | oar3_OAR3_19105792 | oar3_OAR3_19133211 |
| 3 | 19105792 | 19148660 | 13 | 42869 | oar3_OAR3_19105792 | oar3_OAR3_19148660 |
| 3 | 19273531 | 19340837 | 12 | 67307 | oar3_OAR3_19273531 | oar3_OAR3_19340837 |
| 3 | 19434986 | 19474844 | 16 | 39859 | oar3_OAR3_19434986 | oar3_OAR3_19474844 |
| 3 | 19490990 | 19596054 | 28 | 105065 | oar3_OAR3_19490990 | s26580.1 |
| 3 | 28259087 | 28420322 | 41 | 161236 | oar3_OAR3_28259087 | oar3_OAR3_28420322 |
| 3 | 28345851 | 28406600 | 15 | 60750 | oar3_OAR3_28345851 | oar3_OAR3_28406600 |
| 3 | 31290318 | 31346150 | 20 | 55833 | oar3_OAR3_31290318 | oar3_OAR3_31346150 |
| 3 | 31290318 | 31343166 | 18 | 52849 | oar3_OAR3_31290318 | oar3_OAR3_31343166 |
| 3 | 31317417 | 31343166 | 11 | 25750 | oar3_OAR3_31317417 | oar3_OAR3_31343166 |
| 3 | 32574575 | 32657471 | 11 | 82897 | oar3_OAR3_32574575 | oar3_OAR3_32657471 |
| 3 | 33274225 | 33295877 | 6 | 21653 | oar3_OAR3_33274225 | oar3_OAR3_33295877 |
| 3 | 33466650 | 33522202 | 18 | 55553 | oar3_OAR3_33466650 | s12256.1 |
| 3 | 33489137 | 33551428 | 19 | 62292 | oar3_OAR3_33489137 | oar3_OAR3_33551428 |
| 3 | 33499618 | 33612654 | 29 | 113037 | oar3_OAR3_33499618 | oar3_OAR3_33612654 |
| 3 | 33502777 | 33551428 | 15 | 48652 | oar3_OAR3_33502777 | oar3_OAR3_33551428 |
| 3 | 33508498 | 33551428 | 14 | 42931 | oar3_OAR3_33508498 | oar3_OAR3_33551428 |
| 3 | 33708022 | 33797634 | 23 | 89613 | oar3_OAR3_33708022 | oar3_OAR3_33797634 |
| 3 | 33781345 | 33830272 | 16 | 48928 | oar3_OAR3_33781345 | oar3_OAR3_33830272 |
| 3 | 33781345 | 33791270 | 7 | 9926 | oar3_OAR3_33781345 | oar3_OAR3_33791270 |
| 3 | 34041600 | 34080445 | 8 | 38846 | oar3_OAR3_34041600 | oar3_OAR3_34080445 |
| 3 | 34313228 | 34364400 | 10 | 51173 | oar3_OAR3_34313228 | s14929.1 |
| 3 | 34313228 | 34350595 | 7 | 37368 | oar3_OAR3_34313228 | oar3_OAR3_34350595 |
| 3 | 34350311 | 34376804 | 9 | 26494 | oar3_OAR3_34350311 | oar3_OAR3_34376804 |
| 3 | 35784560 | 35809734 | 16 | 25175 | oar3_OAR3_35784560 | oar3_OAR3_35809734 |
| 3 | 35790165 | 35810032 | 16 | 19868 | oar3_OAR3_35790165 | oar3_OAR3_35810032 |
| 3 | 35802228 | 35809734 | 11 | 7507 | oar3_OAR3_35802228 | oar3_OAR3_35809734 |
| 3 | 35905481 | 35950290 | 14 | 44810 | oar3_OAR3_35905481 | oar3_OAR3_35950290 |
| 3 | 37605152 | 37625348 | 7 | 20197 | oar3_OAR3_37605152 | oar3_OAR3_37625348 |
| 3 | 37615117 | 37635492 | 8 | 20376 | oar3_OAR3_37615117 | oar3_OAR3_37635492 |
| 3 | 38278214 | 38287967 | 7 | 9754 | oar3_OAR3_38278214 | oar3_OAR3_38287967 |
| 3 | 38278214 | 38287967 | 7 | 9754 | oar3_OAR3_38278214 | oar3_OAR3_38287967 |
| 3 | 38278214 | 38287967 | 7 | 9754 | oar3_OAR3_38278214 | oar3_OAR3_38287967 |
| 3 | 43547252 | 43569721 | 9 | 22470 | oar3_OAR3_43547252 | oar3_OAR3_43569721 |
| 3 | 43619307 | 43620728 | 4 | 1422 | oar3_OAR3_43619307 | oar3_OAR3_43620728 |
| 3 | 43619307 | 43620728 | 4 | 1422 | oar3_OAR3_43619307 | oar3_OAR3_43620728 |
| 3 | 53556398 | 53557393 | 3 | 996 | oar3_OAR3_53556398 | oar3_OAR3_53557393 |
| 3 | 55043378 | 55100423 | 11 | 57046 | oar3_OAR3_55043378 | oar3_OAR3_55100423 |
| 3 | 55043378 | 55103529 | 12 | 60152 | oar3_OAR3_55043378 | oar3_OAR3_55103529 |
| 3 | 55043378 | 55103529 | 12 | 60152 | oar3_OAR3_55043378 | oar3_OAR3_55103529 |
| 3 | 57052098 | 57099430 | 12 | 47333 | oar3_OAR3_57052098 | oar3_OAR3_57099430 |
| 3 | 57474170 | 57560618 | 17 | 86449 | oar3_OAR3_57474170 | oar3_OAR3_57560618 |
| 3 | 62193929 | 62226905 | 17 | 32977 | oar3_OAR3_62193929 | oar3_OAR3_62226905 |
| 3 | 62193929 | 62273409 | 35 | 79481 | oar3_OAR3_62193929 | oar3_OAR3_62273409 |
| 3 | 62193929 | 62207780 | 9 | 13852 | oar3_OAR3_62193929 | oar3_OAR3_62207780 |
| 3 | 62243033 | 62359579 | 31 | 116547 | oar3_OAR3_62243033 | oar3_OAR3_62359579 |
| 3 | 62258389 | 62316105 | 19 | 57717 | oar3_OAR3_62258389 | oar3_OAR3_62316105 |
| 3 | 62267131 | 62273409 | 8 | 6279 | oar3_OAR3_62267131 | oar3_OAR3_62273409 |
| 3 | 62267131 | 62273409 | 8 | 6279 | oar3_OAR3_62267131 | oar3_OAR3_62273409 |
| 3 | 77173863 | 77217756 | 15 | 43894 | oar3_OAR3_77173863 | oar3_OAR3_77217756 |
| 3 | 77195138 | 77294306 | 28 | 99169 | oar3_OAR3_77195138 | oar3_OAR3_77294306 |
| 3 | 77195138 | 77260820 | 20 | 65683 | oar3_OAR3_77195138 | oar3_OAR3_77260820 |
| 3 | 77195138 | 77251719 | 17 | 56582 | oar3_OAR3_77195138 | oar3_OAR3_77251719 |
| 3 | 77195138 | 77217756 | 10 | 22619 | oar3_OAR3_77195138 | oar3_OAR3_77217756 |
| 3 | 80959430 | 81080103 | 30 | 120674 | oar3_OAR3_80959430 | oar3_OAR3_81080103 |
| 3 | 81281725 | 81304608 | 12 | 22884 | oar3_OAR3_81281725 | oar3_OAR3_81304608 |
| 3 | 81294629 | 81310268 | 12 | 15640 | oar3_OAR3_81294629 | oar3_OAR3_81310268 |
| 3 | 81297315 | 81310268 | 10 | 12954 | oar3_OAR3_81297315 | oar3_OAR3_81310268 |
| 3 | 81297315 | 81304608 | 7 | 7294 | oar3_OAR3_81297315 | oar3_OAR3_81304608 |
| 3 | 81297315 | 81310268 | 10 | 12954 | oar3_OAR3_81297315 | oar3_OAR3_81310268 |
| 3 | 81297315 | 81310268 | 10 | 12954 | oar3_OAR3_81297315 | oar3_OAR3_81310268 |
| 3 | 81297315 | 81310268 | 10 | 12954 | oar3_OAR3_81297315 | oar3_OAR3_81310268 |
| 3 | 83071074 | 83074472 | 3 | 3399 | oar3_OAR3_83071074 | oar3_OAR3_83074472 |
| 3 | 88141194 | 89620468 | 272 | 1479275 | oar3_OAR3_88141194 | oar3_OAR3_89620468 |
| 3 | 90939977 | 90950840 | 7 | 10864 | oar3_OAR3_90939977 | oar3_OAR3_90950840 |
| 3 | 92537473 | 92541466 | 3 | 3994 | oar3_OAR3_92537473 | oar3_OAR3_92541466 |
| 3 | 92864639 | 93000594 | 32 | 135956 | oar3_OAR3_92864639 | oar3_OAR3_93000594 |
| 3 | 93948311 | 94033320 | 18 | 85010 | oar3_OAR3_93948311 | oar3_OAR3_94033320 |
| 3 | 93987345 | 94033320 | 10 | 45976 | oar3_OAR3_93987345 | oar3_OAR3_94033320 |
| 3 | 93987345 | 94033320 | 10 | 45976 | oar3_OAR3_93987345 | oar3_OAR3_94033320 |
| 3 | 93987345 | 94033320 | 10 | 45976 | oar3_OAR3_93987345 | oar3_OAR3_94033320 |
| 3 | 94007313 | 94033320 | 7 | 26008 | oar3_OAR3_94007313 | oar3_OAR3_94033320 |
| 3 | 94007313 | 94033320 | 7 | 26008 | oar3_OAR3_94007313 | oar3_OAR3_94033320 |
| 3 | 96223760 | 96259466 | 11 | 35707 | oar3_OAR3_96223760 | oar3_OAR3_96259466 |
| 3 | 96223760 | 96304846 | 18 | 81087 | oar3_OAR3_96223760 | oar3_OAR3_96304846 |
| 3 | 97114370 | 97162831 | 9 | 48462 | oar3_OAR3_97114370 | oar3_OAR3_97162831 |
| 3 | 97114370 | 97133343 | 4 | 18974 | oar3_OAR3_97114370 | oar3_OAR3_97133343 |
| 3 | 97187127 | 97201209 | 7 | 14083 | s33987.1 | oar3_OAR3_97201209 |
| 3 | 97392236 | 97421123 | 7 | 28888 | oar3_OAR3_97392236 | oar3_OAR3_97421123 |
| 3 | 97399532 | 97421123 | 6 | 21592 | oar3_OAR3_97399532 | oar3_OAR3_97421123 |
| 3 | 1.02E+08 | 1.02E+08 | 3 | 2321 | oar3_OAR3_101674630 | oar3_OAR3_101676950 |
| 3 | 1.02E+08 | 1.02E+08 | 3 | 2321 | oar3_OAR3_101674630 | oar3_OAR3_101676950 |
| 3 | 1.02E+08 | 1.02E+08 | 3 | 2321 | oar3_OAR3_101674630 | oar3_OAR3_101676950 |
| 3 | 1.03E+08 | 1.03E+08 | 16 | 71702 | oar3_OAR3_102630095 | oar3_OAR3_102701796 |
| 3 | 1.03E+08 | 1.03E+08 | 13 | 32710 | s65226.1 | oar3_OAR3_103287634 |
| 3 | 1.03E+08 | 1.03E+08 | 7 | 13011 | s65226.1 | oar3_OAR3_103267935 |
| 3 | 1.03E+08 | 1.03E+08 | 11 | 24142 | oar3_OAR3_103263493 | oar3_OAR3_103287634 |
| 3 | 1.03E+08 | 1.03E+08 | 15 | 38608 | oar3_OAR3_103348284 | oar3_OAR3_103386891 |
| 3 | 1.04E+08 | 1.04E+08 | 16 | 66474 | oar3_OAR3_103569278 | oar3_OAR3_103635751 |
| 3 | 1.04E+08 | 1.04E+08 | 22 | 142773 | oar3_OAR3_103788334 | oar3_OAR3_103931106 |
| 3 | 1.04E+08 | 1.04E+08 | 62 | 226531 | oar3_OAR3_103990787 | s33128.1 |
| 3 | 1.04E+08 | 1.04E+08 | 61 | 218305 | oar3_OAR3_103999013 | s33128.1 |
| 3 | 1.04E+08 | 1.04E+08 | 17 | 53021 | oar3_OAR3_104081169 | oar3_OAR3_104134189 |
| 3 | 1.04E+08 | 1.04E+08 | 17 | 53021 | oar3_OAR3_104081169 | oar3_OAR3_104134189 |
| 3 | 1.15E+08 | 1.15E+08 | 5 | 8054 | oar3_OAR3_114683776 | OAR3_122249804.1 |
| 3 | 1.2E+08 | 1.2E+08 | 6 | 25644 | oar3_OAR3_120371624 | oar3_OAR3_120397267 |
| 3 | 1.2E+08 | 1.2E+08 | 6 | 25644 | oar3_OAR3_120371624 | oar3_OAR3_120397267 |
| 3 | 1.2E+08 | 1.2E+08 | 4 | 8573 | oar3_OAR3_120401806 | oar3_OAR3_120410378 |
| 3 | 1.33E+08 | 1.33E+08 | 4 | 12443 | oar3_OAR3_133241541 | oar3_OAR3_133253983 |
| 3 | 1.34E+08 | 1.34E+08 | 14 | 51629 | oar3_OAR3_133612019 | oar3_OAR3_133663647 |
| 3 | 1.36E+08 | 1.37E+08 | 31 | 109130 | oar3_OAR3_136414555 | oar3_OAR3_136523684 |
| 3 | 1.36E+08 | 1.36E+08 | 16 | 41374 | s34624.1 | oar3_OAR3_136462862 |
| 3 | 1.36E+08 | 1.37E+08 | 35 | 134786 | s34624.1 | oar3_OAR3_136556274 |
| 3 | 1.38E+08 | 1.38E+08 | 17 | 76091 | oar3_OAR3_137528405 | oar3_OAR3_137604495 |
| 3 | 1.38E+08 | 1.38E+08 | 14 | 60302 | oar3_OAR3_137544194 | oar3_OAR3_137604495 |
| 3 | 1.38E+08 | 1.38E+08 | 13 | 55933 | oar3_OAR3_137548563 | oar3_OAR3_137604495 |
| 3 | 1.38E+08 | 1.38E+08 | 9 | 35962 | oar3_OAR3_138142760 | oar3_OAR3_138178721 |
| 3 | 1.38E+08 | 1.39E+08 | 34 | 107157 | oar3_OAR3_138468336 | oar3_OAR3_138575492 |
| 3 | 1.39E+08 | 1.39E+08 | 42 | 136822 | oar3_OAR3_138535174 | oar3_OAR3_138671995 |
| 3 | 1.39E+08 | 1.39E+08 | 19 | 48683 | oar3_OAR3_138548870 | oar3_OAR3_138597552 |
| 3 | 1.39E+08 | 1.39E+08 | 22 | 67543 | oar3_OAR3_138548870 | oar3_OAR3_138616412 |
| 3 | 1.39E+08 | 1.39E+08 | 6 | 10884 | oar3_OAR3_138564609 | oar3_OAR3_138575492 |
| 3 | 1.39E+08 | 1.39E+08 | 9 | 18087 | oar3_OAR3_138653909 | oar3_OAR3_138671995 |
| 3 | 1.58E+08 | 1.58E+08 | 3 | 14913 | oar3_OAR3_158425348 | oar3_OAR3_158440260 |
| 3 | 1.62E+08 | 1.62E+08 | 18 | 72687 | oar3_OAR3_161578909 | oar3_OAR3_161651595 |
| 3 | 1.62E+08 | 1.62E+08 | 15 | 57463 | oar3_OAR3_161594133 | oar3_OAR3_161651595 |
| 3 | 1.62E+08 | 1.62E+08 | 18 | 45117 | oar3_OAR3_161756800 | oar3_OAR3_161801916 |
| 3 | 1.62E+08 | 1.62E+08 | 15 | 22254 | oar3_OAR3_161756800 | oar3_OAR3_161779053 |
| 3 | 1.62E+08 | 1.62E+08 | 15 | 22254 | oar3_OAR3_161756800 | oar3_OAR3_161779053 |
| 3 | 1.62E+08 | 1.62E+08 | 15 | 22254 | oar3_OAR3_161756800 | oar3_OAR3_161779053 |
| 3 | 1.62E+08 | 1.62E+08 | 42 | 132123 | oar3_OAR3_161756800 | oar3_OAR3_161888922 |
| 3 | 1.62E+08 | 1.62E+08 | 17 | 43767 | oar3_OAR3_161758150_dup | oar3_OAR3_161801916 |
| 3 | 1.62E+08 | 1.62E+08 | 14 | 20904 | oar3_OAR3_161758150_dup | oar3_OAR3_161779053 |
| 3 | 1.62E+08 | 1.62E+08 | 14 | 20904 | oar3_OAR3_161758150_dup | oar3_OAR3_161779053 |
| 3 | 1.62E+08 | 1.62E+08 | 7 | 27719 | oar3_OAR3_161765878 | oar3_OAR3_161793596 |
| 3 | 1.62E+08 | 1.62E+08 | 7 | 27719 | oar3_OAR3_161765878 | oar3_OAR3_161793596 |
| 3 | 1.62E+08 | 1.62E+08 | 14 | 80604 | oar3_OAR3_162078846 | oar3_OAR3_162159449 |
| 3 | 1.62E+08 | 1.62E+08 | 9 | 46441 | oar3_OAR3_162078846 | oar3_OAR3_162125286 |
| 3 | 1.62E+08 | 1.62E+08 | 6 | 41954 | oar3_OAR3_162098118 | oar3_OAR3_162140071 |
| 3 | 1.62E+08 | 1.62E+08 | 8 | 24814 | OAR3_173687235.1 | oar3_OAR3_162435809 |
| 3 | 1.64E+08 | 1.64E+08 | 3 | 83712 | oar3_OAR3_164008733 | oar3_OAR3_164092444 |
| 3 | 1.76E+08 | 1.76E+08 | 6 | 24597 | oar3_OAR3_176052365 | oar3_OAR3_176076961 |
| 3 | 1.79E+08 | 1.79E+08 | 30 | 98473 | oar3_OAR3_178627891 | oar3_OAR3_178726363 |
| 3 | 1.79E+08 | 1.79E+08 | 32 | 115835 | oar3_OAR3_178627891 | s60816.1 |
| 3 | 1.79E+08 | 1.79E+08 | 26 | 85475 | oar3_OAR3_178635074 | oar3_OAR3_178720548 |
| 3 | 1.79E+08 | 1.79E+08 | 9 | 23704 | oar3_OAR3_178696845 | oar3_OAR3_178720548 |
| 3 | 1.79E+08 | 1.79E+08 | 11 | 29519 | oar3_OAR3_178696845 | oar3_OAR3_178726363 |
| 3 | 1.79E+08 | 1.79E+08 | 11 | 29519 | oar3_OAR3_178696845 | oar3_OAR3_178726363 |
| 3 | 1.79E+08 | 1.79E+08 | 5 | 13934 | oar3_OAR3_178702144 | oar3_OAR3_178716077 |
| 3 | 1.79E+08 | 1.79E+08 | 3 | 12384 | oar3_OAR3_179472069 | oar3_OAR3_179484452 |
| 3 | 1.8E+08 | 1.8E+08 | 10 | 14925 | oar3_OAR3_179541586 | oar3_OAR3_179556510 |
| 3 | 1.8E+08 | 1.8E+08 | 3 | 2439 | oar3_OAR3_179554072 | oar3_OAR3_179556510 |
| 3 | 1.8E+08 | 1.8E+08 | 14 | 45871 | oar3_OAR3_179554072 | oar3_OAR3_179599942 |
| 3 | 1.8E+08 | 1.8E+08 | 3 | 2439 | oar3_OAR3_179554072 | oar3_OAR3_179556510 |
| 3 | 1.8E+08 | 1.81E+08 | 10 | 52518 | oar3_OAR3_180469720 | oar3_OAR3_180522237 |
| 3 | 1.84E+08 | 1.84E+08 | 3 | 13090 | oar3_OAR3_183597863 | oar3_OAR3_183610952 |
| 3 | 1.86E+08 | 1.86E+08 | 7 | 13836 | oar3_OAR3_185840467 | oar3_OAR3_185854302 |
| 3 | 1.86E+08 | 1.86E+08 | 7 | 13836 | oar3_OAR3_185840467 | oar3_OAR3_185854302 |
| 3 | 1.86E+08 | 1.86E+08 | 7 | 13836 | oar3_OAR3_185840467 | oar3_OAR3_185854302 |
| 3 | 1.86E+08 | 1.86E+08 | 7 | 13836 | oar3_OAR3_185840467 | oar3_OAR3_185854302 |
| 3 | 1.86E+08 | 1.86E+08 | 7 | 13836 | oar3_OAR3_185840467 | oar3_OAR3_185854302 |
| 3 | 1.86E+08 | 1.86E+08 | 7 | 13836 | oar3_OAR3_185840467 | oar3_OAR3_185854302 |
| 3 | 1.95E+08 | 1.95E+08 | 4 | 8776 | oar3_OAR3_195244208 | oar3_OAR3_195252983 |
| 3 | 2.04E+08 | 2.04E+08 | 3 | 25092 | oar3_OAR3_203982300 | oar3_OAR3_204007391 |
| 3 | 2.07E+08 | 2.07E+08 | 7 | 25170 | oar3_OAR3_207204888 | oar3_OAR3_207230057 |
| 3 | 2.07E+08 | 2.07E+08 | 5 | 5010 | oar3_OAR3_207282916 | oar3_OAR3_207287925 |
| 3 | 2.07E+08 | 2.07E+08 | 10 | 36712 | oar3_OAR3_207436102 | oar3_OAR3_207472813 |
| 3 | 2.08E+08 | 2.08E+08 | 86 | 431596 | oar3_OAR3_207526431 | s49588.1 |
| 3 | 2.08E+08 | 2.08E+08 | 12 | 38961 | oar3_OAR3_207588900 | oar3_OAR3_207627860 |
| 3 | 2.08E+08 | 2.08E+08 | 8 | 14728 | oar3_OAR3_207613133 | oar3_OAR3_207627860 |
| 3 | 2.08E+08 | 2.08E+08 | 8 | 14728 | oar3_OAR3_207613133 | oar3_OAR3_207627860 |
| 3 | 2.08E+08 | 2.08E+08 | 12 | 36820 | oar3_OAR3_207613133 | oar3_OAR3_207649952 |
| 3 | 2.08E+08 | 2.08E+08 | 10 | 32219 | oar3_OAR3_207613133 | oar3_OAR3_207645351 |
| 3 | 2.08E+08 | 2.08E+08 | 8 | 31089 | oar3_OAR3_207618864 | oar3_OAR3_207649952 |
| 3 | 2.08E+08 | 2.08E+08 | 8 | 31089 | oar3_OAR3_207618864 | oar3_OAR3_207649952 |
| 3 | 2.08E+08 | 2.08E+08 | 4 | 8997 | oar3_OAR3_207618864 | oar3_OAR3_207627860 |
| 3 | 2.08E+08 | 2.08E+08 | 5 | 19462 | s33636.1 | oar3_OAR3_207795143 |
| 3 | 2.08E+08 | 2.08E+08 | 22 | 91291 | oar3_OAR3_207803064 | oar3_OAR3_207894354 |
| 3 | 2.08E+08 | 2.08E+08 | 5 | 8589 | oar3_OAR3_207808872 | oar3_OAR3_207817460 |
| 3 | 2.08E+08 | 2.08E+08 | 14 | 36708 | oar3_OAR3_208268794 | oar3_OAR3_208305501 |
| 3 | 2.1E+08 | 2.1E+08 | 11 | 36732 | oar3_OAR3_210259595 | oar3_OAR3_210296326 |
| 3 | 2.1E+08 | 2.1E+08 | 11 | 36732 | oar3_OAR3_210259595 | oar3_OAR3_210296326 |
| 3 | 2.1E+08 | 2.1E+08 | 3 | 4584 | oar3_OAR3_210259595 | oar3_OAR3_210264178 |
| 3 | 2.1E+08 | 2.1E+08 | 3 | 4584 | oar3_OAR3_210259595 | oar3_OAR3_210264178 |
| 3 | 2.1E+08 | 2.1E+08 | 8 | 20190 | oar3_OAR3_210472818 | oar3_OAR3_210493007 |
| 3 | 2.11E+08 | 2.11E+08 | 15 | 59896 | s10034.1 | oar3_OAR3_210790799 |
| 3 | 2.11E+08 | 2.11E+08 | 12 | 35776 | OAR3_228560068.1 | oar3_OAR3_210951536 |
| 3 | 2.11E+08 | 2.11E+08 | 12 | 35776 | OAR3_228560068.1 | oar3_OAR3_210951536 |
| 3 | 2.11E+08 | 2.11E+08 | 11 | 31479 | oar3_OAR3_210920058 | oar3_OAR3_210951536 |
| 3 | 2.11E+08 | 2.11E+08 | 6 | 21407 | oar3_OAR3_210920058 | oar3_OAR3_210941464 |
| 3 | 2.11E+08 | 2.11E+08 | 11 | 31479 | oar3_OAR3_210920058 | oar3_OAR3_210951536 |
| 3 | 2.11E+08 | 2.11E+08 | 7 | 22946 | oar3_OAR3_210920058 | oar3_OAR3_210943003 |
| 3 | 2.11E+08 | 2.11E+08 | 50 | 207715 | oar3_OAR3_210981588 | oar3_OAR3_211189302 |
| 3 | 2.11E+08 | 2.11E+08 | 21 | 83731 | oar3_OAR3_211088738 | oar3_OAR3_211172468 |
| 3 | 2.11E+08 | 2.11E+08 | 15 | 57935 | s70631.1 | oar3_OAR3_211174537 |
| 3 | 2.11E+08 | 2.11E+08 | 10 | 45580 | oar3_OAR3_211126889 | oar3_OAR3_211172468 |
| 3 | 2.11E+08 | 2.11E+08 | 10 | 45580 | oar3_OAR3_211126889 | oar3_OAR3_211172468 |
| 3 | 2.12E+08 | 2.12E+08 | 11 | 25718 | oar3_OAR3_211679206 | oar3_OAR3_211704923 |
| 3 | 2.12E+08 | 2.12E+08 | 11 | 22381 | oar3_OAR3_211684917 | oar3_OAR3_211707297 |
| 3 | 2.12E+08 | 2.12E+08 | 11 | 22381 | oar3_OAR3_211684917 | oar3_OAR3_211707297 |
| 3 | 2.12E+08 | 2.12E+08 | 17 | 83477 | oar3_OAR3_212055171 | oar3_OAR3_212138647 |
| 3 | 2.12E+08 | 2.12E+08 | 18 | 58206 | oar3_OAR3_212223340 | oar3_OAR3_212281545 |
| 3 | 2.12E+08 | 2.12E+08 | 10 | 35028 | oar3_OAR3_212226804 | oar3_OAR3_212261831 |
| 3 | 2.12E+08 | 2.12E+08 | 19 | 58897 | oar3_OAR3_212231083 | oar3_OAR3_212289979 |
| 3 | 2.12E+08 | 2.12E+08 | 9 | 30749 | oar3_OAR3_212231083 | oar3_OAR3_212261831 |
| 3 | 2.12E+08 | 2.12E+08 | 9 | 30749 | oar3_OAR3_212231083 | oar3_OAR3_212261831 |
| 3 | 2.12E+08 | 2.12E+08 | 6 | 20467 | oar3_OAR3_212231083 | oar3_OAR3_212251549 |
| 3 | 2.12E+08 | 2.12E+08 | 6 | 20467 | oar3_OAR3_212231083 | oar3_OAR3_212251549 |
| 3 | 2.12E+08 | 2.12E+08 | 6 | 20467 | oar3_OAR3_212231083 | oar3_OAR3_212251549 |
| 3 | 2.13E+08 | 2.13E+08 | 10 | 31864 | oar3_OAR3_212755162 | oar3_OAR3_212787025 |
| 3 | 2.13E+08 | 2.13E+08 | 17 | 51904 | oar3_OAR3_212851018 | oar3_OAR3_212902921 |
| 3 | 2.13E+08 | 2.14E+08 | 31 | 197190 | oar3_OAR3_213374165 | oar3_OAR3_213571354 |
| 3 | 2.13E+08 | 2.14E+08 | 27 | 185631 | oar3_OAR3_213385724 | oar3_OAR3_213571354 |
| 3 | 2.13E+08 | 2.14E+08 | 27 | 185631 | oar3_OAR3_213385724 | oar3_OAR3_213571354 |
| 3 | 2.13E+08 | 2.14E+08 | 22 | 133323 | oar3_OAR3_213408849 | oar3_OAR3_213542171 |
| 3 | 2.13E+08 | 2.14E+08 | 38 | 221379 | oar3_OAR3_213408849 | oar3_OAR3_213630227 |
| 3 | 2.13E+08 | 2.14E+08 | 38 | 221379 | oar3_OAR3_213408849 | oar3_OAR3_213630227 |
| 3 | 2.13E+08 | 2.14E+08 | 14 | 82710 | oar3_OAR3_213459462 | oar3_OAR3_213542171 |
| 3 | 2.14E+08 | 2.14E+08 | 21 | 103243 | oar3_OAR3_213526985 | oar3_OAR3_213630227 |
| 3 | 2.14E+08 | 2.14E+08 | 20 | 76881 | oar3_OAR3_213831212 | oar3_OAR3_213908092 |
| 3 | 2.14E+08 | 2.14E+08 | 11 | 47588 | oar3_OAR3_213831212 | oar3_OAR3_213878799 |
| 3 | 2.14E+08 | 2.14E+08 | 12 | 45865 | oar3_OAR3_213836231 | oar3_OAR3_213882095 |
| 3 | 2.14E+08 | 2.14E+08 | 12 | 31296 | oar3_OAR3_213876797 | oar3_OAR3_213908092 |
| 3 | 2.14E+08 | 2.14E+08 | 10 | 88242 | oar3_OAR3_213977733 | oar3_OAR3_214065974 |
| 3 | 2.14E+08 | 2.14E+08 | 20 | 131785 | oar3_OAR3_214002125 | oar3_OAR3_214133909 |
| 3 | 2.14E+08 | 2.14E+08 | 16 | 80616 | OAR3_232015599.1 | oar3_OAR3_214127214 |
| 3 | 2.14E+08 | 2.14E+08 | 6 | 17142 | oar3_OAR3_214116768 | oar3_OAR3_214133909 |
| 3 | 2.14E+08 | 2.14E+08 | 8 | 17569 | oar3_OAR3_214283044 | oar3_OAR3_214300612 |
| 3 | 2.14E+08 | 2.14E+08 | 5 | 9311 | oar3_OAR3_214341354 | oar3_OAR3_214350664 |
| 3 | 2.14E+08 | 2.14E+08 | 7 | 25240 | oar3_OAR3_214422360 | oar3_OAR3_214447599 |
| 3 | 2.15E+08 | 2.15E+08 | 16 | 53190 | oar3_OAR3_214651731 | s69353.1 |
| 3 | 2.15E+08 | 2.15E+08 | 19 | 63074 | oar3_OAR3_214658837 | oar3_OAR3_214721910 |
| 3 | 2.15E+08 | 2.15E+08 | 32 | 110487 | oar3_OAR3_214871049 | oar3_OAR3_214981535 |
| 3 | 2.15E+08 | 2.15E+08 | 23 | 96170 | oar3_OAR3_215026732 | oar3_OAR3_215122901 |
| 3 | 2.15E+08 | 2.15E+08 | 20 | 74849 | oar3_OAR3_215048053 | oar3_OAR3_215122901 |
| 3 | 2.15E+08 | 2.15E+08 | 22 | 84525 | oar3_OAR3_215048053 | oar3_OAR3_215132577 |
| 3 | 2.17E+08 | 2.17E+08 | 8 | 41560 | oar3_OAR3_216899371 | s11874.1 |
| 3 | 2.17E+08 | 2.17E+08 | 12 | 50031 | oar3_OAR3_217238451 | oar3_OAR3_217288481 |
| 3 | 2.17E+08 | 2.17E+08 | 9 | 47633 | oar3_OAR3_217259103 | oar3_OAR3_217306735 |
| 3 | 2.17E+08 | 2.17E+08 | 7 | 29379 | oar3_OAR3_217259103 | oar3_OAR3_217288481 |
| 3 | 2.17E+08 | 2.17E+08 | 7 | 29379 | oar3_OAR3_217259103 | oar3_OAR3_217288481 |
| 3 | 2.17E+08 | 2.17E+08 | 10 | 51863 | oar3_OAR3_217259103 | s46664.1 |
| 3 | 2.17E+08 | 2.17E+08 | 10 | 51863 | oar3_OAR3_217259103 | s46664.1 |
| 3 | 2.18E+08 | 2.18E+08 | 53 | 204705 | oar3_OAR3_217857297 | oar3_OAR3_218062001 |
| 3 | 2.18E+08 | 2.18E+08 | 16 | 59139 | oar3_OAR3_217930036 | oar3_OAR3_217989174 |
| 3 | 2.18E+08 | 2.18E+08 | 5 | 5343 | oar3_OAR3_217932069 | oar3_OAR3_217937411 |
| 3 | 2.18E+08 | 2.18E+08 | 4 | 10556 | oar3_OAR3_217968819 | oar3_OAR3_217979374 |
| 3 | 2.18E+08 | 2.18E+08 | 13 | 50829 | oar3_OAR3_217970376 | oar3_OAR3_218021204 |
| 3 | 2.18E+08 | 2.18E+08 | 4 | 9895 | oar3_OAR3_218041405 | oar3_OAR3_218051299 |
| 3 | 2.18E+08 | 2.18E+08 | 7 | 20597 | oar3_OAR3_218041405 | oar3_OAR3_218062001 |
| 3 | 2.18E+08 | 2.18E+08 | 4 | 9895 | oar3_OAR3_218041405 | oar3_OAR3_218051299 |
| 3 | 2.18E+08 | 2.18E+08 | 4 | 9895 | oar3_OAR3_218041405 | oar3_OAR3_218051299 |
| 3 | 2.18E+08 | 2.18E+08 | 3 | 3878 | oar3_OAR3_218047422 | oar3_OAR3_218051299 |
| 3 | 2.18E+08 | 2.18E+08 | 32 | 124831 | oar3_OAR3_218174319 | oar3_OAR3_218299149 |
| 3 | 2.19E+08 | 2.19E+08 | 11 | 64530 | oar3_OAR3_218542410 | oar3_OAR3_218606939 |
| 3 | 2.19E+08 | 2.19E+08 | 7 | 38398 | oar3_OAR3_218568542 | oar3_OAR3_218606939 |
| 3 | 2.19E+08 | 2.19E+08 | 28 | 111613 | oar3_OAR3_218866555 | oar3_OAR3_218978167 |
| 3 | 2.19E+08 | 2.19E+08 | 18 | 63059 | s18720.1 | oar3_OAR3_218978167 |
| 3 | 2.19E+08 | 2.19E+08 | 14 | 40517 | oar3_OAR3_218937651 | oar3_OAR3_218978167 |
| 3 | 2.19E+08 | 2.19E+08 | 11 | 30645 | oar3_OAR3_218947523 | oar3_OAR3_218978167 |
| 3 | 2.19E+08 | 2.19E+08 | 45 | 194117 | oar3_OAR3_219233096 | oar3_OAR3_219427212 |
| 3 | 2.19E+08 | 2.19E+08 | 7 | 10435 | oar3_OAR3_219259858 | oar3_OAR3_219270292 |
| 3 | 2.19E+08 | 2.19E+08 | 16 | 62618 | oar3_OAR3_219259858 | oar3_OAR3_219322475 |
| 3 | 2.19E+08 | 2.19E+08 | 12 | 52855 | oar3_OAR3_219259858 | oar3_OAR3_219312712 |
| 3 | 2.19E+08 | 2.19E+08 | 16 | 45209 | oar3_OAR3_219382004 | oar3_OAR3_219427212 |
| 3 | 2.19E+08 | 2.19E+08 | 13 | 24973 | oar3_OAR3_219402240 | oar3_OAR3_219427212 |
| 3 | 2.19E+08 | 2.19E+08 | 8 | 13391 | oar3_OAR3_219413822 | oar3_OAR3_219427212 |
| 3 | 2.2E+08 | 2.2E+08 | 9 | 29862 | oar3_OAR3_219670169 | oar3_OAR3_219700030 |
| 3 | 2.2E+08 | 2.2E+08 | 12 | 36246 | oar3_OAR3_219935830 | oar3_OAR3_219972075 |
| 3 | 2.2E+08 | 2.2E+08 | 12 | 36246 | oar3_OAR3_219935830 | oar3_OAR3_219972075 |
| 3 | 2.2E+08 | 2.2E+08 | 11 | 22334 | oar3_OAR3_219949742 | oar3_OAR3_219972075 |
| 3 | 2.2E+08 | 2.2E+08 | 11 | 22334 | oar3_OAR3_219949742 | oar3_OAR3_219972075 |
| 3 | 2.2E+08 | 2.2E+08 | 10 | 21763 | oar3_OAR3_219963549 | oar3_OAR3_219985311 |
| 3 | 2.2E+08 | 2.2E+08 | 6 | 4221 | oar3_OAR3_219963549 | oar3_OAR3_219967769 |
| 3 | 2.2E+08 | 2.2E+08 | 7 | 8527 | oar3_OAR3_219963549 | oar3_OAR3_219972075 |
| 3 | 2.2E+08 | 2.2E+08 | 6 | 10298 | oar3_OAR3_220212548 | oar3_OAR3_220222845 |
| 3 | 2.2E+08 | 2.2E+08 | 14 | 20539 | oar3_OAR3_220212548 | oar3_OAR3_220233086 |
| 3 | 2.2E+08 | 2.2E+08 | 45 | 195228 | oar3_OAR3_220219088 | oar3_OAR3_220414315 |
| 3 | 2.2E+08 | 2.2E+08 | 42 | 182072 | oar3_OAR3_220222322 | oar3_OAR3_220404393 |
| 3 | 2.2E+08 | 2.2E+08 | 41 | 178666 | oar3_OAR3_220222322 | oar3_OAR3_220400987 |
| 3 | 2.2E+08 | 2.2E+08 | 16 | 80856 | oar3_OAR3_220246530 | s50255.1 |
| 3 | 2.2E+08 | 2.2E+08 | 25 | 138496 | oar3_OAR3_220246530 | oar3_OAR3_220385025 |
| 3 | 2.2E+08 | 2.2E+08 | 30 | 155154 | oar3_OAR3_220249240 | oar3_OAR3_220404393 |
| 3 | 2.2E+08 | 2.2E+08 | 11 | 32922 | oar3_OAR3_220371472 | oar3_OAR3_220404393 |
| 3 | 2.2E+08 | 2.2E+08 | 8 | 21356 | oar3_OAR3_220383038 | oar3_OAR3_220404393 |
| 3 | 2.2E+08 | 2.21E+08 | 32 | 220763 | oar3_OAR3_220383038 | oar3_OAR3_220603800 |
| 3 | 2.2E+08 | 2.2E+08 | 5 | 12699 | oar3_OAR3_220391695 | oar3_OAR3_220404393 |
| 3 | 2.2E+08 | 2.2E+08 | 5 | 12699 | oar3_OAR3_220391695 | oar3_OAR3_220404393 |
| 3 | 2.21E+08 | 2.21E+08 | 15 | 50813 | oar3_OAR3_220518683 | oar3_OAR3_220569495 |
| 3 | 2.21E+08 | 2.21E+08 | 14 | 30905 | oar3_OAR3_220538591 | oar3_OAR3_220569495 |
| 3 | 2.21E+08 | 2.21E+08 | 20 | 65210 | oar3_OAR3_220538591 | oar3_OAR3_220603800 |
| 3 | 2.21E+08 | 2.21E+08 | 11 | 24609 | oar3_OAR3_220544887 | oar3_OAR3_220569495 |
| 3 | 2.21E+08 | 2.21E+08 | 6 | 2159 | oar3_OAR3_220567337 | oar3_OAR3_220569495 |
| 3 | 2.21E+08 | 2.21E+08 | 6 | 2159 | oar3_OAR3_220567337 | oar3_OAR3_220569495 |
| 3 | 2.21E+08 | 2.21E+08 | 15 | 44016 | oar3_OAR3_220567337 | oar3_OAR3_220611352 |
| 3 | 2.21E+08 | 2.21E+08 | 6 | 2159 | oar3_OAR3_220567337 | oar3_OAR3_220569495 |
| 3 | 2.21E+08 | 2.21E+08 | 4 | 490 | oar3_OAR3_220569006 | oar3_OAR3_220569495 |
| 3 | 2.21E+08 | 2.21E+08 | 10 | 73708 | oar3_OAR3_220656591 | oar3_OAR3_220730298 |
| 3 | 2.21E+08 | 2.21E+08 | 22 | 187568 | oar3_OAR3_220656591 | oar3_OAR3_220844158 |
| 3 | 2.21E+08 | 2.21E+08 | 25 | 214936 | oar3_OAR3_220656771 | oar3_OAR3_220871706 |
| 3 | 2.21E+08 | 2.21E+08 | 24 | 210460 | oar3_OAR3_220667058 | oar3_OAR3_220877517 |
| 3 | 2.21E+08 | 2.21E+08 | 19 | 153700 | oar3_OAR3_220704040 | oar3_OAR3_220857739 |
| 3 | 2.21E+08 | 2.21E+08 | 20 | 147570 | OAR3_239187680.1 | oar3_OAR3_220871706 |
| 3 | 2.21E+08 | 2.21E+08 | 20 | 147570 | OAR3_239187680.1 | oar3_OAR3_220871706 |
| 3 | 2.21E+08 | 2.21E+08 | 4 | 28407 | s04588.1 | oar3_OAR3_220844158 |
| 3 | 2.21E+08 | 2.21E+08 | 8 | 55955 | s04588.1 | oar3_OAR3_220871706 |
| 3 | 2.21E+08 | 2.21E+08 | 8 | 55955 | s04588.1 | oar3_OAR3_220871706 |
| 3 | 2.21E+08 | 2.21E+08 | 36 | 126574 | oar3_OAR3_220959316 | oar3_OAR3_221085889 |
| 3 | 2.21E+08 | 2.21E+08 | 36 | 126574 | oar3_OAR3_220959316 | oar3_OAR3_221085889 |
| 3 | 2.21E+08 | 2.21E+08 | 15 | 44461 | oar3_OAR3_220959316 | oar3_OAR3_221003776 |
| 3 | 2.21E+08 | 2.21E+08 | 10 | 21151 | oar3_OAR3_220982626 | oar3_OAR3_221003776 |
| 3 | 2.21E+08 | 2.21E+08 | 9 | 19587 | oar3_OAR3_221042696 | oar3_OAR3_221062282 |
| 3 | 2.21E+08 | 2.21E+08 | 12 | 36436 | oar3_OAR3_221049454 | oar3_OAR3_221085889 |
| 3 | 2.21E+08 | 2.21E+08 | 16 | 66491 | oar3_OAR3_221106811 | oar3_OAR3_221173301 |
| 3 | 2.21E+08 | 2.21E+08 | 15 | 60710 | oar3_OAR3_221112592 | oar3_OAR3_221173301 |
| 3 | 2.21E+08 | 2.21E+08 | 26 | 107357 | oar3_OAR3_221320984 | oar3_OAR3_221428340 |
| 3 | 2.21E+08 | 2.21E+08 | 26 | 107357 | oar3_OAR3_221320984 | oar3_OAR3_221428340 |
| 3 | 2.21E+08 | 2.21E+08 | 24 | 85643 | oar3_OAR3_221342698 | oar3_OAR3_221428340 |
| 3 | 2.21E+08 | 2.21E+08 | 20 | 68020 | oar3_OAR3_221351833 | oar3_OAR3_221419852 |
| 3 | 2.21E+08 | 2.21E+08 | 9 | 22655 | oar3_OAR3_221401047 | oar3_OAR3_221423701 |
| 3 | 2.21E+08 | 2.21E+08 | 7 | 13862 | oar3_OAR3_221414479 | oar3_OAR3_221428340 |
| 3 | 2.21E+08 | 2.21E+08 | 6 | 9223 | oar3_OAR3_221414479 | oar3_OAR3_221423701 |
| 3 | 2.22E+08 | 2.22E+08 | 6 | 12031 | oar3_OAR3_221769837 | oar3_OAR3_221781867 |
| 3 | 2.22E+08 | 2.22E+08 | 10 | 46191 | oar3_OAR3_222057711 | oar3_OAR3_222103901 |
| 3 | 2.22E+08 | 2.22E+08 | 9 | 28989 | oar3_OAR3_222152312 | oar3_OAR3_222181300 |
| 3 | 2.22E+08 | 2.22E+08 | 8 | 26575 | oar3_OAR3_222154726 | oar3_OAR3_222181300 |
| 3 | 2.22E+08 | 2.22E+08 | 4 | 9623 | oar3_OAR3_222171678 | oar3_OAR3_222181300 |
| 3 | 2.22E+08 | 2.22E+08 | 3 | 8017 | oar3_OAR3_222173284 | oar3_OAR3_222181300 |
| 3 | 2.22E+08 | 2.22E+08 | 3 | 8017 | oar3_OAR3_222173284 | oar3_OAR3_222181300 |
| 3 | 2.23E+08 | 2.23E+08 | 14 | 84333 | oar3_OAR3_222525469 | oar3_OAR3_222609801 |
| 3 | 2.23E+08 | 2.23E+08 | 22 | 116153 | oar3_OAR3_222525469 | oar3_OAR3_222641621 |
| 3 | 2.23E+08 | 2.23E+08 | 13 | 74604 | oar3_OAR3_222525469 | oar3_OAR3_222600072 |
| 3 | 2.23E+08 | 2.23E+08 | 9 | 48827 | oar3_OAR3_222525469 | oar3_OAR3_222574295 |
| 3 | 2.23E+08 | 2.23E+08 | 7 | 12387 | oar3_OAR3_222525469 | oar3_OAR3_222537855 |
| 3 | 2.23E+08 | 2.23E+08 | 41 | 251774 | oar3_OAR3_222525469 | oar3_OAR3_222777242 |
| 3 | 2.23E+08 | 2.23E+08 | 25 | 140830 | OAR3_241200714.1 | oar3_OAR3_222674211 |
| 3 | 2.23E+08 | 2.23E+08 | 14 | 88429 | oar3_OAR3_222536130 | oar3_OAR3_222624558 |
| 3 | 2.23E+08 | 2.23E+08 | 27 | 159752 | oar3_OAR3_222536130 | oar3_OAR3_222695881 |
| 3 | 2.23E+08 | 2.23E+08 | 6 | 23349 | oar3_OAR3_222658107 | oar3_OAR3_222681455 |
| 3 | 2.23E+08 | 2.23E+08 | 7 | 35780 | oar3_OAR3_222658107 | oar3_OAR3_222693886 |
| 3 | 2.23E+08 | 2.23E+08 | 15 | 89379 | oar3_OAR3_222658107 | s44809.1 |
| 3 | 2.23E+08 | 2.23E+08 | 14 | 70877 | oar3_OAR3_222741458 | oar3_OAR3_222812334 |
| 3 | 2.23E+08 | 2.23E+08 | 10 | 39857 | oar3_OAR3_222772478 | oar3_OAR3_222812334 |
| 3 | 2.23E+08 | 2.23E+08 | 12 | 34348 | oar3_OAR3_223148193 | oar3_OAR3_223182540 |
| 3 | 2.23E+08 | 2.23E+08 | 12 | 34348 | oar3_OAR3_223148193 | oar3_OAR3_223182540 |
| 3 | 2.23E+08 | 2.23E+08 | 11 | 27684 | oar3_OAR3_223148193 | oar3_OAR3_223175876 |
| 3 | 2.23E+08 | 2.23E+08 | 12 | 34348 | oar3_OAR3_223148193 | oar3_OAR3_223182540 |
| 3 | 2.23E+08 | 2.23E+08 | 11 | 32921 | s37869.1 | oar3_OAR3_223182540 |
| 3 | 2.23E+08 | 2.24E+08 | 38 | 224605 | oar3_OAR3_223347729 | oar3_OAR3_223572333 |
| 3 | 2.23E+08 | 2.23E+08 | 11 | 32524 | oar3_OAR3_223347729 | oar3_OAR3_223380252 |
| 3 | 2.23E+08 | 2.23E+08 | 15 | 53672 | oar3_OAR3_223350309 | oar3_OAR3_223403980 |
| 3 | 2.23E+08 | 2.24E+08 | 50 | 341033 | oar3_OAR3_223350309 | oar3_OAR3_223691341 |
| 3 | 2.23E+08 | 2.23E+08 | 17 | 102668 | oar3_OAR3_223378479 | oar3_OAR3_223481146 |
| 3 | 2.23E+08 | 2.23E+08 | 6 | 23729 | oar3_OAR3_223380252 | oar3_OAR3_223403980 |
| 3 | 2.23E+08 | 2.23E+08 | 15 | 96446 | oar3_OAR3_223384701 | oar3_OAR3_223481146 |
| 3 | 2.23E+08 | 2.23E+08 | 5 | 19280 | oar3_OAR3_223384701 | oar3_OAR3_223403980 |
| 3 | 2.23E+08 | 2.23E+08 | 10 | 56784 | oar3_OAR3_223424363 | oar3_OAR3_223481146 |
| 3 | 2.23E+08 | 2.24E+08 | 22 | 147971 | oar3_OAR3_223424363 | oar3_OAR3_223572333 |
| 3 | 2.23E+08 | 2.24E+08 | 18 | 131855 | oar3_OAR3_223440479 | oar3_OAR3_223572333 |
| 3 | 2.23E+08 | 2.24E+08 | 18 | 131855 | oar3_OAR3_223440479 | oar3_OAR3_223572333 |
| 3 | 2.23E+08 | 2.24E+08 | 7 | 65807 | oar3_OAR3_223466472 | oar3_OAR3_223532278 |
| 3 | 2.24E+08 | 2.24E+08 | 9 | 40056 | oar3_OAR3_223532278 | oar3_OAR3_223572333 |
| 3 | 2.24E+08 | 2.24E+08 | 26 | 188000 | oar3_OAR3_223532278 | oar3_OAR3_223720277 |
| 3 | 2.24E+08 | 2.24E+08 | 22 | 159064 | oar3_OAR3_223532278 | oar3_OAR3_223691341 |
| 3 | 2.24E+08 | 2.24E+08 | 10 | 74783 | oar3_OAR3_223532278 | oar3_OAR3_223607060 |
| 3 | 2.24E+08 | 2.24E+08 | 11 | 75773 | oar3_OAR3_223544473 | oar3_OAR3_223620245 |
| 3 | 2.24E+08 | 2.24E+08 | 13 | 82082 | oar3_OAR3_223638196 | oar3_OAR3_223720277 |
| 3 | 2.24E+08 | 2.24E+08 | 10 | 38800 | oar3_OAR3_223681478 | oar3_OAR3_223720277 |
| 3 | 2.24E+08 | 2.24E+08 | 6 | 9864 | oar3_OAR3_223681478 | oar3_OAR3_223691341 |
| 3 | 2.24E+08 | 2.24E+08 | 78 | 484914 | oar3_OAR3_223691341 | oar3_OAR3_224176254 |
| 3 | 2.24E+08 | 2.24E+08 | 22 | 117887 | oar3_OAR3_223787604 | oar3_OAR3_223905490 |
| 3 | 2.24E+08 | 2.24E+08 | 20 | 95997 | oar3_OAR3_223809494 | oar3_OAR3_223905490 |
| 3 | 2.24E+08 | 2.24E+08 | 20 | 95997 | oar3_OAR3_223809494 | oar3_OAR3_223905490 |
| 3 | 2.24E+08 | 2.24E+08 | 30 | 205037 | oar3_OAR3_223809494 | oar3_OAR3_224014530 |
| 3 | 2.24E+08 | 2.24E+08 | 10 | 46211 | oar3_OAR3_223809494 | s48755.1 |
| 3 | 2.24E+08 | 2.24E+08 | 13 | 54583 | oar3_OAR3_223850908 | oar3_OAR3_223905490 |
| 3 | 2.24E+08 | 2.24E+08 | 25 | 169641 | oar3_OAR3_223850908 | oar3_OAR3_224020548 |
| 3 | 2.24E+08 | 2.24E+08 | 13 | 54583 | oar3_OAR3_223850908 | oar3_OAR3_223905490 |
| 3 | 2.24E+08 | 2.24E+08 | 13 | 54583 | oar3_OAR3_223850908 | oar3_OAR3_223905490 |
| 3 | 2.24E+08 | 2.24E+08 | 13 | 54583 | oar3_OAR3_223850908 | oar3_OAR3_223905490 |
| 3 | 2.24E+08 | 2.24E+08 | 11 | 49787 | s48755.1 | oar3_OAR3_223905490 |
| 3 | 2.24E+08 | 2.24E+08 | 26 | 171861 | s48755.1 | oar3_OAR3_224027564 |
| 3 | 2.24E+08 | 2.24E+08 | 5 | 11787 | oar3_OAR3_223857407 | oar3_OAR3_223869193 |
| 3 | 2.24E+08 | 2.24E+08 | 17 | 140906 | oar3_OAR3_223879643 | oar3_OAR3_224020548 |
| 3 | 2.24E+08 | 2.24E+08 | 22 | 156258 | oar3_OAR3_223879643 | oar3_OAR3_224035900 |
| 3 | 2.24E+08 | 2.24E+08 | 3 | 11617 | oar3_OAR3_223893874 | oar3_OAR3_223905490 |
| 3 | 2.24E+08 | 2.24E+08 | 6 | 40619 | oar3_OAR3_223973912 | oar3_OAR3_224014530 |
| 3 | 2.24E+08 | 2.24E+08 | 10 | 39437 | oar3_OAR3_223996464 | oar3_OAR3_224035900 |
| 3 | 2.24E+08 | 2.24E+08 | 8 | 31101 | oar3_OAR3_223996464 | oar3_OAR3_224027564 |
| 3 | 2.24E+08 | 2.24E+08 | 5 | 24085 | oar3_OAR3_223996464 | oar3_OAR3_224020548 |
| 3 | 2.24E+08 | 2.24E+08 | 5 | 24085 | oar3_OAR3_223996464 | oar3_OAR3_224020548 |
| 3 | 2.24E+08 | 2.24E+08 | 13 | 57977 | oar3_OAR3_224131151 | oar3_OAR3_224189127 |
| 3 | 2.24E+08 | 2.24E+08 | 5 | 35002 | oar3_OAR3_224131151 | s35224.1 |
| 3 | 2.24E+08 | 2.24E+08 | 12 | 46821 | oar3_OAR3_224169535 | oar3_OAR3_224216355 |
| 3 | 2.24E+08 | 2.24E+08 | 5 | 6127 | oar3_OAR3_224170128 | oar3_OAR3_224176254 |
| 3 | 2.24E+08 | 2.24E+08 | 6 | 21108 | oar3_OAR3_224175493 | oar3_OAR3_224196600 |
| 3 | 2.24E+08 | 2.24E+08 | 7 | 27634 | oar3_OAR3_224175493 | oar3_OAR3_224203126 |
| 3 | 2.24E+08 | 2.24E+08 | 11 | 37725 | oar3_OAR3_224240480 | oar3_OAR3_224278204 |
| 4 | 49139 | 93370 | 8 | 44232 | oar3_OAR4_49139 | oar3_OAR4_93370 |
| 4 | 4826260 | 4830192 | 10 | 3933 | oar3_OAR4_4826260 | oar3_OAR4_4830192 |
| 4 | 5079824 | 5087463 | 5 | 7640 | s56283.1 | oar3_OAR4_5087463 |
| 4 | 10335965 | 10338973 | 3 | 3009 | oar3_OAR4_10335965 | oar3_OAR4_10338973 |
| 4 | 19162064 | 19186857 | 6 | 24794 | OAR4_19586970_X.1 | oar3_OAR4_19186857 |
| 4 | 20530157 | 20549104 | 6 | 18948 | oar3_OAR4_20530157 | oar3_OAR4_20549104 |
| 4 | 32535992 | 32651470 | 13 | 115479 | oar3_OAR4_32535992 | oar3_OAR4_32651470 |
| 4 | 32734650 | 32808431 | 8 | 73782 | oar3_OAR4_32734650 | oar3_OAR4_32808431 |
| 4 | 32851694 | 32933344 | 9 | 81651 | oar3_OAR4_32851694 | oar3_OAR4_32933344 |
| 4 | 32851694 | 32933344 | 9 | 81651 | oar3_OAR4_32851694 | oar3_OAR4_32933344 |
| 4 | 34042056 | 34081825 | 9 | 39770 | oar3_OAR4_34042056 | oar3_OAR4_34081825 |
| 4 | 41599767 | 41632016 | 6 | 32250 | oar3_OAR4_41599767 | oar3_OAR4_41632016 |
| 4 | 41599767 | 41632016 | 6 | 32250 | oar3_OAR4_41599767 | oar3_OAR4_41632016 |
| 4 | 42683287 | 42702642 | 5 | 19356 | oar3_OAR4_42683287 | oar3_OAR4_42702642 |
| 4 | 50091386 | 50099862 | 4 | 8477 | OAR4_52906204.1 | oar3_OAR4_50099862 |
| 4 | 54535843 | 54536713 | 3 | 871 | oar3_OAR4_54535843 | oar3_OAR4_54536713 |
| 4 | 65402342 | 65407945 | 6 | 5604 | oar3_OAR4_65402342 | oar3_OAR4_65407945 |
| 4 | 74306738 | 74338543 | 8 | 31806 | oar3_OAR4_74306738 | oar3_OAR4_74338543 |
| 4 | 74948644 | 74963295 | 9 | 14652 | oar3_OAR4_74948644 | oar3_OAR4_74963295 |
| 4 | 76593159 | 76616578 | 7 | 23420 | oar3_OAR4_76593159 | oar3_OAR4_76616578 |
| 4 | 76593159 | 76616578 | 7 | 23420 | oar3_OAR4_76593159 | oar3_OAR4_76616578 |
| 4 | 76798315 | 76809327 | 8 | 11013 | oar3_OAR4_76798315 | oar3_OAR4_76809327 |
| 4 | 76802322 | 76809327 | 5 | 7006 | oar3_OAR4_76802322 | oar3_OAR4_76809327 |
| 4 | 77070692 | 77149943 | 23 | 79252 | s74434.1 | oar3_OAR4_77149943 |
| 4 | 77075306 | 77149943 | 22 | 74638 | oar3_OAR4_77075306 | oar3_OAR4_77149943 |
| 4 | 77075306 | 77149943 | 22 | 74638 | oar3_OAR4_77075306 | oar3_OAR4_77149943 |
| 4 | 77075306 | 77169441 | 27 | 94136 | oar3_OAR4_77075306 | oar3_OAR4_77169441 |
| 4 | 77084520 | 77149943 | 21 | 65424 | oar3_OAR4_77084520 | oar3_OAR4_77149943 |
| 4 | 77243180 | 77278244 | 10 | 35065 | oar3_OAR4_77243180 | oar3_OAR4_77278244 |
| 4 | 79119725 | 79131133 | 7 | 11409 | oar3_OAR4_79119725 | oar3_OAR4_79131133 |
| 4 | 92351764 | 92370227 | 9 | 18464 | oar3_OAR4_92351764 | oar3_OAR4_92370227 |
| 4 | 92351764 | 92390899 | 13 | 39136 | oar3_OAR4_92351764 | oar3_OAR4_92390899 |
| 4 | 92616221 | 92678741 | 14 | 62521 | oar3_OAR4_92616221 | oar3_OAR4_92678741 |
| 4 | 92645843 | 92719868 | 13 | 74026 | oar3_OAR4_92645843 | oar3_OAR4_92719868 |
| 4 | 92817945 | 92921145 | 18 | 103201 | oar3_OAR4_92817945 | oar3_OAR4_92921145 |
| 4 | 92862327 | 92965345 | 22 | 103019 | oar3_OAR4_92862327 | oar3_OAR4_92965345 |
| 4 | 92862327 | 92965345 | 22 | 103019 | oar3_OAR4_92862327 | oar3_OAR4_92965345 |
| 4 | 92862327 | 92965345 | 22 | 103019 | oar3_OAR4_92862327 | oar3_OAR4_92965345 |
| 4 | 92862327 | 92965345 | 22 | 103019 | oar3_OAR4_92862327 | oar3_OAR4_92965345 |
| 4 | 92862327 | 92965345 | 22 | 103019 | oar3_OAR4_92862327 | oar3_OAR4_92965345 |
| 4 | 95835182 | 95860348 | 8 | 25167 | oar3_OAR4_95835182 | oar3_OAR4_95860348 |
| 4 | 95835182 | 95845960 | 6 | 10779 | oar3_OAR4_95835182 | oar3_OAR4_95845960 |
| 4 | 95845285 | 95874047 | 7 | 28763 | oar3_OAR4_95845285 | oar3_OAR4_95874047 |
| 4 | 98776794 | 98866421 | 27 | 89628 | oar3_OAR4_98776794 | oar3_OAR4_98866421 |
| 4 | 1.11E+08 | 1.11E+08 | 8 | 36976 | oar3_OAR4_111351494 | oar3_OAR4_111388469 |
| 4 | 1.12E+08 | 1.12E+08 | 5 | 13677 | oar3_OAR4_111790133 | oar3_OAR4_111803809 |
| 4 | 1.12E+08 | 1.12E+08 | 5 | 13677 | oar3_OAR4_111790133 | oar3_OAR4_111803809 |
| 4 | 1.12E+08 | 1.12E+08 | 5 | 13677 | oar3_OAR4_111790133 | oar3_OAR4_111803809 |
| 4 | 1.12E+08 | 1.12E+08 | 5 | 13677 | oar3_OAR4_111790133 | oar3_OAR4_111803809 |
| 4 | 1.12E+08 | 1.12E+08 | 5 | 13677 | oar3_OAR4_111790133 | oar3_OAR4_111803809 |
| 4 | 1.12E+08 | 1.12E+08 | 64 | 220085 | oar3_OAR4_111934724 | oar3_OAR4_112154808 |
| 4 | 1.12E+08 | 1.12E+08 | 3 | 13394 | oar3_OAR4_111934724 | oar3_OAR4_111948117 |
| 4 | 1.12E+08 | 1.12E+08 | 33 | 101517 | oar3_OAR4_111941134 | oar3_OAR4_112042650 |
| 4 | 1.12E+08 | 1.12E+08 | 23 | 57371 | s00918.1 | oar3_OAR4_112046617 |
| 4 | 1.12E+08 | 1.12E+08 | 10 | 10793 | oar3_OAR4_111998812 | oar3_OAR4_112009604 |
| 4 | 1.12E+08 | 1.12E+08 | 22 | 47806 | oar3_OAR4_111998812 | oar3_OAR4_112046617 |
| 4 | 1.12E+08 | 1.12E+08 | 18 | 38332 | oar3_OAR4_111998812 | oar3_OAR4_112037143 |
| 4 | 1.12E+08 | 1.12E+08 | 10 | 10793 | oar3_OAR4_111998812 | oar3_OAR4_112009604 |
| 4 | 1.12E+08 | 1.12E+08 | 15 | 37719 | oar3_OAR4_111999425 | oar3_OAR4_112037143 |
| 4 | 1.12E+08 | 1.12E+08 | 19 | 47193 | oar3_OAR4_111999425 | oar3_OAR4_112046617 |
| 4 | 1.12E+08 | 1.12E+08 | 17 | 43069 | oar3_OAR4_112003549 | oar3_OAR4_112046617 |
| 4 | 1.12E+08 | 1.12E+08 | 12 | 27676 | oar3_OAR4_112018942 | oar3_OAR4_112046617 |
| 4 | 1.12E+08 | 1.12E+08 | 14 | 24714 | oar3_OAR4_112080350 | oar3_OAR4_112105063 |
| 4 | 1.12E+08 | 1.12E+08 | 10 | 45337 | oar3_OAR4_112119037 | oar3_OAR4_112164373 |
| 4 | 1.12E+08 | 1.12E+08 | 7 | 34111 | oar3_OAR4_112152355 | oar3_OAR4_112186465 |
| 4 | 1.12E+08 | 1.12E+08 | 3 | 11201 | oar3_OAR4_112240314 | oar3_OAR4_112251514 |
| 4 | 1.12E+08 | 1.12E+08 | 12 | 103776 | oar3_OAR4_112240314 | oar3_OAR4_112344089 |
| 4 | 1.12E+08 | 1.12E+08 | 6 | 44708 | oar3_OAR4_112240314 | oar3_OAR4_112285021 |
| 4 | 1.12E+08 | 1.12E+08 | 6 | 44708 | oar3_OAR4_112240314 | oar3_OAR4_112285021 |
| 4 | 1.12E+08 | 1.12E+08 | 3 | 11201 | oar3_OAR4_112240314 | oar3_OAR4_112251514 |
| 4 | 1.12E+08 | 1.12E+08 | 8 | 64539 | oar3_OAR4_112240314 | oar3_OAR4_112304852 |
| 4 | 1.12E+08 | 1.12E+08 | 6 | 44708 | oar3_OAR4_112240314 | oar3_OAR4_112285021 |
| 4 | 1.12E+08 | 1.12E+08 | 5 | 39610 | oar3_OAR4_112240314 | oar3_OAR4_112279923 |
| 4 | 1.12E+08 | 1.12E+08 | 15 | 147466 | oar3_OAR4_112240314 | oar3_OAR4_112387779 |
| 4 | 1.12E+08 | 1.12E+08 | 15 | 147466 | oar3_OAR4_112240314 | oar3_OAR4_112387779 |
| 4 | 1.12E+08 | 1.12E+08 | 5 | 110340 | oar3_OAR4_112387201 | oar3_OAR4_112497540 |
| 4 | 1.12E+08 | 1.12E+08 | 5 | 110340 | oar3_OAR4_112387201 | oar3_OAR4_112497540 |
| 4 | 1.12E+08 | 1.13E+08 | 4 | 96980 | oar3_OAR4_112497540 | oar3_OAR4_112594519 |
| 4 | 1.12E+08 | 1.13E+08 | 4 | 96980 | oar3_OAR4_112497540 | oar3_OAR4_112594519 |
| 4 | 1.13E+08 | 1.13E+08 | 7 | 55631 | oar3_OAR4_112936909 | OAR4_120520710.1 |
| 4 | 1.13E+08 | 1.13E+08 | 13 | 112943 | oar3_OAR4_112936909 | oar3_OAR4_113049851 |
| 4 | 1.13E+08 | 1.13E+08 | 21 | 157705 | oar3_OAR4_112936909 | oar3_OAR4_113094613 |
| 4 | 1.13E+08 | 1.13E+08 | 21 | 121034 | oar3_OAR4_113033573 | oar3_OAR4_113154606 |
| 4 | 1.13E+08 | 1.13E+08 | 6 | 19626 | oar3_OAR4_113203302 | oar3_OAR4_113222927 |
| 4 | 1.13E+08 | 1.13E+08 | 41 | 194424 | oar3_OAR4_113203302 | oar3_OAR4_113397725 |
| 4 | 1.13E+08 | 1.13E+08 | 6 | 19626 | oar3_OAR4_113203302 | oar3_OAR4_113222927 |
| 4 | 1.15E+08 | 1.15E+08 | 9 | 19567 | oar3_OAR4_114590116 | oar3_OAR4_114609682 |
| 4 | 1.16E+08 | 1.16E+08 | 5 | 5945 | oar3_OAR4_116249978 | oar3_OAR4_116255922 |
| 4 | 1.16E+08 | 1.16E+08 | 31 | 113908 | oar3_OAR4_116249978 | oar3_OAR4_116363885 |
| 4 | 1.16E+08 | 1.16E+08 | 4 | 3161 | oar3_OAR4_116249978 | oar3_OAR4_116253138 |
| 4 | 1.16E+08 | 1.16E+08 | 30 | 111498 | oar3_OAR4_116252388 | oar3_OAR4_116363885 |
| 4 | 1.16E+08 | 1.16E+08 | 12 | 36494 | oar3_OAR4_116311772 | OAR4_124183666.1 |
| 4 | 1.17E+08 | 1.17E+08 | 14 | 53533 | oar3_OAR4_116631255 | oar3_OAR4_116684787 |
| 4 | 1.17E+08 | 1.17E+08 | 25 | 104746 | oar3_OAR4_116631255 | oar3_OAR4_116736000 |
| 4 | 1.17E+08 | 1.17E+08 | 9 | 22747 | oar3_OAR4_116643386 | oar3_OAR4_116666132 |
| 4 | 1.17E+08 | 1.17E+08 | 23 | 92615 | oar3_OAR4_116643386 | oar3_OAR4_116736000 |
| 4 | 1.17E+08 | 1.17E+08 | 8 | 39559 | oar3_OAR4_116666132 | oar3_OAR4_116705690 |
| 4 | 1.17E+08 | 1.17E+08 | 13 | 55962 | oar3_OAR4_116680039 | oar3_OAR4_116736000 |
| 4 | 1.17E+08 | 1.17E+08 | 11 | 44707 | oar3_OAR4_116872341 | oar3_OAR4_116917047 |
| 4 | 1.17E+08 | 1.17E+08 | 16 | 74389 | oar3_OAR4_116948710 | oar3_OAR4_117023098 |
| 4 | 1.17E+08 | 1.17E+08 | 5 | 15110 | oar3_OAR4_117255845 | oar3_OAR4_117270954 |
| 4 | 1.17E+08 | 1.17E+08 | 8 | 40222 | oar3_OAR4_117349450 | oar3_OAR4_117389671 |
| 4 | 1.18E+08 | 1.18E+08 | 22 | 92957 | oar3_OAR4_117549609 | oar3_OAR4_117642565 |
| 4 | 1.18E+08 | 1.18E+08 | 9 | 39166 | oar3_OAR4_117698915 | oar3_OAR4_117738080 |
| 4 | 1.18E+08 | 1.18E+08 | 12 | 20389 | oar3_OAR4_117753115 | oar3_OAR4_117773503 |
| 4 | 1.18E+08 | 1.18E+08 | 10 | 13785 | oar3_OAR4_117759719 | oar3_OAR4_117773503 |
| 4 | 1.18E+08 | 1.18E+08 | 6 | 7991 | oar3_OAR4_117765513 | oar3_OAR4_117773503 |
| 4 | 1.18E+08 | 1.18E+08 | 9 | 43878 | s37914.1 | oar3_OAR4_117811035 |
| 4 | 1.18E+08 | 1.18E+08 | 54 | 268489 | oar3_OAR4_117906511 | oar3_OAR4_118174999 |
| 4 | 1.18E+08 | 1.18E+08 | 14 | 73631 | oar3_OAR4_117914951 | oar3_OAR4_117988581 |
| 4 | 1.18E+08 | 1.18E+08 | 13 | 55609 | oar3_OAR4_118094847 | oar3_OAR4_118150455 |
| 4 | 1.18E+08 | 1.18E+08 | 26 | 102715 | oar3_OAR4_118230272 | oar3_OAR4_118332986 |
| 4 | 1.18E+08 | 1.18E+08 | 5 | 28849 | oar3_OAR4_118260539 | s47661.1 |
| 4 | 1.18E+08 | 1.18E+08 | 28 | 104285 | oar3_OAR4_118307775 | oar3_OAR4_118412059 |
| 4 | 1.18E+08 | 1.18E+08 | 45 | 190707 | oar3_OAR4_118307775 | oar3_OAR4_118498481 |
| 4 | 1.18E+08 | 1.18E+08 | 18 | 69547 | oar3_OAR4_118328374 | s39821.1 |
| 4 | 1.18E+08 | 1.18E+08 | 8 | 28885 | oar3_OAR4_118383175 | oar3_OAR4_118412059 |
| 4 | 1.18E+08 | 1.18E+08 | 3 | 352 | oar3_OAR4_118411708 | oar3_OAR4_118412059 |
| 4 | 1.18E+08 | 1.18E+08 | 11 | 32030 | oar3_OAR4_118466452 | oar3_OAR4_118498481 |
| 4 | 1.18E+08 | 1.19E+08 | 14 | 42836 | oar3_OAR4_118466452 | oar3_OAR4_118509287 |
| 4 | 1.18E+08 | 1.18E+08 | 10 | 25529 | s67969.1 | oar3_OAR4_118498481 |
| 4 | 1.19E+08 | 1.19E+08 | 8 | 23179 | oar3_OAR4_118538048 | oar3_OAR4_118561226 |
| 4 | 1.19E+08 | 1.19E+08 | 10 | 34256 | oar3_OAR4_118549452 | oar3_OAR4_118583707 |
| 4 | 1.19E+08 | 1.19E+08 | 21 | 132422 | oar3_OAR4_118578104 | oar3_OAR4_118710525 |
| 4 | 1.19E+08 | 1.19E+08 | 18 | 115879 | oar3_OAR4_118583707 | oar3_OAR4_118699585 |
| 4 | 1.19E+08 | 1.19E+08 | 8 | 40739 | oar3_OAR4_118658847 | oar3_OAR4_118699585 |
| 4 | 1.19E+08 | 1.19E+08 | 28 | 134547 | oar3_OAR4_118683955 | oar3_OAR4_118818501 |
| 4 | 1.19E+08 | 1.19E+08 | 28 | 149472 | oar3_OAR4_118724017 | oar3_OAR4_118873488 |
| 4 | 1.19E+08 | 1.19E+08 | 33 | 147090 | oar3_OAR4_118835508 | oar3_OAR4_118982597 |
| 4 | 1.19E+08 | 1.19E+08 | 20 | 60383 | oar3_OAR4_118922215 | oar3_OAR4_118982597 |
| 4 | 1.19E+08 | 1.19E+08 | 20 | 60383 | oar3_OAR4_118922215 | oar3_OAR4_118982597 |
| 4 | 1.19E+08 | 1.19E+08 | 12 | 39771 | oar3_OAR4_118942827 | oar3_OAR4_118982597 |
| 4 | 1.19E+08 | 1.19E+08 | 11 | 33942 | oar3_OAR4_118948656 | oar3_OAR4_118982597 |
| 4 | 1.19E+08 | 1.19E+08 | 13 | 20745 | oar3_OAR4_118966550 | s68340.1 |
| 4 | 1.19E+08 | 1.19E+08 | 19 | 58190 | oar3_OAR4_119044265 | OAR4_127053991.1 |
| 4 | 1.19E+08 | 1.19E+08 | 14 | 38971 | oar3_OAR4_119063484 | OAR4_127053991.1 |
| 4 | 1.19E+08 | 1.19E+08 | 14 | 38971 | oar3_OAR4_119063484 | OAR4_127053991.1 |
| 4 | 1.19E+08 | 1.19E+08 | 10 | 31042 | oar3_OAR4_119071413 | OAR4_127053991.1 |
| 4 | 1.19E+08 | 1.19E+08 | 5 | 4671 | oar3_OAR4_119097784 | OAR4_127053991.1 |
| 5 | 279153 | 285474 | 4 | 6322 | oar3_OAR5_279153 | oar3_OAR5_285474 |
| 5 | 480492 | 507091 | 3 | 26600 | oar3_OAR5_480492 | oar3_OAR5_507091 |
| 5 | 576704 | 594272 | 6 | 17569 | oar3_OAR5_576704 | oar3_OAR5_594272 |
| 5 | 1062225 | 1082247 | 7 | 20023 | oar3_OAR5_1062225 | oar3_OAR5_1082247 |
| 5 | 1062225 | 1082247 | 7 | 20023 | oar3_OAR5_1062225 | oar3_OAR5_1082247 |
| 5 | 1062225 | 1082247 | 7 | 20023 | oar3_OAR5_1062225 | oar3_OAR5_1082247 |
| 5 | 1594885 | 1612977 | 6 | 18093 | oar3_OAR5_1594885 | oar3_OAR5_1612977 |
| 5 | 1609483 | 1612977 | 3 | 3495 | oar3_OAR5_1609483 | oar3_OAR5_1612977 |
| 5 | 2363181 | 2422596 | 22 | 59416 | oar3_OAR5_2363181 | oar3_OAR5_2422596 |
| 5 | 2363181 | 2425534 | 24 | 62354 | oar3_OAR5_2363181 | oar3_OAR5_2425534 |
| 5 | 2363181 | 2425534 | 24 | 62354 | oar3_OAR5_2363181 | oar3_OAR5_2425534 |
| 5 | 2392592 | 2447481 | 19 | 54890 | oar3_OAR5_2392592 | oar3_OAR5_2447481 |
| 5 | 2401444 | 2431253 | 12 | 29810 | s12772.1 | oar3_OAR5_2431253 |
| 5 | 2411941 | 2425534 | 7 | 13594 | oar3_OAR5_2411941 | oar3_OAR5_2425534 |
| 5 | 2586793 | 2593942 | 6 | 7150 | oar3_OAR5_2586793 | oar3_OAR5_2593942 |
| 5 | 2652411 | 2679070 | 11 | 26660 | oar3_OAR5_2652411 | oar3_OAR5_2679070 |
| 5 | 2659971 | 2693540 | 11 | 33570 | oar3_OAR5_2659971 | oar3_OAR5_2693540 |
| 5 | 2659971 | 2693540 | 11 | 33570 | oar3_OAR5_2659971 | oar3_OAR5_2693540 |
| 5 | 2665122 | 2687360 | 8 | 22239 | oar3_OAR5_2665122 | oar3_OAR5_2687360 |
| 5 | 2665122 | 2701680 | 11 | 36559 | oar3_OAR5_2665122 | oar3_OAR5_2701680 |
| 5 | 3428208 | 3448064 | 8 | 19857 | oar3_OAR5_3428208 | oar3_OAR5_3448064 |
| 5 | 3435700 | 3448064 | 5 | 12365 | oar3_OAR5_3435700 | oar3_OAR5_3448064 |
| 5 | 3443064 | 3475920 | 9 | 32857 | oar3_OAR5_3443064 | oar3_OAR5_3475920 |
| 5 | 3575161 | 3617609 | 16 | 42449 | oar3_OAR5_3575161 | oar3_OAR5_3617609 |
| 5 | 3735431 | 3796188 | 15 | 60758 | oar3_OAR5_3735431 | s22426.1 |
| 5 | 3917674 | 4024241 | 26 | 106568 | oar3_OAR5_3917674 | oar3_OAR5_4024241 |
| 5 | 3917674 | 3948248 | 12 | 30575 | oar3_OAR5_3917674 | oar3_OAR5_3948248 |
| 5 | 3938546 | 3948248 | 6 | 9703 | oar3_OAR5_3938546 | oar3_OAR5_3948248 |
| 5 | 3938546 | 3948248 | 6 | 9703 | oar3_OAR5_3938546 | oar3_OAR5_3948248 |
| 5 | 4048214 | 4117267 | 24 | 69054 | oar3_OAR5_4048214 | oar3_OAR5_4117267 |
| 5 | 4052916 | 4126191 | 25 | 73276 | oar3_OAR5_4052916 | oar3_OAR5_4126191 |
| 5 | 4052916 | 4117267 | 23 | 64352 | oar3_OAR5_4052916 | oar3_OAR5_4117267 |
| 5 | 4069022 | 4117267 | 15 | 48246 | oar3_OAR5_4069022 | oar3_OAR5_4117267 |
| 5 | 4069022 | 4677530 | 116 | 608509 | oar3_OAR5_4069022 | oar3_OAR5_4677530 |
| 5 | 4069022 | 4117267 | 15 | 48246 | oar3_OAR5_4069022 | oar3_OAR5_4117267 |
| 5 | 4074511 | 4117267 | 14 | 42757 | oar3_OAR5_4074511 | oar3_OAR5_4117267 |
| 5 | 4074511 | 4117267 | 14 | 42757 | oar3_OAR5_4074511 | oar3_OAR5_4117267 |
| 5 | 4074511 | 4113559 | 13 | 39049 | oar3_OAR5_4074511 | oar3_OAR5_4113559 |
| 5 | 4074511 | 4117267 | 14 | 42757 | oar3_OAR5_4074511 | oar3_OAR5_4117267 |
| 5 | 4074511 | 4117267 | 14 | 42757 | oar3_OAR5_4074511 | oar3_OAR5_4117267 |
| 5 | 4080295 | 4117267 | 11 | 36973 | oar3_OAR5_4080295 | oar3_OAR5_4117267 |
| 5 | 4188542 | 4214958 | 7 | 26417 | oar3_OAR5_4188542 | oar3_OAR5_4214958 |
| 5 | 4351961 | 4371089 | 5 | 19129 | oar3_OAR5_4351961 | oar3_OAR5_4371089 |
| 5 | 4351961 | 4365537 | 4 | 13577 | oar3_OAR5_4351961 | s51932.1 |
| 5 | 4351961 | 4365537 | 4 | 13577 | oar3_OAR5_4351961 | s51932.1 |
| 5 | 4351961 | 4396830 | 13 | 44870 | oar3_OAR5_4351961 | oar3_OAR5_4396830 |
| 5 | 4351961 | 4371089 | 5 | 19129 | oar3_OAR5_4351961 | oar3_OAR5_4371089 |
| 5 | 4362814 | 4371089 | 4 | 8276 | oar3_OAR5_4362814 | oar3_OAR5_4371089 |
| 5 | 4487167 | 4523066 | 10 | 35900 | s58545.1 | oar3_OAR5_4523066 |
| 5 | 4502075 | 4517957 | 4 | 15883 | s03215.1 | oar3_OAR5_4517957 |
| 5 | 4585973 | 4677530 | 13 | 91558 | oar3_OAR5_4585973 | oar3_OAR5_4677530 |
| 5 | 4784099 | 4809749 | 11 | 25651 | oar3_OAR5_4784099 | oar3_OAR5_4809749 |
| 5 | 4992314 | 5004620 | 6 | 12307 | oar3_OAR5_4992314 | oar3_OAR5_5004620 |
| 5 | 5105077 | 5110575 | 5 | 5499 | oar3_OAR5_5105077 | oar3_OAR5_5110575 |
| 5 | 5246505 | 5356797 | 25 | 110293 | oar3_OAR5_5246505 | oar3_OAR5_5356797 |
| 5 | 5276990 | 5345245 | 18 | 68256 | oar3_OAR5_5276990 | oar3_OAR5_5345245 |
| 5 | 5492372 | 5575635 | 23 | 83264 | oar3_OAR5_5492372 | oar3_OAR5_5575635 |
| 5 | 5530736 | 5638078 | 36 | 107343 | oar3_OAR5_5530736 | oar3_OAR5_5638078 |
| 5 | 5607107 | 5637543 | 10 | 30437 | oar3_OAR5_5607107 | oar3_OAR5_5637543 |
| 5 | 5614494 | 5638078 | 8 | 23585 | oar3_OAR5_5614494 | oar3_OAR5_5638078 |
| 5 | 5903988 | 5927709 | 7 | 23722 | oar3_OAR5_5903988 | oar3_OAR5_5927709 |
| 5 | 5919755 | 5927709 | 5 | 7955 | oar3_OAR5_5919755 | oar3_OAR5_5927709 |
| 5 | 5919755 | 5927709 | 5 | 7955 | oar3_OAR5_5919755 | oar3_OAR5_5927709 |
| 5 | 5919755 | 5927709 | 5 | 7955 | oar3_OAR5_5919755 | oar3_OAR5_5927709 |
| 5 | 5919755 | 5927709 | 5 | 7955 | oar3_OAR5_5919755 | oar3_OAR5_5927709 |
| 5 | 6212614 | 6239117 | 6 | 26504 | oar3_OAR5_6212614 | oar3_OAR5_6239117 |
| 5 | 6216668 | 6253355 | 9 | 36688 | oar3_OAR5_6216668 | oar3_OAR5_6253355 |
| 5 | 6230237 | 6239117 | 4 | 8881 | oar3_OAR5_6230237 | oar3_OAR5_6239117 |
| 5 | 6230237 | 6253355 | 8 | 23119 | oar3_OAR5_6230237 | oar3_OAR5_6253355 |
| 5 | 6514224 | 6545153 | 8 | 30930 | oar3_OAR5_6514224 | oar3_OAR5_6545153 |
| 5 | 6514224 | 6545153 | 8 | 30930 | oar3_OAR5_6514224 | oar3_OAR5_6545153 |
| 5 | 6514224 | 6545153 | 8 | 30930 | oar3_OAR5_6514224 | oar3_OAR5_6545153 |
| 5 | 6514224 | 6568433 | 13 | 54210 | oar3_OAR5_6514224 | oar3_OAR5_6568433 |
| 5 | 7473864 | 7522436 | 13 | 48573 | oar3_OAR5_7473864 | oar3_OAR5_7522436 |
| 5 | 7473864 | 7508434 | 10 | 34571 | oar3_OAR5_7473864 | oar3_OAR5_7508434 |
| 5 | 7473864 | 7508434 | 10 | 34571 | oar3_OAR5_7473864 | oar3_OAR5_7508434 |
| 5 | 7476244 | 7508434 | 9 | 32191 | s29940.1 | oar3_OAR5_7508434 |
| 5 | 8517493 | 8591881 | 13 | 74389 | oar3_OAR5_8517493 | oar3_OAR5_8591881 |
| 5 | 9054122 | 9099375 | 10 | 45254 | oar3_OAR5_9054122 | oar3_OAR5_9099375 |
| 5 | 9054122 | 9099375 | 10 | 45254 | oar3_OAR5_9054122 | oar3_OAR5_9099375 |
| 5 | 9054122 | 9099375 | 10 | 45254 | oar3_OAR5_9054122 | oar3_OAR5_9099375 |
| 5 | 9327513 | 9423404 | 22 | 95892 | oar3_OAR5_9327513 | oar3_OAR5_9423404 |
| 5 | 9365622 | 9423404 | 14 | 57783 | oar3_OAR5_9365622 | oar3_OAR5_9423404 |
| 5 | 9380817 | 9410148 | 8 | 29332 | oar3_OAR5_9380817 | oar3_OAR5_9410148 |
| 5 | 9452504 | 9491441 | 15 | 38938 | oar3_OAR5_9452504 | OAR5_10884558.1 |
| 5 | 9603655 | 9718493 | 28 | 114839 | oar3_OAR5_9603655 | oar3_OAR5_9718493 |
| 5 | 9603655 | 9664233 | 20 | 60579 | oar3_OAR5_9603655 | oar3_OAR5_9664233 |
| 5 | 9603655 | 9640371 | 7 | 36717 | oar3_OAR5_9603655 | s37424.1 |
| 5 | 9715072 | 9739341 | 8 | 24270 | oar3_OAR5_9715072 | oar3_OAR5_9739341 |
| 5 | 9805894 | 9825255 | 5 | 19362 | oar3_OAR5_9805894 | oar3_OAR5_9825255 |
| 5 | 9856618 | 9868828 | 8 | 12211 | oar3_OAR5_9856618 | oar3_OAR5_9868828 |
| 5 | 9858609 | 9876849 | 8 | 18241 | oar3_OAR5_9858609 | oar3_OAR5_9876849 |
| 5 | 9862769 | 9983158 | 47 | 120390 | oar3_OAR5_9862769 | oar3_OAR5_9983158 |
| 5 | 9862769 | 9876849 | 7 | 14081 | oar3_OAR5_9862769 | oar3_OAR5_9876849 |
| 5 | 9862769 | 9876849 | 7 | 14081 | oar3_OAR5_9862769 | oar3_OAR5_9876849 |
| 5 | 9892589 | 9898574 | 6 | 5986 | oar3_OAR5_9892589 | oar3_OAR5_9898574 |
| 5 | 9892589 | 9898574 | 6 | 5986 | oar3_OAR5_9892589 | oar3_OAR5_9898574 |
| 5 | 9892589 | 9898574 | 6 | 5986 | oar3_OAR5_9892589 | oar3_OAR5_9898574 |
| 5 | 9892589 | 9905872 | 13 | 13284 | oar3_OAR5_9892589 | oar3_OAR5_9905872 |
| 5 | 9892589 | 9898574 | 6 | 5986 | oar3_OAR5_9892589 | oar3_OAR5_9898574 |
| 5 | 9892589 | 9905872 | 13 | 13284 | oar3_OAR5_9892589 | oar3_OAR5_9905872 |
| 5 | 9905653 | 9983158 | 25 | 77506 | oar3_OAR5_9905653 | oar3_OAR5_9983158 |
| 5 | 9938933 | 9983158 | 20 | 44226 | oar3_OAR5_9938933 | oar3_OAR5_9983158 |
| 5 | 9951421 | 9958993 | 7 | 7573 | oar3_OAR5_9951421 | oar3_OAR5_9958993 |
| 5 | 9951421 | 9983158 | 18 | 31738 | oar3_OAR5_9951421 | oar3_OAR5_9983158 |
| 5 | 11780139 | 11793513 | 3 | 13375 | oar3_OAR5_11780139 | oar3_OAR5_11793513 |
| 5 | 11780139 | 11793513 | 3 | 13375 | oar3_OAR5_11780139 | oar3_OAR5_11793513 |
| 5 | 11780139 | 11793513 | 3 | 13375 | oar3_OAR5_11780139 | oar3_OAR5_11793513 |
| 5 | 12359796 | 12379946 | 8 | 20151 | oar3_OAR5_12359796 | oar3_OAR5_12379946 |
| 5 | 12372302 | 12384942 | 5 | 12641 | s14924.1 | oar3_OAR5_12384942 |
| 5 | 12505631 | 12522895 | 7 | 17265 | oar3_OAR5_12505631 | oar3_OAR5_12522895 |
| 5 | 12505631 | 12579430 | 17 | 73800 | oar3_OAR5_12505631 | oar3_OAR5_12579430 |
| 5 | 12505631 | 12522895 | 7 | 17265 | oar3_OAR5_12505631 | oar3_OAR5_12522895 |
| 5 | 12938275 | 13053169 | 23 | 114895 | oar3_OAR5_12938275 | oar3_OAR5_13053169 |
| 5 | 13300390 | 13302150 | 4 | 1761 | oar3_OAR5_13300390 | oar3_OAR5_13302150 |
| 5 | 13465723 | 13537301 | 12 | 71579 | oar3_OAR5_13465723 | oar3_OAR5_13537301 |
| 5 | 14019984 | 14060870 | 11 | 40887 | oar3_OAR5_14019984 | oar3_OAR5_14060870 |
| 5 | 14054204 | 14144786 | 20 | 90583 | s43744.1 | oar3_OAR5_14144786 |
| 5 | 14286122 | 14365690 | 16 | 79569 | oar3_OAR5_14286122 | oar3_OAR5_14365690 |
| 5 | 14295638 | 14365690 | 15 | 70053 | oar3_OAR5_14295638 | oar3_OAR5_14365690 |
| 5 | 14301575 | 14356907 | 11 | 55333 | oar3_OAR5_14301575 | oar3_OAR5_14356907 |
| 5 | 14320525 | 14365690 | 11 | 45166 | oar3_OAR5_14320525 | oar3_OAR5_14365690 |
| 5 | 14631730 | 14660923 | 13 | 29194 | oar3_OAR5_14631730 | oar3_OAR5_14660923 |
| 5 | 14631730 | 14660923 | 13 | 29194 | oar3_OAR5_14631730 | oar3_OAR5_14660923 |
| 5 | 15620974 | 15692976 | 18 | 72003 | oar3_OAR5_15620974 | oar3_OAR5_15692976 |
| 5 | 15635592 | 15684515 | 9 | 48924 | oar3_OAR5_15635592 | oar3_OAR5_15684515 |
| 5 | 16110275 | 16214150 | 30 | 103876 | oar3_OAR5_16110275 | oar3_OAR5_16214150 |
| 5 | 16115157 | 16126024 | 6 | 10868 | oar3_OAR5_16115157 | oar3_OAR5_16126024 |
| 5 | 16118617 | 16216983 | 27 | 98367 | oar3_OAR5_16118617 | oar3_OAR5_16216983 |
| 5 | 16118617 | 16158542 | 10 | 39926 | oar3_OAR5_16118617 | oar3_OAR5_16158542 |
| 5 | 16190705 | 16216983 | 10 | 26279 | oar3_OAR5_16190705 | oar3_OAR5_16216983 |
| 5 | 16197209 | 16216983 | 9 | 19775 | oar3_OAR5_16197209 | oar3_OAR5_16216983 |
| 5 | 16203101 | 16216983 | 8 | 13883 | s29567.1 | oar3_OAR5_16216983 |
| 5 | 16203101 | 16225409 | 10 | 22309 | s29567.1 | oar3_OAR5_16225409 |
| 5 | 16206594 | 16216983 | 7 | 10390 | oar3_OAR5_16206594 | oar3_OAR5_16216983 |
| 5 | 16206594 | 16216983 | 7 | 10390 | oar3_OAR5_16206594 | oar3_OAR5_16216983 |
| 5 | 16677746 | 16717700 | 18 | 39955 | oar3_OAR5_16677746 | oar3_OAR5_16717700 |
| 5 | 16681548 | 16712102 | 14 | 30555 | oar3_OAR5_16681548 | oar3_OAR5_16712102 |
| 5 | 16683311 | 16818633 | 38 | 135323 | oar3_OAR5_16683311 | s39969.1 |
| 5 | 16683311 | 16712102 | 13 | 28792 | oar3_OAR5_16683311 | oar3_OAR5_16712102 |
| 5 | 16683311 | 16842873 | 41 | 159563 | oar3_OAR5_16683311 | oar3_OAR5_16842873 |
| 5 | 16683311 | 16795013 | 33 | 111703 | oar3_OAR5_16683311 | oar3_OAR5_16795013 |
| 5 | 16683311 | 16842873 | 41 | 159563 | oar3_OAR5_16683311 | oar3_OAR5_16842873 |
| 5 | 16684125 | 16702681 | 9 | 18557 | s62462.1 | oar3_OAR5_16702681 |
| 5 | 16685394 | 16821127 | 37 | 135734 | oar3_OAR5_16685394 | oar3_OAR5_16821127 |
| 5 | 16685394 | 16712102 | 10 | 26709 | oar3_OAR5_16685394 | oar3_OAR5_16712102 |
| 5 | 16685394 | 16698577 | 6 | 13184 | oar3_OAR5_16685394 | oar3_OAR5_16698577 |
| 5 | 17175437 | 17338642 | 30 | 163206 | oar3_OAR5_17175437 | oar3_OAR5_17338642 |
| 5 | 17200026 | 17291702 | 17 | 91677 | oar3_OAR5_17200026 | oar3_OAR5_17291702 |
| 5 | 17520965 | 17560475 | 7 | 39511 | oar3_OAR5_17520965 | oar3_OAR5_17560475 |
| 5 | 17623821 | 17653259 | 6 | 29439 | oar3_OAR5_17623821 | oar3_OAR5_17653259 |
| 5 | 17623821 | 17764619 | 34 | 140799 | oar3_OAR5_17623821 | oar3_OAR5_17764619 |
| 5 | 17680843 | 17766760 | 23 | 85918 | oar3_OAR5_17680843 | oar3_OAR5_17766760 |
| 5 | 17734828 | 17737727 | 4 | 2900 | oar3_OAR5_17734828 | oar3_OAR5_17737727 |
| 5 | 17734828 | 17797384 | 24 | 62557 | oar3_OAR5_17734828 | oar3_OAR5_17797384 |
| 5 | 17757309 | 17766760 | 6 | 9452 | oar3_OAR5_17757309 | oar3_OAR5_17766760 |
| 5 | 17757309 | 17766760 | 6 | 9452 | oar3_OAR5_17757309 | oar3_OAR5_17766760 |
| 5 | 17868416 | 17894768 | 8 | 26353 | oar3_OAR5_17868416 | oar3_OAR5_17894768 |
| 5 | 17875099 | 17934072 | 13 | 58974 | oar3_OAR5_17875099 | oar3_OAR5_17934072 |
| 5 | 17928182 | 17934072 | 5 | 5891 | oar3_OAR5_17928182 | oar3_OAR5_17934072 |
| 5 | 18245590 | 18266146 | 6 | 20557 | s71913.1 | oar3_OAR5_18266146 |
| 5 | 18250403 | 18266146 | 5 | 15744 | oar3_OAR5_18250403 | oar3_OAR5_18266146 |
| 5 | 18250403 | 18266146 | 5 | 15744 | oar3_OAR5_18250403 | oar3_OAR5_18266146 |
| 5 | 18250403 | 18266146 | 5 | 15744 | oar3_OAR5_18250403 | oar3_OAR5_18266146 |
| 5 | 18250403 | 18266146 | 5 | 15744 | oar3_OAR5_18250403 | oar3_OAR5_18266146 |
| 5 | 18250403 | 18266146 | 5 | 15744 | oar3_OAR5_18250403 | oar3_OAR5_18266146 |
| 5 | 18250403 | 18266146 | 5 | 15744 | oar3_OAR5_18250403 | oar3_OAR5_18266146 |
| 5 | 18444755 | 18516001 | 18 | 71247 | oar3_OAR5_18444755 | oar3_OAR5_18516001 |
| 5 | 18584921 | 18699832 | 21 | 114912 | oar3_OAR5_18584921 | oar3_OAR5_18699832 |
| 5 | 18634502 | 18664287 | 9 | 29786 | oar3_OAR5_18634502 | oar3_OAR5_18664287 |
| 5 | 18634502 | 18738106 | 23 | 103605 | oar3_OAR5_18634502 | oar3_OAR5_18738106 |
| 5 | 18688934 | 19002025 | 54 | 313092 | oar3_OAR5_18688934 | oar3_OAR5_19002025 |
| 5 | 18688934 | 18755012 | 13 | 66079 | oar3_OAR5_18688934 | oar3_OAR5_18755012 |
| 5 | 18688934 | 18755012 | 13 | 66079 | oar3_OAR5_18688934 | oar3_OAR5_18755012 |
| 5 | 18821154 | 19002025 | 27 | 180872 | oar3_OAR5_18821154 | oar3_OAR5_19002025 |
| 5 | 18821154 | 19002025 | 27 | 180872 | oar3_OAR5_18821154 | oar3_OAR5_19002025 |
| 5 | 18831846 | 18854145 | 8 | 22300 | oar3_OAR5_18831846 | oar3_OAR5_18854145 |
| 5 | 18853954 | 18897055 | 6 | 43102 | oar3_OAR5_18853954 | oar3_OAR5_18897055 |
| 5 | 29854282 | 29892161 | 9 | 37880 | oar3_OAR5_29854282 | oar3_OAR5_29892161 |
| 5 | 35548622 | 35561233 | 10 | 12612 | oar3_OAR5_35548622 | oar3_OAR5_35561233 |
| 5 | 35548622 | 35653159 | 30 | 104538 | oar3_OAR5_35548622 | oar3_OAR5_35653159 |
| 5 | 35548622 | 35555853 | 7 | 7232 | oar3_OAR5_35548622 | oar3_OAR5_35555853 |
| 5 | 35553113 | 35557216 | 7 | 4104 | oar3_OAR5_35553113 | s58626.1 |
| 5 | 35553113 | 35571684 | 10 | 18572 | oar3_OAR5_35553113 | oar3_OAR5_35571684 |
| 5 | 35553113 | 35555853 | 6 | 2741 | oar3_OAR5_35553113 | oar3_OAR5_35555853 |
| 5 | 35791264 | 35816109 | 5 | 24846 | oar3_OAR5_35791264 | oar3_OAR5_35816109 |
| 5 | 35791264 | 35816109 | 5 | 24846 | oar3_OAR5_35791264 | oar3_OAR5_35816109 |
| 5 | 36395813 | 36485710 | 19 | 89898 | oar3_OAR5_36395813 | oar3_OAR5_36485710 |
| 5 | 36472972 | 36485710 | 5 | 12739 | oar3_OAR5_36472972 | oar3_OAR5_36485710 |
| 5 | 36696165 | 36699532 | 7 | 3368 | oar3_OAR5_36696165 | oar3_OAR5_36699532 |
| 5 | 39181048 | 39289081 | 23 | 108034 | oar3_OAR5_39181048 | oar3_OAR5_39289081 |
| 5 | 39184107 | 39289081 | 22 | 104975 | oar3_OAR5_39184107 | oar3_OAR5_39289081 |
| 5 | 39199571 | 39289081 | 20 | 89511 | oar3_OAR5_39199571 | oar3_OAR5_39289081 |
| 5 | 40491192 | 40508408 | 6 | 17217 | oar3_OAR5_40491192 | s74709.1 |
| 5 | 40491192 | 40581330 | 18 | 90139 | oar3_OAR5_40491192 | oar3_OAR5_40581330 |
| 5 | 40491192 | 40861912 | 50 | 370721 | oar3_OAR5_40491192 | oar3_OAR5_40861912 |
| 5 | 40491826 | 40562582 | 15 | 70757 | oar3_OAR5_40491826 | oar3_OAR5_40562582 |
| 5 | 40559403 | 40699527 | 16 | 140125 | oar3_OAR5_40559403 | oar3_OAR5_40699527 |
| 5 | 40562582 | 40632288 | 9 | 69707 | oar3_OAR5_40562582 | oar3_OAR5_40632288 |
| 5 | 40562582 | 40723135 | 19 | 160554 | oar3_OAR5_40562582 | oar3_OAR5_40723135 |
| 5 | 40606479 | 40748921 | 21 | 142443 | oar3_OAR5_40606479 | oar3_OAR5_40748921 |
| 5 | 40606479 | 40668603 | 8 | 62125 | oar3_OAR5_40606479 | OAR5_44370956.1 |
| 5 | 40632288 | 40740170 | 14 | 107883 | oar3_OAR5_40632288 | oar3_OAR5_40740170 |
| 5 | 40713566 | 40740170 | 7 | 26605 | s72060.1 | oar3_OAR5_40740170 |
| 5 | 40713566 | 40810293 | 14 | 96728 | s72060.1 | oar3_OAR5_40810293 |
| 5 | 40713566 | 40740170 | 7 | 26605 | s72060.1 | oar3_OAR5_40740170 |
| 5 | 40951501 | 41124198 | 14 | 172698 | oar3_OAR5_40951501 | oar3_OAR5_41124198 |
| 5 | 41013546 | 41146299 | 13 | 132754 | oar3_OAR5_41013546 | oar3_OAR5_41146299 |
| 5 | 41013546 | 41124198 | 10 | 110653 | oar3_OAR5_41013546 | oar3_OAR5_41124198 |
| 5 | 41229955 | 41273804 | 7 | 43850 | oar3_OAR5_41229955 | oar3_OAR5_41273804 |
| 5 | 41229955 | 41273804 | 7 | 43850 | oar3_OAR5_41229955 | oar3_OAR5_41273804 |
| 5 | 41231733 | 41382558 | 26 | 150826 | oar3_OAR5_41231733 | oar3_OAR5_41382558 |
| 5 | 41321757 | 41382558 | 18 | 60802 | oar3_OAR5_41321757 | oar3_OAR5_41382558 |
| 5 | 41332156 | 41360939 | 10 | 28784 | oar3_OAR5_41332156 | oar3_OAR5_41360939 |
| 5 | 41332156 | 41360939 | 10 | 28784 | oar3_OAR5_41332156 | oar3_OAR5_41360939 |
| 5 | 41354145 | 41386035 | 8 | 31891 | oar3_OAR5_41354145 | oar3_OAR5_41386035 |
| 5 | 41357859 | 41386035 | 7 | 28177 | s23065.1 | oar3_OAR5_41386035 |
| 5 | 41810351 | 41822201 | 9 | 11851 | oar3_OAR5_41810351 | oar3_OAR5_41822201 |
| 5 | 41810351 | 41816133 | 6 | 5783 | oar3_OAR5_41810351 | oar3_OAR5_41816133 |
| 5 | 41810351 | 41822201 | 9 | 11851 | oar3_OAR5_41810351 | oar3_OAR5_41822201 |
| 5 | 41810351 | 41844222 | 17 | 33872 | oar3_OAR5_41810351 | oar3_OAR5_41844222 |
| 5 | 42205722 | 42211706 | 5 | 5985 | oar3_OAR5_42205722 | oar3_OAR5_42211706 |
| 5 | 43845834 | 43849320 | 5 | 3487 | oar3_OAR5_43845834 | s70893.1 |
| 5 | 48233642 | 48289428 | 14 | 55787 | oar3_OAR5_48233642 | oar3_OAR5_48289428 |
| 5 | 48233642 | 48485897 | 40 | 252256 | oar3_OAR5_48233642 | oar3_OAR5_48485897 |
| 5 | 48248624 | 48258512 | 3 | 9889 | oar3_OAR5_48248624 | oar3_OAR5_48258512 |
| 5 | 48248624 | 48299659 | 10 | 51036 | oar3_OAR5_48248624 | oar3_OAR5_48299659 |
| 5 | 48248624 | 48299659 | 10 | 51036 | oar3_OAR5_48248624 | oar3_OAR5_48299659 |
| 5 | 49257922 | 49354105 | 28 | 96184 | oar3_OAR5_49257922 | oar3_OAR5_49354105 |
| 5 | 49326763 | 49346970 | 8 | 20208 | oar3_OAR5_49326763 | oar3_OAR5_49346970 |
| 5 | 49816770 | 49868840 | 25 | 52071 | oar3_OAR5_49816770 | oar3_OAR5_49868840 |
| 5 | 49816770 | 49836561 | 12 | 19792 | oar3_OAR5_49816770 | oar3_OAR5_49836561 |
| 5 | 49820899 | 49856780 | 20 | 35882 | oar3_OAR5_49820899 | oar3_OAR5_49856780 |
| 5 | 55120252 | 55161860 | 8 | 41609 | oar3_OAR5_55120252 | oar3_OAR5_55161860 |
| 5 | 55151055 | 55160779 | 5 | 9725 | oar3_OAR5_55151055 | oar3_OAR5_55160779 |
| 5 | 58285914 | 58288154 | 3 | 2241 | oar3_OAR5_58285914 | oar3_OAR5_58288154 |
| 5 | 58975033 | 59153140 | 48 | 178108 | oar3_OAR5_58975033 | oar3_OAR5_59153140 |
| 5 | 59465860 | 59475661 | 5 | 9802 | oar3_OAR5_59465860 | oar3_OAR5_59475661 |
| 5 | 59816504 | 59998629 | 38 | 182126 | s44821.1 | oar3_OAR5_59998629 |
| 5 | 75096880 | 75101514 | 3 | 4635 | oar3_OAR5_75096880 | oar3_OAR5_75101514 |
| 5 | 83135394 | 83213540 | 17 | 78147 | oar3_OAR5_83135394 | oar3_OAR5_83213540 |
| 5 | 83145537 | 83213540 | 15 | 68004 | oar3_OAR5_83145537 | oar3_OAR5_83213540 |
| 5 | 90652479 | 90661782 | 4 | 9304 | oar3_OAR5_90652479 | oar3_OAR5_90661782 |
| 5 | 1.03E+08 | 1.03E+08 | 4 | 5247 | oar3_OAR5_102779903 | OAR5_111889268.1 |
| 5 | 1.03E+08 | 1.03E+08 | 4 | 5247 | oar3_OAR5_102779903 | OAR5_111889268.1 |
| 5 | 1.06E+08 | 1.06E+08 | 33 | 101643 | s67854.1 | oar3_OAR5_106259400 |
| 5 | 1.06E+08 | 1.06E+08 | 22 | 61856 | oar3_OAR5_106178522 | oar3_OAR5_106240377 |
| 5 | 1.06E+08 | 1.06E+08 | 4 | 776 | oar3_OAR5_106239602 | oar3_OAR5_106240377 |
| 6 | 1244494 | 1265938 | 4 | 21445 | oar3_OAR6_1244494 | oar3_OAR6_1265938 |
| 6 | 21746226 | 21755240 | 3 | 9015 | oar3_OAR6_21746226 | oar3_OAR6_21755240 |
| 6 | 25642049 | 25647719 | 4 | 5671 | oar3_OAR6_25642049 | oar3_OAR6_25647719 |
| 6 | 34274575 | 34308545 | 9 | 33971 | oar3_OAR6_34274575 | oar3_OAR6_34308545 |
| 6 | 37353878 | 37359421 | 6 | 5544 | oar3_OAR6_37353878 | OAR6_41583796.1 |
| 6 | 51471221 | 51485243 | 4 | 14023 | oar3_OAR6_51471221 | oar3_OAR6_51485243 |
| 6 | 51471221 | 51485243 | 4 | 14023 | oar3_OAR6_51471221 | oar3_OAR6_51485243 |
| 6 | 59074588 | 59129160 | 15 | 54573 | oar3_OAR6_59074588 | oar3_OAR6_59129160 |
| 6 | 67472133 | 67484202 | 4 | 12070 | OAR6_73940599.1 | oar3_OAR6_67484202 |
| 6 | 74004726 | 74134542 | 22 | 129817 | oar3_OAR6_74004726 | oar3_OAR6_74134542 |
| 6 | 78459562 | 78510804 | 9 | 51243 | oar3_OAR6_78459562 | oar3_OAR6_78510804 |
| 6 | 78459562 | 78483214 | 6 | 23653 | oar3_OAR6_78459562 | oar3_OAR6_78483214 |
| 6 | 79002905 | 79204372 | 36 | 201468 | oar3_OAR6_79002905 | oar3_OAR6_79204372 |
| 6 | 85406274 | 85416541 | 63 | 10268 | oar3_OAR6_85406274 | oar3_OAR6_85416541 |
| 6 | 99004147 | 99004493 | 4 | 347 | oar3_OAR6_99004147 | oar3_OAR6_99004493 |
| 6 | 99004147 | 99004493 | 4 | 347 | oar3_OAR6_99004147 | oar3_OAR6_99004493 |
| 6 | 99004147 | 99011271 | 5 | 7125 | oar3_OAR6_99004147 | oar3_OAR6_99011271 |
| 6 | 99004147 | 99004493 | 4 | 347 | oar3_OAR6_99004147 | oar3_OAR6_99004493 |
| 6 | 1.02E+08 | 1.02E+08 | 40 | 120679 | oar3_OAR6_102185688 | oar3_OAR6_102306366 |
| 6 | 1.02E+08 | 1.02E+08 | 6 | 12595 | oar3_OAR6_102255817 | oar3_OAR6_102268411 |
| 6 | 1.02E+08 | 1.02E+08 | 7 | 15441 | oar3_OAR6_102255817 | oar3_OAR6_102271257 |
| 6 | 1.02E+08 | 1.02E+08 | 7 | 15441 | oar3_OAR6_102255817 | oar3_OAR6_102271257 |
| 6 | 1.02E+08 | 1.02E+08 | 7 | 8652 | oar3_OAR6_102297715 | oar3_OAR6_102306366 |
| 6 | 1.13E+08 | 1.13E+08 | 11 | 49641 | oar3_OAR6_112837715 | oar3_OAR6_112887355 |
| 6 | 1.13E+08 | 1.13E+08 | 10 | 40894 | oar3_OAR6_112837715 | oar3_OAR6_112878608 |
| 6 | 1.13E+08 | 1.13E+08 | 12 | 54459 | oar3_OAR6_112837715 | oar3_OAR6_112892173 |
| 6 | 1.13E+08 | 1.13E+08 | 7 | 29264 | oar3_OAR6_112868694 | oar3_OAR6_112897957 |
| 6 | 1.13E+08 | 1.13E+08 | 8 | 27817 | oar3_OAR6_112887355 | oar3_OAR6_112915171 |
| 6 | 1.13E+08 | 1.13E+08 | 7 | 15009 | oar3_OAR6_112887355 | oar3_OAR6_112902363 |
| 6 | 1.13E+08 | 1.13E+08 | 17 | 63355 | oar3_OAR6_113184138 | oar3_OAR6_113247492 |
| 6 | 1.13E+08 | 1.13E+08 | 40 | 130287 | oar3_OAR6_113352099 | oar3_OAR6_113482385 |
| 6 | 1.13E+08 | 1.13E+08 | 25 | 70430 | s35073.1 | oar3_OAR6_113482385 |
| 6 | 1.13E+08 | 1.13E+08 | 9 | 15495 | oar3_OAR6_113466891 | oar3_OAR6_113482385 |
| 6 | 1.14E+08 | 1.14E+08 | 37 | 134821 | oar3_OAR6_113703896 | oar3_OAR6_113838716 |
| 6 | 1.14E+08 | 1.14E+08 | 65 | 259506 | oar3_OAR6_113703896 | oar3_OAR6_113963401 |
| 6 | 1.14E+08 | 1.14E+08 | 5 | 9948 | oar3_OAR6_113703896 | oar3_OAR6_113713843 |
| 6 | 1.14E+08 | 1.14E+08 | 8 | 26425 | oar3_OAR6_113712358 | oar3_OAR6_113738782 |
| 6 | 1.14E+08 | 1.14E+08 | 5 | 9704 | oar3_OAR6_113729079 | oar3_OAR6_113738782 |
| 6 | 1.14E+08 | 1.14E+08 | 12 | 40625 | oar3_OAR6_113791072 | oar3_OAR6_113831696 |
| 6 | 1.14E+08 | 1.14E+08 | 7 | 16372 | oar3_OAR6_113815325 | oar3_OAR6_113831696 |
| 6 | 1.14E+08 | 1.14E+08 | 33 | 132381 | oar3_OAR6_113825607 | s36182.1 |
| 6 | 1.14E+08 | 1.14E+08 | 16 | 68552 | s47946.1 | oar3_OAR6_113950389 |
| 6 | 1.14E+08 | 1.14E+08 | 11 | 30577 | oar3_OAR6_113927411 | s36182.1 |
| 6 | 1.14E+08 | 1.14E+08 | 13 | 35991 | oar3_OAR6_113927411 | oar3_OAR6_113963401 |
| 6 | 1.14E+08 | 1.14E+08 | 11 | 30577 | oar3_OAR6_113927411 | s36182.1 |
| 6 | 1.14E+08 | 1.14E+08 | 11 | 30577 | oar3_OAR6_113927411 | s36182.1 |
| 6 | 1.14E+08 | 1.14E+08 | 9 | 22979 | oar3_OAR6_113927411 | oar3_OAR6_113950389 |
| 6 | 1.14E+08 | 1.14E+08 | 13 | 35991 | oar3_OAR6_113927411 | oar3_OAR6_113963401 |
| 6 | 1.14E+08 | 1.14E+08 | 11 | 30577 | oar3_OAR6_113927411 | s36182.1 |
| 6 | 1.14E+08 | 1.14E+08 | 11 | 30600 | oar3_OAR6_113932802 | oar3_OAR6_113963401 |
| 6 | 1.14E+08 | 1.14E+08 | 34 | 91257 | oar3_OAR6_113940654 | oar3_OAR6_114031910 |
| 6 | 1.14E+08 | 1.14E+08 | 11 | 14575 | oar3_OAR6_113992616 | oar3_OAR6_114007190 |
| 6 | 1.14E+08 | 1.14E+08 | 12 | 19336 | oar3_OAR6_113995457 | oar3_OAR6_114014792 |
| 6 | 1.14E+08 | 1.14E+08 | 10 | 11734 | oar3_OAR6_113995457 | oar3_OAR6_114007190 |
| 6 | 1.14E+08 | 1.14E+08 | 11 | 15787 | oar3_OAR6_113999006 | oar3_OAR6_114014792 |
| 6 | 1.14E+08 | 1.14E+08 | 16 | 32905 | oar3_OAR6_113999006 | oar3_OAR6_114031910 |
| 6 | 1.14E+08 | 1.14E+08 | 11 | 31946 | oar3_OAR6_114130520 | oar3_OAR6_114162465 |
| 6 | 1.14E+08 | 1.14E+08 | 7 | 28843 | oar3_OAR6_114190456 | oar3_OAR6_114219298 |
| 6 | 1.14E+08 | 1.14E+08 | 5 | 18113 | oar3_OAR6_114201186 | oar3_OAR6_114219298 |
| 6 | 1.14E+08 | 1.14E+08 | 18 | 68724 | oar3_OAR6_114253910 | oar3_OAR6_114322633 |
| 6 | 1.14E+08 | 1.14E+08 | 14 | 43610 | oar3_OAR6_114279024 | oar3_OAR6_114322633 |
| 6 | 1.14E+08 | 1.14E+08 | 36 | 122462 | oar3_OAR6_114279024 | oar3_OAR6_114401485 |
| 6 | 1.14E+08 | 1.14E+08 | 7 | 24222 | oar3_OAR6_114298412 | oar3_OAR6_114322633 |
| 6 | 1.14E+08 | 1.14E+08 | 21 | 73643 | oar3_OAR6_114310337 | oar3_OAR6_114383979 |
| 6 | 1.14E+08 | 1.14E+08 | 5 | 6255 | oar3_OAR6_114350263 | oar3_OAR6_114356517 |
| 6 | 1.14E+08 | 1.14E+08 | 12 | 33493 | oar3_OAR6_114350263 | oar3_OAR6_114383755 |
| 6 | 1.14E+08 | 1.14E+08 | 9 | 23147 | oar3_OAR6_114350263 | oar3_OAR6_114373409 |
| 6 | 1.14E+08 | 1.14E+08 | 31 | 112373 | oar3_OAR6_114354188 | oar3_OAR6_114466560 |
| 6 | 1.14E+08 | 1.14E+08 | 12 | 52787 | oar3_OAR6_114413774 | oar3_OAR6_114466560 |
| 6 | 1.14E+08 | 1.15E+08 | 21 | 101279 | oar3_OAR6_114413774 | oar3_OAR6_114515052 |
| 6 | 1.14E+08 | 1.14E+08 | 5 | 18969 | oar3_OAR6_114421667 | oar3_OAR6_114440635 |
| 6 | 1.14E+08 | 1.14E+08 | 4 | 10104 | oar3_OAR6_114430532 | oar3_OAR6_114440635 |
| 6 | 1.14E+08 | 1.14E+08 | 3 | 5731 | oar3_OAR6_114430532 | oar3_OAR6_114436262 |
| 6 | 1.14E+08 | 1.14E+08 | 3 | 5731 | oar3_OAR6_114430532 | oar3_OAR6_114436262 |
| 6 | 1.14E+08 | 1.15E+08 | 8 | 32803 | oar3_OAR6_114482250 | oar3_OAR6_114515052 |
| 6 | 1.14E+08 | 1.15E+08 | 22 | 104646 | oar3_OAR6_114488785 | oar3_OAR6_114593430 |
| 6 | 1.14E+08 | 1.15E+08 | 5 | 19040 | oar3_OAR6_114496013 | oar3_OAR6_114515052 |
| 6 | 1.14E+08 | 1.15E+08 | 5 | 19040 | oar3_OAR6_114496013 | oar3_OAR6_114515052 |
| 6 | 1.15E+08 | 1.15E+08 | 5 | 3533 | oar3_OAR6_114589898 | oar3_OAR6_114593430 |
| 6 | 1.15E+08 | 1.15E+08 | 6 | 24634 | oar3_OAR6_114741394 | oar3_OAR6_114766027 |
| 6 | 1.15E+08 | 1.15E+08 | 6 | 24634 | oar3_OAR6_114741394 | oar3_OAR6_114766027 |
| 6 | 1.15E+08 | 1.15E+08 | 16 | 106622 | oar3_OAR6_114753824 | oar3_OAR6_114860445 |
| 6 | 1.15E+08 | 1.15E+08 | 7 | 24860 | oar3_OAR6_114753824 | CL635944_160.1 |
| 6 | 1.15E+08 | 1.15E+08 | 8 | 40268 | oar3_OAR6_114753824 | oar3_OAR6_114794091 |
| 6 | 1.15E+08 | 1.15E+08 | 55 | 300270 | CL635944_160.1 | oar3_OAR6_115078952 |
| 6 | 1.15E+08 | 1.15E+08 | 21 | 124411 | oar3_OAR6_114829699 | oar3_OAR6_114954109 |
| 6 | 1.15E+08 | 1.15E+08 | 58 | 276987 | s75351.1 | oar3_OAR6_115114591 |
| 6 | 1.15E+08 | 1.15E+08 | 5 | 9821 | oar3_OAR6_114859914 | oar3_OAR6_114869734 |
| 6 | 1.15E+08 | 1.15E+08 | 4 | 7834 | oar3_OAR6_114910280 | oar3_OAR6_114918113 |
| 6 | 1.15E+08 | 1.15E+08 | 67 | 283088 | oar3_OAR6_114917900 | oar3_OAR6_115200987 |
| 6 | 1.15E+08 | 1.15E+08 | 35 | 161053 | oar3_OAR6_114917900 | oar3_OAR6_115078952 |
| 6 | 1.15E+08 | 1.15E+08 | 27 | 119125 | oar3_OAR6_114954109 | oar3_OAR6_115073233 |
| 6 | 1.15E+08 | 1.15E+08 | 27 | 119125 | oar3_OAR6_114954109 | oar3_OAR6_115073233 |
| 6 | 1.15E+08 | 1.15E+08 | 27 | 119672 | oar3_OAR6_114960701 | oar3_OAR6_115080372 |
| 6 | 1.15E+08 | 1.15E+08 | 32 | 114950 | oar3_OAR6_114999642 | oar3_OAR6_115114591 |
| 6 | 1.15E+08 | 1.15E+08 | 21 | 77537 | oar3_OAR6_114999642 | oar3_OAR6_115077178 |
| 6 | 1.15E+08 | 1.15E+08 | 9 | 15312 | oar3_OAR6_115016063 | oar3_OAR6_115031374 |
| 6 | 1.15E+08 | 1.15E+08 | 14 | 38912 | oar3_OAR6_115023538 | oar3_OAR6_115062449 |
| 6 | 1.15E+08 | 1.15E+08 | 7 | 7837 | oar3_OAR6_115023538 | oar3_OAR6_115031374 |
| 6 | 1.15E+08 | 1.15E+08 | 15 | 54715 | oar3_OAR6_115113649 | oar3_OAR6_115168363 |
| 6 | 1.15E+08 | 1.15E+08 | 8 | 20248 | oar3_OAR6_115148116 | oar3_OAR6_115168363 |
| 6 | 1.15E+08 | 1.15E+08 | 13 | 110937 | oar3_OAR6_115324704 | oar3_OAR6_115435640 |
| 6 | 1.15E+08 | 1.16E+08 | 34 | 287842 | oar3_OAR6_115324704 | oar3_OAR6_115612545 |
| 6 | 1.15E+08 | 1.15E+08 | 6 | 18270 | oar3_OAR6_115398727 | oar3_OAR6_115416996 |
| 6 | 1.16E+08 | 1.16E+08 | 10 | 39660 | oar3_OAR6_115727183 | oar3_OAR6_115766842 |
| 6 | 1.16E+08 | 1.16E+08 | 6 | 25522 | oar3_OAR6_115727183 | oar3_OAR6_115752704 |
| 6 | 1.16E+08 | 1.16E+08 | 7 | 22095 | oar3_OAR6_115744748 | oar3_OAR6_115766842 |
| 6 | 1.16E+08 | 1.16E+08 | 3 | 7957 | oar3_OAR6_115744748 | oar3_OAR6_115752704 |
| 6 | 1.16E+08 | 1.16E+08 | 3 | 7957 | oar3_OAR6_115744748 | oar3_OAR6_115752704 |
| 6 | 1.16E+08 | 1.16E+08 | 3 | 7957 | oar3_OAR6_115744748 | oar3_OAR6_115752704 |
| 6 | 1.16E+08 | 1.17E+08 | 67 | 913407 | oar3_OAR6_115908289 | oar3_OAR6_116821695 |
| 6 | 1.16E+08 | 1.16E+08 | 5 | 31494 | oar3_OAR6_115928842 | OAR6_117616126.1 |
| 6 | 1.16E+08 | 1.16E+08 | 16 | 146963 | oar3_OAR6_115928842 | oar3_OAR6_116075804 |
| 6 | 1.16E+08 | 1.16E+08 | 11 | 62222 | oar3_OAR6_115928842 | s30544.1 |
| 6 | 1.16E+08 | 1.16E+08 | 23 | 196982 | oar3_OAR6_115928842 | oar3_OAR6_116125823 |
| 6 | 1.16E+08 | 1.16E+08 | 23 | 269828 | oar3_OAR6_115981701 | oar3_OAR6_116251528 |
| 6 | 1.16E+08 | 1.17E+08 | 59 | 839995 | oar3_OAR6_115981701 | oar3_OAR6_116821695 |
| 6 | 1.16E+08 | 1.16E+08 | 4 | 23874 | oar3_OAR6_116051931 | oar3_OAR6_116075804 |
| 6 | 1.16E+08 | 1.16E+08 | 10 | 58667 | oar3_OAR6_116067157 | oar3_OAR6_116125823 |
| 6 | 1.16E+08 | 1.16E+08 | 10 | 58667 | oar3_OAR6_116067157 | oar3_OAR6_116125823 |
| 6 | 1.16E+08 | 1.16E+08 | 10 | 58667 | oar3_OAR6_116067157 | oar3_OAR6_116125823 |
| 6 | 1.16E+08 | 1.16E+08 | 9 | 50743 | oar3_OAR6_116075081 | oar3_OAR6_116125823 |
| 6 | 1.16E+08 | 1.16E+08 | 13 | 150682 | oar3_OAR6_116100847 | oar3_OAR6_116251528 |
| 6 | 1.16E+08 | 1.16E+08 | 4 | 21204 | oar3_OAR6_116104620 | oar3_OAR6_116125823 |
| 6 | 1.16E+08 | 1.16E+08 | 4 | 21204 | oar3_OAR6_116104620 | oar3_OAR6_116125823 |
| 6 | 1.16E+08 | 1.16E+08 | 15 | 201790 | oar3_OAR6_116104620 | oar3_OAR6_116306409 |
| 6 | 1.16E+08 | 1.16E+08 | 10 | 137863 | oar3_OAR6_116113666 | oar3_OAR6_116251528 |
| 6 | 1.16E+08 | 1.17E+08 | 17 | 253020 | oar3_OAR6_116297338 | oar3_OAR6_116550357 |
| 6 | 1.16E+08 | 1.17E+08 | 7 | 108884 | s14138.1 | oar3_OAR6_116550357 |
| 6 | 1.16E+08 | 1.17E+08 | 18 | 329183 | s14138.1 | oar3_OAR6_116770656 |
| 6 | 1.16E+08 | 1.17E+08 | 7 | 108884 | s14138.1 | oar3_OAR6_116550357 |
| 6 | 1.16E+08 | 1.17E+08 | 26 | 380222 | s14138.1 | oar3_OAR6_116821695 |
| 6 | 1.16E+08 | 1.17E+08 | 18 | 329183 | s14138.1 | oar3_OAR6_116770656 |
| 6 | 1.16E+08 | 1.17E+08 | 7 | 108884 | s14138.1 | oar3_OAR6_116550357 |
| 6 | 1.16E+08 | 1.17E+08 | 7 | 108884 | s14138.1 | oar3_OAR6_116550357 |
| 6 | 1.17E+08 | 1.17E+08 | 5 | 33157 | oar3_OAR6_116517201 | oar3_OAR6_116550357 |
| 6 | 1.17E+08 | 1.17E+08 | 5 | 33157 | oar3_OAR6_116517201 | oar3_OAR6_116550357 |
| 6 | 1.17E+08 | 1.17E+08 | 4 | 25249 | oar3_OAR6_116525109 | oar3_OAR6_116550357 |
| 6 | 1.17E+08 | 1.17E+08 | 4 | 25249 | oar3_OAR6_116525109 | oar3_OAR6_116550357 |
| 6 | 1.17E+08 | 1.17E+08 | 13 | 82232 | oar3_OAR6_116739464 | oar3_OAR6_116821695 |
| 6 | 1.17E+08 | 1.17E+08 | 9 | 164526 | oar3_OAR6_116785413 | oar3_OAR6_116949938 |
| 6 | 1.17E+08 | 1.17E+08 | 6 | 140349 | oar3_OAR6_116809590 | oar3_OAR6_116949938 |
| 7 | 9361626 | 9434449 | 15 | 72824 | OAR7_9376658.1 | oar3_OAR7_9434449 |
| 7 | 11766244 | 11790491 | 10 | 24248 | oar3_OAR7_11766244 | oar3_OAR7_11790491 |
| 7 | 11770733 | 11801898 | 11 | 31166 | oar3_OAR7_11770733 | s61866.1 |
| 7 | 14807041 | 14816726 | 6 | 9686 | oar3_OAR7_14807041 | s51937.1 |
| 7 | 15650582 | 15688527 | 14 | 37946 | oar3_OAR7_15650582 | oar3_OAR7_15688527 |
| 7 | 16301367 | 16372377 | 22 | 71011 | oar3_OAR7_16301367 | oar3_OAR7_16372377 |
| 7 | 20147611 | 20177512 | 9 | 29902 | oar3_OAR7_20147611 | oar3_OAR7_20177512 |
| 7 | 20147611 | 20177512 | 9 | 29902 | oar3_OAR7_20147611 | oar3_OAR7_20177512 |
| 7 | 20147611 | 20177512 | 9 | 29902 | oar3_OAR7_20147611 | oar3_OAR7_20177512 |
| 7 | 20147611 | 20177512 | 9 | 29902 | oar3_OAR7_20147611 | oar3_OAR7_20177512 |
| 7 | 20153292 | 20177512 | 7 | 24221 | s58125.1 | oar3_OAR7_20177512 |
| 7 | 20153292 | 20177512 | 7 | 24221 | s58125.1 | oar3_OAR7_20177512 |
| 7 | 20164636 | 20177512 | 5 | 12877 | oar3_OAR7_20164636 | oar3_OAR7_20177512 |
| 7 | 20519968 | 20550375 | 9 | 30408 | oar3_OAR7_20519968 | oar3_OAR7_20550375 |
| 7 | 20519968 | 20556382 | 10 | 36415 | oar3_OAR7_20519968 | OAR7_21409209.1 |
| 7 | 20598890 | 20703691 | 21 | 104802 | oar3_OAR7_20598890 | oar3_OAR7_20703691 |
| 7 | 20689379 | 20703691 | 9 | 14313 | oar3_OAR7_20689379 | oar3_OAR7_20703691 |
| 7 | 20689379 | 20694878 | 5 | 5500 | oar3_OAR7_20689379 | oar3_OAR7_20694878 |
| 7 | 21003015 | 21033433 | 10 | 30419 | oar3_OAR7_21003015 | oar3_OAR7_21033433 |
| 7 | 21003015 | 21033433 | 10 | 30419 | oar3_OAR7_21003015 | oar3_OAR7_21033433 |
| 7 | 21003015 | 21033433 | 10 | 30419 | oar3_OAR7_21003015 | oar3_OAR7_21033433 |
| 7 | 21019784 | 21033433 | 6 | 13650 | oar3_OAR7_21019784 | oar3_OAR7_21033433 |
| 7 | 21019784 | 21033433 | 6 | 13650 | oar3_OAR7_21019784 | oar3_OAR7_21033433 |
| 7 | 21026420 | 21033433 | 4 | 7014 | oar3_OAR7_21026420 | oar3_OAR7_21033433 |
| 7 | 21143416 | 21219575 | 13 | 76160 | oar3_OAR7_21143416 | oar3_OAR7_21219575 |
| 7 | 22476125 | 22638714 | 6 | 162590 | oar3_OAR7_22476125 | oar3_OAR7_22638714 |
| 7 | 23219994 | 23255426 | 13 | 35433 | oar3_OAR7_23219994 | oar3_OAR7_23255426 |
| 7 | 23219994 | 23243026 | 12 | 23033 | oar3_OAR7_23219994 | oar3_OAR7_23243026 |
| 7 | 23219994 | 23255426 | 13 | 35433 | oar3_OAR7_23219994 | oar3_OAR7_23255426 |
| 7 | 23224140 | 23243026 | 10 | 18887 | oar3_OAR7_23224140 | oar3_OAR7_23243026 |
| 7 | 23224140 | 23237983 | 8 | 13844 | oar3_OAR7_23224140 | oar3_OAR7_23237983 |
| 7 | 25264028 | 25273864 | 7 | 9837 | oar3_OAR7_25264028 | oar3_OAR7_25273864 |
| 7 | 26452668 | 26463583 | 7 | 10916 | DU377445_470.1 | oar3_OAR7_26463583 |
| 7 | 32906114 | 33001901 | 18 | 95788 | oar3_OAR7_32906114 | oar3_OAR7_33001901 |
| 7 | 32906114 | 33001901 | 18 | 95788 | oar3_OAR7_32906114 | oar3_OAR7_33001901 |
| 7 | 32906114 | 32998823 | 17 | 92710 | oar3_OAR7_32906114 | oar3_OAR7_32998823 |
| 7 | 32906114 | 32921865 | 6 | 15752 | oar3_OAR7_32906114 | s02962.1 |
| 7 | 32911371 | 32921865 | 4 | 10495 | oar3_OAR7_32911371 | s02962.1 |
| 7 | 32911371 | 32921865 | 4 | 10495 | oar3_OAR7_32911371 | s02962.1 |
| 7 | 32958587 | 33001901 | 10 | 43315 | oar3_OAR7_32958587 | oar3_OAR7_33001901 |
| 7 | 33221692 | 33233845 | 9 | 12154 | oar3_OAR7_33221692 | oar3_OAR7_33233845 |
| 7 | 33221692 | 33233845 | 9 | 12154 | oar3_OAR7_33221692 | oar3_OAR7_33233845 |
| 7 | 33945895 | 33984469 | 16 | 38575 | oar3_OAR7_33945895 | oar3_OAR7_33984469 |
| 7 | 33945895 | 33971142 | 15 | 25248 | oar3_OAR7_33945895 | s31602.1 |
| 7 | 33962733 | 33984469 | 12 | 21737 | oar3_OAR7_33962733 | oar3_OAR7_33984469 |
| 7 | 34241384 | 34283320 | 26 | 41937 | oar3_OAR7_34241384 | oar3_OAR7_34283320 |
| 7 | 34241384 | 34290402 | 31 | 49019 | oar3_OAR7_34241384 | oar3_OAR7_34290402 |
| 7 | 34269109 | 34320896 | 40 | 51788 | oar3_OAR7_34269109 | oar3_OAR7_34320896 |
| 7 | 34277258 | 34302595 | 21 | 25338 | oar3_OAR7_34277258 | oar3_OAR7_34302595 |
| 7 | 34281593 | 34283873 | 5 | 2281 | oar3_OAR7_34281593 | oar3_OAR7_34283873 |
| 7 | 34289609 | 34299506 | 6 | 9898 | oar3_OAR7_34289609 | oar3_OAR7_34299506 |
| 7 | 34298714 | 34307556 | 9 | 8843 | oar3_OAR7_34298714 | oar3_OAR7_34307556 |
| 7 | 35030356 | 35047065 | 22 | 16710 | oar3_OAR7_35030356 | oar3_OAR7_35047065 |
| 7 | 35030356 | 35039554 | 21 | 9199 | oar3_OAR7_35030356 | oar3_OAR7_35039554 |
| 7 | 35030356 | 35039554 | 21 | 9199 | oar3_OAR7_35030356 | oar3_OAR7_35039554 |
| 7 | 35034280 | 35047065 | 14 | 12786 | oar3_OAR7_35034280 | oar3_OAR7_35047065 |
| 7 | 35034940 | 35037811 | 10 | 2872 | oar3_OAR7_35034940 | oar3_OAR7_35037811 |
| 7 | 42322028 | 42342100 | 7 | 20073 | oar3_OAR7_42322028 | oar3_OAR7_42342100 |
| 7 | 62184546 | 62300818 | 26 | 116273 | oar3_OAR7_62184546 | oar3_OAR7_62300818 |
| 7 | 62184546 | 62300818 | 26 | 116273 | oar3_OAR7_62184546 | oar3_OAR7_62300818 |
| 7 | 62291817 | 62300818 | 5 | 9002 | oar3_OAR7_62291817 | oar3_OAR7_62300818 |
| 7 | 77509367 | 77547656 | 9 | 38290 | oar3_OAR7_77509367 | oar3_OAR7_77547656 |
| 7 | 77512772 | 77579014 | 17 | 66243 | oar3_OAR7_77512772 | oar3_OAR7_77579014 |
| 7 | 77620277 | 77643107 | 9 | 22831 | oar3_OAR7_77620277 | oar3_OAR7_77643107 |
| 7 | 81610788 | 81649901 | 14 | 39114 | oar3_OAR7_81610788 | oar3_OAR7_81649901 |
| 7 | 81633018 | 81649901 | 10 | 16884 | oar3_OAR7_81633018 | oar3_OAR7_81649901 |
| 7 | 81637706 | 81646443 | 7 | 8738 | oar3_OAR7_81637706 | oar3_OAR7_81646443 |
| 7 | 81937477 | 82000777 | 17 | 63301 | oar3_OAR7_81937477 | oar3_OAR7_82000777 |
| 7 | 81962439 | 82000777 | 12 | 38339 | oar3_OAR7_81962439 | oar3_OAR7_82000777 |
| 7 | 82708460 | 82721286 | 7 | 12827 | oar3_OAR7_82708460 | oar3_OAR7_82721286 |
| 7 | 83047639 | 83125319 | 22 | 77681 | oar3_OAR7_83047639 | s22606.1 |
| 7 | 83065416 | 83125319 | 14 | 59904 | oar3_OAR7_83065416 | s22606.1 |
| 7 | 83078354 | 83128721 | 12 | 50368 | oar3_OAR7_83078354 | oar3_OAR7_83128721 |
| 7 | 84637144 | 84738474 | 32 | 101331 | oar3_OAR7_84637144 | oar3_OAR7_84738474 |
| 7 | 84676824 | 84726064 | 18 | 49241 | oar3_OAR7_84676824 | oar3_OAR7_84726064 |
| 7 | 84693413 | 84738474 | 15 | 45062 | s41241.1 | oar3_OAR7_84738474 |
| 7 | 84723005 | 84726064 | 3 | 3060 | oar3_OAR7_84723005 | oar3_OAR7_84726064 |
| 7 | 85367675 | 85396788 | 9 | 29114 | oar3_OAR7_85367675 | oar3_OAR7_85396788 |
| 7 | 85367675 | 85396788 | 9 | 29114 | oar3_OAR7_85367675 | oar3_OAR7_85396788 |
| 7 | 85622496 | 85623350 | 4 | 855 | oar3_OAR7_85622496 | oar3_OAR7_85623350 |
| 7 | 85622496 | 85623350 | 4 | 855 | oar3_OAR7_85622496 | oar3_OAR7_85623350 |
| 7 | 85622496 | 85636694 | 6 | 14199 | oar3_OAR7_85622496 | oar3_OAR7_85636694 |
| 7 | 85622496 | 85623350 | 4 | 855 | oar3_OAR7_85622496 | oar3_OAR7_85623350 |
| 7 | 85622496 | 85636694 | 6 | 14199 | oar3_OAR7_85622496 | oar3_OAR7_85636694 |
| 7 | 85622496 | 85623350 | 4 | 855 | oar3_OAR7_85622496 | oar3_OAR7_85623350 |
| 7 | 85622496 | 85636694 | 6 | 14199 | oar3_OAR7_85622496 | oar3_OAR7_85636694 |
| 7 | 91257932 | 91261844 | 4 | 3913 | oar3_OAR7_91257932 | oar3_OAR7_91261844 |
| 7 | 97036239 | 97062299 | 9 | 26061 | oar3_OAR7_97036239 | oar3_OAR7_97062299 |
| 7 | 97038976 | 97062299 | 8 | 23324 | oar3_OAR7_97038976 | oar3_OAR7_97062299 |
| 7 | 98502328 | 98610873 | 38 | 108546 | oar3_OAR7_98502328 | oar3_OAR7_98610873 |
| 7 | 98699532 | 98711465 | 4 | 11934 | oar3_OAR7_98699532 | oar3_OAR7_98711465 |
| 7 | 98754548 | 98767294 | 5 | 12747 | oar3_OAR7_98754548 | oar3_OAR7_98767294 |
| 7 | 98754548 | 98767294 | 5 | 12747 | oar3_OAR7_98754548 | oar3_OAR7_98767294 |
| 7 | 98953848 | 99101517 | 30 | 147670 | oar3_OAR7_98953848 | oar3_OAR7_99101517 |
| 7 | 99044010 | 99113674 | 18 | 69665 | s03853.1 | oar3_OAR7_99113674 |
| 7 | 99154920 | 99304722 | 39 | 149803 | oar3_OAR7_99154920 | oar3_OAR7_99304722 |
| 7 | 99154920 | 99181489 | 4 | 26570 | oar3_OAR7_99154920 | s20973.1 |
| 7 | 99210191 | 99260462 | 12 | 50272 | oar3_OAR7_99210191 | oar3_OAR7_99260462 |
| 7 | 99486980 | 99543061 | 15 | 56082 | oar3_OAR7_99486980 | oar3_OAR7_99543061 |
| 8 | 2208100 | 2437064 | 24 | 228965 | oar3_OAR8_2208100 | oar3_OAR8_2437064 |
| 8 | 2222170 | 2418546 | 20 | 196377 | oar3_OAR8_2222170 | oar3_OAR8_2418546 |
| 8 | 2222170 | 2418546 | 20 | 196377 | oar3_OAR8_2222170 | oar3_OAR8_2418546 |
| 8 | 2222170 | 2418546 | 20 | 196377 | oar3_OAR8_2222170 | oar3_OAR8_2418546 |
| 8 | 2222170 | 2418546 | 20 | 196377 | oar3_OAR8_2222170 | oar3_OAR8_2418546 |
| 8 | 2229149 | 2268393 | 3 | 39245 | oar3_OAR8_2229149 | oar3_OAR8_2268393 |
| 8 | 2229149 | 2418546 | 19 | 189398 | oar3_OAR8_2229149 | oar3_OAR8_2418546 |
| 8 | 2245361 | 2418546 | 18 | 173186 | oar3_OAR8_2245361 | oar3_OAR8_2418546 |
| 8 | 2268393 | 2418546 | 17 | 150154 | oar3_OAR8_2268393 | oar3_OAR8_2418546 |
| 8 | 2268393 | 2418546 | 17 | 150154 | oar3_OAR8_2268393 | oar3_OAR8_2418546 |
| 8 | 2356165 | 2387275 | 7 | 31111 | oar3_OAR8_2356165 | oar3_OAR8_2387275 |
| 8 | 16912966 | 16932947 | 5 | 19982 | oar3_OAR8_16912966 | oar3_OAR8_16932947 |
| 8 | 26130497 | 26152510 | 10 | 22014 | oar3_OAR8_26130497 | oar3_OAR8_26152510 |
| 8 | 26957330 | 26971077 | 4 | 13748 | oar3_OAR8_26957330 | oar3_OAR8_26971077 |
| 8 | 37294450 | 37308253 | 5 | 13804 | oar3_OAR8_37294450 | oar3_OAR8_37308253 |
| 8 | 68518284 | 68525551 | 8 | 7268 | oar3_OAR8_68518284 | oar3_OAR8_68525551 |
| 8 | 68518284 | 68525551 | 8 | 7268 | oar3_OAR8_68518284 | oar3_OAR8_68525551 |
| 8 | 82111696 | 82119289 | 5 | 7594 | s41250.1 | s73785.1 |
| 8 | 82181191 | 82185911 | 9 | 4721 | oar3_OAR8_82181191 | oar3_OAR8_82185911 |
| 8 | 82741966 | 82749657 | 5 | 7692 | oar3_OAR8_82741966 | oar3_OAR8_82749657 |
| 8 | 84376133 | 84403873 | 6 | 27741 | oar3_OAR8_84376133 | oar3_OAR8_84403873 |
| 8 | 84512852 | 84516989 | 7 | 4138 | oar3_OAR8_84512852 | s32108.1 |
| 8 | 84587878 | 84665808 | 17 | 77931 | oar3_OAR8_84587878 | oar3_OAR8_84665808 |
| 8 | 85375089 | 85399203 | 9 | 24115 | s18231.1 | oar3_OAR8_85399203 |
| 8 | 85375089 | 85395977 | 8 | 20889 | s18231.1 | oar3_OAR8_85395977 |
| 8 | 85375089 | 85395977 | 8 | 20889 | s18231.1 | oar3_OAR8_85395977 |
| 8 | 85375582 | 85401256 | 8 | 25675 | oar3_OAR8_85375582 | oar3_OAR8_85401256 |
| 8 | 85740759 | 85752685 | 4 | 11927 | s42386.1 | oar3_OAR8_85752685 |
| 8 | 87784921 | 87927291 | 23 | 142371 | oar3_OAR8_87784921 | oar3_OAR8_87927291 |
| 8 | 88105733 | 88110888 | 5 | 5156 | oar3_OAR8_88105733 | OAR8_94906557.1 |
| 8 | 88105733 | 88148657 | 20 | 42925 | oar3_OAR8_88105733 | oar3_OAR8_88148657 |
| 8 | 88105733 | 88148657 | 20 | 42925 | oar3_OAR8_88105733 | oar3_OAR8_88148657 |
| 8 | 88452264 | 88504997 | 13 | 52734 | OAR8_95443730.1 | oar3_OAR8_88504997 |
| 8 | 88483637 | 89549115 | 278 | 1065479 | oar3_OAR8_88483637 | oar3_OAR8_89549115 |
| 8 | 88561673 | 88601461 | 20 | 39789 | oar3_OAR8_88561673 | oar3_OAR8_88601461 |
| 8 | 88614121 | 88625060 | 7 | 10940 | oar3_OAR8_88614121 | oar3_OAR8_88625060 |
| 8 | 88819528 | 88996463 | 36 | 176936 | oar3_OAR8_88819528 | oar3_OAR8_88996463 |
| 8 | 88888309 | 88928046 | 9 | 39738 | oar3_OAR8_88888309 | s31877.1 |
| 8 | 89040079 | 89113594 | 30 | 73516 | oar3_OAR8_89040079 | oar3_OAR8_89113594 |
| 8 | 89047092 | 89101802 | 24 | 54711 | oar3_OAR8_89047092 | oar3_OAR8_89101802 |
| 8 | 89047092 | 89086179 | 17 | 39088 | oar3_OAR8_89047092 | oar3_OAR8_89086179 |
| 8 | 89052144 | 89085069 | 13 | 32926 | oar3_OAR8_89052144 | oar3_OAR8_89085069 |
| 8 | 89085069 | 89300931 | 47 | 215863 | oar3_OAR8_89085069 | oar3_OAR8_89300931 |
| 8 | 89122646 | 89300931 | 29 | 178286 | oar3_OAR8_89122646 | oar3_OAR8_89300931 |
| 8 | 89131246 | 89384534 | 57 | 253289 | oar3_OAR8_89131246 | oar3_OAR8_89384534 |
| 8 | 89156227 | 89183987 | 6 | 27761 | oar3_OAR8_89156227 | oar3_OAR8_89183987 |
| 8 | 89156227 | 89300931 | 24 | 144705 | oar3_OAR8_89156227 | oar3_OAR8_89300931 |
| 8 | 89156227 | 89202991 | 9 | 46765 | oar3_OAR8_89156227 | oar3_OAR8_89202991 |
| 8 | 89156227 | 89300931 | 24 | 144705 | oar3_OAR8_89156227 | oar3_OAR8_89300931 |
| 8 | 89179291 | 89300931 | 22 | 121641 | oar3_OAR8_89179291 | oar3_OAR8_89300931 |
| 8 | 89179291 | 89300931 | 22 | 121641 | oar3_OAR8_89179291 | oar3_OAR8_89300931 |
| 8 | 89241137 | 89378314 | 33 | 137178 | oar3_OAR8_89241137 | oar3_OAR8_89378314 |
| 8 | 89341556 | 89379235 | 15 | 37680 | oar3_OAR8_89341556 | oar3_OAR8_89379235 |
| 8 | 89374608 | 89384534 | 6 | 9927 | oar3_OAR8_89374608 | oar3_OAR8_89384534 |
| 8 | 89503515 | 89549115 | 9 | 45601 | oar3_OAR8_89503515 | oar3_OAR8_89549115 |
| 8 | 89533869 | 89549115 | 5 | 15247 | oar3_OAR8_89533869 | oar3_OAR8_89549115 |
| 8 | 89673472 | 89675851 | 4 | 2380 | oar3_OAR8_89673472 | oar3_OAR8_89675851 |
| 8 | 89750290 | 89778556 | 9 | 28267 | oar3_OAR8_89750290 | s41332.1 |
| 8 | 89750290 | 90102642 | 78 | 352353 | oar3_OAR8_89750290 | oar3_OAR8_90102642 |
| 8 | 89750290 | 89798851 | 12 | 48562 | oar3_OAR8_89750290 | s49893.1 |
| 8 | 89833205 | 90016455 | 38 | 183251 | oar3_OAR8_89833205 | oar3_OAR8_90016455 |
| 8 | 89860881 | 89977104 | 26 | 116224 | oar3_OAR8_89860881 | oar3_OAR8_89977104 |
| 8 | 89884968 | 90085293 | 49 | 200326 | oar3_OAR8_89884968 | oar3_OAR8_90085293 |
| 8 | 89884968 | 90016455 | 28 | 131488 | oar3_OAR8_89884968 | oar3_OAR8_90016455 |
| 8 | 89904509 | 90102642 | 47 | 198134 | oar3_OAR8_89904509 | oar3_OAR8_90102642 |
| 8 | 89907877 | 90016455 | 22 | 108579 | oar3_OAR8_89907877 | oar3_OAR8_90016455 |
| 8 | 89907877 | 89933901 | 9 | 26025 | oar3_OAR8_89907877 | oar3_OAR8_89933901 |
| 8 | 89975619 | 90102642 | 34 | 127024 | oar3_OAR8_89975619 | oar3_OAR8_90102642 |
| 8 | 90006665 | 90016455 | 5 | 9791 | oar3_OAR8_90006665 | oar3_OAR8_90016455 |
| 8 | 90053928 | 90085293 | 16 | 31366 | OAR8_97126899.1 | oar3_OAR8_90085293 |
| 8 | 90165244 | 90630803 | 69 | 465560 | oar3_OAR8_90165244 | oar3_OAR8_90630803 |
| 8 | 90190726 | 90525479 | 46 | 334754 | oar3_OAR8_90190726 | oar3_OAR8_90525479 |
| 8 | 90220060 | 90225862 | 4 | 5803 | oar3_OAR8_90220060 | oar3_OAR8_90225862 |
| 8 | 90220060 | 90416289 | 30 | 196230 | oar3_OAR8_90220060 | oar3_OAR8_90416289 |
| 8 | 90220060 | 90240785 | 8 | 20726 | oar3_OAR8_90220060 | oar3_OAR8_90240785 |
| 8 | 90220060 | 90389627 | 28 | 169568 | oar3_OAR8_90220060 | oar3_OAR8_90389627 |
| 8 | 90220060 | 90416289 | 30 | 196230 | oar3_OAR8_90220060 | oar3_OAR8_90416289 |
| 8 | 90225509 | 90225862 | 3 | 354 | oar3_OAR8_90225509 | oar3_OAR8_90225862 |
| 8 | 90240785 | 90301876 | 9 | 61092 | oar3_OAR8_90240785 | oar3_OAR8_90301876 |
| 8 | 90240785 | 90630803 | 55 | 390019 | oar3_OAR8_90240785 | oar3_OAR8_90630803 |
| 8 | 90281832 | 90389627 | 17 | 107796 | oar3_OAR8_90281832 | oar3_OAR8_90389627 |
| 8 | 90365157 | 90416289 | 6 | 51133 | oar3_OAR8_90365157 | oar3_OAR8_90416289 |
| 8 | 90584882 | 90630803 | 8 | 45922 | oar3_OAR8_90584882 | oar3_OAR8_90630803 |
| 9 | 9321384 | 9335875 | 4 | 14492 | oar3_OAR9_9321384 | oar3_OAR9_9335875 |
| 9 | 12042743 | 12118904 | 17 | 76162 | oar3_OAR9_12042743 | oar3_OAR9_12118904 |
| 9 | 13197375 | 13228772 | 10 | 31398 | oar3_OAR9_13197375 | oar3_OAR9_13228772 |
| 9 | 13199744 | 13228772 | 9 | 29029 | s21274.1 | oar3_OAR9_13228772 |
| 9 | 13296806 | 13389619 | 16 | 92814 | oar3_OAR9_13296806 | oar3_OAR9_13389619 |
| 9 | 13296806 | 13339063 | 8 | 42258 | oar3_OAR9_13296806 | oar3_OAR9_13339063 |
| 9 | 13351715 | 13640820 | 40 | 289106 | s36767.1 | oar3_OAR9_13640820 |
| 9 | 13371655 | 13469179 | 20 | 97525 | oar3_OAR9_13371655 | oar3_OAR9_13469179 |
| 9 | 13380701 | 13613727 | 31 | 233027 | oar3_OAR9_13380701 | oar3_OAR9_13613727 |
| 9 | 13388911 | 13632637 | 34 | 243727 | oar3_OAR9_13388911 | oar3_OAR9_13632637 |
| 9 | 13416452 | 13469179 | 14 | 52728 | oar3_OAR9_13416452 | oar3_OAR9_13469179 |
| 9 | 13428352 | 13613727 | 24 | 185376 | oar3_OAR9_13428352 | oar3_OAR9_13613727 |
| 9 | 13428352 | 13449896 | 7 | 21545 | oar3_OAR9_13428352 | oar3_OAR9_13449896 |
| 9 | 13428352 | 13449896 | 7 | 21545 | oar3_OAR9_13428352 | oar3_OAR9_13449896 |
| 9 | 13428352 | 13449617 | 6 | 21266 | oar3_OAR9_13428352 | oar3_OAR9_13449617 |
| 9 | 13445596 | 13676344 | 31 | 230749 | oar3_OAR9_13445596 | oar3_OAR9_13676344 |
| 9 | 13445596 | 13469179 | 8 | 23584 | oar3_OAR9_13445596 | oar3_OAR9_13469179 |
| 9 | 13623805 | 13702237 | 14 | 78433 | oar3_OAR9_13623805 | oar3_OAR9_13702237 |
| 9 | 13773895 | 13846026 | 7 | 72132 | oar3_OAR9_13773895 | oar3_OAR9_13846026 |
| 9 | 13773895 | 13895976 | 19 | 122082 | oar3_OAR9_13773895 | oar3_OAR9_13895976 |
| 9 | 13773895 | 13846026 | 7 | 72132 | oar3_OAR9_13773895 | oar3_OAR9_13846026 |
| 9 | 13832425 | 13848339 | 4 | 15915 | oar3_OAR9_13832425 | oar3_OAR9_13848339 |
| 9 | 13869098 | 13985953 | 23 | 116856 | oar3_OAR9_13869098 | oar3_OAR9_13985953 |
| 9 | 13869098 | 14101405 | 44 | 232308 | oar3_OAR9_13869098 | oar3_OAR9_14101405 |
| 9 | 13888048 | 13936940 | 10 | 48893 | oar3_OAR9_13888048 | oar3_OAR9_13936940 |
| 9 | 13888048 | 13945946 | 11 | 57899 | oar3_OAR9_13888048 | oar3_OAR9_13945946 |
| 9 | 13888048 | 13893491 | 5 | 5444 | oar3_OAR9_13888048 | oar3_OAR9_13893491 |
| 9 | 13888048 | 13893491 | 5 | 5444 | oar3_OAR9_13888048 | oar3_OAR9_13893491 |
| 9 | 13888048 | 14000208 | 23 | 112161 | oar3_OAR9_13888048 | oar3_OAR9_14000208 |
| 9 | 13945946 | 13981116 | 9 | 35171 | oar3_OAR9_13945946 | oar3_OAR9_13981116 |
| 9 | 13950581 | 13991690 | 11 | 41110 | oar3_OAR9_13950581 | oar3_OAR9_13991690 |
| 9 | 13977094 | 13991690 | 6 | 14597 | oar3_OAR9_13977094 | oar3_OAR9_13991690 |
| 9 | 14034392 | 14101405 | 13 | 67014 | oar3_OAR9_14034392 | oar3_OAR9_14101405 |
| 9 | 14034392 | 14135544 | 21 | 101153 | oar3_OAR9_14034392 | oar3_OAR9_14135544 |
| 9 | 14034392 | 14090126 | 9 | 55735 | oar3_OAR9_14034392 | oar3_OAR9_14090126 |
| 9 | 14034392 | 14217161 | 36 | 182770 | oar3_OAR9_14034392 | oar3_OAR9_14217161 |
| 9 | 14034392 | 14135544 | 21 | 101153 | oar3_OAR9_14034392 | oar3_OAR9_14135544 |
| 9 | 14034392 | 14214698 | 35 | 180307 | oar3_OAR9_14034392 | oar3_OAR9_14214698 |
| 9 | 14042355 | 14240556 | 39 | 198202 | s52013.1 | oar3_OAR9_14240556 |
| 9 | 14063256 | 14274332 | 49 | 211077 | oar3_OAR9_14063256 | oar3_OAR9_14274332 |
| 9 | 14063256 | 14217161 | 33 | 153906 | oar3_OAR9_14063256 | oar3_OAR9_14217161 |
| 9 | 14112212 | 14244852 | 28 | 132641 | oar3_OAR9_14112212 | oar3_OAR9_14244852 |
| 9 | 14173592 | 14250061 | 20 | 76470 | oar3_OAR9_14173592 | oar3_OAR9_14250061 |
| 9 | 14181680 | 14250061 | 19 | 68382 | oar3_OAR9_14181680 | oar3_OAR9_14250061 |
| 9 | 14209909 | 14303907 | 30 | 93999 | oar3_OAR9_14209909 | OAR9_14653377.1 |
| 9 | 14229348 | 14302684 | 23 | 73337 | oar3_OAR9_14229348 | oar3_OAR9_14302684 |
| 9 | 14264804 | 14301493 | 12 | 36690 | oar3_OAR9_14264804 | oar3_OAR9_14301493 |
| 9 | 14264804 | 14302684 | 14 | 37881 | oar3_OAR9_14264804 | oar3_OAR9_14302684 |
| 9 | 14272267 | 14303156 | 12 | 30890 | s69711.1 | oar3_OAR9_14303156 |
| 9 | 14296802 | 14303907 | 7 | 7106 | oar3_OAR9_14296802 | OAR9_14653377.1 |
| 9 | 14296802 | 14303907 | 7 | 7106 | oar3_OAR9_14296802 | OAR9_14653377.1 |
| 9 | 14454312 | 14753674 | 54 | 299363 | oar3_OAR9_14454312 | oar3_OAR9_14753674 |
| 9 | 14478807 | 14499979 | 12 | 21173 | oar3_OAR9_14478807 | oar3_OAR9_14499979 |
| 9 | 14478807 | 14499979 | 12 | 21173 | oar3_OAR9_14478807 | oar3_OAR9_14499979 |
| 9 | 14479964 | 14630106 | 29 | 150143 | oar3_OAR9_14479964 | oar3_OAR9_14630106 |
| 9 | 14488304 | 14592115 | 20 | 103812 | oar3_OAR9_14488304 | oar3_OAR9_14592115 |
| 9 | 14529698 | 14592115 | 9 | 62418 | oar3_OAR9_14529698 | oar3_OAR9_14592115 |
| 9 | 14574678 | 14630106 | 8 | 55429 | oar3_OAR9_14574678 | oar3_OAR9_14630106 |
| 9 | 14574678 | 14659489 | 13 | 84812 | oar3_OAR9_14574678 | oar3_OAR9_14659489 |
| 9 | 14617163 | 14649079 | 6 | 31917 | oar3_OAR9_14617163 | oar3_OAR9_14649079 |
| 9 | 14643564 | 14711331 | 13 | 67768 | oar3_OAR9_14643564 | oar3_OAR9_14711331 |
| 9 | 14643564 | 14649079 | 4 | 5516 | oar3_OAR9_14643564 | oar3_OAR9_14649079 |
| 9 | 14659489 | 14836433 | 41 | 176945 | oar3_OAR9_14659489 | oar3_OAR9_14836433 |
| 9 | 14675654 | 14759561 | 18 | 83908 | oar3_OAR9_14675654 | oar3_OAR9_14759561 |
| 9 | 14685884 | 14728164 | 7 | 42281 | oar3_OAR9_14685884 | oar3_OAR9_14728164 |
| 9 | 14776804 | 14836433 | 18 | 59630 | oar3_OAR9_14776804 | oar3_OAR9_14836433 |
| 9 | 14796015 | 14841067 | 17 | 45053 | oar3_OAR9_14796015 | oar3_OAR9_14841067 |
| 9 | 14797444 | 14857997 | 19 | 60554 | oar3_OAR9_14797444 | oar3_OAR9_14857997 |
| 9 | 14797444 | 14841067 | 14 | 43624 | oar3_OAR9_14797444 | oar3_OAR9_14841067 |
| 9 | 14797444 | 14811773 | 6 | 14330 | oar3_OAR9_14797444 | oar3_OAR9_14811773 |
| 9 | 14817127 | 14841067 | 7 | 23941 | oar3_OAR9_14817127 | oar3_OAR9_14841067 |
| 9 | 14817127 | 14963594 | 35 | 146468 | oar3_OAR9_14817127 | oar3_OAR9_14963594 |
| 9 | 14818910 | 14882990 | 13 | 64081 | oar3_OAR9_14818910 | oar3_OAR9_14882990 |
| 9 | 14841067 | 14895823 | 9 | 54757 | oar3_OAR9_14841067 | oar3_OAR9_14895823 |
| 9 | 14841067 | 14920053 | 15 | 78987 | oar3_OAR9_14841067 | oar3_OAR9_14920053 |
| 9 | 14951176 | 15032534 | 23 | 81359 | oar3_OAR9_14951176 | oar3_OAR9_15032534 |
| 9 | 15183574 | 15245885 | 22 | 62312 | oar3_OAR9_15183574 | s46107.1 |
| 9 | 15311128 | 15524377 | 46 | 213250 | oar3_OAR9_15311128 | oar3_OAR9_15524377 |
| 9 | 15329806 | 15526453 | 41 | 196648 | oar3_OAR9_15329806 | oar3_OAR9_15526453 |
| 9 | 15355050 | 15543361 | 39 | 188312 | oar3_OAR9_15355050 | oar3_OAR9_15543361 |
| 9 | 15373923 | 15400018 | 7 | 26096 | oar3_OAR9_15373923 | s27671.1 |
| 9 | 15390039 | 15470065 | 20 | 80027 | oar3_OAR9_15390039 | oar3_OAR9_15470065 |
| 9 | 15418969 | 15526453 | 24 | 107485 | oar3_OAR9_15418969 | oar3_OAR9_15526453 |
| 9 | 15429600 | 15524377 | 20 | 94778 | oar3_OAR9_15429600 | oar3_OAR9_15524377 |
| 9 | 15460797 | 15598346 | 28 | 137550 | oar3_OAR9_15460797 | oar3_OAR9_15598346 |
| 9 | 15467431 | 15494389 | 6 | 26959 | oar3_OAR9_15467431 | oar3_OAR9_15494389 |
| 9 | 15488634 | 15543361 | 14 | 54728 | oar3_OAR9_15488634 | oar3_OAR9_15543361 |
| 9 | 15522693 | 15598346 | 14 | 75654 | oar3_OAR9_15522693 | oar3_OAR9_15598346 |
| 9 | 15569254 | 15598346 | 7 | 29093 | oar3_OAR9_15569254 | oar3_OAR9_15598346 |
| 9 | 15589845 | 15598346 | 5 | 8502 | oar3_OAR9_15589845 | oar3_OAR9_15598346 |
| 9 | 15589845 | 15593842 | 3 | 3998 | oar3_OAR9_15589845 | oar3_OAR9_15593842 |
| 9 | 15858013 | 15943473 | 20 | 85461 | oar3_OAR9_15858013 | oar3_OAR9_15943473 |
| 9 | 15896621 | 16007813 | 25 | 111193 | oar3_OAR9_15896621 | OAR9_16457521.1 |
| 9 | 15922153 | 16007813 | 18 | 85661 | oar3_OAR9_15922153 | OAR9_16457521.1 |
| 9 | 16328178 | 16416198 | 26 | 88021 | oar3_OAR9_16328178 | oar3_OAR9_16416198 |
| 9 | 16357134 | 16379159 | 9 | 22026 | oar3_OAR9_16357134 | oar3_OAR9_16379159 |
| 9 | 16357134 | 16402125 | 17 | 44992 | oar3_OAR9_16357134 | oar3_OAR9_16402125 |
| 9 | 16375749 | 16416198 | 14 | 40450 | oar3_OAR9_16375749 | oar3_OAR9_16416198 |
| 9 | 16385897 | 16416198 | 10 | 30302 | oar3_OAR9_16385897 | oar3_OAR9_16416198 |
| 9 | 16390410 | 16441079 | 15 | 50670 | oar3_OAR9_16390410 | oar3_OAR9_16441079 |
| 9 | 17138420 | 17193461 | 25 | 55042 | oar3_OAR9_17138420 | oar3_OAR9_17193461 |
| 9 | 17166343 | 17193461 | 13 | 27119 | oar3_OAR9_17166343 | oar3_OAR9_17193461 |
| 9 | 17173676 | 17193461 | 12 | 19786 | s04789.1 | oar3_OAR9_17193461 |
| 9 | 17173676 | 17185372 | 8 | 11697 | s04789.1 | oar3_OAR9_17185372 |
| 9 | 17173676 | 17193461 | 12 | 19786 | s04789.1 | oar3_OAR9_17193461 |
| 9 | 17173676 | 17185372 | 8 | 11697 | s04789.1 | oar3_OAR9_17185372 |
| 9 | 17176669 | 17193461 | 11 | 16793 | oar3_OAR9_17176669 | oar3_OAR9_17193461 |
| 9 | 17177932 | 17185372 | 6 | 7441 | oar3_OAR9_17177932 | oar3_OAR9_17185372 |
| 9 | 17179180 | 17185372 | 5 | 6193 | oar3_OAR9_17179180 | oar3_OAR9_17185372 |
| 9 | 17182413 | 17193461 | 7 | 11049 | oar3_OAR9_17182413 | oar3_OAR9_17193461 |
| 9 | 17182413 | 17185372 | 3 | 2960 | oar3_OAR9_17182413 | oar3_OAR9_17185372 |
| 9 | 20088642 | 20185465 | 19 | 96824 | oar3_OAR9_20088642 | oar3_OAR9_20185465 |
| 9 | 20906488 | 20938830 | 10 | 32343 | oar3_OAR9_20906488 | oar3_OAR9_20938830 |
| 9 | 20906488 | 20934755 | 9 | 28268 | oar3_OAR9_20906488 | oar3_OAR9_20934755 |
| 9 | 20907507 | 20938830 | 9 | 31324 | oar3_OAR9_20907507 | oar3_OAR9_20938830 |
| 9 | 32279972 | 32446863 | 55 | 166892 | oar3_OAR9_32279972 | oar3_OAR9_32446863 |
| 9 | 32502488 | 32505005 | 4 | 2518 | oar3_OAR9_32502488 | oar3_OAR9_32505005 |
| 9 | 32547728 | 32560297 | 5 | 12570 | oar3_OAR9_32547728 | oar3_OAR9_32560297 |
| 9 | 62835617 | 62843078 | 3 | 7462 | oar3_OAR9_62835617 | oar3_OAR9_62843078 |
| 9 | 65690514 | 65716043 | 9 | 25530 | oar3_OAR9_65690514 | oar3_OAR9_65716043 |
| 9 | 71274282 | 71293588 | 5 | 19307 | oar3_OAR9_71274282 | OAR9_75687828.1 |
| 9 | 75858747 | 75878130 | 7 | 19384 | oar3_OAR9_75858747 | oar3_OAR9_75878130 |
| 9 | 75858747 | 75860948 | 4 | 2202 | oar3_OAR9_75858747 | oar3_OAR9_75860948 |
| 9 | 75858747 | 75878130 | 7 | 19384 | oar3_OAR9_75858747 | oar3_OAR9_75878130 |
| 9 | 75858747 | 75878130 | 7 | 19384 | oar3_OAR9_75858747 | oar3_OAR9_75878130 |
| 9 | 75858747 | 75878130 | 7 | 19384 | oar3_OAR9_75858747 | oar3_OAR9_75878130 |
| 9 | 75858747 | 75860948 | 4 | 2202 | oar3_OAR9_75858747 | oar3_OAR9_75860948 |
| 9 | 75858747 | 75860948 | 4 | 2202 | oar3_OAR9_75858747 | oar3_OAR9_75860948 |
| 9 | 75858747 | 75860948 | 4 | 2202 | oar3_OAR9_75858747 | oar3_OAR9_75860948 |
| 9 | 77377746 | 77430104 | 8 | 52359 | OAR9_81995689.1 | oar3_OAR9_77430104 |
| 9 | 78645950 | 78677840 | 15 | 31891 | oar3_OAR9_78645950 | s03389.1 |
| 10 | 4351868 | 4406293 | 15 | 54426 | oar3_OAR10_4351868 | oar3_OAR10_4406293 |
| 10 | 7786490 | 7884154 | 10 | 97665 | oar3_OAR10_7786490 | oar3_OAR10_7884154 |
| 10 | 8051493 | 8064515 | 3 | 13023 | oar3_OAR10_8051493 | oar3_OAR10_8064515 |
| 10 | 10834077 | 10839183 | 5 | 5107 | oar3_OAR10_10834077 | oar3_OAR10_10839183 |
| 10 | 12658600 | 12658732 | 5 | 133 | oar3_OAR10_12658600 | oar3_OAR10_12658732 |
| 10 | 13203562 | 13205433 | 3 | 1872 | oar3_OAR10_13203562 | oar3_OAR10_13205433 |
| 10 | 15008992 | 15010988 | 4 | 1997 | oar3_OAR10_15008992 | oar3_OAR10_15010988 |
| 10 | 15008992 | 15010988 | 4 | 1997 | oar3_OAR10_15008992 | oar3_OAR10_15010988 |
| 10 | 16661706 | 16665002 | 4 | 3297 | oar3_OAR10_16661706 | oar3_OAR10_16665002 |
| 10 | 30601697 | 30645132 | 19 | 43436 | oar3_OAR10_30601697 | oar3_OAR10_30645132 |
| 10 | 31224788 | 31256672 | 16 | 31885 | oar3_OAR10_31224788 | oar3_OAR10_31256672 |
| 10 | 32300354 | 32335528 | 10 | 35175 | oar3_OAR10_32300354 | oar3_OAR10_32335528 |
| 10 | 33124953 | 33144585 | 5 | 19633 | s26846.1 | oar3_OAR10_33144585 |
| 10 | 33124953 | 33150871 | 6 | 25919 | s26846.1 | oar3_OAR10_33150871 |
| 10 | 33599410 | 33626776 | 8 | 27367 | oar3_OAR10_33599410 | oar3_OAR10_33626776 |
| 10 | 34408109 | 34556874 | 41 | 148766 | oar3_OAR10_34408109 | oar3_OAR10_34556874 |
| 10 | 34461325 | 34572481 | 30 | 111157 | oar3_OAR10_34461325 | oar3_OAR10_34572481 |
| 10 | 34856935 | 34870291 | 6 | 13357 | oar3_OAR10_34856935 | oar3_OAR10_34870291 |
| 10 | 35197500 | 35220486 | 8 | 22987 | oar3_OAR10_35197500 | oar3_OAR10_35220486 |
| 10 | 35838530 | 35885222 | 6 | 46693 | oar3_OAR10_35838530 | s22929.1 |
| 10 | 35851977 | 35885222 | 4 | 33246 | oar3_OAR10_35851977 | s22929.1 |
| 10 | 36911464 | 36959483 | 17 | 48020 | oar3_OAR10_36911464 | oar3_OAR10_36959483 |
| 10 | 36911464 | 36959483 | 17 | 48020 | oar3_OAR10_36911464 | oar3_OAR10_36959483 |
| 10 | 36911464 | 36959483 | 17 | 48020 | oar3_OAR10_36911464 | oar3_OAR10_36959483 |
| 10 | 36911464 | 36954426 | 16 | 42963 | oar3_OAR10_36911464 | oar3_OAR10_36954426 |
| 10 | 36911464 | 36959483 | 17 | 48020 | oar3_OAR10_36911464 | oar3_OAR10_36959483 |
| 10 | 36911464 | 36914457 | 5 | 2994 | oar3_OAR10_36911464 | oar3_OAR10_36914457 |
| 10 | 36930595 | 36959483 | 11 | 28889 | oar3_OAR10_36930595 | oar3_OAR10_36959483 |
| 10 | 44589070 | 44596003 | 4 | 6934 | oar3_OAR10_44589070 | OAR10_45180731.1 |
| 10 | 58675092 | 58687706 | 5 | 12615 | oar3_OAR10_58675092 | oar3_OAR10_58687706 |
| 10 | 63339130 | 63350196 | 5 | 11067 | oar3_OAR10_63339130 | oar3_OAR10_63350196 |
| 10 | 63339130 | 63350196 | 5 | 11067 | oar3_OAR10_63339130 | oar3_OAR10_63350196 |
| 10 | 70408753 | 70418977 | 3 | 10225 | oar3_OAR10_70408753 | oar3_OAR10_70418977 |
| 10 | 70552773 | 70567744 | 3 | 14972 | oar3_OAR10_70552773 | oar3_OAR10_70567744 |
| 10 | 70552773 | 70829630 | 8 | 276858 | oar3_OAR10_70552773 | oar3_OAR10_70829630 |
| 10 | 70552773 | 70567744 | 3 | 14972 | oar3_OAR10_70552773 | oar3_OAR10_70567744 |
| 10 | 70552773 | 70643700 | 4 | 90928 | oar3_OAR10_70552773 | oar3_OAR10_70643700 |
| 10 | 70552773 | 70921298 | 11 | 368526 | oar3_OAR10_70552773 | oar3_OAR10_70921298 |
| 10 | 70552773 | 70567744 | 3 | 14972 | oar3_OAR10_70552773 | oar3_OAR10_70567744 |
| 10 | 70558536 | 71192248 | 21 | 633713 | oar3_OAR10_70558536 | oar3_OAR10_71192248 |
| 10 | 70567744 | 70691775 | 4 | 124032 | oar3_OAR10_70567744 | oar3_OAR10_70691775 |
| 10 | 70809029 | 71280608 | 24 | 471580 | oar3_OAR10_70809029 | oar3_OAR10_71280608 |
| 10 | 70809029 | 71055395 | 10 | 246367 | oar3_OAR10_70809029 | oar3_OAR10_71055395 |
| 10 | 71049075 | 71399757 | 24 | 350683 | oar3_OAR10_71049075 | oar3_OAR10_71399757 |
| 10 | 71128030 | 71211465 | 10 | 83436 | oar3_OAR10_71128030 | oar3_OAR10_71211465 |
| 10 | 71167657 | 71192248 | 5 | 24592 | oar3_OAR10_71167657 | oar3_OAR10_71192248 |
| 10 | 71192100 | 71221008 | 7 | 28909 | oar3_OAR10_71192100 | oar3_OAR10_71221008 |
| 10 | 71192100 | 71367917 | 15 | 175818 | oar3_OAR10_71192100 | oar3_OAR10_71367917 |
| 10 | 71234043 | 71280608 | 3 | 46566 | oar3_OAR10_71234043 | oar3_OAR10_71280608 |
| 10 | 71364196 | 71399757 | 6 | 35562 | oar3_OAR10_71364196 | oar3_OAR10_71399757 |
| 10 | 71364196 | 71399757 | 6 | 35562 | oar3_OAR10_71364196 | oar3_OAR10_71399757 |
| 10 | 71708845 | 71776607 | 3 | 67763 | oar3_OAR10_71708845 | oar3_OAR10_71776607 |
| 10 | 71987850 | 72102214 | 11 | 114365 | oar3_OAR10_71987850 | oar3_OAR10_72102214 |
| 10 | 72062277 | 72102214 | 4 | 39938 | oar3_OAR10_72062277 | oar3_OAR10_72102214 |
| 10 | 72143155 | 72247452 | 11 | 104298 | oar3_OAR10_72143155 | oar3_OAR10_72247452 |
| 10 | 72143155 | 72247452 | 11 | 104298 | oar3_OAR10_72143155 | oar3_OAR10_72247452 |
| 10 | 72189690 | 72235010 | 5 | 45321 | oar3_OAR10_72189690 | oar3_OAR10_72235010 |
| 10 | 72189690 | 72247452 | 6 | 57763 | oar3_OAR10_72189690 | oar3_OAR10_72247452 |
| 10 | 72189690 | 72235010 | 5 | 45321 | oar3_OAR10_72189690 | oar3_OAR10_72235010 |
| 10 | 74783030 | 74803045 | 8 | 20016 | oar3_OAR10_74783030 | oar3_OAR10_74803045 |
| 10 | 74784345 | 74796442 | 4 | 12098 | oar3_OAR10_74784345 | oar3_OAR10_74796442 |
| 10 | 74784345 | 74799665 | 5 | 15321 | oar3_OAR10_74784345 | oar3_OAR10_74799665 |
| 10 | 74792413 | 74808621 | 8 | 16209 | oar3_OAR10_74792413 | oar3_OAR10_74808621 |
| 10 | 76064013 | 76077959 | 5 | 13947 | oar3_OAR10_76064013 | oar3_OAR10_76077959 |
| 10 | 76064013 | 76077959 | 5 | 13947 | oar3_OAR10_76064013 | oar3_OAR10_76077959 |
| 10 | 76513987 | 76534664 | 7 | 20678 | oar3_OAR10_76513987 | oar3_OAR10_76534664 |
| 10 | 84211472 | 84242227 | 7 | 30756 | oar3_OAR10_84211472 | oar3_OAR10_84242227 |
| 10 | 84463260 | 84486115 | 9 | 22856 | oar3_OAR10_84463260 | oar3_OAR10_84486115 |
| 10 | 84463260 | 84504835 | 16 | 41576 | oar3_OAR10_84463260 | oar3_OAR10_84504835 |
| 10 | 84463260 | 84508631 | 18 | 45372 | oar3_OAR10_84463260 | oar3_OAR10_84508631 |
| 10 | 84463260 | 84482379 | 8 | 19120 | oar3_OAR10_84463260 | oar3_OAR10_84482379 |
| 10 | 84463260 | 84504835 | 16 | 41576 | oar3_OAR10_84463260 | oar3_OAR10_84504835 |
| 10 | 84463260 | 84601611 | 36 | 138352 | oar3_OAR10_84463260 | oar3_OAR10_84601611 |
| 10 | 84575318 | 84617246 | 9 | 41929 | oar3_OAR10_84575318 | oar3_OAR10_84617246 |
| 10 | 84580697 | 84898582 | 74 | 317886 | oar3_OAR10_84580697 | OAR10_92625357.1 |
| 10 | 84764011 | 84797100 | 10 | 33090 | oar3_OAR10_84764011 | oar3_OAR10_84797100 |
| 10 | 84785540 | 84917190 | 38 | 131651 | oar3_OAR10_84785540 | oar3_OAR10_84917190 |
| 10 | 84797100 | 84832908 | 12 | 35809 | oar3_OAR10_84797100 | oar3_OAR10_84832908 |
| 10 | 84803804 | 84898582 | 31 | 94779 | oar3_OAR10_84803804 | OAR10_92625357.1 |
| 10 | 84805713 | 84986276 | 49 | 180564 | oar3_OAR10_84805713 | oar3_OAR10_84986276 |
| 10 | 84805713 | 84859208 | 19 | 53496 | oar3_OAR10_84805713 | oar3_OAR10_84859208 |
| 10 | 84817050 | 84842908 | 8 | 25859 | oar3_OAR10_84817050 | OAR10_92571454.1 |
| 10 | 84817050 | 84842908 | 8 | 25859 | oar3_OAR10_84817050 | OAR10_92571454.1 |
| 10 | 84930922 | 85022309 | 22 | 91388 | oar3_OAR10_84930922 | oar3_OAR10_85022309 |
| 10 | 84963626 | 85022309 | 13 | 58684 | oar3_OAR10_84963626 | oar3_OAR10_85022309 |
| 10 | 85079245 | 85146914 | 19 | 67670 | oar3_OAR10_85079245 | oar3_OAR10_85146914 |
| 10 | 85178675 | 85202201 | 14 | 23527 | oar3_OAR10_85178675 | oar3_OAR10_85202201 |
| 10 | 85178675 | 85202201 | 14 | 23527 | oar3_OAR10_85178675 | oar3_OAR10_85202201 |
| 10 | 85178675 | 85560968 | 103 | 382294 | oar3_OAR10_85178675 | oar3_OAR10_85560968 |
| 10 | 85178675 | 85218789 | 21 | 40115 | oar3_OAR10_85178675 | oar3_OAR10_85218789 |
| 10 | 85190514 | 85214395 | 9 | 23882 | oar3_OAR10_85190514 | oar3_OAR10_85214395 |
| 10 | 85195857 | 85202201 | 6 | 6345 | oar3_OAR10_85195857 | oar3_OAR10_85202201 |
| 10 | 85195857 | 85225122 | 14 | 29266 | oar3_OAR10_85195857 | oar3_OAR10_85225122 |
| 10 | 85295325 | 85313597 | 12 | 18273 | oar3_OAR10_85295325 | oar3_OAR10_85313597 |
| 10 | 85295325 | 85410011 | 33 | 114687 | oar3_OAR10_85295325 | oar3_OAR10_85410011 |
| 10 | 85300446 | 85543104 | 61 | 242659 | oar3_OAR10_85300446 | oar3_OAR10_85543104 |
| 10 | 85349922 | 85355728 | 4 | 5807 | oar3_OAR10_85349922 | oar3_OAR10_85355728 |
| 10 | 85349922 | 85355728 | 4 | 5807 | oar3_OAR10_85349922 | oar3_OAR10_85355728 |
| 10 | 85349922 | 85410011 | 17 | 60090 | oar3_OAR10_85349922 | oar3_OAR10_85410011 |
| 10 | 85349922 | 85355728 | 4 | 5807 | oar3_OAR10_85349922 | oar3_OAR10_85355728 |
| 10 | 85513474 | 85566962 | 16 | 53489 | oar3_OAR10_85513474 | oar3_OAR10_85566962 |
| 10 | 85641108 | 85896147 | 68 | 255040 | oar3_OAR10_85641108 | oar3_OAR10_85896147 |
| 10 | 85668292 | 85742117 | 25 | 73826 | oar3_OAR10_85668292 | oar3_OAR10_85742117 |
| 10 | 85689187 | 85742117 | 22 | 52931 | oar3_OAR10_85689187 | oar3_OAR10_85742117 |
| 10 | 85695176 | 85742117 | 17 | 46942 | s73474.1 | oar3_OAR10_85742117 |
| 10 | 85714981 | 85742117 | 11 | 27137 | oar3_OAR10_85714981 | oar3_OAR10_85742117 |
| 10 | 85714981 | 85742117 | 11 | 27137 | oar3_OAR10_85714981 | oar3_OAR10_85742117 |
| 10 | 85714981 | 85997986 | 71 | 283006 | oar3_OAR10_85714981 | oar3_OAR10_85997986 |
| 10 | 85793444 | 85834326 | 14 | 40883 | oar3_OAR10_85793444 | oar3_OAR10_85834326 |
| 10 | 85793444 | 85834326 | 14 | 40883 | oar3_OAR10_85793444 | oar3_OAR10_85834326 |
| 10 | 85800658 | 86439042 | 128 | 638385 | oar3_OAR10_85800658 | oar3_OAR10_86439042 |
| 10 | 85800658 | 85863284 | 18 | 62627 | oar3_OAR10_85800658 | oar3_OAR10_85863284 |
| 10 | 85824771 | 85834326 | 6 | 9556 | oar3_OAR10_85824771 | oar3_OAR10_85834326 |
| 10 | 85833628 | 85957057 | 29 | 123430 | oar3_OAR10_85833628 | oar3_OAR10_85957057 |
| 10 | 85921090 | 85997986 | 24 | 76897 | oar3_OAR10_85921090 | oar3_OAR10_85997986 |
| 10 | 85937102 | 85978913 | 13 | 41812 | oar3_OAR10_85937102 | oar3_OAR10_85978913 |
| 10 | 85937102 | 85988526 | 15 | 51425 | oar3_OAR10_85937102 | oar3_OAR10_85988526 |
| 10 | 85945048 | 85978913 | 11 | 33866 | s37825.1 | oar3_OAR10_85978913 |
| 10 | 85953233 | 85985388 | 11 | 32156 | oar3_OAR10_85953233 | oar3_OAR10_85985388 |
| 10 | 85974504 | 86001882 | 11 | 27379 | oar3_OAR10_85974504 | s74523.1 |
| 10 | 86044154 | 86159415 | 22 | 115262 | oar3_OAR10_86044154 | oar3_OAR10_86159415 |
| 10 | 86052854 | 86159415 | 20 | 106562 | oar3_OAR10_86052854 | oar3_OAR10_86159415 |
| 10 | 86087688 | 86159415 | 18 | 71728 | s75340.1 | oar3_OAR10_86159415 |
| 10 | 86087688 | 86159415 | 18 | 71728 | s75340.1 | oar3_OAR10_86159415 |
| 10 | 86087688 | 86165414 | 19 | 77727 | s75340.1 | oar3_OAR10_86165414 |
| 10 | 86087688 | 86159415 | 18 | 71728 | s75340.1 | oar3_OAR10_86159415 |
| 10 | 86087688 | 86373686 | 53 | 285999 | s75340.1 | oar3_OAR10_86373686 |
| 10 | 86087688 | 86260521 | 31 | 172834 | s75340.1 | oar3_OAR10_86260521 |
| 10 | 86100187 | 86159415 | 17 | 59229 | oar3_OAR10_86100187 | oar3_OAR10_86159415 |
| 10 | 86100187 | 86110808 | 6 | 10622 | oar3_OAR10_86100187 | oar3_OAR10_86110808 |
| 10 | 86100187 | 86373686 | 52 | 273500 | oar3_OAR10_86100187 | oar3_OAR10_86373686 |
| 10 | 86100187 | 86159415 | 17 | 59229 | oar3_OAR10_86100187 | oar3_OAR10_86159415 |
| 10 | 86146676 | 86165414 | 5 | 18739 | oar3_OAR10_86146676 | oar3_OAR10_86165414 |
| 10 | 86217351 | 86385186 | 27 | 167836 | oar3_OAR10_86217351 | oar3_OAR10_86385186 |
| 10 | 86217351 | 86268830 | 6 | 51480 | oar3_OAR10_86217351 | DU455689_544.1 |
| 10 | 86248344 | 86373686 | 25 | 125343 | oar3_OAR10_86248344 | oar3_OAR10_86373686 |
| 10 | 86329286 | 86373686 | 11 | 44401 | oar3_OAR10_86329286 | oar3_OAR10_86373686 |
| 10 | 86340569 | 86373686 | 9 | 33118 | oar3_OAR10_86340569 | oar3_OAR10_86373686 |
| 10 | 86365977 | 86427606 | 11 | 61630 | oar3_OAR10_86365977 | oar3_OAR10_86427606 |
| 11 | 5883754 | 6079775 | 37 | 196022 | oar3_OAR11_5883754 | oar3_OAR11_6079775 |
| 11 | 7898325 | 7911082 | 6 | 12758 | s36598.1 | oar3_OAR11_7911082 |
| 11 | 7977824 | 7982544 | 5 | 4721 | oar3_OAR11_7977824 | oar3_OAR11_7982544 |
| 11 | 7977824 | 8011802 | 14 | 33979 | oar3_OAR11_7977824 | oar3_OAR11_8011802 |
| 11 | 7995447 | 8029624 | 10 | 34178 | oar3_OAR11_7995447 | s48086.1 |
| 11 | 8822393 | 8858442 | 11 | 36050 | s55203.1 | oar3_OAR11_8858442 |
| 11 | 8822393 | 8858442 | 11 | 36050 | s55203.1 | oar3_OAR11_8858442 |
| 11 | 8835088 | 8850710 | 7 | 15623 | oar3_OAR11_8835088 | oar3_OAR11_8850710 |
| 11 | 8837978 | 8887688 | 14 | 49711 | oar3_OAR11_8837978 | oar3_OAR11_8887688 |
| 11 | 11169386 | 11272711 | 28 | 103326 | oar3_OAR11_11169386 | s22806.1 |
| 11 | 11169386 | 11272711 | 28 | 103326 | oar3_OAR11_11169386 | s22806.1 |
| 11 | 11185011 | 11205587 | 7 | 20577 | oar3_OAR11_11185011 | oar3_OAR11_11205587 |
| 11 | 11186663 | 11205587 | 6 | 18925 | oar3_OAR11_11186663 | oar3_OAR11_11205587 |
| 11 | 11186663 | 11272711 | 22 | 86049 | oar3_OAR11_11186663 | s22806.1 |
| 11 | 11228820 | 11254958 | 10 | 26139 | oar3_OAR11_11228820 | oar3_OAR11_11254958 |
| 11 | 11228820 | 11254958 | 10 | 26139 | oar3_OAR11_11228820 | oar3_OAR11_11254958 |
| 11 | 11228820 | 11254958 | 10 | 26139 | oar3_OAR11_11228820 | oar3_OAR11_11254958 |
| 11 | 11229387 | 11272711 | 11 | 43325 | s26679.1 | s22806.1 |
| 11 | 11229387 | 11254958 | 9 | 25572 | s26679.1 | oar3_OAR11_11254958 |
| 11 | 11242837 | 11272711 | 10 | 29875 | s26637.1 | s22806.1 |
| 11 | 11242837 | 11254958 | 8 | 12122 | s26637.1 | oar3_OAR11_11254958 |
| 11 | 11242837 | 11272711 | 10 | 29875 | s26637.1 | s22806.1 |
| 11 | 12377481 | 12387862 | 7 | 10382 | s11161.1 | oar3_OAR11_12387862 |
| 11 | 12377481 | 12384450 | 6 | 6970 | s11161.1 | oar3_OAR11_12384450 |
| 11 | 12377481 | 12387862 | 7 | 10382 | s11161.1 | oar3_OAR11_12387862 |
| 11 | 12377648 | 12384450 | 5 | 6803 | oar3_OAR11_12377648 | oar3_OAR11_12384450 |
| 11 | 12411072 | 12436039 | 11 | 24968 | oar3_OAR11_12411072 | oar3_OAR11_12436039 |
| 11 | 12414342 | 12440130 | 14 | 25789 | s40053.1 | oar3_OAR11_12440130 |
| 11 | 12426518 | 12442570 | 10 | 16053 | oar3_OAR11_12426518 | oar3_OAR11_12442570 |
| 11 | 14192110 | 14234275 | 15 | 42166 | oar3_OAR11_14192110 | oar3_OAR11_14234275 |
| 11 | 14211449 | 14228600 | 8 | 17152 | oar3_OAR11_14211449 | oar3_OAR11_14228600 |
| 11 | 14211449 | 14234275 | 9 | 22827 | oar3_OAR11_14211449 | oar3_OAR11_14234275 |
| 11 | 14211449 | 14234275 | 9 | 22827 | oar3_OAR11_14211449 | oar3_OAR11_14234275 |
| 11 | 14211449 | 14228600 | 8 | 17152 | oar3_OAR11_14211449 | oar3_OAR11_14228600 |
| 11 | 14227157 | 14234275 | 5 | 7119 | oar3_OAR11_14227157 | oar3_OAR11_14234275 |
| 11 | 14227157 | 14234275 | 5 | 7119 | oar3_OAR11_14227157 | oar3_OAR11_14234275 |
| 11 | 14503429 | 14573754 | 20 | 70326 | oar3_OAR11_14503429 | oar3_OAR11_14573754 |
| 11 | 14533381 | 14573754 | 12 | 40374 | oar3_OAR11_14533381 | oar3_OAR11_14573754 |
| 11 | 14547807 | 14599741 | 15 | 51935 | oar3_OAR11_14547807 | oar3_OAR11_14599741 |
| 11 | 14547807 | 14560638 | 6 | 12832 | oar3_OAR11_14547807 | oar3_OAR11_14560638 |
| 11 | 14547807 | 14560638 | 6 | 12832 | oar3_OAR11_14547807 | oar3_OAR11_14560638 |
| 11 | 14547807 | 14560638 | 6 | 12832 | oar3_OAR11_14547807 | oar3_OAR11_14560638 |
| 11 | 14905266 | 14939292 | 12 | 34027 | oar3_OAR11_14905266 | oar3_OAR11_14939292 |
| 11 | 14905266 | 14943970 | 13 | 38705 | oar3_OAR11_14905266 | oar3_OAR11_14943970 |
| 11 | 15044054 | 15115650 | 19 | 71597 | oar3_OAR11_15044054 | oar3_OAR11_15115650 |
| 11 | 15055122 | 15115650 | 15 | 60529 | oar3_OAR11_15055122 | oar3_OAR11_15115650 |
| 11 | 15069561 | 15115650 | 12 | 46090 | oar3_OAR11_15069561 | oar3_OAR11_15115650 |
| 11 | 15069561 | 15115650 | 12 | 46090 | oar3_OAR11_15069561 | oar3_OAR11_15115650 |
| 11 | 19475195 | 19517094 | 12 | 41900 | oar3_OAR11_19475195 | oar3_OAR11_19517094 |
| 11 | 19582031 | 19637438 | 16 | 55408 | oar3_OAR11_19582031 | oar3_OAR11_19637438 |
| 11 | 19623316 | 19637438 | 5 | 14123 | oar3_OAR11_19623316 | oar3_OAR11_19637438 |
| 11 | 19781057 | 19782666 | 3 | 1610 | oar3_OAR11_19781057 | oar3_OAR11_19782666 |
| 11 | 20161424 | 20192046 | 10 | 30623 | oar3_OAR11_20161424 | oar3_OAR11_20192046 |
| 11 | 20454692 | 20505743 | 13 | 51052 | oar3_OAR11_20454692 | oar3_OAR11_20505743 |
| 11 | 20467139 | 20505743 | 10 | 38605 | oar3_OAR11_20467139 | oar3_OAR11_20505743 |
| 11 | 21216407 | 21257157 | 13 | 40751 | oar3_OAR11_21216407 | oar3_OAR11_21257157 |
| 11 | 21335420 | 21345537 | 5 | 10118 | oar3_OAR11_21335420 | oar3_OAR11_21345537 |
| 11 | 21440822 | 21483181 | 13 | 42360 | s28931.1 | oar3_OAR11_21483181 |
| 11 | 21473777 | 21483181 | 5 | 9405 | oar3_OAR11_21473777 | oar3_OAR11_21483181 |
| 11 | 22072132 | 22102513 | 9 | 30382 | oar3_OAR11_22072132 | oar3_OAR11_22102513 |
| 11 | 22095899 | 22113776 | 8 | 17878 | oar3_OAR11_22095899 | oar3_OAR11_22113776 |
| 11 | 22127540 | 22141277 | 4 | 13738 | oar3_OAR11_22127540 | oar3_OAR11_22141277 |
| 11 | 22219991 | 22318466 | 21 | 98476 | oar3_OAR11_22219991 | oar3_OAR11_22318466 |
| 11 | 22266211 | 22373624 | 25 | 107414 | oar3_OAR11_22266211 | oar3_OAR11_22373624 |
| 11 | 22644757 | 22711206 | 15 | 66450 | oar3_OAR11_22644757 | oar3_OAR11_22711206 |
| 11 | 22662765 | 22711206 | 11 | 48442 | oar3_OAR11_22662765 | oar3_OAR11_22711206 |
| 11 | 22682876 | 22711206 | 6 | 28331 | oar3_OAR11_22682876 | oar3_OAR11_22711206 |
| 11 | 22944345 | 22997302 | 14 | 52958 | oar3_OAR11_22944345 | oar3_OAR11_22997302 |
| 11 | 23201077 | 23270342 | 16 | 69266 | oar3_OAR11_23201077 | oar3_OAR11_23270342 |
| 11 | 23220397 | 23243607 | 9 | 23211 | oar3_OAR11_23220397 | oar3_OAR11_23243607 |
| 11 | 24128485 | 24211850 | 18 | 83366 | oar3_OAR11_24128485 | oar3_OAR11_24211850 |
| 11 | 24151007 | 24219944 | 13 | 68938 | oar3_OAR11_24151007 | oar3_OAR11_24219944 |
| 11 | 24170989 | 24219944 | 10 | 48956 | oar3_OAR11_24170989 | oar3_OAR11_24219944 |
| 11 | 24170989 | 24252950 | 18 | 81962 | oar3_OAR11_24170989 | oar3_OAR11_24252950 |
| 11 | 24170989 | 24219944 | 10 | 48956 | oar3_OAR11_24170989 | oar3_OAR11_24219944 |
| 11 | 24170989 | 24219944 | 10 | 48956 | oar3_OAR11_24170989 | oar3_OAR11_24219944 |
| 11 | 24170989 | 24219944 | 10 | 48956 | oar3_OAR11_24170989 | oar3_OAR11_24219944 |
| 11 | 24170989 | 24219944 | 10 | 48956 | oar3_OAR11_24170989 | oar3_OAR11_24219944 |
| 11 | 24170989 | 24211850 | 8 | 40862 | oar3_OAR11_24170989 | oar3_OAR11_24211850 |
| 11 | 24635038 | 24727793 | 21 | 92756 | oar3_OAR11_24635038 | oar3_OAR11_24727793 |
| 11 | 24713257 | 24727793 | 4 | 14537 | oar3_OAR11_24713257 | oar3_OAR11_24727793 |
| 11 | 25033422 | 25034930 | 5 | 1509 | oar3_OAR11_25033422 | oar3_OAR11_25034930 |
| 11 | 25033422 | 25034930 | 5 | 1509 | oar3_OAR11_25033422 | oar3_OAR11_25034930 |
| 11 | 25033422 | 25055089 | 10 | 21668 | oar3_OAR11_25033422 | oar3_OAR11_25055089 |
| 11 | 26580667 | 26768180 | 26 | 187514 | oar3_OAR11_26580667 | oar3_OAR11_26768180 |
| 11 | 26580667 | 26768180 | 26 | 187514 | oar3_OAR11_26580667 | oar3_OAR11_26768180 |
| 11 | 26701950 | 26731105 | 7 | 29156 | OAR11_27937828.1 | oar3_OAR11_26731105 |
| 11 | 26723654 | 26752605 | 8 | 28952 | oar3_OAR11_26723654 | oar3_OAR11_26752605 |
| 11 | 26723654 | 26752605 | 8 | 28952 | oar3_OAR11_26723654 | oar3_OAR11_26752605 |
| 11 | 27857148 | 27882187 | 9 | 25040 | oar3_OAR11_27857148 | oar3_OAR11_27882187 |
| 11 | 27857148 | 27869067 | 7 | 11920 | oar3_OAR11_27857148 | oar3_OAR11_27869067 |
| 11 | 28010143 | 28019509 | 4 | 9367 | oar3_OAR11_28010143 | s48574.1 |
| 11 | 28010143 | 28029244 | 6 | 19102 | oar3_OAR11_28010143 | oar3_OAR11_28029244 |
| 11 | 28202090 | 28220616 | 6 | 18527 | oar3_OAR11_28202090 | oar3_OAR11_28220616 |
| 11 | 28887977 | 28934932 | 14 | 46956 | oar3_OAR11_28887977 | oar3_OAR11_28934932 |
| 11 | 28887977 | 28913560 | 10 | 25584 | oar3_OAR11_28887977 | oar3_OAR11_28913560 |
| 11 | 30902492 | 30936599 | 15 | 34108 | oar3_OAR11_30902492 | oar3_OAR11_30936599 |
| 11 | 30902492 | 30936599 | 15 | 34108 | oar3_OAR11_30902492 | oar3_OAR11_30936599 |
| 11 | 30902492 | 30936599 | 15 | 34108 | oar3_OAR11_30902492 | oar3_OAR11_30936599 |
| 11 | 30912724 | 30936599 | 13 | 23876 | oar3_OAR11_30912724 | oar3_OAR11_30936599 |
| 11 | 31742414 | 31763362 | 10 | 20949 | oar3_OAR11_31742414 | oar3_OAR11_31763362 |
| 11 | 33600844 | 33811826 | 44 | 210983 | oar3_OAR11_33600844 | oar3_OAR11_33811826 |
| 11 | 33603019 | 34885663 | 244 | 1282645 | oar3_OAR11_33603019 | s71936.1 |
| 11 | 33603019 | 33811826 | 43 | 208808 | oar3_OAR11_33603019 | oar3_OAR11_33811826 |
| 11 | 33603019 | 33811826 | 43 | 208808 | oar3_OAR11_33603019 | oar3_OAR11_33811826 |
| 11 | 33607756 | 33720323 | 24 | 112568 | oar3_OAR11_33607756 | oar3_OAR11_33720323 |
| 11 | 33649879 | 33724963 | 19 | 75085 | oar3_OAR11_33649879 | oar3_OAR11_33724963 |
| 11 | 33755883 | 33811826 | 10 | 55944 | oar3_OAR11_33755883 | oar3_OAR11_33811826 |
| 11 | 33755883 | 33976428 | 38 | 220546 | oar3_OAR11_33755883 | oar3_OAR11_33976428 |
| 11 | 33755883 | 33811826 | 10 | 55944 | oar3_OAR11_33755883 | oar3_OAR11_33811826 |
| 11 | 33755883 | 33766197 | 3 | 10315 | oar3_OAR11_33755883 | oar3_OAR11_33766197 |
| 11 | 33793692 | 33808725 | 6 | 15034 | s73872.1 | oar3_OAR11_33808725 |
| 11 | 33868688 | 34029369 | 28 | 160682 | oar3_OAR11_33868688 | OAR11_36358079.1 |
| 11 | 33880806 | 34029369 | 27 | 148564 | oar3_OAR11_33880806 | OAR11_36358079.1 |
| 11 | 33896538 | 33922382 | 9 | 25845 | oar3_OAR11_33896538 | oar3_OAR11_33922382 |
| 11 | 33967610 | 34124033 | 40 | 156424 | oar3_OAR11_33967610 | oar3_OAR11_34124033 |
| 11 | 34072630 | 34091412 | 10 | 18783 | s33971.1 | s55707.1 |
| 11 | 34072630 | 34244675 | 40 | 172046 | s33971.1 | oar3_OAR11_34244675 |
| 11 | 34213586 | 34244675 | 11 | 31090 | oar3_OAR11_34213586 | oar3_OAR11_34244675 |
| 11 | 34213586 | 34244675 | 11 | 31090 | oar3_OAR11_34213586 | oar3_OAR11_34244675 |
| 11 | 34213586 | 34244675 | 11 | 31090 | oar3_OAR11_34213586 | oar3_OAR11_34244675 |
| 11 | 34219844 | 34244675 | 10 | 24832 | oar3_OAR11_34219844 | oar3_OAR11_34244675 |
| 11 | 34340494 | 34377894 | 10 | 37401 | oar3_OAR11_34340494 | s04372.1 |
| 11 | 34444047 | 34586518 | 33 | 142472 | oar3_OAR11_34444047 | oar3_OAR11_34586518 |
| 11 | 34491596 | 34586518 | 22 | 94923 | oar3_OAR11_34491596 | oar3_OAR11_34586518 |
| 11 | 34511665 | 34586518 | 21 | 74854 | oar3_OAR11_34511665 | oar3_OAR11_34586518 |
| 11 | 34511665 | 34579114 | 19 | 67450 | oar3_OAR11_34511665 | oar3_OAR11_34579114 |
| 11 | 34511665 | 34579114 | 19 | 67450 | oar3_OAR11_34511665 | oar3_OAR11_34579114 |
| 11 | 34511665 | 34579114 | 19 | 67450 | oar3_OAR11_34511665 | oar3_OAR11_34579114 |
| 11 | 34511665 | 34548051 | 8 | 36387 | oar3_OAR11_34511665 | oar3_OAR11_34548051 |
| 11 | 34511665 | 34586518 | 21 | 74854 | oar3_OAR11_34511665 | oar3_OAR11_34586518 |
| 11 | 34511665 | 34548051 | 8 | 36387 | oar3_OAR11_34511665 | oar3_OAR11_34548051 |
| 11 | 34524475 | 34579114 | 17 | 54640 | s12868.1 | oar3_OAR11_34579114 |
| 11 | 34645916 | 34660421 | 5 | 14506 | oar3_OAR11_34645916 | oar3_OAR11_34660421 |
| 11 | 34738597 | 34807524 | 20 | 68928 | oar3_OAR11_34738597 | oar3_OAR11_34807524 |
| 11 | 34761410 | 34840831 | 19 | 79422 | oar3_OAR11_34761410 | oar3_OAR11_34840831 |
| 11 | 34761410 | 34805719 | 10 | 44310 | oar3_OAR11_34761410 | s48160.1 |
| 11 | 34787666 | 34807524 | 9 | 19859 | oar3_OAR11_34787666 | oar3_OAR11_34807524 |
| 11 | 34876441 | 34894423 | 8 | 17983 | oar3_OAR11_34876441 | oar3_OAR11_34894423 |
| 11 | 34876441 | 34894423 | 8 | 17983 | oar3_OAR11_34876441 | oar3_OAR11_34894423 |
| 11 | 34876441 | 34894423 | 8 | 17983 | oar3_OAR11_34876441 | oar3_OAR11_34894423 |
| 11 | 34876441 | 34894423 | 8 | 17983 | oar3_OAR11_34876441 | oar3_OAR11_34894423 |
| 11 | 35634797 | 35786289 | 43 | 151493 | s13385.1 | oar3_OAR11_35786289 |
| 11 | 35658354 | 35732984 | 21 | 74631 | oar3_OAR11_35658354 | oar3_OAR11_35732984 |
| 11 | 35658354 | 35784020 | 35 | 125667 | oar3_OAR11_35658354 | oar3_OAR11_35784020 |
| 11 | 35772661 | 35786289 | 7 | 13629 | oar3_OAR11_35772661 | oar3_OAR11_35786289 |
| 11 | 36001807 | 36332804 | 82 | 330998 | oar3_OAR11_36001807 | oar3_OAR11_36332804 |
| 11 | 36001807 | 36087607 | 28 | 85801 | oar3_OAR11_36001807 | s70713.1 |
| 11 | 36288338 | 36332804 | 9 | 44467 | oar3_OAR11_36288338 | oar3_OAR11_36332804 |
| 11 | 36288338 | 36332804 | 9 | 44467 | oar3_OAR11_36288338 | oar3_OAR11_36332804 |
| 11 | 36571336 | 36583136 | 7 | 11801 | oar3_OAR11_36571336 | oar3_OAR11_36583136 |
| 11 | 36578601 | 36663978 | 22 | 85378 | oar3_OAR11_36578601 | oar3_OAR11_36663978 |
| 11 | 36578601 | 36583136 | 5 | 4536 | oar3_OAR11_36578601 | oar3_OAR11_36583136 |
| 11 | 36578601 | 36583136 | 5 | 4536 | oar3_OAR11_36578601 | oar3_OAR11_36583136 |
| 11 | 37398200 | 37501773 | 19 | 103574 | oar3_OAR11_37398200 | oar3_OAR11_37501773 |
| 11 | 38170551 | 38207311 | 17 | 36761 | oar3_OAR11_38170551 | oar3_OAR11_38207311 |
| 11 | 38170551 | 38230196 | 22 | 59646 | oar3_OAR11_38170551 | oar3_OAR11_38230196 |
| 11 | 38554499 | 38566670 | 15 | 12172 | oar3_OAR11_38554499 | oar3_OAR11_38566670 |
| 11 | 38554499 | 38915095 | 73 | 360597 | oar3_OAR11_38554499 | oar3_OAR11_38915095 |
| 11 | 38554499 | 38589393 | 22 | 34895 | oar3_OAR11_38554499 | oar3_OAR11_38589393 |
| 11 | 38556314 | 38566670 | 14 | 10357 | oar3_OAR11_38556314 | oar3_OAR11_38566670 |
| 11 | 38556314 | 38566670 | 14 | 10357 | oar3_OAR11_38556314 | oar3_OAR11_38566670 |
| 11 | 38739538 | 38815011 | 14 | 75474 | oar3_OAR11_38739538 | oar3_OAR11_38815011 |
| 11 | 39247881 | 39293059 | 12 | 45179 | oar3_OAR11_39247881 | oar3_OAR11_39293059 |
| 11 | 39260250 | 39318212 | 17 | 57963 | oar3_OAR11_39260250 | oar3_OAR11_39318212 |
| 11 | 39260250 | 39318212 | 17 | 57963 | oar3_OAR11_39260250 | oar3_OAR11_39318212 |
| 11 | 39260250 | 39283306 | 8 | 23057 | oar3_OAR11_39260250 | oar3_OAR11_39283306 |
| 11 | 39260250 | 39283306 | 8 | 23057 | oar3_OAR11_39260250 | oar3_OAR11_39283306 |
| 11 | 39594580 | 39691100 | 26 | 96521 | oar3_OAR11_39594580 | oar3_OAR11_39691100 |
| 11 | 39642572 | 39655624 | 8 | 13053 | oar3_OAR11_39642572 | oar3_OAR11_39655624 |
| 11 | 39642572 | 39691100 | 16 | 48529 | oar3_OAR11_39642572 | oar3_OAR11_39691100 |
| 11 | 39642572 | 39691100 | 16 | 48529 | oar3_OAR11_39642572 | oar3_OAR11_39691100 |
| 11 | 39642572 | 39655624 | 8 | 13053 | oar3_OAR11_39642572 | oar3_OAR11_39655624 |
| 11 | 40104864 | 40285832 | 18 | 180969 | oar3_OAR11_40104864 | oar3_OAR11_40285832 |
| 11 | 41323953 | 41361079 | 16 | 37127 | oar3_OAR11_41323953 | oar3_OAR11_41361079 |
| 11 | 41323953 | 41363952 | 17 | 40000 | oar3_OAR11_41323953 | oar3_OAR11_41363952 |
| 11 | 41323953 | 41363952 | 17 | 40000 | oar3_OAR11_41323953 | oar3_OAR11_41363952 |
| 11 | 41660394 | 41846360 | 37 | 185967 | oar3_OAR11_41660394 | oar3_OAR11_41846360 |
| 11 | 41660394 | 41719022 | 11 | 58629 | oar3_OAR11_41660394 | oar3_OAR11_41719022 |
| 11 | 41660394 | 41719022 | 11 | 58629 | oar3_OAR11_41660394 | oar3_OAR11_41719022 |
| 11 | 41660394 | 41699221 | 9 | 38828 | oar3_OAR11_41660394 | oar3_OAR11_41699221 |
| 11 | 41670181 | 41692364 | 6 | 22184 | oar3_OAR11_41670181 | s02748.1 |
| 11 | 41692364 | 41846360 | 31 | 153997 | s02748.1 | oar3_OAR11_41846360 |
| 11 | 41698306 | 41796063 | 21 | 97758 | oar3_OAR11_41698306 | oar3_OAR11_41796063 |
| 11 | 41761686 | 41827949 | 15 | 66264 | oar3_OAR11_41761686 | oar3_OAR11_41827949 |
| 11 | 41769201 | 41788310 | 7 | 19110 | oar3_OAR11_41769201 | OAR11_44415869.1 |
| 11 | 42087180 | 42143777 | 14 | 56598 | oar3_OAR11_42087180 | oar3_OAR11_42143777 |
| 11 | 42090320 | 42117127 | 11 | 26808 | oar3_OAR11_42090320 | oar3_OAR11_42117127 |
| 11 | 42111273 | 42135398 | 5 | 24126 | oar3_OAR11_42111273 | oar3_OAR11_42135398 |
| 11 | 43075709 | 43141662 | 19 | 65954 | oar3_OAR11_43075709 | oar3_OAR11_43141662 |
| 11 | 43332867 | 43596072 | 50 | 263206 | oar3_OAR11_43332867 | oar3_OAR11_43596072 |
| 11 | 43332867 | 43353189 | 7 | 20323 | oar3_OAR11_43332867 | oar3_OAR11_43353189 |
| 11 | 43395800 | 43453822 | 13 | 58023 | oar3_OAR11_43395800 | oar3_OAR11_43453822 |
| 11 | 43572013 | 43578270 | 4 | 6258 | s60148.1 | oar3_OAR11_43578270 |
| 11 | 44031324 | 44101148 | 23 | 69825 | oar3_OAR11_44031324 | s27884.1 |
| 11 | 44034755 | 44503975 | 134 | 469221 | oar3_OAR11_44034755 | oar3_OAR11_44503975 |
| 11 | 44135630 | 44163273 | 13 | 27644 | oar3_OAR11_44135630 | oar3_OAR11_44163273 |
| 11 | 44135630 | 44260239 | 38 | 124610 | oar3_OAR11_44135630 | oar3_OAR11_44260239 |
| 11 | 44143555 | 44195928 | 20 | 52374 | oar3_OAR11_44143555 | oar3_OAR11_44195928 |
| 11 | 44151112 | 44159904 | 7 | 8793 | oar3_OAR11_44151112 | oar3_OAR11_44159904 |
| 11 | 44212313 | 44314667 | 31 | 102355 | oar3_OAR11_44212313 | oar3_OAR11_44314667 |
| 11 | 44231137 | 44260239 | 11 | 29103 | OAR11_47020039.1 | oar3_OAR11_44260239 |
| 11 | 44232928 | 44243639 | 5 | 10712 | oar3_OAR11_44232928 | oar3_OAR11_44243639 |
| 11 | 44291122 | 44359836 | 24 | 68715 | oar3_OAR11_44291122 | oar3_OAR11_44359836 |
| 11 | 44338754 | 44361965 | 12 | 23212 | oar3_OAR11_44338754 | oar3_OAR11_44361965 |
| 11 | 44338754 | 44361965 | 12 | 23212 | oar3_OAR11_44338754 | oar3_OAR11_44361965 |
| 11 | 44468151 | 44521859 | 14 | 53709 | oar3_OAR11_44468151 | oar3_OAR11_44521859 |
| 11 | 44476413 | 44503975 | 11 | 27563 | oar3_OAR11_44476413 | oar3_OAR11_44503975 |
| 11 | 44630080 | 44865090 | 58 | 235011 | oar3_OAR11_44630080 | oar3_OAR11_44865090 |
| 11 | 44753284 | 44772635 | 6 | 19352 | OAR11_47584423.1 | oar3_OAR11_44772635 |
| 11 | 45389392 | 45402092 | 10 | 12701 | oar3_OAR11_45389392 | oar3_OAR11_45402092 |
| 11 | 46457922 | 46543485 | 29 | 85564 | oar3_OAR11_46457922 | oar3_OAR11_46543485 |
| 11 | 46492824 | 46507910 | 9 | 15087 | oar3_OAR11_46492824 | oar3_OAR11_46507910 |
| 11 | 47178911 | 47286015 | 30 | 107105 | oar3_OAR11_47178911 | oar3_OAR11_47286015 |
| 11 | 47254475 | 47286015 | 12 | 31541 | oar3_OAR11_47254475 | oar3_OAR11_47286015 |
| 11 | 47272387 | 47275592 | 3 | 3206 | oar3_OAR11_47272387 | oar3_OAR11_47275592 |
| 11 | 47272387 | 47286015 | 5 | 13629 | oar3_OAR11_47272387 | oar3_OAR11_47286015 |
| 11 | 47272387 | 47286015 | 5 | 13629 | oar3_OAR11_47272387 | oar3_OAR11_47286015 |
| 11 | 47567085 | 47711853 | 37 | 144769 | oar3_OAR11_47567085 | oar3_OAR11_47711853 |
| 11 | 47567085 | 47586499 | 8 | 19415 | oar3_OAR11_47567085 | oar3_OAR11_47586499 |
| 11 | 47573681 | 47650980 | 17 | 77300 | oar3_OAR11_47573681 | oar3_OAR11_47650980 |
| 11 | 47573681 | 47583294 | 4 | 9614 | oar3_OAR11_47573681 | oar3_OAR11_47583294 |
| 11 | 48978053 | 49038684 | 18 | 60632 | oar3_OAR11_48978053 | oar3_OAR11_49038684 |
| 11 | 49013992 | 49043988 | 12 | 29997 | oar3_OAR11_49013992 | oar3_OAR11_49043988 |
| 11 | 49013992 | 49038684 | 11 | 24693 | oar3_OAR11_49013992 | oar3_OAR11_49038684 |
| 11 | 49013992 | 49025008 | 4 | 11017 | oar3_OAR11_49013992 | oar3_OAR11_49025008 |
| 11 | 49100050 | 49126020 | 13 | 25971 | oar3_OAR11_49100050 | oar3_OAR11_49126020 |
| 11 | 49115827 | 49126020 | 8 | 10194 | oar3_OAR11_49115827 | oar3_OAR11_49126020 |
| 11 | 49146801 | 49178365 | 10 | 31565 | oar3_OAR11_49146801 | oar3_OAR11_49178365 |
| 11 | 49146801 | 49178365 | 10 | 31565 | oar3_OAR11_49146801 | oar3_OAR11_49178365 |
| 11 | 49146801 | 49178365 | 10 | 31565 | oar3_OAR11_49146801 | oar3_OAR11_49178365 |
| 11 | 49146801 | 49178365 | 10 | 31565 | oar3_OAR11_49146801 | oar3_OAR11_49178365 |
| 11 | 49146801 | 49178365 | 10 | 31565 | oar3_OAR11_49146801 | oar3_OAR11_49178365 |
| 11 | 49146801 | 49178365 | 10 | 31565 | oar3_OAR11_49146801 | oar3_OAR11_49178365 |
| 11 | 49146801 | 49159191 | 5 | 12391 | oar3_OAR11_49146801 | oar3_OAR11_49159191 |
| 11 | 49146801 | 49178365 | 10 | 31565 | oar3_OAR11_49146801 | oar3_OAR11_49178365 |
| 11 | 49146801 | 49178365 | 10 | 31565 | oar3_OAR11_49146801 | oar3_OAR11_49178365 |
| 11 | 49498051 | 49550932 | 15 | 52882 | oar3_OAR11_49498051 | oar3_OAR11_49550932 |
| 11 | 49498051 | 49513561 | 7 | 15511 | oar3_OAR11_49498051 | oar3_OAR11_49513561 |
| 11 | 49498051 | 49530131 | 11 | 32081 | oar3_OAR11_49498051 | oar3_OAR11_49530131 |
| 11 | 49590326 | 49655442 | 6 | 65117 | oar3_OAR11_49590326 | oar3_OAR11_49655442 |
| 11 | 49956916 | 50795750 | 190 | 838835 | oar3_OAR11_49956916 | s65293.1 |
| 11 | 49956916 | 49987461 | 5 | 30546 | oar3_OAR11_49956916 | s08804.1 |
| 11 | 49956916 | 50099290 | 26 | 142375 | oar3_OAR11_49956916 | oar3_OAR11_50099290 |
| 11 | 49956916 | 49987461 | 5 | 30546 | oar3_OAR11_49956916 | s08804.1 |
| 11 | 49956916 | 49987461 | 5 | 30546 | oar3_OAR11_49956916 | s08804.1 |
| 11 | 49975930 | 49984979 | 3 | 9050 | oar3_OAR11_49975930 | oar3_OAR11_49984979 |
| 11 | 49975930 | 49987461 | 4 | 11532 | oar3_OAR11_49975930 | s08804.1 |
| 11 | 49975930 | 49987461 | 4 | 11532 | oar3_OAR11_49975930 | s08804.1 |
| 11 | 49975930 | 49987461 | 4 | 11532 | oar3_OAR11_49975930 | s08804.1 |
| 11 | 50087824 | 50266978 | 41 | 179155 | oar3_OAR11_50087824 | oar3_OAR11_50266978 |
| 11 | 50087824 | 50131944 | 14 | 44121 | oar3_OAR11_50087824 | OAR11_53479950.1 |
| 11 | 50087824 | 50096346 | 4 | 8523 | oar3_OAR11_50087824 | oar3_OAR11_50096346 |
| 11 | 50093481 | 50247507 | 36 | 154027 | oar3_OAR11_50093481 | oar3_OAR11_50247507 |
| 11 | 50093481 | 50099290 | 5 | 5810 | oar3_OAR11_50093481 | oar3_OAR11_50099290 |
| 11 | 50109029 | 50166979 | 15 | 57951 | oar3_OAR11_50109029 | oar3_OAR11_50166979 |
| 11 | 50125796 | 50166979 | 12 | 41184 | oar3_OAR11_50125796 | oar3_OAR11_50166979 |
| 11 | 50212646 | 50266978 | 13 | 54333 | oar3_OAR11_50212646 | oar3_OAR11_50266978 |
| 11 | 50212646 | 50249471 | 10 | 36826 | oar3_OAR11_50212646 | oar3_OAR11_50249471 |
| 11 | 50217656 | 50231458 | 7 | 13803 | s10035.1 | oar3_OAR11_50231458 |
| 11 | 50367730 | 50397427 | 15 | 29698 | oar3_OAR11_50367730 | oar3_OAR11_50397427 |
| 11 | 50367730 | 50397427 | 15 | 29698 | oar3_OAR11_50367730 | oar3_OAR11_50397427 |
| 11 | 50367730 | 50501570 | 37 | 133841 | oar3_OAR11_50367730 | oar3_OAR11_50501570 |
| 11 | 50367730 | 50391316 | 12 | 23587 | oar3_OAR11_50367730 | oar3_OAR11_50391316 |
| 11 | 50373431 | 50387839 | 9 | 14409 | oar3_OAR11_50373431 | oar3_OAR11_50387839 |
| 11 | 50379750 | 50416518 | 13 | 36769 | oar3_OAR11_50379750 | oar3_OAR11_50416518 |
| 11 | 50381092 | 50416518 | 9 | 35427 | oar3_OAR11_50381092 | oar3_OAR11_50416518 |
| 11 | 50381092 | 50485749 | 25 | 104658 | oar3_OAR11_50381092 | oar3_OAR11_50485749 |
| 11 | 50381092 | 50485749 | 25 | 104658 | oar3_OAR11_50381092 | oar3_OAR11_50485749 |
| 11 | 50381092 | 50425941 | 12 | 44850 | oar3_OAR11_50381092 | oar3_OAR11_50425941 |
| 11 | 50391316 | 50416518 | 6 | 25203 | oar3_OAR11_50391316 | oar3_OAR11_50416518 |
| 11 | 50393913 | 50404133 | 4 | 10221 | oar3_OAR11_50393913 | oar3_OAR11_50404133 |
| 11 | 50393913 | 50404133 | 4 | 10221 | oar3_OAR11_50393913 | oar3_OAR11_50404133 |
| 11 | 50464670 | 50533836 | 15 | 69167 | oar3_OAR11_50464670 | oar3_OAR11_50533836 |
| 11 | 50464670 | 50536165 | 16 | 71496 | oar3_OAR11_50464670 | oar3_OAR11_50536165 |
| 11 | 50469638 | 50536165 | 14 | 66528 | oar3_OAR11_50469638 | oar3_OAR11_50536165 |
| 11 | 50469638 | 50485749 | 4 | 16112 | oar3_OAR11_50469638 | oar3_OAR11_50485749 |
| 11 | 50586911 | 50735863 | 39 | 148953 | oar3_OAR11_50586911 | oar3_OAR11_50735863 |
| 11 | 50620546 | 50652703 | 11 | 32158 | s48700.1 | oar3_OAR11_50652703 |
| 11 | 50620546 | 50652703 | 11 | 32158 | s48700.1 | oar3_OAR11_50652703 |
| 11 | 50630769 | 50705798 | 27 | 75030 | oar3_OAR11_50630769 | oar3_OAR11_50705798 |
| 11 | 50630769 | 50747155 | 35 | 116387 | oar3_OAR11_50630769 | oar3_OAR11_50747155 |
| 11 | 50638637 | 50767653 | 45 | 129017 | oar3_OAR11_50638637 | oar3_OAR11_50767653 |
| 11 | 50641580 | 50795750 | 48 | 154171 | oar3_OAR11_50641580 | s65293.1 |
| 11 | 50695446 | 50747155 | 12 | 51710 | oar3_OAR11_50695446 | oar3_OAR11_50747155 |
| 11 | 50695446 | 50791477 | 27 | 96032 | oar3_OAR11_50695446 | oar3_OAR11_50791477 |
| 11 | 50705798 | 50749943 | 13 | 44146 | oar3_OAR11_50705798 | oar3_OAR11_50749943 |
| 11 | 50732695 | 50747155 | 6 | 14461 | oar3_OAR11_50732695 | oar3_OAR11_50747155 |
| 11 | 50732695 | 50791477 | 21 | 58783 | oar3_OAR11_50732695 | oar3_OAR11_50791477 |
| 11 | 51408119 | 51545739 | 39 | 137621 | oar3_OAR11_51408119 | oar3_OAR11_51545739 |
| 11 | 51408119 | 51428915 | 9 | 20797 | oar3_OAR11_51408119 | oar3_OAR11_51428915 |
| 11 | 51495855 | 51506952 | 6 | 11098 | s35678.1 | oar3_OAR11_51506952 |
| 11 | 51621092 | 51761587 | 24 | 140496 | oar3_OAR11_51621092 | OAR11_55335521.1 |
| 11 | 51723368 | 51748575 | 5 | 25208 | oar3_OAR11_51723368 | oar3_OAR11_51748575 |
| 11 | 52226107 | 52453689 | 71 | 227583 | oar3_OAR11_52226107 | oar3_OAR11_52453689 |
| 11 | 52226107 | 52278468 | 19 | 52362 | oar3_OAR11_52226107 | oar3_OAR11_52278468 |
| 11 | 52233003 | 52278468 | 18 | 45466 | oar3_OAR11_52233003 | oar3_OAR11_52278468 |
| 11 | 52244305 | 52342352 | 39 | 98048 | oar3_OAR11_52244305 | oar3_OAR11_52342352 |
| 11 | 52263807 | 52278468 | 9 | 14662 | oar3_OAR11_52263807 | oar3_OAR11_52278468 |
| 11 | 52263807 | 52278468 | 9 | 14662 | oar3_OAR11_52263807 | oar3_OAR11_52278468 |
| 11 | 52273618 | 52342352 | 25 | 68735 | oar3_OAR11_52273618 | oar3_OAR11_52342352 |
| 11 | 52275191 | 52342352 | 24 | 67162 | s54009.1 | oar3_OAR11_52342352 |
| 11 | 52309783 | 52342352 | 13 | 32570 | oar3_OAR11_52309783 | oar3_OAR11_52342352 |
| 11 | 52718317 | 53101425 | 95 | 383109 | oar3_OAR11_52718317 | oar3_OAR11_53101425 |
| 11 | 52906832 | 52918380 | 5 | 11549 | oar3_OAR11_52906832 | oar3_OAR11_52918380 |
| 11 | 52978696 | 53017346 | 10 | 38651 | oar3_OAR11_52978696 | oar3_OAR11_53017346 |
| 11 | 52978696 | 53029788 | 14 | 51093 | oar3_OAR11_52978696 | oar3_OAR11_53029788 |
| 11 | 52978696 | 53064435 | 21 | 85740 | oar3_OAR11_52978696 | oar3_OAR11_53064435 |
| 11 | 53013672 | 53029788 | 7 | 16117 | oar3_OAR11_53013672 | oar3_OAR11_53029788 |
| 11 | 53531201 | 53572893 | 10 | 41693 | oar3_OAR11_53531201 | oar3_OAR11_53572893 |
| 11 | 53536305 | 53567568 | 8 | 31264 | s36067.1 | oar3_OAR11_53567568 |
| 11 | 54218762 | 54475613 | 64 | 256852 | oar3_OAR11_54218762 | oar3_OAR11_54475613 |
| 11 | 54258558 | 54286225 | 9 | 27668 | oar3_OAR11_54258558 | oar3_OAR11_54286225 |
| 11 | 54274134 | 54286225 | 4 | 12092 | oar3_OAR11_54274134 | oar3_OAR11_54286225 |
| 11 | 54337019 | 54367874 | 14 | 30856 | oar3_OAR11_54337019 | oar3_OAR11_54367874 |
| 11 | 54337019 | 54364072 | 12 | 27054 | oar3_OAR11_54337019 | oar3_OAR11_54364072 |
| 11 | 54342615 | 54364072 | 10 | 21458 | oar3_OAR11_54342615 | oar3_OAR11_54364072 |
| 11 | 54346416 | 54364072 | 9 | 17657 | oar3_OAR11_54346416 | oar3_OAR11_54364072 |
| 11 | 54351685 | 54361632 | 5 | 9948 | oar3_OAR11_54351685 | oar3_OAR11_54361632 |
| 11 | 54536495 | 54591901 | 20 | 55407 | oar3_OAR11_54536495 | oar3_OAR11_54591901 |
| 11 | 54540051 | 54567268 | 13 | 27218 | oar3_OAR11_54540051 | oar3_OAR11_54567268 |
| 11 | 54556828 | 54597245 | 13 | 40418 | s66714.1 | oar3_OAR11_54597245 |
| 11 | 54556828 | 54597245 | 13 | 40418 | s66714.1 | oar3_OAR11_54597245 |
| 11 | 54635396 | 54650480 | 15 | 15085 | oar3_OAR11_54635396 | oar3_OAR11_54650480 |
| 11 | 54635396 | 54660249 | 20 | 24854 | oar3_OAR11_54635396 | oar3_OAR11_54660249 |
| 11 | 54635396 | 54660249 | 20 | 24854 | oar3_OAR11_54635396 | oar3_OAR11_54660249 |
| 11 | 54635396 | 54650480 | 15 | 15085 | oar3_OAR11_54635396 | oar3_OAR11_54650480 |
| 11 | 54635396 | 54650480 | 15 | 15085 | oar3_OAR11_54635396 | oar3_OAR11_54650480 |
| 11 | 54635849 | 54650480 | 14 | 14632 | oar3_OAR11_54635849 | oar3_OAR11_54650480 |
| 11 | 54638872 | 54660249 | 18 | 21378 | oar3_OAR11_54638872 | oar3_OAR11_54660249 |
| 11 | 54644051 | 54650480 | 11 | 6430 | oar3_OAR11_54644051 | oar3_OAR11_54650480 |
| 11 | 54646102 | 54650480 | 8 | 4379 | oar3_OAR11_54646102 | oar3_OAR11_54650480 |
| 11 | 54646102 | 54650480 | 8 | 4379 | oar3_OAR11_54646102 | oar3_OAR11_54650480 |
| 11 | 54725273 | 54744146 | 8 | 18874 | oar3_OAR11_54725273 | oar3_OAR11_54744146 |
| 11 | 54725273 | 54744146 | 8 | 18874 | oar3_OAR11_54725273 | oar3_OAR11_54744146 |
| 11 | 54725273 | 54744146 | 8 | 18874 | oar3_OAR11_54725273 | oar3_OAR11_54744146 |
| 11 | 54725273 | 54753307 | 9 | 28035 | oar3_OAR11_54725273 | oar3_OAR11_54753307 |
| 11 | 54725273 | 54744146 | 8 | 18874 | oar3_OAR11_54725273 | oar3_OAR11_54744146 |
| 11 | 54742899 | 54886764 | 29 | 143866 | oar3_OAR11_54742899 | oar3_OAR11_54886764 |
| 11 | 54781724 | 54889451 | 24 | 107728 | oar3_OAR11_54781724 | s39480.1 |
| 11 | 54863204 | 54886764 | 15 | 23561 | oar3_OAR11_54863204 | oar3_OAR11_54886764 |
| 11 | 54863204 | 55063612 | 60 | 200409 | oar3_OAR11_54863204 | oar3_OAR11_55063612 |
| 11 | 54863204 | 54875173 | 8 | 11970 | oar3_OAR11_54863204 | oar3_OAR11_54875173 |
| 11 | 54863204 | 54886764 | 15 | 23561 | oar3_OAR11_54863204 | oar3_OAR11_54886764 |
| 11 | 54863927 | 54886764 | 14 | 22838 | oar3_OAR11_54863927 | oar3_OAR11_54886764 |
| 11 | 54886449 | 54886764 | 5 | 316 | oar3_OAR11_54886449 | oar3_OAR11_54886764 |
| 11 | 54936652 | 55055124 | 25 | 118473 | oar3_OAR11_54936652 | oar3_OAR11_55055124 |
| 11 | 54939690 | 55035237 | 23 | 95548 | s39842.1 | oar3_OAR11_55035237 |
| 11 | 54959292 | 55035237 | 22 | 75946 | oar3_OAR11_54959292 | oar3_OAR11_55035237 |
| 11 | 54970096 | 54994390 | 9 | 24295 | oar3_OAR11_54970096 | oar3_OAR11_54994390 |
| 11 | 54970096 | 55016668 | 15 | 46573 | oar3_OAR11_54970096 | oar3_OAR11_55016668 |
| 11 | 55016096 | 55055124 | 9 | 39029 | oar3_OAR11_55016096 | oar3_OAR11_55055124 |
| 11 | 55226624 | 55244728 | 6 | 18105 | oar3_OAR11_55226624 | oar3_OAR11_55244728 |
| 11 | 55238733 | 55449023 | 34 | 210291 | s27539.1 | oar3_OAR11_55449023 |
| 11 | 55238733 | 55244728 | 5 | 5996 | s27539.1 | oar3_OAR11_55244728 |
| 11 | 55238733 | 55244728 | 5 | 5996 | s27539.1 | oar3_OAR11_55244728 |
| 11 | 55348491 | 55575804 | 29 | 227314 | oar3_OAR11_55348491 | oar3_OAR11_55575804 |
| 11 | 55348491 | 55515914 | 19 | 167424 | oar3_OAR11_55348491 | oar3_OAR11_55515914 |
| 11 | 55466565 | 55601874 | 18 | 135310 | oar3_OAR11_55466565 | oar3_OAR11_55601874 |
| 11 | 55506864 | 55556754 | 9 | 49891 | oar3_OAR11_55506864 | oar3_OAR11_55556754 |
| 11 | 55515914 | 55568077 | 9 | 52164 | oar3_OAR11_55515914 | oar3_OAR11_55568077 |
| 11 | 55535713 | 55575804 | 8 | 40092 | oar3_OAR11_55535713 | oar3_OAR11_55575804 |
| 11 | 55545688 | 55556754 | 4 | 11067 | oar3_OAR11_55545688 | oar3_OAR11_55556754 |
| 11 | 55545688 | 55639035 | 19 | 93348 | oar3_OAR11_55545688 | oar3_OAR11_55639035 |
| 11 | 55591366 | 55660103 | 16 | 68738 | oar3_OAR11_55591366 | oar3_OAR11_55660103 |
| 11 | 55628501 | 55660103 | 10 | 31603 | oar3_OAR11_55628501 | oar3_OAR11_55660103 |
| 11 | 55929179 | 55973318 | 11 | 44140 | oar3_OAR11_55929179 | oar3_OAR11_55973318 |
| 11 | 55929179 | 55973318 | 11 | 44140 | oar3_OAR11_55929179 | oar3_OAR11_55973318 |
| 11 | 55953839 | 55973318 | 6 | 19480 | oar3_OAR11_55953839 | oar3_OAR11_55973318 |
| 11 | 56027069 | 56126152 | 29 | 99084 | oar3_OAR11_56027069 | oar3_OAR11_56126152 |
| 11 | 56027069 | 56130729 | 30 | 103661 | oar3_OAR11_56027069 | oar3_OAR11_56130729 |
| 11 | 56027069 | 56117662 | 27 | 90594 | oar3_OAR11_56027069 | oar3_OAR11_56117662 |
| 11 | 56981978 | 56990394 | 7 | 8417 | oar3_OAR11_56981978 | oar3_OAR11_56990394 |
| 11 | 57046929 | 57063049 | 6 | 16121 | oar3_OAR11_57046929 | oar3_OAR11_57063049 |
| 11 | 57566187 | 57589319 | 9 | 23133 | oar3_OAR11_57566187 | oar3_OAR11_57589319 |
| 11 | 60240740 | 60247111 | 4 | 6372 | oar3_OAR11_60240740 | oar3_OAR11_60247111 |
| 11 | 60240740 | 60247111 | 4 | 6372 | oar3_OAR11_60240740 | oar3_OAR11_60247111 |
| 11 | 60676156 | 60694016 | 11 | 17861 | oar3_OAR11_60676156 | oar3_OAR11_60694016 |
| 11 | 60676156 | 60713226 | 14 | 37071 | oar3_OAR11_60676156 | oar3_OAR11_60713226 |
| 11 | 60684489 | 60694016 | 10 | 9528 | oar3_OAR11_60684489 | oar3_OAR11_60694016 |
| 11 | 60689655 | 60694016 | 7 | 4362 | oar3_OAR11_60689655 | oar3_OAR11_60694016 |
| 11 | 60847932 | 60911025 | 16 | 63094 | oar3_OAR11_60847932 | oar3_OAR11_60911025 |
| 11 | 60872607 | 60911025 | 12 | 38419 | s21716.1 | oar3_OAR11_60911025 |
| 11 | 61042542 | 61086751 | 16 | 44210 | oar3_OAR11_61042542 | oar3_OAR11_61086751 |
| 11 | 61083935 | 61127378 | 15 | 43444 | oar3_OAR11_61083935 | oar3_OAR11_61127378 |
| 11 | 61083935 | 61127378 | 15 | 43444 | oar3_OAR11_61083935 | oar3_OAR11_61127378 |
| 11 | 61107143 | 61127378 | 7 | 20236 | oar3_OAR11_61107143 | oar3_OAR11_61127378 |
| 11 | 61111321 | 61127378 | 6 | 16058 | CZ923139_448.1 | oar3_OAR11_61127378 |
| 11 | 61111321 | 61127378 | 6 | 16058 | CZ923139_448.1 | oar3_OAR11_61127378 |
| 11 | 61118589 | 61152861 | 6 | 34273 | oar3_OAR11_61118589 | oar3_OAR11_61152861 |
| 11 | 61346794 | 61359174 | 7 | 12381 | oar3_OAR11_61346794 | oar3_OAR11_61359174 |
| 11 | 61510671 | 61537473 | 13 | 26803 | oar3_OAR11_61510671 | oar3_OAR11_61537473 |
| 11 | 61573597 | 61697946 | 39 | 124350 | oar3_OAR11_61573597 | oar3_OAR11_61697946 |
| 11 | 61768110 | 61884323 | 23 | 116214 | oar3_OAR11_61768110 | oar3_OAR11_61884323 |
| 11 | 61775184 | 61852193 | 16 | 77010 | oar3_OAR11_61775184 | oar3_OAR11_61852193 |
| 11 | 61784915 | 61852193 | 14 | 67279 | oar3_OAR11_61784915 | oar3_OAR11_61852193 |
| 11 | 61803254 | 61884323 | 16 | 81070 | oar3_OAR11_61803254 | oar3_OAR11_61884323 |
| 11 | 61837142 | 62043545 | 37 | 206404 | s50441.1 | oar3_OAR11_62043545 |
| 11 | 61837142 | 61876212 | 8 | 39071 | s50441.1 | oar3_OAR11_61876212 |
| 12 | 461929 | 517921 | 15 | 55993 | oar3_OAR12_461929 | oar3_OAR12_517921 |
| 12 | 640528 | 686482 | 17 | 45955 | oar3_OAR12_640528 | oar3_OAR12_686482 |
| 12 | 640528 | 686482 | 17 | 45955 | oar3_OAR12_640528 | oar3_OAR12_686482 |
| 12 | 1190747 | 1315546 | 38 | 124800 | oar3_OAR12_1190747 | oar3_OAR12_1315546 |
| 12 | 1220802 | 1312900 | 29 | 92099 | oar3_OAR12_1220802 | oar3_OAR12_1312900 |
| 12 | 1227408 | 1310102 | 26 | 82695 | oar3_OAR12_1227408 | oar3_OAR12_1310102 |
| 12 | 1227408 | 1313425 | 29 | 86018 | oar3_OAR12_1227408 | oar3_OAR12_1313425 |
| 12 | 1227408 | 1272639 | 14 | 45232 | oar3_OAR12_1227408 | oar3_OAR12_1272639 |
| 12 | 1243505 | 1294178 | 19 | 50674 | oar3_OAR12_1243505 | oar3_OAR12_1294178 |
| 12 | 1258167 | 1272639 | 8 | 14473 | oar3_OAR12_1258167 | oar3_OAR12_1272639 |
| 12 | 1488704 | 1542514 | 18 | 53811 | oar3_OAR12_1488704 | OAR12_4247318.1 |
| 12 | 2030972 | 2150145 | 33 | 119174 | oar3_OAR12_2030972 | oar3_OAR12_2150145 |
| 12 | 2097607 | 2142102 | 14 | 44496 | oar3_OAR12_2097607 | oar3_OAR12_2142102 |
| 12 | 2121348 | 2142102 | 11 | 20755 | oar3_OAR12_2121348 | oar3_OAR12_2142102 |
| 12 | 2121348 | 2142102 | 11 | 20755 | oar3_OAR12_2121348 | oar3_OAR12_2142102 |
| 12 | 2491684 | 2529805 | 15 | 38122 | oar3_OAR12_2491684 | oar3_OAR12_2529805 |
| 12 | 2499928 | 2600680 | 23 | 100753 | oar3_OAR12_2499928 | oar3_OAR12_2600680 |
| 12 | 2499928 | 2600680 | 23 | 100753 | oar3_OAR12_2499928 | oar3_OAR12_2600680 |
| 12 | 2499928 | 2520423 | 9 | 20496 | oar3_OAR12_2499928 | oar3_OAR12_2520423 |
| 12 | 2503010 | 2529805 | 11 | 26796 | oar3_OAR12_2503010 | oar3_OAR12_2529805 |
| 12 | 2721797 | 2738615 | 5 | 16819 | oar3_OAR12_2721797 | oar3_OAR12_2738615 |
| 12 | 3519763 | 3648368 | 32 | 128606 | oar3_OAR12_3519763 | oar3_OAR12_3648368 |
| 12 | 3519763 | 3564584 | 14 | 44822 | oar3_OAR12_3519763 | oar3_OAR12_3564584 |
| 12 | 3519763 | 3648368 | 32 | 128606 | oar3_OAR12_3519763 | oar3_OAR12_3648368 |
| 12 | 3519763 | 3561178 | 13 | 41416 | oar3_OAR12_3519763 | oar3_OAR12_3561178 |
| 12 | 4099657 | 4100269 | 3 | 613 | oar3_OAR12_4099657 | oar3_OAR12_4100269 |
| 12 | 4388517 | 4418922 | 12 | 30406 | oar3_OAR12_4388517 | oar3_OAR12_4418922 |
| 12 | 4879090 | 4909916 | 5 | 30827 | oar3_OAR12_4879090 | oar3_OAR12_4909916 |
| 12 | 7416737 | 7421332 | 4 | 4596 | oar3_OAR12_7416737 | oar3_OAR12_7421332 |
| 12 | 7416737 | 7421332 | 4 | 4596 | oar3_OAR12_7416737 | oar3_OAR12_7421332 |
| 12 | 7416737 | 7421332 | 4 | 4596 | oar3_OAR12_7416737 | oar3_OAR12_7421332 |
| 12 | 11271315 | 11275528 | 4 | 4214 | oar3_OAR12_11271315 | oar3_OAR12_11275528 |
| 12 | 13672175 | 13678745 | 3 | 6571 | oar3_OAR12_13672175 | oar3_OAR12_13678745 |
| 12 | 25189663 | 25200516 | 12 | 10854 | oar3_OAR12_25189663 | oar3_OAR12_25200516 |
| 12 | 33156090 | 33156819 | 4 | 730 | oar3_OAR12_33156090 | oar3_OAR12_33156819 |
| 12 | 35195425 | 35200370 | 4 | 4946 | oar3_OAR12_35195425 | oar3_OAR12_35200370 |
| 12 | 35802920 | 35842247 | 7 | 39328 | oar3_OAR12_35802920 | OAR12_40042046.1 |
| 12 | 40019433 | 40033295 | 9 | 13863 | s30607.1 | oar3_OAR12_40033295 |
| 12 | 40023632 | 40042093 | 9 | 18462 | oar3_OAR12_40023632 | s27913.1 |
| 12 | 40023632 | 40042093 | 9 | 18462 | oar3_OAR12_40023632 | s27913.1 |
| 12 | 40023632 | 40042093 | 9 | 18462 | oar3_OAR12_40023632 | s27913.1 |
| 12 | 40023632 | 40044754 | 10 | 21123 | oar3_OAR12_40023632 | oar3_OAR12_40044754 |
| 12 | 40023632 | 40042093 | 9 | 18462 | oar3_OAR12_40023632 | s27913.1 |
| 12 | 40985572 | 41116411 | 34 | 130840 | s35634.1 | oar3_OAR12_41116411 |
| 12 | 41006699 | 41116411 | 29 | 109713 | oar3_OAR12_41006699 | oar3_OAR12_41116411 |
| 12 | 41006699 | 41076545 | 19 | 69847 | oar3_OAR12_41006699 | oar3_OAR12_41076545 |
| 12 | 41006699 | 41061405 | 13 | 54707 | oar3_OAR12_41006699 | oar3_OAR12_41061405 |
| 12 | 41006699 | 41061405 | 13 | 54707 | oar3_OAR12_41006699 | oar3_OAR12_41061405 |
| 12 | 41070303 | 41108437 | 14 | 38135 | oar3_OAR12_41070303 | oar3_OAR12_41108437 |
| 12 | 41070303 | 41108437 | 14 | 38135 | oar3_OAR12_41070303 | oar3_OAR12_41108437 |
| 12 | 41074647 | 41091645 | 8 | 16999 | oar3_OAR12_41074647 | oar3_OAR12_41091645 |
| 12 | 41076545 | 41108437 | 9 | 31893 | oar3_OAR12_41076545 | oar3_OAR12_41108437 |
| 12 | 41090571 | 41108437 | 7 | 17867 | oar3_OAR12_41090571 | oar3_OAR12_41108437 |
| 12 | 41090571 | 41108437 | 7 | 17867 | oar3_OAR12_41090571 | oar3_OAR12_41108437 |
| 12 | 41103886 | 41108437 | 4 | 4552 | oar3_OAR12_41103886 | oar3_OAR12_41108437 |
| 12 | 41927550 | 41972201 | 10 | 44652 | oar3_OAR12_41927550 | oar3_OAR12_41972201 |
| 12 | 41927550 | 41972201 | 10 | 44652 | oar3_OAR12_41927550 | oar3_OAR12_41972201 |
| 12 | 44888051 | 45051279 | 37 | 163229 | oar3_OAR12_44888051 | oar3_OAR12_45051279 |
| 12 | 44893586 | 45060153 | 37 | 166568 | oar3_OAR12_44893586 | oar3_OAR12_45060153 |
| 12 | 44908171 | 44937409 | 9 | 29239 | oar3_OAR12_44908171 | oar3_OAR12_44937409 |
| 12 | 44967486 | 45086911 | 27 | 119426 | oar3_OAR12_44967486 | oar3_OAR12_45086911 |
| 12 | 44984688 | 45011103 | 9 | 26416 | oar3_OAR12_44984688 | oar3_OAR12_45011103 |
| 12 | 44984688 | 45014344 | 10 | 29657 | oar3_OAR12_44984688 | oar3_OAR12_45014344 |
| 12 | 44984688 | 45014344 | 10 | 29657 | oar3_OAR12_44984688 | oar3_OAR12_45014344 |
| 12 | 44993883 | 45011103 | 7 | 17221 | oar3_OAR12_44993883 | oar3_OAR12_45011103 |
| 12 | 45004187 | 45011103 | 4 | 6917 | s05989.1 | oar3_OAR12_45011103 |
| 12 | 45108155 | 45198676 | 24 | 90522 | oar3_OAR12_45108155 | oar3_OAR12_45198676 |
| 12 | 45123856 | 45402829 | 60 | 278974 | oar3_OAR12_45123856 | oar3_OAR12_45402829 |
| 12 | 45158493 | 45198676 | 14 | 40184 | oar3_OAR12_45158493 | oar3_OAR12_45198676 |
| 12 | 45181348 | 45208922 | 7 | 27575 | oar3_OAR12_45181348 | oar3_OAR12_45208922 |
| 12 | 45193506 | 45230745 | 6 | 37240 | oar3_OAR12_45193506 | oar3_OAR12_45230745 |
| 12 | 45293253 | 45419733 | 29 | 126481 | oar3_OAR12_45293253 | oar3_OAR12_45419733 |
| 12 | 45330316 | 45362075 | 9 | 31760 | oar3_OAR12_45330316 | oar3_OAR12_45362075 |
| 12 | 45330316 | 45335456 | 4 | 5141 | oar3_OAR12_45330316 | oar3_OAR12_45335456 |
| 12 | 45330316 | 45362075 | 9 | 31760 | oar3_OAR12_45330316 | oar3_OAR12_45362075 |
| 12 | 45330316 | 45457186 | 37 | 126871 | oar3_OAR12_45330316 | oar3_OAR12_45457186 |
| 12 | 45536262 | 45569694 | 12 | 33433 | oar3_OAR12_45536262 | oar3_OAR12_45569694 |
| 12 | 45536262 | 45580806 | 15 | 44545 | oar3_OAR12_45536262 | oar3_OAR12_45580806 |
| 12 | 45536262 | 45580806 | 15 | 44545 | oar3_OAR12_45536262 | oar3_OAR12_45580806 |
| 12 | 45536262 | 45580806 | 15 | 44545 | oar3_OAR12_45536262 | oar3_OAR12_45580806 |
| 12 | 47312033 | 47382942 | 25 | 70910 | oar3_OAR12_47312033 | oar3_OAR12_47382942 |
| 12 | 47312033 | 47352865 | 13 | 40833 | oar3_OAR12_47312033 | oar3_OAR12_47352865 |
| 12 | 47312033 | 47663859 | 86 | 351827 | oar3_OAR12_47312033 | s56642.1 |
| 12 | 47312033 | 47422012 | 33 | 109980 | oar3_OAR12_47312033 | oar3_OAR12_47422012 |
| 12 | 47313035 | 47327495 | 4 | 14461 | oar3_OAR12_47313035 | oar3_OAR12_47327495 |
| 12 | 47373735 | 47425511 | 22 | 51777 | oar3_OAR12_47373735 | oar3_OAR12_47425511 |
| 12 | 47379555 | 47382942 | 8 | 3388 | oar3_OAR12_47379555 | oar3_OAR12_47382942 |
| 12 | 47379555 | 47382942 | 8 | 3388 | oar3_OAR12_47379555 | oar3_OAR12_47382942 |
| 12 | 47457266 | 47526102 | 9 | 68837 | s05958.1 | oar3_OAR12_47526102 |
| 12 | 47559317 | 47694493 | 39 | 135177 | oar3_OAR12_47559317 | oar3_OAR12_47694493 |
| 12 | 47566434 | 47754647 | 47 | 188214 | s45117.1 | oar3_OAR12_47754647 |
| 12 | 47585007 | 47662564 | 22 | 77558 | oar3_OAR12_47585007 | oar3_OAR12_47662564 |
| 12 | 47606949 | 47787618 | 42 | 180670 | oar3_OAR12_47606949 | oar3_OAR12_47787618 |
| 12 | 47610936 | 47682659 | 21 | 71724 | s53319.1 | oar3_OAR12_47682659 |
| 12 | 47610936 | 47733750 | 29 | 122815 | s53319.1 | oar3_OAR12_47733750 |
| 12 | 47617911 | 47662564 | 14 | 44654 | oar3_OAR12_47617911 | oar3_OAR12_47662564 |
| 12 | 47634712 | 47662564 | 10 | 27853 | oar3_OAR12_47634712 | oar3_OAR12_47662564 |
| 12 | 47649947 | 47656780 | 5 | 6834 | oar3_OAR12_47649947 | oar3_OAR12_47656780 |
| 12 | 47649947 | 47663859 | 7 | 13913 | oar3_OAR12_47649947 | s56642.1 |
| 12 | 47676570 | 47766485 | 18 | 89916 | oar3_OAR12_47676570 | oar3_OAR12_47766485 |
| 12 | 47676570 | 47694493 | 8 | 17924 | oar3_OAR12_47676570 | oar3_OAR12_47694493 |
| 12 | 47676570 | 47787618 | 22 | 111049 | oar3_OAR12_47676570 | oar3_OAR12_47787618 |
| 12 | 47752206 | 47766485 | 6 | 14280 | oar3_OAR12_47752206 | oar3_OAR12_47766485 |
| 12 | 47914023 | 47933926 | 7 | 19904 | oar3_OAR12_47914023 | oar3_OAR12_47933926 |
| 12 | 47927464 | 47980776 | 17 | 53313 | oar3_OAR12_47927464 | oar3_OAR12_47980776 |
| 12 | 47927914 | 48060405 | 30 | 132492 | oar3_OAR12_47927914 | oar3_OAR12_48060405 |
| 12 | 47965535 | 47999887 | 11 | 34353 | oar3_OAR12_47965535 | oar3_OAR12_47999887 |
| 12 | 47967601 | 48060405 | 21 | 92805 | oar3_OAR12_47967601 | oar3_OAR12_48060405 |
| 12 | 47988439 | 48060405 | 14 | 71967 | oar3_OAR12_47988439 | oar3_OAR12_48060405 |
| 12 | 48072312 | 48083129 | 4 | 10818 | s15193.1 | oar3_OAR12_48083129 |
| 12 | 48189224 | 48475147 | 67 | 285924 | oar3_OAR12_48189224 | oar3_OAR12_48475147 |
| 12 | 48189224 | 48230605 | 14 | 41382 | oar3_OAR12_48189224 | oar3_OAR12_48230605 |
| 12 | 48211431 | 48230605 | 7 | 19175 | s70019.1 | oar3_OAR12_48230605 |
| 12 | 48258622 | 48412883 | 37 | 154262 | oar3_OAR12_48258622 | s45370.1 |
| 12 | 48258622 | 48367086 | 25 | 108465 | oar3_OAR12_48258622 | oar3_OAR12_48367086 |
| 12 | 48288842 | 48475147 | 40 | 186306 | s51614.1 | oar3_OAR12_48475147 |
| 12 | 48292646 | 48323762 | 7 | 31117 | oar3_OAR12_48292646 | oar3_OAR12_48323762 |
| 12 | 48302992 | 48361847 | 8 | 58856 | oar3_OAR12_48302992 | oar3_OAR12_48361847 |
| 12 | 48310162 | 48351358 | 5 | 41197 | oar3_OAR12_48310162 | oar3_OAR12_48351358 |
| 12 | 48310162 | 48323762 | 3 | 13601 | oar3_OAR12_48310162 | oar3_OAR12_48323762 |
| 12 | 48351358 | 48654056 | 63 | 302699 | oar3_OAR12_48351358 | oar3_OAR12_48654056 |
| 12 | 48351358 | 48412883 | 17 | 61526 | oar3_OAR12_48351358 | s45370.1 |
| 12 | 48457012 | 48647970 | 39 | 190959 | oar3_OAR12_48457012 | oar3_OAR12_48647970 |
| 12 | 48457012 | 48475147 | 7 | 18136 | oar3_OAR12_48457012 | oar3_OAR12_48475147 |
| 12 | 48463533 | 48475147 | 5 | 11615 | oar3_OAR12_48463533 | oar3_OAR12_48475147 |
| 12 | 48463533 | 48475147 | 5 | 11615 | oar3_OAR12_48463533 | oar3_OAR12_48475147 |
| 12 | 48466546 | 48593143 | 28 | 126598 | oar3_OAR12_48466546 | oar3_OAR12_48593143 |
| 12 | 48505975 | 48654056 | 30 | 148082 | oar3_OAR12_48505975 | oar3_OAR12_48654056 |
| 12 | 48530979 | 48634508 | 22 | 103530 | oar3_OAR12_48530979 | oar3_OAR12_48634508 |
| 12 | 48537071 | 48634508 | 21 | 97438 | s65171.1 | oar3_OAR12_48634508 |
| 12 | 48539835 | 48566982 | 8 | 27148 | oar3_OAR12_48539835 | oar3_OAR12_48566982 |
| 12 | 48552470 | 48597260 | 8 | 44791 | oar3_OAR12_48552470 | oar3_OAR12_48597260 |
| 12 | 48556424 | 48647970 | 14 | 91547 | oar3_OAR12_48556424 | oar3_OAR12_48647970 |
| 12 | 48556424 | 48647970 | 14 | 91547 | oar3_OAR12_48556424 | oar3_OAR12_48647970 |
| 12 | 48556424 | 48654056 | 15 | 97633 | oar3_OAR12_48556424 | oar3_OAR12_48654056 |
| 12 | 48566982 | 48654056 | 14 | 87075 | oar3_OAR12_48566982 | oar3_OAR12_48654056 |
| 12 | 48566982 | 48647970 | 13 | 80989 | oar3_OAR12_48566982 | oar3_OAR12_48647970 |
| 12 | 48850939 | 48935792 | 12 | 84854 | oar3_OAR12_48850939 | oar3_OAR12_48935792 |
| 12 | 48850939 | 48896529 | 7 | 45591 | oar3_OAR12_48850939 | oar3_OAR12_48896529 |
| 12 | 48850939 | 49005423 | 24 | 154485 | oar3_OAR12_48850939 | oar3_OAR12_49005423 |
| 12 | 48850939 | 48896529 | 7 | 45591 | oar3_OAR12_48850939 | oar3_OAR12_48896529 |
| 12 | 48979139 | 49073457 | 13 | 94319 | oar3_OAR12_48979139 | s40386.1 |
| 12 | 49104120 | 49311151 | 19 | 207032 | oar3_OAR12_49104120 | oar3_OAR12_49311151 |
| 12 | 49104120 | 49311151 | 19 | 207032 | oar3_OAR12_49104120 | oar3_OAR12_49311151 |
| 12 | 49104120 | 49537920 | 55 | 433801 | oar3_OAR12_49104120 | oar3_OAR12_49537920 |
| 12 | 49270130 | 49406856 | 18 | 136727 | s75397.1 | oar3_OAR12_49406856 |
| 12 | 49304631 | 49311151 | 4 | 6521 | oar3_OAR12_49304631 | oar3_OAR12_49311151 |
| 12 | 49304631 | 49311151 | 4 | 6521 | oar3_OAR12_49304631 | oar3_OAR12_49311151 |
| 12 | 49304631 | 49308097 | 3 | 3467 | oar3_OAR12_49304631 | oar3_OAR12_49308097 |
| 12 | 49304631 | 49308097 | 3 | 3467 | oar3_OAR12_49304631 | oar3_OAR12_49308097 |
| 12 | 49304631 | 49327042 | 7 | 22412 | oar3_OAR12_49304631 | oar3_OAR12_49327042 |
| 12 | 49304631 | 49311151 | 4 | 6521 | oar3_OAR12_49304631 | oar3_OAR12_49311151 |
| 12 | 49304631 | 49311151 | 4 | 6521 | oar3_OAR12_49304631 | oar3_OAR12_49311151 |
| 12 | 49304631 | 49311151 | 4 | 6521 | oar3_OAR12_49304631 | oar3_OAR12_49311151 |
| 12 | 49304631 | 49488765 | 28 | 184135 | oar3_OAR12_49304631 | oar3_OAR12_49488765 |
| 12 | 49311151 | 49472884 | 22 | 161734 | oar3_OAR12_49311151 | oar3_OAR12_49472884 |
| 12 | 49471209 | 49494173 | 6 | 22965 | s00229.1 | oar3_OAR12_49494173 |
| 12 | 49472884 | 49507607 | 12 | 34724 | oar3_OAR12_49472884 | oar3_OAR12_49507607 |
| 12 | 49501032 | 49603494 | 16 | 102463 | oar3_OAR12_49501032 | oar3_OAR12_49603494 |
| 12 | 49857648 | 49901432 | 14 | 43785 | oar3_OAR12_49857648 | oar3_OAR12_49901432 |
| 12 | 49857648 | 49901432 | 14 | 43785 | oar3_OAR12_49857648 | oar3_OAR12_49901432 |
| 12 | 49880602 | 49890260 | 5 | 9659 | oar3_OAR12_49880602 | oar3_OAR12_49890260 |
| 12 | 60110268 | 60124631 | 9 | 14364 | oar3_OAR12_60110268 | oar3_OAR12_60124631 |
| 12 | 60110268 | 60138660 | 18 | 28393 | oar3_OAR12_60110268 | oar3_OAR12_60138660 |
| 12 | 60111965 | 60152013 | 20 | 40049 | oar3_OAR12_60111965 | oar3_OAR12_60152013 |
| 12 | 60111965 | 60138660 | 17 | 26696 | oar3_OAR12_60111965 | oar3_OAR12_60138660 |
| 12 | 60133036 | 60138660 | 7 | 5625 | oar3_OAR12_60133036 | oar3_OAR12_60138660 |
| 12 | 60133036 | 60138660 | 7 | 5625 | oar3_OAR12_60133036 | oar3_OAR12_60138660 |
| 12 | 60137122 | 60152013 | 8 | 14892 | oar3_OAR12_60137122 | oar3_OAR12_60152013 |
| 12 | 60137122 | 60138660 | 5 | 1539 | oar3_OAR12_60137122 | oar3_OAR12_60138660 |
| 12 | 60137122 | 60138660 | 5 | 1539 | oar3_OAR12_60137122 | oar3_OAR12_60138660 |
| 12 | 69211215 | 69269910 | 19 | 58696 | oar3_OAR12_69211215 | s26719.1 |
| 12 | 77151593 | 77227752 | 22 | 76160 | oar3_OAR12_77151593 | oar3_OAR12_77227752 |
| 12 | 77353500 | 77402823 | 27 | 49324 | oar3_OAR12_77353500 | s49528.1 |
| 12 | 77353500 | 77378830 | 15 | 25331 | oar3_OAR12_77353500 | oar3_OAR12_77378830 |
| 12 | 77562638 | 79070188 | 230 | 1507551 | oar3_OAR12_77562638 | oar3_OAR12_79070188 |
| 12 | 77562638 | 77590625 | 8 | 27988 | oar3_OAR12_77562638 | s36170.1 |
| 12 | 77562638 | 77590625 | 8 | 27988 | oar3_OAR12_77562638 | s36170.1 |
| 12 | 77562638 | 77769961 | 44 | 207324 | oar3_OAR12_77562638 | oar3_OAR12_77769961 |
| 12 | 77585432 | 77715116 | 27 | 129685 | oar3_OAR12_77585432 | oar3_OAR12_77715116 |
| 12 | 77606200 | 77678588 | 16 | 72389 | oar3_OAR12_77606200 | oar3_OAR12_77678588 |
| 12 | 77615915 | 77849417 | 45 | 233503 | oar3_OAR12_77615915 | s01727.1 |
| 12 | 77615915 | 77792634 | 36 | 176720 | oar3_OAR12_77615915 | oar3_OAR12_77792634 |
| 12 | 77623571 | 77760497 | 29 | 136927 | oar3_OAR12_77623571 | oar3_OAR12_77760497 |
| 12 | 77623571 | 77673553 | 12 | 49983 | oar3_OAR12_77623571 | oar3_OAR12_77673553 |
| 12 | 77632337 | 77662800 | 9 | 30464 | oar3_OAR12_77632337 | oar3_OAR12_77662800 |
| 12 | 77636079 | 77678588 | 9 | 42510 | oar3_OAR12_77636079 | oar3_OAR12_77678588 |
| 12 | 77646107 | 77678588 | 7 | 32482 | oar3_OAR12_77646107 | oar3_OAR12_77678588 |
| 12 | 77657266 | 77868570 | 37 | 211305 | oar3_OAR12_77657266 | oar3_OAR12_77868570 |
| 12 | 77721294 | 77792634 | 15 | 71341 | oar3_OAR12_77721294 | oar3_OAR12_77792634 |
| 12 | 77736126 | 77787452 | 9 | 51327 | oar3_OAR12_77736126 | oar3_OAR12_77787452 |
| 12 | 77742766 | 77912057 | 30 | 169292 | oar3_OAR12_77742766 | oar3_OAR12_77912057 |
| 12 | 77877008 | 77912057 | 9 | 35050 | oar3_OAR12_77877008 | oar3_OAR12_77912057 |
| 12 | 77885622 | 77912057 | 5 | 26436 | s19641.1 | oar3_OAR12_77912057 |
| 12 | 77984201 | 78036908 | 15 | 52708 | oar3_OAR12_77984201 | oar3_OAR12_78036908 |
| 12 | 77984201 | 78042737 | 19 | 58537 | oar3_OAR12_77984201 | oar3_OAR12_78042737 |
| 12 | 77984201 | 78038682 | 17 | 54482 | oar3_OAR12_77984201 | oar3_OAR12_78038682 |
| 12 | 78018853 | 78038682 | 6 | 19830 | oar3_OAR12_78018853 | oar3_OAR12_78038682 |
| 12 | 78103892 | 79070188 | 118 | 966297 | oar3_OAR12_78103892 | oar3_OAR12_79070188 |
| 12 | 78111323 | 78198927 | 20 | 87605 | oar3_OAR12_78111323 | oar3_OAR12_78198927 |
| 12 | 78164219 | 78168737 | 4 | 4519 | oar3_OAR12_78164219 | oar3_OAR12_78168737 |
| 12 | 78164219 | 79070188 | 103 | 905970 | oar3_OAR12_78164219 | oar3_OAR12_79070188 |
| 12 | 78266900 | 79070188 | 90 | 803289 | oar3_OAR12_78266900 | oar3_OAR12_79070188 |
| 12 | 78321769 | 78456686 | 24 | 134918 | oar3_OAR12_78321769 | oar3_OAR12_78456686 |
| 12 | 78321769 | 79070188 | 87 | 748420 | oar3_OAR12_78321769 | oar3_OAR12_79070188 |
| 12 | 78384686 | 78433202 | 12 | 48517 | oar3_OAR12_78384686 | oar3_OAR12_78433202 |
| 12 | 78390080 | 79070188 | 76 | 680109 | oar3_OAR12_78390080 | oar3_OAR12_79070188 |
| 12 | 78395361 | 79070188 | 75 | 674828 | oar3_OAR12_78395361 | oar3_OAR12_79070188 |
| 12 | 78395361 | 79070188 | 75 | 674828 | oar3_OAR12_78395361 | oar3_OAR12_79070188 |
| 12 | 78395361 | 78613305 | 22 | 217945 | oar3_OAR12_78395361 | oar3_OAR12_78613305 |
| 12 | 78399491 | 78456686 | 11 | 57196 | oar3_OAR12_78399491 | oar3_OAR12_78456686 |
| 12 | 78399491 | 78436511 | 9 | 37021 | oar3_OAR12_78399491 | oar3_OAR12_78436511 |
| 12 | 78412601 | 78449224 | 8 | 36624 | oar3_OAR12_78412601 | oar3_OAR12_78449224 |
| 12 | 78456686 | 79070188 | 64 | 613503 | oar3_OAR12_78456686 | oar3_OAR12_79070188 |
| 12 | 78783092 | 78815501 | 8 | 32410 | oar3_OAR12_78783092 | oar3_OAR12_78815501 |
| 12 | 78925714 | 78957972 | 6 | 32259 | s33621.1 | oar3_OAR12_78957972 |
| 12 | 79001383 | 79070188 | 6 | 68806 | oar3_OAR12_79001383 | oar3_OAR12_79070188 |
| 12 | 79057458 | 79070188 | 3 | 12731 | oar3_OAR12_79057458 | oar3_OAR12_79070188 |
| 13 | 3398767 | 3419981 | 9 | 21215 | oar3_OAR13_3398767 | oar3_OAR13_3419981 |
| 13 | 3415900 | 3419981 | 5 | 4082 | oar3_OAR13_3415900 | oar3_OAR13_3419981 |
| 13 | 3415900 | 3419981 | 5 | 4082 | oar3_OAR13_3415900 | oar3_OAR13_3419981 |
| 13 | 10615247 | 10618387 | 10 | 3141 | oar3_OAR13_10615247 | oar3_OAR13_10618387 |
| 13 | 10615247 | 10643358 | 16 | 28112 | oar3_OAR13_10615247 | oar3_OAR13_10643358 |
| 13 | 10615247 | 10618387 | 10 | 3141 | oar3_OAR13_10615247 | oar3_OAR13_10618387 |
| 13 | 10615247 | 10618387 | 10 | 3141 | oar3_OAR13_10615247 | oar3_OAR13_10618387 |
| 13 | 10674690 | 10688852 | 8 | 14163 | oar3_OAR13_10674690 | oar3_OAR13_10688852 |
| 13 | 12060907 | 12075388 | 6 | 14482 | oar3_OAR13_12060907 | s16039.1 |
| 13 | 21806857 | 21827247 | 5 | 20391 | s07619.1 | oar3_OAR13_21827247 |
| 13 | 22511949 | 22526188 | 4 | 14240 | oar3_OAR13_22511949 | OAR13_25068711.1 |
| 13 | 22522816 | 22526188 | 3 | 3373 | oar3_OAR13_22522816 | OAR13_25068711.1 |
| 13 | 22522816 | 22541301 | 4 | 18486 | oar3_OAR13_22522816 | oar3_OAR13_22541301 |
| 13 | 34112883 | 34121253 | 11 | 8371 | oar3_OAR13_34112883 | oar3_OAR13_34121253 |
| 13 | 34118520 | 34120422 | 6 | 1903 | oar3_OAR13_34118520 | oar3_OAR13_34120422 |
| 13 | 34118520 | 34120878 | 8 | 2359 | oar3_OAR13_34118520 | oar3_OAR13_34120878 |
| 13 | 38649024 | 38654562 | 4 | 5539 | oar3_OAR13_38649024 | oar3_OAR13_38654562 |
| 13 | 39780643 | 39782606 | 4 | 1964 | oar3_OAR13_39780643 | oar3_OAR13_39782606 |
| 13 | 40005790 | 40017177 | 8 | 11388 | oar3_OAR13_40005790 | oar3_OAR13_40017177 |
| 13 | 40709163 | 40718309 | 7 | 9147 | oar3_OAR13_40709163 | oar3_OAR13_40718309 |
| 13 | 40950072 | 40982284 | 10 | 32213 | oar3_OAR13_40950072 | oar3_OAR13_40982284 |
| 13 | 41204574 | 41215823 | 6 | 11250 | oar3_OAR13_41204574 | oar3_OAR13_41215823 |
| 13 | 41670795 | 41775594 | 27 | 104800 | oar3_OAR13_41670795 | oar3_OAR13_41775594 |
| 13 | 41700059 | 41775594 | 20 | 75536 | oar3_OAR13_41700059 | oar3_OAR13_41775594 |
| 13 | 42586361 | 42606978 | 3 | 20618 | oar3_OAR13_42586361 | oar3_OAR13_42606978 |
| 13 | 45283310 | 45294446 | 7 | 11137 | oar3_OAR13_45283310 | oar3_OAR13_45294446 |
| 13 | 45366948 | 45502830 | 41 | 135883 | oar3_OAR13_45366948 | oar3_OAR13_45502830 |
| 13 | 45428730 | 45502830 | 24 | 74101 | oar3_OAR13_45428730 | oar3_OAR13_45502830 |
| 13 | 45634081 | 45723202 | 32 | 89122 | oar3_OAR13_45634081 | oar3_OAR13_45723202 |
| 13 | 45705391 | 45723202 | 14 | 17812 | oar3_OAR13_45705391 | oar3_OAR13_45723202 |
| 13 | 50814842 | 50859710 | 22 | 44869 | oar3_OAR13_50814842 | oar3_OAR13_50859710 |
| 13 | 50814842 | 50859710 | 22 | 44869 | oar3_OAR13_50814842 | oar3_OAR13_50859710 |
| 13 | 50839437 | 50859710 | 8 | 20274 | oar3_OAR13_50839437 | oar3_OAR13_50859710 |
| 13 | 50839437 | 50870495 | 11 | 31059 | oar3_OAR13_50839437 | oar3_OAR13_50870495 |
| 13 | 51674060 | 51691730 | 7 | 17671 | oar3_OAR13_51674060 | oar3_OAR13_51691730 |
| 13 | 52308913 | 52332337 | 5 | 23425 | oar3_OAR13_52308913 | s30922.1 |
| 13 | 52622441 | 52627584 | 3 | 5144 | oar3_OAR13_52622441 | oar3_OAR13_52627584 |
| 13 | 53035139 | 53294265 | 21 | 259127 | oar3_OAR13_53035139 | oar3_OAR13_53294265 |
| 13 | 53173647 | 53669096 | 13 | 495450 | s66957.1 | oar3_OAR13_53669096 |
| 13 | 53173647 | 53294265 | 5 | 120619 | s66957.1 | oar3_OAR13_53294265 |
| 13 | 53173647 | 53669096 | 13 | 495450 | s66957.1 | oar3_OAR13_53669096 |
| 13 | 53173647 | 53294265 | 5 | 120619 | s66957.1 | oar3_OAR13_53294265 |
| 13 | 53173647 | 53294265 | 5 | 120619 | s66957.1 | oar3_OAR13_53294265 |
| 13 | 53173647 | 53669096 | 13 | 495450 | s66957.1 | oar3_OAR13_53669096 |
| 13 | 53173647 | 53669096 | 13 | 495450 | s66957.1 | oar3_OAR13_53669096 |
| 13 | 53208135 | 53669096 | 12 | 460962 | oar3_OAR13_53208135 | oar3_OAR13_53669096 |
| 13 | 53208135 | 53294265 | 4 | 86131 | oar3_OAR13_53208135 | oar3_OAR13_53294265 |
| 13 | 53208135 | 53669096 | 12 | 460962 | oar3_OAR13_53208135 | oar3_OAR13_53669096 |
| 13 | 53208135 | 53294265 | 4 | 86131 | oar3_OAR13_53208135 | oar3_OAR13_53294265 |
| 13 | 53208135 | 53810351 | 35 | 602217 | oar3_OAR13_53208135 | oar3_OAR13_53810351 |
| 13 | 53208135 | 53647951 | 9 | 439817 | oar3_OAR13_53208135 | oar3_OAR13_53647951 |
| 13 | 53632670 | 53651829 | 5 | 19160 | OAR13_58349162.1 | oar3_OAR13_53651829 |
| 13 | 53632670 | 53669096 | 7 | 36427 | OAR13_58349162.1 | oar3_OAR13_53669096 |
| 13 | 53632670 | 53651829 | 5 | 19160 | OAR13_58349162.1 | oar3_OAR13_53651829 |
| 13 | 53722094 | 53763103 | 8 | 41010 | s74462.1 | oar3_OAR13_53763103 |
| 13 | 53722094 | 53827335 | 20 | 105242 | s74462.1 | oar3_OAR13_53827335 |
| 13 | 53763103 | 53827335 | 13 | 64233 | oar3_OAR13_53763103 | oar3_OAR13_53827335 |
| 13 | 53763103 | 53827335 | 13 | 64233 | oar3_OAR13_53763103 | oar3_OAR13_53827335 |
| 13 | 53763103 | 53827335 | 13 | 64233 | oar3_OAR13_53763103 | oar3_OAR13_53827335 |
| 13 | 53773914 | 54083235 | 62 | 309322 | oar3_OAR13_53773914 | oar3_OAR13_54083235 |
| 13 | 53789339 | 53966077 | 32 | 176739 | oar3_OAR13_53789339 | oar3_OAR13_53966077 |
| 13 | 53807682 | 53819011 | 4 | 11330 | oar3_OAR13_53807682 | oar3_OAR13_53819011 |
| 13 | 53933954 | 54394650 | 96 | 460697 | oar3_OAR13_53933954 | oar3_OAR13_54394650 |
| 13 | 54015784 | 54083235 | 19 | 67452 | oar3_OAR13_54015784 | oar3_OAR13_54083235 |
| 13 | 54032485 | 54158427 | 31 | 125943 | oar3_OAR13_54032485 | oar3_OAR13_54158427 |
| 13 | 54032485 | 54371431 | 74 | 338947 | oar3_OAR13_54032485 | oar3_OAR13_54371431 |
| 13 | 54035784 | 54055331 | 5 | 19548 | oar3_OAR13_54035784 | oar3_OAR13_54055331 |
| 13 | 54055331 | 54135127 | 18 | 79797 | oar3_OAR13_54055331 | oar3_OAR13_54135127 |
| 13 | 54073910 | 54172009 | 24 | 98100 | oar3_OAR13_54073910 | OAR13_58925553.1 |
| 13 | 54096368 | 54158427 | 15 | 62060 | oar3_OAR13_54096368 | oar3_OAR13_54158427 |
| 13 | 54096368 | 54158427 | 15 | 62060 | oar3_OAR13_54096368 | oar3_OAR13_54158427 |
| 13 | 54096368 | 54162730 | 17 | 66363 | oar3_OAR13_54096368 | oar3_OAR13_54162730 |
| 13 | 54105999 | 54158427 | 14 | 52429 | oar3_OAR13_54105999 | oar3_OAR13_54158427 |
| 13 | 54112804 | 54249654 | 33 | 136851 | oar3_OAR13_54112804 | oar3_OAR13_54249654 |
| 13 | 54112804 | 54172009 | 19 | 59206 | oar3_OAR13_54112804 | OAR13_58925553.1 |
| 13 | 54134315 | 54184814 | 20 | 50500 | oar3_OAR13_54134315 | oar3_OAR13_54184814 |
| 13 | 54150311 | 54202746 | 19 | 52436 | oar3_OAR13_54150311 | oar3_OAR13_54202746 |
| 13 | 54152919 | 54230135 | 22 | 77217 | oar3_OAR13_54152919 | oar3_OAR13_54230135 |
| 13 | 54167841 | 54227966 | 14 | 60126 | oar3_OAR13_54167841 | oar3_OAR13_54227966 |
| 13 | 54223233 | 54342199 | 25 | 118967 | oar3_OAR13_54223233 | oar3_OAR13_54342199 |
| 13 | 54223233 | 54355577 | 27 | 132345 | oar3_OAR13_54223233 | oar3_OAR13_54355577 |
| 13 | 54223233 | 54249654 | 7 | 26422 | oar3_OAR13_54223233 | oar3_OAR13_54249654 |
| 13 | 54223233 | 54355577 | 27 | 132345 | oar3_OAR13_54223233 | oar3_OAR13_54355577 |
| 13 | 54223233 | 54371431 | 30 | 148199 | oar3_OAR13_54223233 | oar3_OAR13_54371431 |
| 13 | 54223233 | 54249654 | 7 | 26422 | oar3_OAR13_54223233 | oar3_OAR13_54249654 |
| 13 | 54223233 | 54249654 | 7 | 26422 | oar3_OAR13_54223233 | oar3_OAR13_54249654 |
| 13 | 54230135 | 54249654 | 4 | 19520 | oar3_OAR13_54230135 | oar3_OAR13_54249654 |
| 13 | 54308715 | 54342199 | 13 | 33485 | oar3_OAR13_54308715 | oar3_OAR13_54342199 |
| 13 | 54329744 | 54371431 | 12 | 41688 | oar3_OAR13_54329744 | oar3_OAR13_54371431 |
| 13 | 54438009 | 54480063 | 13 | 42055 | oar3_OAR13_54438009 | oar3_OAR13_54480063 |
| 13 | 54438009 | 54471235 | 12 | 33227 | oar3_OAR13_54438009 | oar3_OAR13_54471235 |
| 13 | 54458275 | 54496287 | 10 | 38013 | oar3_OAR13_54458275 | oar3_OAR13_54496287 |
| 13 | 59387896 | 59411249 | 7 | 23354 | oar3_OAR13_59387896 | oar3_OAR13_59411249 |
| 13 | 59686176 | 59761937 | 13 | 75762 | oar3_OAR13_59686176 | oar3_OAR13_59761937 |
| 13 | 59740445 | 59761937 | 7 | 21493 | oar3_OAR13_59740445 | oar3_OAR13_59761937 |
| 13 | 59869770 | 59925126 | 9 | 55357 | oar3_OAR13_59869770 | oar3_OAR13_59925126 |
| 13 | 60432575 | 60496530 | 14 | 63956 | s70152.1 | oar3_OAR13_60496530 |
| 13 | 61175104 | 61213750 | 12 | 38647 | oar3_OAR13_61175104 | oar3_OAR13_61213750 |
| 13 | 61191695 | 61204853 | 6 | 13159 | oar3_OAR13_61191695 | oar3_OAR13_61204853 |
| 13 | 61191695 | 61213750 | 9 | 22056 | oar3_OAR13_61191695 | oar3_OAR13_61213750 |
| 13 | 61204853 | 61345823 | 33 | 140971 | oar3_OAR13_61204853 | oar3_OAR13_61345823 |
| 13 | 61262154 | 61305745 | 12 | 43592 | oar3_OAR13_61262154 | oar3_OAR13_61305745 |
| 13 | 61270203 | 61344167 | 14 | 73965 | oar3_OAR13_61270203 | oar3_OAR13_61344167 |
| 13 | 61541954 | 61564578 | 9 | 22625 | oar3_OAR13_61541954 | oar3_OAR13_61564578 |
| 13 | 61541954 | 61542306 | 3 | 353 | oar3_OAR13_61541954 | oar3_OAR13_61542306 |
| 13 | 62546975 | 62578536 | 7 | 31562 | oar3_OAR13_62546975 | oar3_OAR13_62578536 |
| 13 | 62546975 | 62578536 | 7 | 31562 | oar3_OAR13_62546975 | oar3_OAR13_62578536 |
| 13 | 63841217 | 63881639 | 10 | 40423 | oar3_OAR13_63841217 | OAR13_68959441.1 |
| 13 | 63854385 | 63881639 | 6 | 27255 | oar3_OAR13_63854385 | OAR13_68959441.1 |
| 13 | 64886116 | 64899241 | 7 | 13126 | oar3_OAR13_64886116 | oar3_OAR13_64899241 |
| 13 | 65873700 | 65903030 | 7 | 29331 | oar3_OAR13_65873700 | oar3_OAR13_65903030 |
| 13 | 65887795 | 65905470 | 7 | 17676 | oar3_OAR13_65887795 | oar3_OAR13_65905470 |
| 13 | 66349766 | 66422625 | 18 | 72860 | oar3_OAR13_66349766 | oar3_OAR13_66422625 |
| 13 | 66357072 | 66422625 | 16 | 65554 | oar3_OAR13_66357072 | oar3_OAR13_66422625 |
| 13 | 69557938 | 69605299 | 12 | 47362 | oar3_OAR13_69557938 | oar3_OAR13_69605299 |
| 13 | 69557938 | 69580083 | 7 | 22146 | oar3_OAR13_69557938 | oar3_OAR13_69580083 |
| 13 | 69557938 | 69614210 | 15 | 56273 | oar3_OAR13_69557938 | oar3_OAR13_69614210 |
| 13 | 69557938 | 69614210 | 15 | 56273 | oar3_OAR13_69557938 | oar3_OAR13_69614210 |
| 13 | 69561417 | 69580083 | 5 | 18667 | oar3_OAR13_69561417 | oar3_OAR13_69580083 |
| 13 | 69561417 | 69580083 | 5 | 18667 | oar3_OAR13_69561417 | oar3_OAR13_69580083 |
| 13 | 72459702 | 72465341 | 6 | 5640 | oar3_OAR13_72459702 | oar3_OAR13_72465341 |
| 13 | 73129416 | 73209107 | 19 | 79692 | oar3_OAR13_73129416 | oar3_OAR13_73209107 |
| 13 | 73139706 | 73209107 | 17 | 69402 | oar3_OAR13_73139706 | oar3_OAR13_73209107 |
| 13 | 73139706 | 73161040 | 6 | 21335 | oar3_OAR13_73139706 | oar3_OAR13_73161040 |
| 13 | 74177321 | 74321307 | 31 | 143987 | oar3_OAR13_74177321 | oar3_OAR13_74321307 |
| 13 | 74202720 | 74321307 | 25 | 118588 | oar3_OAR13_74202720 | oar3_OAR13_74321307 |
| 13 | 74230538 | 74278793 | 7 | 48256 | oar3_OAR13_74230538 | oar3_OAR13_74278793 |
| 13 | 76485269 | 76532438 | 10 | 47170 | oar3_OAR13_76485269 | oar3_OAR13_76532438 |
| 13 | 78114920 | 78189312 | 15 | 74393 | oar3_OAR13_78114920 | s31649.1 |
| 13 | 78321656 | 78339356 | 8 | 17701 | oar3_OAR13_78321656 | oar3_OAR13_78339356 |
| 13 | 78321656 | 78339356 | 8 | 17701 | oar3_OAR13_78321656 | oar3_OAR13_78339356 |
| 13 | 78374859 | 78475773 | 26 | 100915 | oar3_OAR13_78374859 | oar3_OAR13_78475773 |
| 13 | 78443373 | 78475773 | 12 | 32401 | oar3_OAR13_78443373 | oar3_OAR13_78475773 |
| 13 | 78751082 | 78752389 | 3 | 1308 | oar3_OAR13_78751082 | oar3_OAR13_78752389 |
| 13 | 78887715 | 78913524 | 8 | 25810 | oar3_OAR13_78887715 | oar3_OAR13_78913524 |
| 13 | 78935598 | 78982806 | 10 | 47209 | oar3_OAR13_78935598 | oar3_OAR13_78982806 |
| 13 | 78965201 | 78982806 | 4 | 17606 | oar3_OAR13_78965201 | oar3_OAR13_78982806 |
| 13 | 78965201 | 78982806 | 4 | 17606 | oar3_OAR13_78965201 | oar3_OAR13_78982806 |
| 13 | 81219980 | 81248271 | 9 | 28292 | oar3_OAR13_81219980 | oar3_OAR13_81248271 |
| 13 | 81593932 | 81629202 | 13 | 35271 | oar3_OAR13_81593932 | oar3_OAR13_81629202 |
| 14 | 10294 | 67713 | 3 | 57420 | oar3_OAR14_10294 | oar3_OAR14_67713 |
| 14 | 573970 | 627999 | 17 | 54030 | oar3_OAR14_573970 | oar3_OAR14_627999 |
| 14 | 695356 | 707146 | 7 | 11791 | oar3_OAR14_695356 | oar3_OAR14_707146 |
| 14 | 832575 | 906125 | 11 | 73551 | oar3_OAR14_832575 | oar3_OAR14_906125 |
| 14 | 875672 | 889311 | 5 | 13640 | OAR14_1314896_X.1 | oar3_OAR14_889311 |
| 14 | 875672 | 889311 | 5 | 13640 | OAR14_1314896_X.1 | oar3_OAR14_889311 |
| 14 | 887169 | 889311 | 4 | 2143 | oar3_OAR14_887169 | oar3_OAR14_889311 |
| 14 | 1027381 | 1047677 | 8 | 20297 | oar3_OAR14_1027381 | oar3_OAR14_1047677 |
| 14 | 1033489 | 1047677 | 5 | 14189 | oar3_OAR14_1033489 | oar3_OAR14_1047677 |
| 14 | 1033489 | 1047677 | 5 | 14189 | oar3_OAR14_1033489 | oar3_OAR14_1047677 |
| 14 | 1180711 | 1302274 | 34 | 121564 | oar3_OAR14_1180711 | oar3_OAR14_1302274 |
| 14 | 1186098 | 1320060 | 39 | 133963 | oar3_OAR14_1186098 | oar3_OAR14_1320060 |
| 14 | 1186098 | 1247097 | 23 | 61000 | oar3_OAR14_1186098 | oar3_OAR14_1247097 |
| 14 | 1196132 | 1230902 | 15 | 34771 | oar3_OAR14_1196132 | oar3_OAR14_1230902 |
| 14 | 1214803 | 1230230 | 6 | 15428 | oar3_OAR14_1214803 | oar3_OAR14_1230230 |
| 14 | 1221926 | 1302274 | 17 | 80349 | oar3_OAR14_1221926 | oar3_OAR14_1302274 |
| 14 | 1227378 | 1320060 | 21 | 92683 | OAR14_1671498.1 | oar3_OAR14_1320060 |
| 14 | 1230902 | 1302274 | 13 | 71373 | oar3_OAR14_1230902 | oar3_OAR14_1302274 |
| 14 | 1287873 | 1306323 | 5 | 18451 | oar3_OAR14_1287873 | oar3_OAR14_1306323 |
| 14 | 1529954 | 1564584 | 9 | 34631 | oar3_OAR14_1529954 | oar3_OAR14_1564584 |
| 14 | 1529954 | 1564584 | 9 | 34631 | oar3_OAR14_1529954 | oar3_OAR14_1564584 |
| 14 | 1929266 | 1984905 | 15 | 55640 | oar3_OAR14_1929266 | oar3_OAR14_1984905 |
| 14 | 1929266 | 1984905 | 15 | 55640 | oar3_OAR14_1929266 | oar3_OAR14_1984905 |
| 14 | 1929266 | 1984905 | 15 | 55640 | oar3_OAR14_1929266 | oar3_OAR14_1984905 |
| 14 | 1937666 | 1984905 | 13 | 47240 | oar3_OAR14_1937666 | oar3_OAR14_1984905 |
| 14 | 1955256 | 1976002 | 10 | 20747 | oar3_OAR14_1955256 | s15674.1 |
| 14 | 1955256 | 1984905 | 11 | 29650 | oar3_OAR14_1955256 | oar3_OAR14_1984905 |
| 14 | 1955256 | 1984905 | 11 | 29650 | oar3_OAR14_1955256 | oar3_OAR14_1984905 |
| 14 | 1955256 | 1984905 | 11 | 29650 | oar3_OAR14_1955256 | oar3_OAR14_1984905 |
| 14 | 1973512 | 1984905 | 6 | 11394 | oar3_OAR14_1973512 | oar3_OAR14_1984905 |
| 14 | 1973512 | 1976002 | 5 | 2491 | oar3_OAR14_1973512 | s15674.1 |
| 14 | 3795957 | 3827787 | 10 | 31831 | oar3_OAR14_3795957 | oar3_OAR14_3827787 |
| 14 | 3795957 | 3813758 | 8 | 17802 | oar3_OAR14_3795957 | s30966.1 |
| 14 | 3849633 | 3853116 | 3 | 3484 | oar3_OAR14_3849633 | oar3_OAR14_3853116 |
| 14 | 5538455 | 5568858 | 13 | 30404 | oar3_OAR14_5538455 | oar3_OAR14_5568858 |
| 14 | 5548812 | 5568858 | 10 | 20047 | oar3_OAR14_5548812 | oar3_OAR14_5568858 |
| 14 | 5929340 | 5932586 | 5 | 3247 | oar3_OAR14_5929340 | oar3_OAR14_5932586 |
| 14 | 5929340 | 5932586 | 5 | 3247 | oar3_OAR14_5929340 | oar3_OAR14_5932586 |
| 14 | 7276845 | 7282380 | 3 | 5536 | oar3_OAR14_7276845 | oar3_OAR14_7282380 |
| 14 | 7515845 | 7646144 | 31 | 130300 | oar3_OAR14_7515845 | oar3_OAR14_7646144 |
| 14 | 7515845 | 7618594 | 23 | 102750 | oar3_OAR14_7515845 | oar3_OAR14_7618594 |
| 14 | 7819058 | 7869073 | 18 | 50016 | oar3_OAR14_7819058 | oar3_OAR14_7869073 |
| 14 | 7842613 | 7842732 | 3 | 120 | oar3_OAR14_7842613 | oar3_OAR14_7842732 |
| 14 | 10154272 | 10175901 | 9 | 21630 | oar3_OAR14_10154272 | oar3_OAR14_10175901 |
| 14 | 10162718 | 10217988 | 22 | 55271 | oar3_OAR14_10162718 | oar3_OAR14_10217988 |
| 14 | 10512755 | 10697127 | 51 | 184373 | oar3_OAR14_10512755 | oar3_OAR14_10697127 |
| 14 | 10600795 | 10635781 | 15 | 34987 | oar3_OAR14_10600795 | oar3_OAR14_10635781 |
| 14 | 10773826 | 10844811 | 17 | 70986 | oar3_OAR14_10773826 | oar3_OAR14_10844811 |
| 14 | 10773826 | 11046625 | 50 | 272800 | oar3_OAR14_10773826 | oar3_OAR14_11046625 |
| 14 | 10773826 | 10912371 | 31 | 138546 | oar3_OAR14_10773826 | oar3_OAR14_10912371 |
| 14 | 10773826 | 11275510 | 102 | 501685 | oar3_OAR14_10773826 | oar3_OAR14_11275510 |
| 14 | 10773826 | 10874369 | 23 | 100544 | oar3_OAR14_10773826 | oar3_OAR14_10874369 |
| 14 | 10877678 | 10908470 | 7 | 30793 | oar3_OAR14_10877678 | oar3_OAR14_10908470 |
| 14 | 10877678 | 11133766 | 42 | 256089 | oar3_OAR14_10877678 | oar3_OAR14_11133766 |
| 14 | 10888492 | 10899756 | 5 | 11265 | oar3_OAR14_10888492 | s53513.1 |
| 14 | 10888492 | 10912371 | 7 | 23880 | oar3_OAR14_10888492 | oar3_OAR14_10912371 |
| 14 | 10888492 | 10899756 | 5 | 11265 | oar3_OAR14_10888492 | s53513.1 |
| 14 | 10888492 | 10975657 | 16 | 87166 | oar3_OAR14_10888492 | oar3_OAR14_10975657 |
| 14 | 10888492 | 10959500 | 15 | 71009 | oar3_OAR14_10888492 | oar3_OAR14_10959500 |
| 14 | 10894027 | 10899756 | 3 | 5730 | oar3_OAR14_10894027 | s53513.1 |
| 14 | 10896299 | 10959500 | 12 | 63202 | oar3_OAR14_10896299 | oar3_OAR14_10959500 |
| 14 | 11032187 | 11142721 | 23 | 110535 | oar3_OAR14_11032187 | oar3_OAR14_11142721 |
| 14 | 11085350 | 11122745 | 8 | 37396 | oar3_OAR14_11085350 | oar3_OAR14_11122745 |
| 14 | 12099770 | 12156890 | 16 | 57121 | oar3_OAR14_12099770 | s54654.1 |
| 14 | 12664356 | 12728354 | 16 | 63999 | oar3_OAR14_12664356 | oar3_OAR14_12728354 |
| 14 | 12682987 | 12689973 | 4 | 6987 | oar3_OAR14_12682987 | oar3_OAR14_12689973 |
| 14 | 12768369 | 12807928 | 13 | 39560 | oar3_OAR14_12768369 | oar3_OAR14_12807928 |
| 14 | 12837860 | 12855235 | 10 | 17376 | oar3_OAR14_12837860 | oar3_OAR14_12855235 |
| 14 | 12842546 | 12860977 | 10 | 18432 | oar3_OAR14_12842546 | oar3_OAR14_12860977 |
| 14 | 12847451 | 12860977 | 8 | 13527 | oar3_OAR14_12847451 | oar3_OAR14_12860977 |
| 14 | 12944301 | 12962297 | 9 | 17997 | oar3_OAR14_12944301 | oar3_OAR14_12962297 |
| 14 | 12944301 | 12975255 | 11 | 30955 | oar3_OAR14_12944301 | oar3_OAR14_12975255 |
| 14 | 12944301 | 13010000 | 19 | 65700 | oar3_OAR14_12944301 | oar3_OAR14_13010000 |
| 14 | 12944301 | 13010000 | 19 | 65700 | oar3_OAR14_12944301 | oar3_OAR14_13010000 |
| 14 | 12955343 | 12962297 | 5 | 6955 | oar3_OAR14_12955343 | oar3_OAR14_12962297 |
| 14 | 13134280 | 13227221 | 18 | 92942 | oar3_OAR14_13134280 | oar3_OAR14_13227221 |
| 14 | 13157531 | 13227221 | 15 | 69691 | oar3_OAR14_13157531 | oar3_OAR14_13227221 |
| 14 | 13157531 | 13331069 | 35 | 173539 | oar3_OAR14_13157531 | oar3_OAR14_13331069 |
| 14 | 13157531 | 13227221 | 15 | 69691 | oar3_OAR14_13157531 | oar3_OAR14_13227221 |
| 14 | 13158400 | 13207684 | 8 | 49285 | s48619.1 | oar3_OAR14_13207684 |
| 14 | 13158400 | 13207684 | 8 | 49285 | s48619.1 | oar3_OAR14_13207684 |
| 14 | 13170773 | 13227221 | 11 | 56449 | oar3_OAR14_13170773 | oar3_OAR14_13227221 |
| 14 | 13170773 | 13209979 | 9 | 39207 | oar3_OAR14_13170773 | oar3_OAR14_13209979 |
| 14 | 13170773 | 13209979 | 9 | 39207 | oar3_OAR14_13170773 | oar3_OAR14_13209979 |
| 14 | 13176285 | 13209979 | 8 | 33695 | oar3_OAR14_13176285 | oar3_OAR14_13209979 |
| 14 | 13192235 | 13227221 | 9 | 34987 | oar3_OAR14_13192235 | oar3_OAR14_13227221 |
| 14 | 13193632 | 13227221 | 8 | 33590 | oar3_OAR14_13193632 | oar3_OAR14_13227221 |
| 14 | 13209804 | 13227221 | 4 | 17418 | oar3_OAR14_13209804 | oar3_OAR14_13227221 |
| 14 | 13252965 | 13345213 | 22 | 92249 | oar3_OAR14_13252965 | oar3_OAR14_13345213 |
| 14 | 13329605 | 13418934 | 23 | 89330 | oar3_OAR14_13329605 | oar3_OAR14_13418934 |
| 14 | 13329605 | 13340667 | 8 | 11063 | oar3_OAR14_13329605 | oar3_OAR14_13340667 |
| 14 | 13386712 | 13604818 | 40 | 218107 | oar3_OAR14_13386712 | s20732.1 |
| 14 | 13386712 | 13664080 | 56 | 277369 | oar3_OAR14_13386712 | oar3_OAR14_13664080 |
| 14 | 13386712 | 13488760 | 20 | 102049 | oar3_OAR14_13386712 | oar3_OAR14_13488760 |
| 14 | 13418934 | 13483521 | 9 | 64588 | oar3_OAR14_13418934 | oar3_OAR14_13483521 |
| 14 | 13443326 | 13483521 | 6 | 40196 | oar3_OAR14_13443326 | oar3_OAR14_13483521 |
| 14 | 13512286 | 13604818 | 20 | 92533 | oar3_OAR14_13512286 | s20732.1 |
| 14 | 13525677 | 13604818 | 19 | 79142 | oar3_OAR14_13525677 | s20732.1 |
| 14 | 13585274 | 13696861 | 27 | 111588 | oar3_OAR14_13585274 | s42649.1 |
| 14 | 13585274 | 13619101 | 7 | 33828 | oar3_OAR14_13585274 | oar3_OAR14_13619101 |
| 14 | 13721933 | 13871650 | 27 | 149718 | s11062.1 | oar3_OAR14_13871650 |
| 14 | 13767352 | 13835313 | 9 | 67962 | s65572.1 | oar3_OAR14_13835313 |
| 14 | 13782223 | 13835313 | 7 | 53091 | oar3_OAR14_13782223 | oar3_OAR14_13835313 |
| 14 | 13978204 | 14073103 | 28 | 94900 | oar3_OAR14_13978204 | oar3_OAR14_14073103 |
| 14 | 13998910 | 14040152 | 16 | 41243 | oar3_OAR14_13998910 | oar3_OAR14_14040152 |
| 14 | 13998910 | 14044425 | 17 | 45516 | oar3_OAR14_13998910 | oar3_OAR14_14044425 |
| 14 | 14002347 | 14051638 | 15 | 49292 | oar3_OAR14_14002347 | oar3_OAR14_14051638 |
| 14 | 14022405 | 14046707 | 10 | 24303 | oar3_OAR14_14022405 | oar3_OAR14_14046707 |
| 14 | 14103318 | 14207502 | 28 | 104185 | oar3_OAR14_14103318 | s26449.1 |
| 14 | 14222615 | 14251648 | 14 | 29034 | oar3_OAR14_14222615 | oar3_OAR14_14251648 |
| 14 | 14222615 | 14251648 | 14 | 29034 | oar3_OAR14_14222615 | oar3_OAR14_14251648 |
| 14 | 14222615 | 14251648 | 14 | 29034 | oar3_OAR14_14222615 | oar3_OAR14_14251648 |
| 14 | 14227586 | 14314607 | 32 | 87022 | oar3_OAR14_14227586 | oar3_OAR14_14314607 |
| 14 | 14249926 | 14314607 | 21 | 64682 | oar3_OAR14_14249926 | oar3_OAR14_14314607 |
| 14 | 14302506 | 14342559 | 9 | 40054 | oar3_OAR14_14302506 | oar3_OAR14_14342559 |
| 14 | 14683684 | 14693032 | 4 | 9349 | oar3_OAR14_14683684 | oar3_OAR14_14693032 |
| 14 | 14691342 | 14693032 | 3 | 1691 | OAR14_14894096.1 | oar3_OAR14_14693032 |
| 14 | 17649078 | 17661082 | 6 | 12005 | oar3_OAR14_17649078 | oar3_OAR14_17661082 |
| 14 | 18567612 | 18607834 | 14 | 40223 | oar3_OAR14_18567612 | oar3_OAR14_18607834 |
| 14 | 18576227 | 18607834 | 12 | 31608 | oar3_OAR14_18576227 | oar3_OAR14_18607834 |
| 14 | 18602707 | 18607834 | 7 | 5128 | oar3_OAR14_18602707 | oar3_OAR14_18607834 |
| 14 | 22803515 | 22828379 | 4 | 24865 | oar3_OAR14_22803515 | oar3_OAR14_22828379 |
| 14 | 23720128 | 23786623 | 14 | 66496 | oar3_OAR14_23720128 | oar3_OAR14_23786623 |
| 14 | 23747271 | 23786623 | 11 | 39353 | oar3_OAR14_23747271 | oar3_OAR14_23786623 |
| 14 | 23753094 | 23786623 | 10 | 33530 | oar3_OAR14_23753094 | oar3_OAR14_23786623 |
| 14 | 24909797 | 24955605 | 11 | 45809 | oar3_OAR14_24909797 | oar3_OAR14_24955605 |
| 14 | 24941895 | 24984529 | 9 | 42635 | oar3_OAR14_24941895 | oar3_OAR14_24984529 |
| 14 | 25126096 | 25171821 | 10 | 45726 | oar3_OAR14_25126096 | oar3_OAR14_25171821 |
| 14 | 25144976 | 25176839 | 9 | 31864 | oar3_OAR14_25144976 | oar3_OAR14_25176839 |
| 14 | 25371970 | 25407161 | 12 | 35192 | oar3_OAR14_25371970 | oar3_OAR14_25407161 |
| 14 | 33905765 | 34002979 | 29 | 97215 | oar3_OAR14_33905765 | oar3_OAR14_34002979 |
| 14 | 33930065 | 34002979 | 26 | 72915 | oar3_OAR14_33930065 | oar3_OAR14_34002979 |
| 14 | 33930065 | 33992585 | 23 | 62521 | oar3_OAR14_33930065 | oar3_OAR14_33992585 |
| 14 | 33957350 | 33965858 | 7 | 8509 | oar3_OAR14_33957350 | oar3_OAR14_33965858 |
| 14 | 33964696 | 33965858 | 5 | 1163 | oar3_OAR14_33964696 | oar3_OAR14_33965858 |
| 14 | 33964696 | 33965858 | 5 | 1163 | oar3_OAR14_33964696 | oar3_OAR14_33965858 |
| 14 | 34384667 | 34470259 | 17 | 85593 | oar3_OAR14_34384667 | oar3_OAR14_34470259 |
| 14 | 34533929 | 34555098 | 15 | 21170 | oar3_OAR14_34533929 | oar3_OAR14_34555098 |
| 14 | 34533929 | 34555098 | 15 | 21170 | oar3_OAR14_34533929 | oar3_OAR14_34555098 |
| 14 | 34533929 | 34555098 | 15 | 21170 | oar3_OAR14_34533929 | oar3_OAR14_34555098 |
| 14 | 34533929 | 34555098 | 15 | 21170 | oar3_OAR14_34533929 | oar3_OAR14_34555098 |
| 14 | 34533929 | 34545201 | 9 | 11273 | oar3_OAR14_34533929 | oar3_OAR14_34545201 |
| 14 | 34533929 | 34563694 | 16 | 29766 | oar3_OAR14_34533929 | oar3_OAR14_34563694 |
| 14 | 34533929 | 34545201 | 9 | 11273 | oar3_OAR14_34533929 | oar3_OAR14_34545201 |
| 14 | 34533929 | 34563694 | 16 | 29766 | oar3_OAR14_34533929 | oar3_OAR14_34563694 |
| 14 | 34533929 | 34555098 | 15 | 21170 | oar3_OAR14_34533929 | oar3_OAR14_34555098 |
| 14 | 34533929 | 34563694 | 16 | 29766 | oar3_OAR14_34533929 | oar3_OAR14_34563694 |
| 14 | 34533929 | 34542855 | 8 | 8927 | oar3_OAR14_34533929 | oar3_OAR14_34542855 |
| 14 | 34533929 | 34545201 | 9 | 11273 | oar3_OAR14_34533929 | oar3_OAR14_34545201 |
| 14 | 34533929 | 34540798 | 5 | 6870 | oar3_OAR14_34533929 | oar3_OAR14_34540798 |
| 14 | 34533929 | 34563694 | 16 | 29766 | oar3_OAR14_34533929 | oar3_OAR14_34563694 |
| 14 | 34533929 | 34545201 | 9 | 11273 | oar3_OAR14_34533929 | oar3_OAR14_34545201 |
| 14 | 34740380 | 34771787 | 11 | 31408 | oar3_OAR14_34740380 | oar3_OAR14_34771787 |
| 14 | 34951605 | 34981254 | 10 | 29650 | oar3_OAR14_34951605 | oar3_OAR14_34981254 |
| 14 | 34951605 | 34981254 | 10 | 29650 | oar3_OAR14_34951605 | oar3_OAR14_34981254 |
| 14 | 34954598 | 34981254 | 9 | 26657 | oar3_OAR14_34954598 | oar3_OAR14_34981254 |
| 14 | 37466093 | 37491431 | 8 | 25339 | oar3_OAR14_37466093 | oar3_OAR14_37491431 |
| 14 | 37487721 | 37502042 | 4 | 14322 | oar3_OAR14_37487721 | oar3_OAR14_37502042 |
| 14 | 39913640 | 39923034 | 7 | 9395 | oar3_OAR14_39913640 | oar3_OAR14_39923034 |
| 14 | 40184299 | 40196472 | 8 | 12174 | oar3_OAR14_40184299 | oar3_OAR14_40196472 |
| 14 | 42923610 | 42952372 | 15 | 28763 | oar3_OAR14_42923610 | oar3_OAR14_42952372 |
| 14 | 42923610 | 42952372 | 15 | 28763 | oar3_OAR14_42923610 | oar3_OAR14_42952372 |
| 14 | 42923610 | 42967982 | 18 | 44373 | oar3_OAR14_42923610 | oar3_OAR14_42967982 |
| 14 | 42923610 | 42976818 | 19 | 53209 | oar3_OAR14_42923610 | oar3_OAR14_42976818 |
| 14 | 42923610 | 42976818 | 19 | 53209 | oar3_OAR14_42923610 | oar3_OAR14_42976818 |
| 14 | 42935672 | 43027485 | 25 | 91814 | s44639.1 | s68508.1 |
| 14 | 42935672 | 42952372 | 10 | 16701 | s44639.1 | oar3_OAR14_42952372 |
| 14 | 42941926 | 42952372 | 9 | 10447 | oar3_OAR14_42941926 | oar3_OAR14_42952372 |
| 14 | 42941926 | 42952372 | 9 | 10447 | oar3_OAR14_42941926 | oar3_OAR14_42952372 |
| 14 | 42941926 | 42952372 | 9 | 10447 | oar3_OAR14_42941926 | oar3_OAR14_42952372 |
| 14 | 42952055 | 42967982 | 6 | 15928 | oar3_OAR14_42952055 | oar3_OAR14_42967982 |
| 14 | 43099757 | 43187555 | 19 | 87799 | oar3_OAR14_43099757 | s25120.1 |
| 14 | 43099757 | 43113080 | 6 | 13324 | oar3_OAR14_43099757 | oar3_OAR14_43113080 |
| 14 | 43105221 | 43133156 | 9 | 27936 | oar3_OAR14_43105221 | oar3_OAR14_43133156 |
| 14 | 43105221 | 43113080 | 5 | 7860 | oar3_OAR14_43105221 | oar3_OAR14_43113080 |
| 14 | 43105221 | 43177984 | 15 | 72764 | oar3_OAR14_43105221 | oar3_OAR14_43177984 |
| 14 | 43105221 | 43118370 | 6 | 13150 | oar3_OAR14_43105221 | oar3_OAR14_43118370 |
| 14 | 43105221 | 43118370 | 6 | 13150 | oar3_OAR14_43105221 | oar3_OAR14_43118370 |
| 14 | 43292608 | 43339099 | 13 | 46492 | oar3_OAR14_43292608 | oar3_OAR14_43339099 |
| 14 | 45073313 | 45118555 | 10 | 45243 | OAR14_47394164.1 | oar3_OAR14_45118555 |
| 14 | 45459317 | 45466605 | 8 | 7289 | oar3_OAR14_45459317 | oar3_OAR14_45466605 |
| 14 | 45459317 | 45463949 | 7 | 4633 | oar3_OAR14_45459317 | oar3_OAR14_45463949 |
| 14 | 45459317 | 45466605 | 8 | 7289 | oar3_OAR14_45459317 | oar3_OAR14_45466605 |
| 14 | 45459317 | 45463949 | 7 | 4633 | oar3_OAR14_45459317 | oar3_OAR14_45463949 |
| 14 | 45459317 | 45466605 | 8 | 7289 | oar3_OAR14_45459317 | oar3_OAR14_45466605 |
| 14 | 45459317 | 45470366 | 10 | 11050 | oar3_OAR14_45459317 | oar3_OAR14_45470366 |
| 14 | 45555737 | 45631722 | 25 | 75986 | oar3_OAR14_45555737 | oar3_OAR14_45631722 |
| 14 | 45558243 | 45626329 | 22 | 68087 | oar3_OAR14_45558243 | s07549.1 |
| 14 | 45587237 | 45626329 | 15 | 39093 | oar3_OAR14_45587237 | s07549.1 |
| 14 | 45603150 | 45603907 | 5 | 758 | oar3_OAR14_45603150 | oar3_OAR14_45603907 |
| 14 | 45884122 | 45901130 | 7 | 17009 | oar3_OAR14_45884122 | s50401.1 |
| 14 | 45886100 | 45893975 | 5 | 7876 | oar3_OAR14_45886100 | oar3_OAR14_45893975 |
| 14 | 47375489 | 47397369 | 6 | 21881 | oar3_OAR14_47375489 | oar3_OAR14_47397369 |
| 14 | 47381817 | 47417085 | 8 | 35269 | oar3_OAR14_47381817 | oar3_OAR14_47417085 |
| 14 | 47568761 | 47620133 | 14 | 51373 | oar3_OAR14_47568761 | oar3_OAR14_47620133 |
| 14 | 47649878 | 47667451 | 5 | 17574 | oar3_OAR14_47649878 | oar3_OAR14_47667451 |
| 14 | 47649878 | 47688753 | 12 | 38876 | oar3_OAR14_47649878 | oar3_OAR14_47688753 |
| 14 | 47656640 | 47688753 | 11 | 32114 | oar3_OAR14_47656640 | oar3_OAR14_47688753 |
| 14 | 48271380 | 48282238 | 7 | 10859 | oar3_OAR14_48271380 | oar3_OAR14_48282238 |
| 14 | 48271380 | 48283367 | 8 | 11988 | oar3_OAR14_48271380 | oar3_OAR14_48283367 |
| 14 | 48556836 | 48580195 | 7 | 23360 | oar3_OAR14_48556836 | oar3_OAR14_48580195 |
| 14 | 48738235 | 48825455 | 17 | 87221 | oar3_OAR14_48738235 | oar3_OAR14_48825455 |
| 14 | 48738235 | 48769169 | 9 | 30935 | oar3_OAR14_48738235 | oar3_OAR14_48769169 |
| 14 | 48976459 | 49165067 | 44 | 188609 | oar3_OAR14_48976459 | oar3_OAR14_49165067 |
| 14 | 49127911 | 49165067 | 14 | 37157 | oar3_OAR14_49127911 | oar3_OAR14_49165067 |
| 14 | 49127911 | 49177147 | 16 | 49237 | oar3_OAR14_49127911 | oar3_OAR14_49177147 |
| 14 | 49127911 | 49165067 | 14 | 37157 | oar3_OAR14_49127911 | oar3_OAR14_49165067 |
| 14 | 49135580 | 49165067 | 12 | 29488 | OAR14_51753220.1 | oar3_OAR14_49165067 |
| 14 | 49140834 | 49165067 | 11 | 24234 | oar3_OAR14_49140834 | oar3_OAR14_49165067 |
| 14 | 49140834 | 49165067 | 11 | 24234 | oar3_OAR14_49140834 | oar3_OAR14_49165067 |
| 14 | 49140834 | 49165067 | 11 | 24234 | oar3_OAR14_49140834 | oar3_OAR14_49165067 |
| 14 | 49140834 | 49142455 | 5 | 1622 | oar3_OAR14_49140834 | oar3_OAR14_49142455 |
| 14 | 49947004 | 50039584 | 14 | 92581 | oar3_OAR14_49947004 | oar3_OAR14_50039584 |
| 14 | 50285766 | 50428350 | 29 | 142585 | oar3_OAR14_50285766 | oar3_OAR14_50428350 |
| 14 | 50285766 | 50286963 | 3 | 1198 | oar3_OAR14_50285766 | oar3_OAR14_50286963 |
| 14 | 50285766 | 50406220 | 25 | 120455 | oar3_OAR14_50285766 | oar3_OAR14_50406220 |
| 14 | 50285766 | 50286963 | 3 | 1198 | oar3_OAR14_50285766 | oar3_OAR14_50286963 |
| 14 | 50285766 | 50295887 | 4 | 10122 | oar3_OAR14_50285766 | s40738.1 |
| 14 | 50285766 | 50295887 | 4 | 10122 | oar3_OAR14_50285766 | s40738.1 |
| 14 | 50349850 | 50416651 | 15 | 66802 | oar3_OAR14_50349850 | oar3_OAR14_50416651 |
| 14 | 50378486 | 50406220 | 8 | 27735 | oar3_OAR14_50378486 | oar3_OAR14_50406220 |
| 14 | 50892396 | 50903463 | 9 | 11068 | oar3_OAR14_50892396 | oar3_OAR14_50903463 |
| 14 | 50892396 | 50903463 | 9 | 11068 | oar3_OAR14_50892396 | oar3_OAR14_50903463 |
| 14 | 51699180 | 51718006 | 7 | 18827 | oar3_OAR14_51699180 | oar3_OAR14_51718006 |
| 14 | 51818528 | 51880604 | 18 | 62077 | oar3_OAR14_51818528 | oar3_OAR14_51880604 |
| 14 | 51818528 | 51853244 | 8 | 34717 | oar3_OAR14_51818528 | oar3_OAR14_51853244 |
| 14 | 51818528 | 51880604 | 18 | 62077 | oar3_OAR14_51818528 | oar3_OAR14_51880604 |
| 14 | 51853244 | 51880604 | 11 | 27361 | oar3_OAR14_51853244 | oar3_OAR14_51880604 |
| 14 | 51853244 | 51880604 | 11 | 27361 | oar3_OAR14_51853244 | oar3_OAR14_51880604 |
| 14 | 51869118 | 51880604 | 4 | 11487 | oar3_OAR14_51869118 | oar3_OAR14_51880604 |
| 14 | 51869118 | 51880604 | 4 | 11487 | oar3_OAR14_51869118 | oar3_OAR14_51880604 |
| 14 | 51869118 | 51880604 | 4 | 11487 | oar3_OAR14_51869118 | oar3_OAR14_51880604 |
| 14 | 52433333 | 52440673 | 6 | 7341 | oar3_OAR14_52433333 | oar3_OAR14_52440673 |
| 14 | 52433333 | 52469860 | 12 | 36528 | oar3_OAR14_52433333 | oar3_OAR14_52469860 |
| 14 | 52433333 | 52490037 | 16 | 56705 | oar3_OAR14_52433333 | oar3_OAR14_52490037 |
| 14 | 52451245 | 52469860 | 6 | 18616 | oar3_OAR14_52451245 | oar3_OAR14_52469860 |
| 14 | 52892807 | 52924247 | 8 | 31441 | oar3_OAR14_52892807 | oar3_OAR14_52924247 |
| 14 | 52892807 | 52924247 | 8 | 31441 | oar3_OAR14_52892807 | oar3_OAR14_52924247 |
| 14 | 53134002 | 53191571 | 15 | 57570 | oar3_OAR14_53134002 | oar3_OAR14_53191571 |
| 14 | 53175002 | 53191571 | 6 | 16570 | oar3_OAR14_53175002 | oar3_OAR14_53191571 |
| 14 | 53175002 | 53200892 | 9 | 25891 | oar3_OAR14_53175002 | oar3_OAR14_53200892 |
| 14 | 53180943 | 53200892 | 7 | 19950 | oar3_OAR14_53180943 | oar3_OAR14_53200892 |
| 14 | 54899954 | 54913688 | 8 | 13735 | oar3_OAR14_54899954 | OAR14_58084909.1 |
| 14 | 55215389 | 55292749 | 15 | 77361 | oar3_OAR14_55215389 | oar3_OAR14_55292749 |
| 14 | 55215389 | 55275828 | 13 | 60440 | oar3_OAR14_55215389 | oar3_OAR14_55275828 |
| 14 | 55673986 | 55771151 | 21 | 97166 | oar3_OAR14_55673986 | oar3_OAR14_55771151 |
| 14 | 55764084 | 55768776 | 4 | 4693 | oar3_OAR14_55764084 | oar3_OAR14_55768776 |
| 14 | 55764084 | 55768776 | 4 | 4693 | oar3_OAR14_55764084 | oar3_OAR14_55768776 |
| 14 | 56310348 | 56344462 | 10 | 34115 | oar3_OAR14_56310348 | oar3_OAR14_56344462 |
| 14 | 58396369 | 58449682 | 5 | 53314 | oar3_OAR14_58396369 | oar3_OAR14_58449682 |
| 14 | 58704442 | 58742021 | 5 | 37580 | oar3_OAR14_58704442 | oar3_OAR14_58742021 |
| 14 | 59200677 | 59234430 | 6 | 33754 | oar3_OAR14_59200677 | oar3_OAR14_59234430 |
| 14 | 59268403 | 59282586 | 5 | 14184 | oar3_OAR14_59268403 | s13476.1 |
| 14 | 59309428 | 59375504 | 10 | 66077 | oar3_OAR14_59309428 | oar3_OAR14_59375504 |
| 14 | 59372644 | 59406372 | 8 | 33729 | s06052.1 | oar3_OAR14_59406372 |
| 14 | 59434285 | 59440976 | 6 | 6692 | oar3_OAR14_59434285 | oar3_OAR14_59440976 |
| 14 | 59440037 | 59456425 | 7 | 16389 | oar3_OAR14_59440037 | oar3_OAR14_59456425 |
| 14 | 59474370 | 59503334 | 10 | 28965 | oar3_OAR14_59474370 | oar3_OAR14_59503334 |
| 14 | 59474370 | 59505614 | 12 | 31245 | oar3_OAR14_59474370 | oar3_OAR14_59505614 |
| 14 | 59474370 | 59553471 | 33 | 79102 | oar3_OAR14_59474370 | oar3_OAR14_59553471 |
| 14 | 59492088 | 59512695 | 9 | 20608 | oar3_OAR14_59492088 | oar3_OAR14_59512695 |
| 14 | 59868493 | 60004598 | 9 | 136106 | oar3_OAR14_59868493 | oar3_OAR14_60004598 |
| 14 | 59911156 | 60004598 | 6 | 93443 | oar3_OAR14_59911156 | oar3_OAR14_60004598 |
| 14 | 59955030 | 60004598 | 3 | 49569 | oar3_OAR14_59955030 | oar3_OAR14_60004598 |
| 14 | 59955030 | 60004598 | 3 | 49569 | oar3_OAR14_59955030 | oar3_OAR14_60004598 |
| 14 | 59955030 | 60004598 | 3 | 49569 | oar3_OAR14_59955030 | oar3_OAR14_60004598 |
| 14 | 60051230 | 60067483 | 5 | 16254 | oar3_OAR14_60051230 | oar3_OAR14_60067483 |
| 14 | 60051230 | 60160844 | 23 | 109615 | oar3_OAR14_60051230 | oar3_OAR14_60160844 |
| 14 | 60148265 | 60160844 | 6 | 12580 | oar3_OAR14_60148265 | oar3_OAR14_60160844 |
| 14 | 60202519 | 60225980 | 7 | 23462 | oar3_OAR14_60202519 | oar3_OAR14_60225980 |
| 14 | 60202519 | 60225980 | 7 | 23462 | oar3_OAR14_60202519 | oar3_OAR14_60225980 |
| 14 | 60202519 | 60225980 | 7 | 23462 | oar3_OAR14_60202519 | oar3_OAR14_60225980 |
| 14 | 60305045 | 60333950 | 6 | 28906 | oar3_OAR14_60305045 | oar3_OAR14_60333950 |
| 14 | 60305045 | 60333950 | 6 | 28906 | oar3_OAR14_60305045 | oar3_OAR14_60333950 |
| 14 | 60314966 | 60324303 | 3 | 9338 | oar3_OAR14_60314966 | s28142.1 |
| 14 | 60659994 | 60660230 | 3 | 237 | oar3_OAR14_60659994 | oar3_OAR14_60660230 |
| 14 | 60745353 | 60776912 | 5 | 31560 | oar3_OAR14_60745353 | oar3_OAR14_60776912 |
| 14 | 60844276 | 60865666 | 7 | 21391 | oar3_OAR14_60844276 | oar3_OAR14_60865666 |
| 14 | 62329549 | 62357890 | 10 | 28342 | oar3_OAR14_62329549 | oar3_OAR14_62357890 |
| 14 | 62329549 | 62357890 | 10 | 28342 | oar3_OAR14_62329549 | oar3_OAR14_62357890 |
| 14 | 62475233 | 62552503 | 19 | 77271 | oar3_OAR14_62475233 | s52366.1 |
| 14 | 62475233 | 62478060 | 5 | 2828 | oar3_OAR14_62475233 | oar3_OAR14_62478060 |
| 14 | 62476204 | 62524546 | 12 | 48343 | s13638.1 | oar3_OAR14_62524546 |
| 14 | 62684831 | 62712806 | 8 | 27976 | s53928.1 | oar3_OAR14_62712806 |
| 15 | 629000 | 676172 | 5 | 47173 | oar3_OAR15_629000 | oar3_OAR15_676172 |
| 15 | 669213 | 676172 | 3 | 6960 | oar3_OAR15_669213 | oar3_OAR15_676172 |
| 15 | 801367 | 804306 | 5 | 2940 | oar3_OAR15_801367 | s08401.1 |
| 15 | 801367 | 802651 | 4 | 1285 | oar3_OAR15_801367 | oar3_OAR15_802651 |
| 15 | 3712387 | 3721081 | 3 | 8695 | oar3_OAR15_3712387 | oar3_OAR15_3721081 |
| 15 | 7984803 | 7990097 | 5 | 5295 | oar3_OAR15_7984803 | oar3_OAR15_7990097 |
| 15 | 7984803 | 7990097 | 5 | 5295 | oar3_OAR15_7984803 | oar3_OAR15_7990097 |
| 15 | 7984803 | 7990097 | 5 | 5295 | oar3_OAR15_7984803 | oar3_OAR15_7990097 |
| 15 | 9064342 | 9074874 | 4 | 10533 | oar3_OAR15_9064342 | OAR15_8828624.1 |
| 15 | 9064342 | 9065029 | 3 | 688 | oar3_OAR15_9064342 | oar3_OAR15_9065029 |
| 15 | 10579996 | 10756644 | 23 | 176649 | oar3_OAR15_10579996 | oar3_OAR15_10756644 |
| 15 | 10579996 | 10756644 | 23 | 176649 | oar3_OAR15_10579996 | oar3_OAR15_10756644 |
| 15 | 10860490 | 10868236 | 10 | 7747 | oar3_OAR15_10860490 | oar3_OAR15_10868236 |
| 15 | 11821781 | 11840982 | 6 | 19202 | oar3_OAR15_11821781 | OAR15_11778538.1 |
| 15 | 21175926 | 21184910 | 10 | 8985 | oar3_OAR15_21175926 | oar3_OAR15_21184910 |
| 15 | 21175926 | 21184910 | 10 | 8985 | oar3_OAR15_21175926 | oar3_OAR15_21184910 |
| 15 | 23397812 | 23411843 | 12 | 14032 | oar3_OAR15_23397812 | oar3_OAR15_23411843 |
| 15 | 23406043 | 23428322 | 9 | 22280 | oar3_OAR15_23406043 | oar3_OAR15_23428322 |
| 15 | 25710287 | 25727493 | 6 | 17207 | oar3_OAR15_25710287 | oar3_OAR15_25727493 |
| 15 | 27361124 | 27373372 | 9 | 12249 | oar3_OAR15_27361124 | oar3_OAR15_27373372 |
| 15 | 27361124 | 27404082 | 18 | 42959 | oar3_OAR15_27361124 | oar3_OAR15_27404082 |
| 15 | 27361124 | 27420320 | 21 | 59197 | oar3_OAR15_27361124 | oar3_OAR15_27420320 |
| 15 | 27361124 | 27386148 | 14 | 25025 | oar3_OAR15_27361124 | oar3_OAR15_27386148 |
| 15 | 27942259 | 27983674 | 13 | 41416 | oar3_OAR15_27942259 | oar3_OAR15_27983674 |
| 15 | 28059721 | 28087795 | 12 | 28075 | oar3_OAR15_28059721 | oar3_OAR15_28087795 |
| 15 | 28753772 | 28828662 | 18 | 74891 | oar3_OAR15_28753772 | oar3_OAR15_28828662 |
| 15 | 28773708 | 28803750 | 8 | 30043 | oar3_OAR15_28773708 | oar3_OAR15_28803750 |
| 15 | 28819192 | 28828662 | 6 | 9471 | oar3_OAR15_28819192 | oar3_OAR15_28828662 |
| 15 | 28968350 | 29016174 | 8 | 47825 | s26647.1 | oar3_OAR15_29016174 |
| 15 | 29358952 | 29448947 | 18 | 89996 | oar3_OAR15_29358952 | oar3_OAR15_29448947 |
| 15 | 29358952 | 29408838 | 15 | 49887 | oar3_OAR15_29358952 | oar3_OAR15_29408838 |
| 15 | 29358952 | 29454089 | 20 | 95138 | oar3_OAR15_29358952 | oar3_OAR15_29454089 |
| 15 | 34439098 | 34525777 | 33 | 86680 | oar3_OAR15_34439098 | oar3_OAR15_34525777 |
| 15 | 36160046 | 36174137 | 8 | 14092 | oar3_OAR15_36160046 | OAR15_38015931.1 |
| 15 | 41448307 | 41470852 | 8 | 22546 | oar3_OAR15_41448307 | oar3_OAR15_41470852 |
| 15 | 43857115 | 43898728 | 13 | 41614 | oar3_OAR15_43857115 | oar3_OAR15_43898728 |
| 15 | 43871045 | 43898728 | 10 | 27684 | oar3_OAR15_43871045 | oar3_OAR15_43898728 |
| 15 | 43871045 | 43898728 | 10 | 27684 | oar3_OAR15_43871045 | oar3_OAR15_43898728 |
| 15 | 43991607 | 44007877 | 11 | 16271 | oar3_OAR15_43991607 | oar3_OAR15_44007877 |
| 15 | 43991607 | 44000839 | 8 | 9233 | oar3_OAR15_43991607 | oar3_OAR15_44000839 |
| 15 | 43991607 | 44005146 | 9 | 13540 | oar3_OAR15_43991607 | oar3_OAR15_44005146 |
| 15 | 43991607 | 43998269 | 7 | 6663 | oar3_OAR15_43991607 | oar3_OAR15_43998269 |
| 15 | 45617290 | 45782764 | 25 | 165475 | oar3_OAR15_45617290 | oar3_OAR15_45782764 |
| 15 | 45621463 | 45722606 | 15 | 101144 | oar3_OAR15_45621463 | oar3_OAR15_45722606 |
| 15 | 45712002 | 45749098 | 9 | 37097 | oar3_OAR15_45712002 | oar3_OAR15_45749098 |
| 15 | 45828195 | 45873187 | 17 | 44993 | s63734.1 | oar3_OAR15_45873187 |
| 15 | 45828195 | 45848079 | 9 | 19885 | s63734.1 | oar3_OAR15_45848079 |
| 15 | 45828195 | 45873187 | 17 | 44993 | s63734.1 | oar3_OAR15_45873187 |
| 15 | 45905986 | 45929086 | 9 | 23101 | oar3_OAR15_45905986 | oar3_OAR15_45929086 |
| 15 | 45913946 | 45929086 | 8 | 15141 | oar3_OAR15_45913946 | oar3_OAR15_45929086 |
| 15 | 46589324 | 46589602 | 3 | 279 | oar3_OAR15_46589324 | oar3_OAR15_46589602 |
| 15 | 47507028 | 47555955 | 8 | 48928 | oar3_OAR15_47507028 | oar3_OAR15_47555955 |
| 15 | 47507028 | 47555955 | 8 | 48928 | oar3_OAR15_47507028 | oar3_OAR15_47555955 |
| 15 | 47507028 | 47555955 | 8 | 48928 | oar3_OAR15_47507028 | oar3_OAR15_47555955 |
| 15 | 49209182 | 49228717 | 5 | 19536 | oar3_OAR15_49209182 | oar3_OAR15_49228717 |
| 15 | 49649502 | 49667799 | 7 | 18298 | oar3_OAR15_49649502 | oar3_OAR15_49667799 |
| 15 | 49649502 | 49667799 | 7 | 18298 | oar3_OAR15_49649502 | oar3_OAR15_49667799 |
| 15 | 49649502 | 49667799 | 7 | 18298 | oar3_OAR15_49649502 | oar3_OAR15_49667799 |
| 15 | 49649502 | 49659070 | 4 | 9569 | oar3_OAR15_49649502 | oar3_OAR15_49659070 |
| 15 | 49649502 | 49667799 | 7 | 18298 | oar3_OAR15_49649502 | oar3_OAR15_49667799 |
| 15 | 49835046 | 49855154 | 7 | 20109 | oar3_OAR15_49835046 | oar3_OAR15_49855154 |
| 15 | 49841855 | 49855154 | 6 | 13300 | oar3_OAR15_49841855 | oar3_OAR15_49855154 |
| 15 | 50342943 | 50412751 | 18 | 69809 | oar3_OAR15_50342943 | oar3_OAR15_50412751 |
| 15 | 50347497 | 50393068 | 13 | 45572 | oar3_OAR15_50347497 | oar3_OAR15_50393068 |
| 15 | 50482885 | 50576349 | 24 | 93465 | oar3_OAR15_50482885 | oar3_OAR15_50576349 |
| 15 | 50906930 | 51097039 | 47 | 190110 | oar3_OAR15_50906930 | oar3_OAR15_51097039 |
| 15 | 50994816 | 51083206 | 20 | 88391 | oar3_OAR15_50994816 | oar3_OAR15_51083206 |
| 15 | 51046552 | 51097039 | 10 | 50488 | oar3_OAR15_51046552 | oar3_OAR15_51097039 |
| 15 | 52172246 | 52236445 | 15 | 64200 | oar3_OAR15_52172246 | oar3_OAR15_52236445 |
| 15 | 52628795 | 52711522 | 20 | 82728 | oar3_OAR15_52628795 | oar3_OAR15_52711522 |
| 15 | 53028026 | 53111509 | 18 | 83484 | oar3_OAR15_53028026 | oar3_OAR15_53111509 |
| 15 | 54240770 | 54349173 | 34 | 108404 | oar3_OAR15_54240770 | oar3_OAR15_54349173 |
| 15 | 54576084 | 54662734 | 29 | 86651 | oar3_OAR15_54576084 | oar3_OAR15_54662734 |
| 15 | 54576084 | 54665000 | 30 | 88917 | oar3_OAR15_54576084 | oar3_OAR15_54665000 |
| 15 | 54576084 | 54627085 | 19 | 51002 | oar3_OAR15_54576084 | oar3_OAR15_54627085 |
| 15 | 54582390 | 54627085 | 17 | 44696 | oar3_OAR15_54582390 | oar3_OAR15_54627085 |
| 15 | 54582390 | 54651008 | 24 | 68619 | oar3_OAR15_54582390 | oar3_OAR15_54651008 |
| 15 | 54605229 | 54618243 | 6 | 13015 | oar3_OAR15_54605229 | oar3_OAR15_54618243 |
| 15 | 54611066 | 54625264 | 9 | 14199 | oar3_OAR15_54611066 | oar3_OAR15_54625264 |
| 15 | 54616329 | 54662734 | 19 | 46406 | oar3_OAR15_54616329 | oar3_OAR15_54662734 |
| 15 | 54721799 | 54742999 | 6 | 21201 | oar3_OAR15_54721799 | oar3_OAR15_54742999 |
| 15 | 61943589 | 61963283 | 5 | 19695 | oar3_OAR15_61943589 | oar3_OAR15_61963283 |
| 15 | 64928671 | 64950987 | 9 | 22317 | oar3_OAR15_64928671 | oar3_OAR15_64950987 |
| 15 | 70857292 | 70944492 | 22 | 87201 | oar3_OAR15_70857292 | OAR15_76864698.1 |
| 15 | 72279854 | 72337960 | 16 | 58107 | oar3_OAR15_72279854 | oar3_OAR15_72337960 |
| 15 | 72536427 | 72571995 | 11 | 35569 | s31340.1 | oar3_OAR15_72571995 |
| 15 | 72536427 | 72571995 | 11 | 35569 | s31340.1 | oar3_OAR15_72571995 |
| 15 | 73090453 | 73238817 | 34 | 148365 | oar3_OAR15_73090453 | oar3_OAR15_73238817 |
| 15 | 74068333 | 74157614 | 20 | 89282 | oar3_OAR15_74068333 | oar3_OAR15_74157614 |
| 15 | 74124684 | 74191693 | 12 | 67010 | oar3_OAR15_74124684 | oar3_OAR15_74191693 |
| 15 | 74150649 | 74157614 | 3 | 6966 | oar3_OAR15_74150649 | oar3_OAR15_74157614 |
| 15 | 74541439 | 74583539 | 11 | 42101 | oar3_OAR15_74541439 | oar3_OAR15_74583539 |
| 15 | 74541439 | 74618284 | 16 | 76846 | oar3_OAR15_74541439 | oar3_OAR15_74618284 |
| 15 | 74568287 | 74594662 | 6 | 26376 | oar3_OAR15_74568287 | oar3_OAR15_74594662 |
| 15 | 74568287 | 74605377 | 8 | 37091 | oar3_OAR15_74568287 | oar3_OAR15_74605377 |
| 15 | 74581798 | 74618284 | 7 | 36487 | oar3_OAR15_74581798 | oar3_OAR15_74618284 |
| 15 | 74618189 | 74645248 | 8 | 27060 | s19862.1 | oar3_OAR15_74645248 |
| 15 | 74862644 | 74889465 | 8 | 26822 | oar3_OAR15_74862644 | oar3_OAR15_74889465 |
| 15 | 74862644 | 74867194 | 3 | 4551 | oar3_OAR15_74862644 | oar3_OAR15_74867194 |
| 15 | 74862644 | 74889465 | 8 | 26822 | oar3_OAR15_74862644 | oar3_OAR15_74889465 |
| 15 | 74862644 | 74889465 | 8 | 26822 | oar3_OAR15_74862644 | oar3_OAR15_74889465 |
| 15 | 74862644 | 74897563 | 10 | 34920 | oar3_OAR15_74862644 | oar3_OAR15_74897563 |
| 15 | 74862644 | 74889465 | 8 | 26822 | oar3_OAR15_74862644 | oar3_OAR15_74889465 |
| 15 | 74862644 | 74889465 | 8 | 26822 | oar3_OAR15_74862644 | oar3_OAR15_74889465 |
| 15 | 74866907 | 74897563 | 9 | 30657 | oar3_OAR15_74866907 | oar3_OAR15_74897563 |
| 15 | 74886567 | 74897563 | 5 | 10997 | oar3_OAR15_74886567 | oar3_OAR15_74897563 |
| 15 | 75278442 | 75365036 | 19 | 86595 | oar3_OAR15_75278442 | oar3_OAR15_75365036 |
| 15 | 75386095 | 75494800 | 10 | 108706 | oar3_OAR15_75386095 | oar3_OAR15_75494800 |
| 15 | 75592162 | 75625948 | 9 | 33787 | s27629.1 | oar3_OAR15_75625948 |
| 15 | 75624723 | 75625948 | 3 | 1226 | oar3_OAR15_75624723 | oar3_OAR15_75625948 |
| 15 | 77298425 | 77369151 | 10 | 70727 | oar3_OAR15_77298425 | oar3_OAR15_77369151 |
| 15 | 77675447 | 77809432 | 29 | 133986 | oar3_OAR15_77675447 | oar3_OAR15_77809432 |
| 15 | 77682919 | 77694139 | 7 | 11221 | oar3_OAR15_77682919 | oar3_OAR15_77694139 |
| 15 | 77759366 | 77899849 | 22 | 140484 | oar3_OAR15_77759366 | oar3_OAR15_77899849 |
| 15 | 77878390 | 78013778 | 32 | 135389 | oar3_OAR15_77878390 | oar3_OAR15_78013778 |
| 15 | 77925526 | 78058147 | 35 | 132622 | oar3_OAR15_77925526 | oar3_OAR15_78058147 |
| 15 | 77925526 | 78028783 | 23 | 103258 | oar3_OAR15_77925526 | oar3_OAR15_78028783 |
| 15 | 77926148 | 77980293 | 11 | 54146 | oar3_OAR15_77926148 | oar3_OAR15_77980293 |
| 15 | 77950996 | 78013778 | 15 | 62783 | oar3_OAR15_77950996 | oar3_OAR15_78013778 |
| 15 | 77993435 | 78037504 | 13 | 44070 | oar3_OAR15_77993435 | oar3_OAR15_78037504 |
| 15 | 78003763 | 78028783 | 8 | 25021 | oar3_OAR15_78003763 | oar3_OAR15_78028783 |
| 15 | 78111104 | 78158853 | 7 | 47750 | oar3_OAR15_78111104 | oar3_OAR15_78158853 |
| 15 | 78115359 | 78158853 | 5 | 43495 | oar3_OAR15_78115359 | oar3_OAR15_78158853 |
| 15 | 78452441 | 78462775 | 7 | 10335 | oar3_OAR15_78452441 | oar3_OAR15_78462775 |
| 15 | 79101147 | 79116909 | 10 | 15763 | oar3_OAR15_79101147 | oar3_OAR15_79116909 |
| 15 | 79739740 | 79818988 | 20 | 79249 | oar3_OAR15_79739740 | oar3_OAR15_79818988 |
| 15 | 80417733 | 80687006 | 49 | 269274 | s34471.1 | oar3_OAR15_80687006 |
| 15 | 80417733 | 80586243 | 41 | 168511 | s34471.1 | oar3_OAR15_80586243 |
| 15 | 80431191 | 80505717 | 17 | 74527 | oar3_OAR15_80431191 | oar3_OAR15_80505717 |
| 15 | 80470748 | 80548448 | 15 | 77701 | oar3_OAR15_80470748 | oar3_OAR15_80548448 |
| 15 | 80470748 | 80548448 | 15 | 77701 | oar3_OAR15_80470748 | oar3_OAR15_80548448 |
| 15 | 80489531 | 80505717 | 6 | 16187 | oar3_OAR15_80489531 | oar3_OAR15_80505717 |
| 15 | 80489531 | 80505717 | 6 | 16187 | oar3_OAR15_80489531 | oar3_OAR15_80505717 |
| 15 | 80489531 | 80505717 | 6 | 16187 | oar3_OAR15_80489531 | oar3_OAR15_80505717 |
| 15 | 80504263 | 80687006 | 26 | 182744 | oar3_OAR15_80504263 | oar3_OAR15_80687006 |
| 15 | 80567819 | 80751256 | 18 | 183438 | oar3_OAR15_80567819 | oar3_OAR15_80751256 |
| 15 | 80567819 | 80751256 | 18 | 183438 | oar3_OAR15_80567819 | oar3_OAR15_80751256 |
| 15 | 80642170 | 80687006 | 3 | 44837 | s05017.1 | oar3_OAR15_80687006 |
| 15 | 80745591 | 80812899 | 8 | 67309 | oar3_OAR15_80745591 | oar3_OAR15_80812899 |
| 16 | 2027997 | 2066223 | 17 | 38227 | oar3_OAR16_2027997 | oar3_OAR16_2066223 |
| 16 | 2056847 | 2066223 | 8 | 9377 | oar3_OAR16_2056847 | oar3_OAR16_2066223 |
| 16 | 2059505 | 2099501 | 11 | 39997 | oar3_OAR16_2059505 | oar3_OAR16_2099501 |
| 16 | 5509030 | 5514641 | 5 | 5612 | oar3_OAR16_5509030 | oar3_OAR16_5514641 |
| 16 | 10040544 | 10072505 | 9 | 31962 | oar3_OAR16_10040544 | oar3_OAR16_10072505 |
| 16 | 10050464 | 10051751 | 3 | 1288 | oar3_OAR16_10050464 | oar3_OAR16_10051751 |
| 16 | 11002195 | 11027631 | 6 | 25437 | oar3_OAR16_11002195 | oar3_OAR16_11027631 |
| 16 | 31692779 | 31800984 | 22 | 108206 | oar3_OAR16_31692779 | oar3_OAR16_31800984 |
| 16 | 36140939 | 36141003 | 3 | 65 | oar3_OAR16_36140939 | oar3_OAR16_36141003 |
| 16 | 40239884 | 40258646 | 7 | 18763 | oar3_OAR16_40239884 | OAR16_43758108.1 |
| 16 | 43106724 | 43111237 | 3 | 4514 | oar3_OAR16_43106724 | oar3_OAR16_43111237 |
| 16 | 43106724 | 43111237 | 3 | 4514 | oar3_OAR16_43106724 | oar3_OAR16_43111237 |
| 16 | 44555876 | 44630943 | 10 | 75068 | oar3_OAR16_44555876 | oar3_OAR16_44630943 |
| 16 | 47598980 | 47699808 | 21 | 100829 | oar3_OAR16_47598980 | oar3_OAR16_47699808 |
| 16 | 49634168 | 49655796 | 5 | 21629 | oar3_OAR16_49634168 | oar3_OAR16_49655796 |
| 16 | 50739166 | 50758576 | 9 | 19411 | oar3_OAR16_50739166 | oar3_OAR16_50758576 |
| 16 | 55596457 | 55596781 | 4 | 325 | oar3_OAR16_55596457 | oar3_OAR16_55596781 |
| 16 | 58391949 | 58406533 | 7 | 14585 | oar3_OAR16_58391949 | oar3_OAR16_58406533 |
| 16 | 59895143 | 59901935 | 4 | 6793 | oar3_OAR16_59895143 | oar3_OAR16_59901935 |
| 16 | 62785995 | 62828541 | 10 | 42547 | oar3_OAR16_62785995 | oar3_OAR16_62828541 |
| 16 | 66342415 | 66344550 | 5 | 2136 | oar3_OAR16_66342415 | oar3_OAR16_66344550 |
| 16 | 66342415 | 66344550 | 5 | 2136 | oar3_OAR16_66342415 | oar3_OAR16_66344550 |
| 16 | 66342415 | 66344550 | 5 | 2136 | oar3_OAR16_66342415 | oar3_OAR16_66344550 |
| 16 | 66342415 | 66344550 | 5 | 2136 | oar3_OAR16_66342415 | oar3_OAR16_66344550 |
| 16 | 67888712 | 67902724 | 5 | 14013 | oar3_OAR16_67888712 | oar3_OAR16_67902724 |
| 16 | 69200636 | 69243641 | 14 | 43006 | oar3_OAR16_69200636 | oar3_OAR16_69243641 |
| 16 | 69209408 | 69258183 | 14 | 48776 | s62581.1 | oar3_OAR16_69258183 |
| 16 | 69209408 | 69258183 | 14 | 48776 | s62581.1 | oar3_OAR16_69258183 |
| 16 | 69226093 | 69285081 | 13 | 58989 | oar3_OAR16_69226093 | oar3_OAR16_69285081 |
| 16 | 69513375 | 69562326 | 13 | 48952 | oar3_OAR16_69513375 | oar3_OAR16_69562326 |
| 16 | 69558545 | 69574670 | 8 | 16126 | oar3_OAR16_69558545 | s10704.1 |
| 16 | 69943027 | 69982025 | 12 | 38999 | oar3_OAR16_69943027 | oar3_OAR16_69982025 |
| 16 | 69946001 | 69982025 | 10 | 36025 | oar3_OAR16_69946001 | oar3_OAR16_69982025 |
| 16 | 69946001 | 69982025 | 10 | 36025 | oar3_OAR16_69946001 | oar3_OAR16_69982025 |
| 16 | 70101635 | 70119468 | 11 | 17834 | s70856.1 | oar3_OAR16_70119468 |
| 16 | 70138435 | 70197048 | 19 | 58614 | oar3_OAR16_70138435 | oar3_OAR16_70197048 |
| 16 | 70297574 | 70330183 | 9 | 32610 | oar3_OAR16_70297574 | oar3_OAR16_70330183 |
| 16 | 70355774 | 70369722 | 6 | 13949 | oar3_OAR16_70355774 | oar3_OAR16_70369722 |
| 16 | 70355774 | 70399775 | 13 | 44002 | oar3_OAR16_70355774 | oar3_OAR16_70399775 |
| 16 | 70355774 | 70369722 | 6 | 13949 | oar3_OAR16_70355774 | oar3_OAR16_70369722 |
| 16 | 70477487 | 70935630 | 51 | 458144 | oar3_OAR16_70477487 | oar3_OAR16_70935630 |
| 16 | 70522172 | 71016525 | 50 | 494354 | oar3_OAR16_70522172 | oar3_OAR16_71016525 |
| 16 | 70528271 | 70767624 | 31 | 239354 | oar3_OAR16_70528271 | oar3_OAR16_70767624 |
| 16 | 70528271 | 70820071 | 32 | 291801 | oar3_OAR16_70528271 | oar3_OAR16_70820071 |
| 16 | 70620169 | 70740412 | 19 | 120244 | oar3_OAR16_70620169 | oar3_OAR16_70740412 |
| 16 | 70664506 | 70689063 | 9 | 24558 | OARUn.54_510669.1 | oar3_OAR16_70689063 |
| 16 | 70670652 | 70689063 | 6 | 18412 | oar3_OAR16_70670652 | oar3_OAR16_70689063 |
| 16 | 70670652 | 70689063 | 6 | 18412 | oar3_OAR16_70670652 | oar3_OAR16_70689063 |
| 16 | 70682819 | 70689063 | 5 | 6245 | oar3_OAR16_70682819 | oar3_OAR16_70689063 |
| 16 | 70682819 | 70689063 | 5 | 6245 | oar3_OAR16_70682819 | oar3_OAR16_70689063 |
| 16 | 70909285 | 70990254 | 9 | 80970 | oar3_OAR16_70909285 | oar3_OAR16_70990254 |
| 16 | 70922464 | 70996291 | 8 | 73828 | s37581.1 | oar3_OAR16_70996291 |
| 16 | 70922464 | 70996291 | 8 | 73828 | s37581.1 | oar3_OAR16_70996291 |
| 16 | 70922464 | 70996291 | 8 | 73828 | s37581.1 | oar3_OAR16_70996291 |
| 16 | 70922464 | 71094219 | 30 | 171756 | s37581.1 | oar3_OAR16_71094219 |
| 16 | 70961356 | 70990254 | 3 | 28899 | oar3_OAR16_70961356 | oar3_OAR16_70990254 |
| 16 | 71202218 | 71283960 | 14 | 81743 | oar3_OAR16_71202218 | oar3_OAR16_71283960 |
| 16 | 71235386 | 71311940 | 10 | 76555 | oar3_OAR16_71235386 | oar3_OAR16_71311940 |
| 16 | 71280133 | 71311940 | 5 | 31808 | oar3_OAR16_71280133 | oar3_OAR16_71311940 |
| 16 | 71280133 | 71400046 | 19 | 119914 | oar3_OAR16_71280133 | oar3_OAR16_71400046 |
| 16 | 71280133 | 71400046 | 19 | 119914 | oar3_OAR16_71280133 | oar3_OAR16_71400046 |
| 16 | 71280133 | 71509991 | 48 | 229859 | oar3_OAR16_71280133 | oar3_OAR16_71509991 |
| 16 | 71329227 | 71504452 | 42 | 175226 | oar3_OAR16_71329227 | oar3_OAR16_71504452 |
| 16 | 71435084 | 71583619 | 44 | 148536 | oar3_OAR16_71435084 | oar3_OAR16_71583619 |
| 16 | 71447344 | 71504452 | 21 | 57109 | oar3_OAR16_71447344 | oar3_OAR16_71504452 |
| 16 | 71453950 | 71715325 | 50 | 261376 | oar3_OAR16_71453950 | oar3_OAR16_71715325 |
| 16 | 71455062 | 71504452 | 19 | 49391 | s01775.1 | oar3_OAR16_71504452 |
| 16 | 71458949 | 71574619 | 35 | 115671 | oar3_OAR16_71458949 | oar3_OAR16_71574619 |
| 16 | 71487228 | 71504452 | 10 | 17225 | oar3_OAR16_71487228 | oar3_OAR16_71504452 |
| 16 | 71500128 | 71715325 | 34 | 215198 | oar3_OAR16_71500128 | oar3_OAR16_71715325 |
| 16 | 71503932 | 71538789 | 12 | 34858 | oar3_OAR16_71503932 | oar3_OAR16_71538789 |
| 16 | 71533280 | 71537025 | 4 | 3746 | oar3_OAR16_71533280 | oar3_OAR16_71537025 |
| 16 | 71546574 | 71566227 | 6 | 19654 | oar3_OAR16_71546574 | oar3_OAR16_71566227 |
| 16 | 71551153 | 71574619 | 6 | 23467 | oar3_OAR16_71551153 | oar3_OAR16_71574619 |
| 16 | 71566140 | 71715325 | 17 | 149186 | oar3_OAR16_71566140 | oar3_OAR16_71715325 |
| 16 | 71598158 | 71715325 | 11 | 117168 | oar3_OAR16_71598158 | oar3_OAR16_71715325 |
| 16 | 71598158 | 71715325 | 11 | 117168 | oar3_OAR16_71598158 | oar3_OAR16_71715325 |
| 16 | 71623666 | 71715325 | 8 | 91660 | oar3_OAR16_71623666 | oar3_OAR16_71715325 |
| 16 | 71623666 | 71715325 | 8 | 91660 | oar3_OAR16_71623666 | oar3_OAR16_71715325 |
| 16 | 71649493 | 71715325 | 7 | 65833 | oar3_OAR16_71649493 | oar3_OAR16_71715325 |
| 16 | 71649493 | 71715325 | 7 | 65833 | oar3_OAR16_71649493 | oar3_OAR16_71715325 |
| 16 | 71649493 | 71715325 | 7 | 65833 | oar3_OAR16_71649493 | oar3_OAR16_71715325 |
| 17 | 3817397 | 3907957 | 29 | 90561 | oar3_OAR17_3817397 | oar3_OAR17_3907957 |
| 17 | 3845905 | 3879863 | 10 | 33959 | oar3_OAR17_3845905 | oar3_OAR17_3879863 |
| 17 | 3845905 | 3879193 | 9 | 33289 | oar3_OAR17_3845905 | oar3_OAR17_3879193 |
| 17 | 3845905 | 3879863 | 10 | 33959 | oar3_OAR17_3845905 | oar3_OAR17_3879863 |
| 17 | 3845905 | 3879863 | 10 | 33959 | oar3_OAR17_3845905 | oar3_OAR17_3879863 |
| 17 | 13932282 | 13937279 | 9 | 4998 | oar3_OAR17_13932282 | oar3_OAR17_13937279 |
| 17 | 36134302 | 36140720 | 4 | 6419 | oar3_OAR17_36134302 | oar3_OAR17_36140720 |
| 17 | 38083849 | 38085157 | 3 | 1309 | oar3_OAR17_38083849 | oar3_OAR17_38085157 |
| 17 | 41415836 | 41416203 | 3 | 368 | oar3_OAR17_41415836 | oar3_OAR17_41416203 |
| 17 | 44146315 | 44157703 | 6 | 11389 | oar3_OAR17_44146315 | oar3_OAR17_44157703 |
| 17 | 44153138 | 44157703 | 3 | 4566 | s57981.1 | oar3_OAR17_44157703 |
| 17 | 44153138 | 44157703 | 3 | 4566 | s57981.1 | oar3_OAR17_44157703 |
| 17 | 44214352 | 44303566 | 27 | 89215 | oar3_OAR17_44214352 | OAR17_48387667.1 |
| 17 | 44214352 | 44281811 | 20 | 67460 | oar3_OAR17_44214352 | oar3_OAR17_44281811 |
| 17 | 44536710 | 44612857 | 18 | 76148 | oar3_OAR17_44536710 | oar3_OAR17_44612857 |
| 17 | 44668808 | 44736973 | 23 | 68166 | oar3_OAR17_44668808 | oar3_OAR17_44736973 |
| 17 | 44735329 | 44841332 | 18 | 106004 | oar3_OAR17_44735329 | oar3_OAR17_44841332 |
| 17 | 45326190 | 45417321 | 18 | 91132 | oar3_OAR17_45326190 | oar3_OAR17_45417321 |
| 17 | 50957877 | 51066656 | 17 | 108780 | oar3_OAR17_50957877 | oar3_OAR17_51066656 |
| 17 | 50969961 | 51095639 | 18 | 125679 | oar3_OAR17_50969961 | oar3_OAR17_51095639 |
| 17 | 50969961 | 51066656 | 16 | 96696 | oar3_OAR17_50969961 | oar3_OAR17_51066656 |
| 17 | 50994668 | 51066656 | 12 | 71989 | oar3_OAR17_50994668 | oar3_OAR17_51066656 |
| 17 | 50994668 | 51066656 | 12 | 71989 | oar3_OAR17_50994668 | oar3_OAR17_51066656 |
| 17 | 50994668 | 51095639 | 14 | 100972 | oar3_OAR17_50994668 | oar3_OAR17_51095639 |
| 17 | 51987234 | 51996634 | 5 | 9401 | oar3_OAR17_51987234 | OAR17_56609116.1 |
| 17 | 52107896 | 52253408 | 20 | 145513 | oar3_OAR17_52107896 | oar3_OAR17_52253408 |
| 17 | 52107896 | 52152833 | 7 | 44938 | oar3_OAR17_52107896 | oar3_OAR17_52152833 |
| 17 | 52107896 | 52152833 | 7 | 44938 | oar3_OAR17_52107896 | oar3_OAR17_52152833 |
| 17 | 52120126 | 52178386 | 7 | 58261 | oar3_OAR17_52120126 | oar3_OAR17_52178386 |
| 17 | 52120126 | 52253408 | 18 | 133283 | oar3_OAR17_52120126 | oar3_OAR17_52253408 |
| 17 | 52120126 | 52152833 | 5 | 32708 | oar3_OAR17_52120126 | oar3_OAR17_52152833 |
| 17 | 52130027 | 52152833 | 4 | 22807 | s20058.1 | oar3_OAR17_52152833 |
| 17 | 52130027 | 52152833 | 4 | 22807 | s20058.1 | oar3_OAR17_52152833 |
| 17 | 52808462 | 52824124 | 5 | 15663 | oar3_OAR17_52808462 | oar3_OAR17_52824124 |
| 17 | 53073959 | 53113441 | 10 | 39483 | oar3_OAR17_53073959 | oar3_OAR17_53113441 |
| 17 | 53073959 | 53093948 | 4 | 19990 | oar3_OAR17_53073959 | oar3_OAR17_53093948 |
| 17 | 53076008 | 53106556 | 7 | 30549 | oar3_OAR17_53076008 | oar3_OAR17_53106556 |
| 17 | 53467567 | 53495739 | 7 | 28173 | oar3_OAR17_53467567 | oar3_OAR17_53495739 |
| 17 | 54664922 | 54702507 | 9 | 37586 | oar3_OAR17_54664922 | oar3_OAR17_54702507 |
| 17 | 57548353 | 57553792 | 5 | 5440 | oar3_OAR17_57548353 | oar3_OAR17_57553792 |
| 17 | 59329153 | 59330738 | 4 | 1586 | oar3_OAR17_59329153 | oar3_OAR17_59330738 |
| 17 | 62084288 | 62210304 | 26 | 126017 | oar3_OAR17_62084288 | oar3_OAR17_62210304 |
| 17 | 62084288 | 62210304 | 26 | 126017 | oar3_OAR17_62084288 | oar3_OAR17_62210304 |
| 17 | 62096410 | 62223943 | 23 | 127534 | oar3_OAR17_62096410 | oar3_OAR17_62223943 |
| 17 | 62096410 | 62103470 | 4 | 7061 | oar3_OAR17_62096410 | oar3_OAR17_62103470 |
| 17 | 62165701 | 62232611 | 11 | 66911 | oar3_OAR17_62165701 | oar3_OAR17_62232611 |
| 17 | 63045810 | 63091542 | 14 | 45733 | oar3_OAR17_63045810 | oar3_OAR17_63091542 |
| 17 | 63045810 | 63068641 | 7 | 22832 | oar3_OAR17_63045810 | oar3_OAR17_63068641 |
| 17 | 63045810 | 63077195 | 10 | 31386 | oar3_OAR17_63045810 | oar3_OAR17_63077195 |
| 17 | 63238138 | 63253146 | 7 | 15009 | oar3_OAR17_63238138 | oar3_OAR17_63253146 |
| 17 | 63471418 | 63507442 | 10 | 36025 | oar3_OAR17_63471418 | oar3_OAR17_63507442 |
| 17 | 63487317 | 63517688 | 7 | 30372 | s20296.1 | oar3_OAR17_63517688 |
| 17 | 63502018 | 63507442 | 3 | 5425 | oar3_OAR17_63502018 | oar3_OAR17_63507442 |
| 17 | 63502018 | 63528105 | 11 | 26088 | oar3_OAR17_63502018 | oar3_OAR17_63528105 |
| 17 | 64865564 | 64933950 | 19 | 68387 | oar3_OAR17_64865564 | oar3_OAR17_64933950 |
| 17 | 64865564 | 64869879 | 5 | 4316 | oar3_OAR17_64865564 | oar3_OAR17_64869879 |
| 17 | 64865564 | 64892676 | 11 | 27113 | oar3_OAR17_64865564 | oar3_OAR17_64892676 |
| 17 | 64868266 | 64873643 | 6 | 5378 | oar3_OAR17_64868266 | oar3_OAR17_64873643 |
| 17 | 65815081 | 65866138 | 13 | 51058 | oar3_OAR17_65815081 | oar3_OAR17_65866138 |
| 17 | 65815081 | 65866138 | 13 | 51058 | oar3_OAR17_65815081 | oar3_OAR17_65866138 |
| 17 | 67993369 | 68015518 | 10 | 22150 | oar3_OAR17_67993369 | oar3_OAR17_68015518 |
| 17 | 68015518 | 68059428 | 14 | 43911 | oar3_OAR17_68015518 | oar3_OAR17_68059428 |
| 17 | 68032638 | 68059428 | 10 | 26791 | s69780.1 | oar3_OAR17_68059428 |
| 17 | 68032638 | 68059428 | 10 | 26791 | s69780.1 | oar3_OAR17_68059428 |
| 17 | 68039620 | 68135585 | 24 | 95966 | oar3_OAR17_68039620 | oar3_OAR17_68135585 |
| 17 | 68054863 | 68075463 | 8 | 20601 | oar3_OAR17_68054863 | oar3_OAR17_68075463 |
| 17 | 68056394 | 68059428 | 4 | 3035 | oar3_OAR17_68056394 | oar3_OAR17_68059428 |
| 17 | 68056394 | 68075463 | 7 | 19070 | oar3_OAR17_68056394 | oar3_OAR17_68075463 |
| 17 | 68056394 | 68075463 | 7 | 19070 | oar3_OAR17_68056394 | oar3_OAR17_68075463 |
| 17 | 68117318 | 68135585 | 9 | 18268 | s22875.1 | oar3_OAR17_68135585 |
| 17 | 68117318 | 68135585 | 9 | 18268 | s22875.1 | oar3_OAR17_68135585 |
| 17 | 68117318 | 68135585 | 9 | 18268 | s22875.1 | oar3_OAR17_68135585 |
| 17 | 68349367 | 68369436 | 9 | 20070 | oar3_OAR17_68349367 | oar3_OAR17_68369436 |
| 17 | 68357082 | 68413821 | 18 | 56740 | oar3_OAR17_68357082 | oar3_OAR17_68413821 |
| 17 | 68357082 | 68460531 | 29 | 103450 | oar3_OAR17_68357082 | oar3_OAR17_68460531 |
| 17 | 68357082 | 68460531 | 29 | 103450 | oar3_OAR17_68357082 | oar3_OAR17_68460531 |
| 17 | 68357082 | 68413821 | 18 | 56740 | oar3_OAR17_68357082 | oar3_OAR17_68413821 |
| 17 | 68390997 | 68413821 | 7 | 22825 | oar3_OAR17_68390997 | oar3_OAR17_68413821 |
| 17 | 68399828 | 68413821 | 6 | 13994 | oar3_OAR17_68399828 | oar3_OAR17_68413821 |
| 17 | 68408029 | 68413821 | 5 | 5793 | oar3_OAR17_68408029 | oar3_OAR17_68413821 |
| 17 | 68442424 | 68460531 | 6 | 18108 | oar3_OAR17_68442424 | oar3_OAR17_68460531 |
| 17 | 68442424 | 68460531 | 6 | 18108 | oar3_OAR17_68442424 | oar3_OAR17_68460531 |
| 17 | 68442424 | 68456975 | 4 | 14552 | oar3_OAR17_68442424 | oar3_OAR17_68456975 |
| 17 | 68633306 | 68633485 | 3 | 180 | oar3_OAR17_68633306 | oar3_OAR17_68633485 |
| 17 | 68633306 | 68633485 | 3 | 180 | oar3_OAR17_68633306 | oar3_OAR17_68633485 |
| 17 | 68633306 | 68633485 | 3 | 180 | oar3_OAR17_68633306 | oar3_OAR17_68633485 |
| 17 | 68774463 | 68944483 | 44 | 170021 | oar3_OAR17_68774463 | oar3_OAR17_68944483 |
| 17 | 68809004 | 68851109 | 9 | 42106 | oar3_OAR17_68809004 | oar3_OAR17_68851109 |
| 17 | 68809004 | 68921596 | 29 | 112593 | oar3_OAR17_68809004 | oar3_OAR17_68921596 |
| 17 | 68809004 | 68851109 | 9 | 42106 | oar3_OAR17_68809004 | oar3_OAR17_68851109 |
| 17 | 68809004 | 68925432 | 30 | 116429 | oar3_OAR17_68809004 | oar3_OAR17_68925432 |
| 17 | 68914330 | 68944483 | 12 | 30154 | oar3_OAR17_68914330 | oar3_OAR17_68944483 |
| 17 | 68914330 | 68920176 | 3 | 5847 | oar3_OAR17_68914330 | oar3_OAR17_68920176 |
| 17 | 68914330 | 68957808 | 16 | 43479 | oar3_OAR17_68914330 | oar3_OAR17_68957808 |
| 17 | 68914330 | 68921596 | 4 | 7267 | oar3_OAR17_68914330 | oar3_OAR17_68921596 |
| 17 | 68914330 | 68921596 | 4 | 7267 | oar3_OAR17_68914330 | oar3_OAR17_68921596 |
| 17 | 69022055 | 69296314 | 69 | 274260 | oar3_OAR17_69022055 | s00507.1 |
| 17 | 69022055 | 69059048 | 12 | 36994 | oar3_OAR17_69022055 | oar3_OAR17_69059048 |
| 17 | 69026223 | 69031956 | 5 | 5734 | oar3_OAR17_69026223 | oar3_OAR17_69031956 |
| 17 | 69026223 | 69059048 | 10 | 32826 | oar3_OAR17_69026223 | oar3_OAR17_69059048 |
| 17 | 69026223 | 69059048 | 10 | 32826 | oar3_OAR17_69026223 | oar3_OAR17_69059048 |
| 17 | 69031956 | 69059048 | 6 | 27093 | oar3_OAR17_69031956 | oar3_OAR17_69059048 |
| 17 | 69038111 | 69059048 | 5 | 20938 | oar3_OAR17_69038111 | oar3_OAR17_69059048 |
| 17 | 69055418 | 69059048 | 4 | 3631 | oar3_OAR17_69055418 | oar3_OAR17_69059048 |
| 17 | 69055418 | 69059048 | 4 | 3631 | oar3_OAR17_69055418 | oar3_OAR17_69059048 |
| 17 | 69185077 | 69262147 | 24 | 77071 | oar3_OAR17_69185077 | oar3_OAR17_69262147 |
| 17 | 69226888 | 69296314 | 16 | 69427 | oar3_OAR17_69226888 | s00507.1 |
| 17 | 69404138 | 69449081 | 17 | 44944 | s49767.1 | oar3_OAR17_69449081 |
| 17 | 69404138 | 69453956 | 21 | 49819 | s49767.1 | oar3_OAR17_69453956 |
| 17 | 69408599 | 69453956 | 20 | 45358 | oar3_OAR17_69408599 | oar3_OAR17_69453956 |
| 17 | 69410736 | 69421009 | 9 | 10274 | oar3_OAR17_69410736 | oar3_OAR17_69421009 |
| 17 | 69410736 | 69453956 | 19 | 43221 | oar3_OAR17_69410736 | oar3_OAR17_69453956 |
| 17 | 69410736 | 69453956 | 19 | 43221 | oar3_OAR17_69410736 | oar3_OAR17_69453956 |
| 17 | 69410736 | 69453956 | 19 | 43221 | oar3_OAR17_69410736 | oar3_OAR17_69453956 |
| 17 | 69410736 | 69453956 | 19 | 43221 | oar3_OAR17_69410736 | oar3_OAR17_69453956 |
| 17 | 69410736 | 69490288 | 28 | 79553 | oar3_OAR17_69410736 | oar3_OAR17_69490288 |
| 17 | 69410736 | 69453956 | 19 | 43221 | oar3_OAR17_69410736 | oar3_OAR17_69453956 |
| 17 | 69410736 | 69421009 | 9 | 10274 | oar3_OAR17_69410736 | oar3_OAR17_69421009 |
| 17 | 69410736 | 69428366 | 10 | 17631 | oar3_OAR17_69410736 | oar3_OAR17_69428366 |
| 17 | 69410736 | 69453956 | 19 | 43221 | oar3_OAR17_69410736 | oar3_OAR17_69453956 |
| 17 | 69736651 | 69768576 | 12 | 31926 | oar3_OAR17_69736651 | oar3_OAR17_69768576 |
| 17 | 69736651 | 69768576 | 12 | 31926 | oar3_OAR17_69736651 | oar3_OAR17_69768576 |
| 17 | 69742928 | 69768576 | 10 | 25649 | oar3_OAR17_69742928 | oar3_OAR17_69768576 |
| 17 | 69742928 | 69768576 | 10 | 25649 | oar3_OAR17_69742928 | oar3_OAR17_69768576 |
| 17 | 69754401 | 69768576 | 6 | 14176 | oar3_OAR17_69754401 | oar3_OAR17_69768576 |
| 17 | 69754401 | 69768576 | 6 | 14176 | oar3_OAR17_69754401 | oar3_OAR17_69768576 |
| 17 | 69754401 | 69768576 | 6 | 14176 | oar3_OAR17_69754401 | oar3_OAR17_69768576 |
| 17 | 69754401 | 69768576 | 6 | 14176 | oar3_OAR17_69754401 | oar3_OAR17_69768576 |
| 17 | 70371062 | 70385003 | 5 | 13942 | oar3_OAR17_70371062 | oar3_OAR17_70385003 |
| 17 | 70371062 | 70396354 | 8 | 25293 | oar3_OAR17_70371062 | oar3_OAR17_70396354 |
| 17 | 70429423 | 70457371 | 4 | 27949 | oar3_OAR17_70429423 | oar3_OAR17_70457371 |
| 17 | 70444795 | 70523926 | 16 | 79132 | oar3_OAR17_70444795 | oar3_OAR17_70523926 |
| 17 | 70444795 | 70562725 | 23 | 117931 | oar3_OAR17_70444795 | oar3_OAR17_70562725 |
| 17 | 70457371 | 70819091 | 58 | 361721 | oar3_OAR17_70457371 | oar3_OAR17_70819091 |
| 17 | 70457371 | 70580184 | 26 | 122814 | oar3_OAR17_70457371 | oar3_OAR17_70580184 |
| 17 | 70457371 | 70515104 | 11 | 57734 | oar3_OAR17_70457371 | oar3_OAR17_70515104 |
| 17 | 70475986 | 70562725 | 17 | 86740 | oar3_OAR17_70475986 | oar3_OAR17_70562725 |
| 17 | 70507050 | 70523926 | 8 | 16877 | oar3_OAR17_70507050 | oar3_OAR17_70523926 |
| 17 | 70507050 | 70778509 | 46 | 271460 | oar3_OAR17_70507050 | oar3_OAR17_70778509 |
| 17 | 70507050 | 70580184 | 20 | 73135 | oar3_OAR17_70507050 | oar3_OAR17_70580184 |
| 17 | 70507050 | 70523926 | 8 | 16877 | oar3_OAR17_70507050 | oar3_OAR17_70523926 |
| 17 | 70507050 | 70597444 | 26 | 90395 | oar3_OAR17_70507050 | oar3_OAR17_70597444 |
| 17 | 70507050 | 70597444 | 26 | 90395 | oar3_OAR17_70507050 | oar3_OAR17_70597444 |
| 17 | 70507050 | 70778509 | 46 | 271460 | oar3_OAR17_70507050 | oar3_OAR17_70778509 |
| 17 | 70507050 | 70523926 | 8 | 16877 | oar3_OAR17_70507050 | oar3_OAR17_70523926 |
| 17 | 70507050 | 70685654 | 37 | 178605 | oar3_OAR17_70507050 | oar3_OAR17_70685654 |
| 17 | 70633095 | 70662129 | 6 | 29035 | oar3_OAR17_70633095 | oar3_OAR17_70662129 |
| 17 | 70643399 | 70778509 | 15 | 135111 | oar3_OAR17_70643399 | oar3_OAR17_70778509 |
| 17 | 70753695 | 70932532 | 35 | 178838 | oar3_OAR17_70753695 | oar3_OAR17_70932532 |
| 17 | 70770141 | 70832728 | 11 | 62588 | oar3_OAR17_70770141 | oar3_OAR17_70832728 |
| 17 | 70770141 | 70841325 | 12 | 71185 | oar3_OAR17_70770141 | oar3_OAR17_70841325 |
| 17 | 70854556 | 70915834 | 13 | 61279 | oar3_OAR17_70854556 | oar3_OAR17_70915834 |
| 17 | 70854556 | 70932532 | 16 | 77977 | oar3_OAR17_70854556 | oar3_OAR17_70932532 |
| 17 | 70854556 | 70997998 | 38 | 143443 | oar3_OAR17_70854556 | oar3_OAR17_70997998 |
| 17 | 70905215 | 70997998 | 29 | 92784 | oar3_OAR17_70905215 | oar3_OAR17_70997998 |
| 17 | 70912304 | 71022217 | 30 | 109914 | oar3_OAR17_70912304 | oar3_OAR17_71022217 |
| 17 | 70924123 | 70951663 | 10 | 27541 | s67715.1 | oar3_OAR17_70951663 |
| 17 | 70947964 | 71097650 | 27 | 149687 | oar3_OAR17_70947964 | oar3_OAR17_71097650 |
| 17 | 70966604 | 70983827 | 9 | 17224 | s66157.1 | oar3_OAR17_70983827 |
| 17 | 70966604 | 70997998 | 12 | 31395 | s66157.1 | oar3_OAR17_70997998 |
| 17 | 70966604 | 71033562 | 17 | 66959 | s66157.1 | oar3_OAR17_71033562 |
| 17 | 70966604 | 71033562 | 17 | 66959 | s66157.1 | oar3_OAR17_71033562 |
| 17 | 70966604 | 70997998 | 12 | 31395 | s66157.1 | oar3_OAR17_70997998 |
| 17 | 70973832 | 70997998 | 11 | 24167 | oar3_OAR17_70973832 | oar3_OAR17_70997998 |
| 17 | 70973832 | 70997998 | 11 | 24167 | oar3_OAR17_70973832 | oar3_OAR17_70997998 |
| 17 | 71155824 | 71230956 | 18 | 75133 | s60290.1 | oar3_OAR17_71230956 |
| 17 | 71176575 | 71227241 | 12 | 50667 | oar3_OAR17_71176575 | oar3_OAR17_71227241 |
| 17 | 71180038 | 71227241 | 10 | 47204 | oar3_OAR17_71180038 | oar3_OAR17_71227241 |
| 17 | 71194270 | 71227241 | 8 | 32972 | oar3_OAR17_71194270 | oar3_OAR17_71227241 |
| 17 | 71290034 | 71441867 | 23 | 151834 | oar3_OAR17_71290034 | oar3_OAR17_71441867 |
| 17 | 71318221 | 71770892 | 76 | 452672 | oar3_OAR17_71318221 | oar3_OAR17_71770892 |
| 17 | 71318221 | 71487732 | 27 | 169512 | oar3_OAR17_71318221 | oar3_OAR17_71487732 |
| 17 | 71318221 | 71342354 | 7 | 24134 | oar3_OAR17_71318221 | oar3_OAR17_71342354 |
| 17 | 71318221 | 71396251 | 15 | 78031 | oar3_OAR17_71318221 | oar3_OAR17_71396251 |
| 17 | 71324660 | 71417426 | 15 | 92767 | oar3_OAR17_71324660 | oar3_OAR17_71417426 |
| 17 | 71351032 | 71396251 | 8 | 45220 | oar3_OAR17_71351032 | oar3_OAR17_71396251 |
| 17 | 71364918 | 71561386 | 27 | 196469 | oar3_OAR17_71364918 | oar3_OAR17_71561386 |
| 17 | 71377220 | 71441867 | 9 | 64648 | oar3_OAR17_71377220 | oar3_OAR17_71441867 |
| 17 | 71392135 | 71582959 | 27 | 190825 | OAR17_77709936.1 | oar3_OAR17_71582959 |
| 17 | 71416714 | 71441867 | 5 | 25154 | oar3_OAR17_71416714 | oar3_OAR17_71441867 |
| 17 | 71501363 | 71907753 | 69 | 406391 | oar3_OAR17_71501363 | s50182.1 |
| 17 | 71507556 | 71582959 | 12 | 75404 | oar3_OAR17_71507556 | oar3_OAR17_71582959 |
| 17 | 71507556 | 71582959 | 12 | 75404 | oar3_OAR17_71507556 | oar3_OAR17_71582959 |
| 17 | 71511623 | 71561386 | 7 | 49764 | oar3_OAR17_71511623 | oar3_OAR17_71561386 |
| 17 | 71548639 | 71582959 | 5 | 34321 | oar3_OAR17_71548639 | oar3_OAR17_71582959 |
| 17 | 71548639 | 71582959 | 5 | 34321 | oar3_OAR17_71548639 | oar3_OAR17_71582959 |
| 17 | 71548639 | 71803922 | 48 | 255284 | oar3_OAR17_71548639 | oar3_OAR17_71803922 |
| 17 | 71561386 | 71582959 | 4 | 21574 | oar3_OAR17_71561386 | oar3_OAR17_71582959 |
| 17 | 71579833 | 71582959 | 3 | 3127 | oar3_OAR17_71579833 | oar3_OAR17_71582959 |
| 17 | 71579833 | 71582959 | 3 | 3127 | oar3_OAR17_71579833 | oar3_OAR17_71582959 |
| 17 | 71594799 | 71717061 | 28 | 122263 | oar3_OAR17_71594799 | oar3_OAR17_71717061 |
| 17 | 71613516 | 71907753 | 47 | 294238 | oar3_OAR17_71613516 | s50182.1 |
| 17 | 71640729 | 71767889 | 20 | 127161 | oar3_OAR17_71640729 | oar3_OAR17_71767889 |
| 17 | 71640729 | 71762482 | 19 | 121754 | oar3_OAR17_71640729 | oar3_OAR17_71762482 |
| 17 | 71640729 | 72118353 | 63 | 477625 | oar3_OAR17_71640729 | oar3_OAR17_72118353 |
| 17 | 71640729 | 71767889 | 20 | 127161 | oar3_OAR17_71640729 | oar3_OAR17_71767889 |
| 17 | 71640729 | 71929320 | 45 | 288592 | oar3_OAR17_71640729 | oar3_OAR17_71929320 |
| 17 | 71688536 | 71762482 | 11 | 73947 | oar3_OAR17_71688536 | oar3_OAR17_71762482 |
| 17 | 71688536 | 71907753 | 34 | 219218 | oar3_OAR17_71688536 | s50182.1 |
| 17 | 71782357 | 71816277 | 9 | 33921 | oar3_OAR17_71782357 | oar3_OAR17_71816277 |
| 17 | 71782357 | 71858569 | 13 | 76213 | oar3_OAR17_71782357 | oar3_OAR17_71858569 |
| 17 | 71782357 | 71788945 | 4 | 6589 | oar3_OAR17_71782357 | oar3_OAR17_71788945 |
| 17 | 71782357 | 71929320 | 23 | 146964 | oar3_OAR17_71782357 | oar3_OAR17_71929320 |
| 17 | 71782357 | 71803922 | 7 | 21566 | oar3_OAR17_71782357 | oar3_OAR17_71803922 |
| 17 | 71788945 | 71907753 | 17 | 118809 | oar3_OAR17_71788945 | s50182.1 |
| 17 | 71800849 | 71907753 | 16 | 106905 | oar3_OAR17_71800849 | s50182.1 |
| 17 | 71800849 | 71907753 | 16 | 106905 | oar3_OAR17_71800849 | s50182.1 |
| 17 | 71846358 | 71907753 | 10 | 61396 | oar3_OAR17_71846358 | s50182.1 |
| 17 | 71858569 | 72279291 | 42 | 420723 | oar3_OAR17_71858569 | oar3_OAR17_72279291 |
| 17 | 71858569 | 71907753 | 8 | 49185 | oar3_OAR17_71858569 | s50182.1 |
| 17 | 71869771 | 71907753 | 7 | 37983 | oar3_OAR17_71869771 | s50182.1 |
| 17 | 71869771 | 71907753 | 7 | 37983 | oar3_OAR17_71869771 | s50182.1 |
| 17 | 71890874 | 71907753 | 4 | 16880 | oar3_OAR17_71890874 | s50182.1 |
| 17 | 71890874 | 71929320 | 7 | 38447 | oar3_OAR17_71890874 | oar3_OAR17_71929320 |
| 17 | 72025845 | 72279291 | 24 | 253447 | oar3_OAR17_72025845 | oar3_OAR17_72279291 |
| 17 | 72025845 | 72072667 | 8 | 46823 | oar3_OAR17_72025845 | oar3_OAR17_72072667 |
| 17 | 72025845 | 72052932 | 5 | 27088 | oar3_OAR17_72025845 | oar3_OAR17_72052932 |
| 17 | 72025845 | 72279291 | 24 | 253447 | oar3_OAR17_72025845 | oar3_OAR17_72279291 |
| 17 | 72025845 | 72243814 | 22 | 217970 | oar3_OAR17_72025845 | oar3_OAR17_72243814 |
| 17 | 72032099 | 72243814 | 21 | 211716 | oar3_OAR17_72032099 | oar3_OAR17_72243814 |
| 17 | 72032099 | 72279291 | 23 | 247193 | oar3_OAR17_72032099 | oar3_OAR17_72279291 |
| 17 | 72043885 | 72118353 | 9 | 74469 | oar3_OAR17_72043885 | oar3_OAR17_72118353 |
| 17 | 72096845 | 72128237 | 4 | 31393 | s27077.1 | oar3_OAR17_72128237 |
| 17 | 72181748 | 72243814 | 5 | 62067 | oar3_OAR17_72181748 | oar3_OAR17_72243814 |
| 18 | 4928846 | 4971803 | 9 | 42958 | oar3_OAR18_4928846 | oar3_OAR18_4971803 |
| 18 | 6968921 | 7065862 | 29 | 96942 | oar3_OAR18_6968921 | oar3_OAR18_7065862 |
| 18 | 9697874 | 9716616 | 4 | 18743 | OAR18_9552573.1 | oar3_OAR18_9716616 |
| 18 | 12208388 | 12646915 | 96 | 438528 | oar3_OAR18_12208388 | oar3_OAR18_12646915 |
| 18 | 15570138 | 15574042 | 11 | 3905 | oar3_OAR18_15570138 | oar3_OAR18_15574042 |
| 18 | 15570138 | 15574042 | 11 | 3905 | oar3_OAR18_15570138 | oar3_OAR18_15574042 |
| 18 | 15571694 | 15591799 | 14 | 20106 | oar3_OAR18_15571694 | oar3_OAR18_15591799 |
| 18 | 15571694 | 15574042 | 10 | 2349 | oar3_OAR18_15571694 | oar3_OAR18_15574042 |
| 18 | 15571694 | 15572875 | 6 | 1182 | oar3_OAR18_15571694 | oar3_OAR18_15572875 |
| 18 | 15571694 | 15574042 | 10 | 2349 | oar3_OAR18_15571694 | oar3_OAR18_15574042 |
| 18 | 15571694 | 15574042 | 10 | 2349 | oar3_OAR18_15571694 | oar3_OAR18_15574042 |
| 18 | 15572338 | 15576776 | 10 | 4439 | oar3_OAR18_15572338 | oar3_OAR18_15576776 |
| 18 | 15572338 | 15574042 | 9 | 1705 | oar3_OAR18_15572338 | oar3_OAR18_15574042 |
| 18 | 15572338 | 15574042 | 9 | 1705 | oar3_OAR18_15572338 | oar3_OAR18_15574042 |
| 18 | 15572338 | 15576776 | 10 | 4439 | oar3_OAR18_15572338 | oar3_OAR18_15576776 |
| 18 | 15572773 | 15576776 | 8 | 4004 | oar3_OAR18_15572773 | oar3_OAR18_15576776 |
| 18 | 15573754 | 15574042 | 4 | 289 | oar3_OAR18_15573754 | oar3_OAR18_15574042 |
| 18 | 15693402 | 15733526 | 14 | 40125 | oar3_OAR18_15693402 | oar3_OAR18_15733526 |
| 18 | 15713073 | 15781109 | 18 | 68037 | oar3_OAR18_15713073 | oar3_OAR18_15781109 |
| 18 | 15713073 | 15781109 | 18 | 68037 | oar3_OAR18_15713073 | oar3_OAR18_15781109 |
| 18 | 18724672 | 18807401 | 21 | 82730 | oar3_OAR18_18724672 | oar3_OAR18_18807401 |
| 18 | 18764204 | 18807401 | 10 | 43198 | oar3_OAR18_18764204 | oar3_OAR18_18807401 |
| 18 | 18764204 | 18807401 | 10 | 43198 | oar3_OAR18_18764204 | oar3_OAR18_18807401 |
| 18 | 19528729 | 19599474 | 15 | 70746 | oar3_OAR18_19528729 | oar3_OAR18_19599474 |
| 18 | 20164883 | 20201439 | 14 | 36557 | oar3_OAR18_20164883 | oar3_OAR18_20201439 |
| 18 | 20255185 | 20317531 | 26 | 62347 | oar3_OAR18_20255185 | OAR18_20817623.1 |
| 18 | 20255185 | 20317531 | 26 | 62347 | oar3_OAR18_20255185 | OAR18_20817623.1 |
| 18 | 20255185 | 20303327 | 16 | 48143 | oar3_OAR18_20255185 | oar3_OAR18_20303327 |
| 18 | 20518649 | 20688403 | 34 | 169755 | oar3_OAR18_20518649 | oar3_OAR18_20688403 |
| 18 | 20555849 | 20592729 | 10 | 36881 | oar3_OAR18_20555849 | oar3_OAR18_20592729 |
| 18 | 20562401 | 20585746 | 7 | 23346 | oar3_OAR18_20562401 | oar3_OAR18_20585746 |
| 18 | 20572820 | 20616424 | 11 | 43605 | oar3_OAR18_20572820 | oar3_OAR18_20616424 |
| 18 | 20572820 | 20688403 | 25 | 115584 | oar3_OAR18_20572820 | oar3_OAR18_20688403 |
| 18 | 20684866 | 20735451 | 13 | 50586 | oar3_OAR18_20684866 | oar3_OAR18_20735451 |
| 18 | 20684866 | 20716571 | 9 | 31706 | oar3_OAR18_20684866 | oar3_OAR18_20716571 |
| 18 | 20684866 | 20735451 | 13 | 50586 | oar3_OAR18_20684866 | oar3_OAR18_20735451 |
| 18 | 20812528 | 20906946 | 25 | 94419 | oar3_OAR18_20812528 | OAR18_21489818.1 |
| 18 | 20819180 | 20906946 | 24 | 87767 | oar3_OAR18_20819180 | OAR18_21489818.1 |
| 18 | 20826655 | 20893147 | 19 | 66493 | oar3_OAR18_20826655 | oar3_OAR18_20893147 |
| 18 | 20851851 | 20893147 | 13 | 41297 | oar3_OAR18_20851851 | oar3_OAR18_20893147 |
| 18 | 20865439 | 20893147 | 9 | 27709 | oar3_OAR18_20865439 | oar3_OAR18_20893147 |
| 18 | 20865439 | 20898908 | 10 | 33470 | oar3_OAR18_20865439 | oar3_OAR18_20898908 |
| 18 | 20886055 | 20893147 | 4 | 7093 | oar3_OAR18_20886055 | oar3_OAR18_20893147 |
| 18 | 21062983 | 21078370 | 7 | 15388 | oar3_OAR18_21062983 | oar3_OAR18_21078370 |
| 18 | 21411245 | 21419007 | 7 | 7763 | oar3_OAR18_21411245 | oar3_OAR18_21419007 |
| 18 | 24327736 | 24349186 | 8 | 21451 | oar3_OAR18_24327736 | oar3_OAR18_24349186 |
| 18 | 26117515 | 26138361 | 7 | 20847 | oar3_OAR18_26117515 | oar3_OAR18_26138361 |
| 18 | 26117515 | 26144037 | 8 | 26523 | oar3_OAR18_26117515 | oar3_OAR18_26144037 |
| 18 | 26521136 | 26523658 | 5 | 2523 | oar3_OAR18_26521136 | oar3_OAR18_26523658 |
| 18 | 26521136 | 26754749 | 36 | 233614 | oar3_OAR18_26521136 | OAR18_27752153.1 |
| 18 | 26521136 | 26523658 | 5 | 2523 | oar3_OAR18_26521136 | oar3_OAR18_26523658 |
| 18 | 27121623 | 27153363 | 16 | 31741 | oar3_OAR18_27121623 | oar3_OAR18_27153363 |
| 18 | 27121623 | 27147013 | 15 | 25391 | oar3_OAR18_27121623 | oar3_OAR18_27147013 |
| 18 | 27126414 | 27153363 | 13 | 26950 | s28933.1 | oar3_OAR18_27153363 |
| 18 | 27998817 | 28050377 | 19 | 51561 | oar3_OAR18_27998817 | oar3_OAR18_28050377 |
| 18 | 28000921 | 28014455 | 8 | 13535 | oar3_OAR18_28000921 | oar3_OAR18_28014455 |
| 18 | 28042652 | 28177915 | 24 | 135264 | oar3_OAR18_28042652 | oar3_OAR18_28177915 |
| 18 | 29349010 | 29427922 | 16 | 78913 | oar3_OAR18_29349010 | oar3_OAR18_29427922 |
| 18 | 29349010 | 29369061 | 6 | 20052 | oar3_OAR18_29349010 | oar3_OAR18_29369061 |
| 18 | 29536092 | 29537855 | 5 | 1764 | oar3_OAR18_29536092 | oar3_OAR18_29537855 |
| 18 | 30461647 | 30468944 | 7 | 7298 | oar3_OAR18_30461647 | oar3_OAR18_30468944 |
| 18 | 30461647 | 30502431 | 17 | 40785 | oar3_OAR18_30461647 | oar3_OAR18_30502431 |
| 18 | 30467621 | 30468944 | 4 | 1324 | oar3_OAR18_30467621 | oar3_OAR18_30468944 |
| 18 | 31123765 | 31177877 | 18 | 54113 | oar3_OAR18_31123765 | oar3_OAR18_31177877 |
| 18 | 31139647 | 31177877 | 13 | 38231 | oar3_OAR18_31139647 | oar3_OAR18_31177877 |
| 18 | 31139647 | 31177877 | 13 | 38231 | oar3_OAR18_31139647 | oar3_OAR18_31177877 |
| 18 | 31172865 | 31177877 | 6 | 5013 | s17874.1 | oar3_OAR18_31177877 |
| 18 | 31172865 | 31177877 | 6 | 5013 | s17874.1 | oar3_OAR18_31177877 |
| 18 | 31786788 | 31901118 | 36 | 114331 | oar3_OAR18_31786788 | oar3_OAR18_31901118 |
| 18 | 31799476 | 31826177 | 6 | 26702 | oar3_OAR18_31799476 | oar3_OAR18_31826177 |
| 18 | 31988016 | 32013765 | 10 | 25750 | oar3_OAR18_31988016 | oar3_OAR18_32013765 |
| 18 | 32000557 | 32013765 | 6 | 13209 | oar3_OAR18_32000557 | oar3_OAR18_32013765 |
| 18 | 32046093 | 32119173 | 26 | 73081 | oar3_OAR18_32046093 | oar3_OAR18_32119173 |
| 18 | 32046093 | 32148498 | 34 | 102406 | oar3_OAR18_32046093 | oar3_OAR18_32148498 |
| 18 | 32046093 | 32058174 | 8 | 12082 | oar3_OAR18_32046093 | oar3_OAR18_32058174 |
| 18 | 32054968 | 32136593 | 25 | 81626 | OAR18_33440951.1 | oar3_OAR18_32136593 |
| 18 | 32082322 | 32131764 | 18 | 49443 | oar3_OAR18_32082322 | oar3_OAR18_32131764 |
| 18 | 32084397 | 32131764 | 17 | 47368 | oar3_OAR18_32084397 | oar3_OAR18_32131764 |
| 18 | 32099419 | 32106701 | 7 | 7283 | oar3_OAR18_32099419 | oar3_OAR18_32106701 |
| 18 | 32099419 | 32131764 | 11 | 32346 | oar3_OAR18_32099419 | oar3_OAR18_32131764 |
| 18 | 32418745 | 32452463 | 8 | 33719 | oar3_OAR18_32418745 | s08407.1 |
| 18 | 32418745 | 32452463 | 8 | 33719 | oar3_OAR18_32418745 | s08407.1 |
| 18 | 32418745 | 32487822 | 13 | 69078 | oar3_OAR18_32418745 | oar3_OAR18_32487822 |
| 18 | 32418745 | 32487822 | 13 | 69078 | oar3_OAR18_32418745 | oar3_OAR18_32487822 |
| 18 | 32450099 | 32487822 | 10 | 37724 | oar3_OAR18_32450099 | oar3_OAR18_32487822 |
| 18 | 32450099 | 32487822 | 10 | 37724 | oar3_OAR18_32450099 | oar3_OAR18_32487822 |
| 18 | 32450099 | 32487822 | 10 | 37724 | oar3_OAR18_32450099 | oar3_OAR18_32487822 |
| 18 | 32707811 | 32838428 | 20 | 130618 | oar3_OAR18_32707811 | oar3_OAR18_32838428 |
| 18 | 32756020 | 32838428 | 15 | 82409 | oar3_OAR18_32756020 | oar3_OAR18_32838428 |
| 18 | 33362062 | 33401638 | 14 | 39577 | s62663.1 | oar3_OAR18_33401638 |
| 18 | 33362062 | 33389758 | 11 | 27697 | s62663.1 | oar3_OAR18_33389758 |
| 18 | 33373663 | 33389758 | 8 | 16096 | oar3_OAR18_33373663 | oar3_OAR18_33389758 |
| 18 | 33545911 | 33577387 | 10 | 31477 | oar3_OAR18_33545911 | oar3_OAR18_33577387 |
| 18 | 40486126 | 40510688 | 6 | 24563 | oar3_OAR18_40486126 | oar3_OAR18_40510688 |
| 18 | 54236103 | 54242511 | 5 | 6409 | oar3_OAR18_54236103 | oar3_OAR18_54242511 |
| 18 | 56237914 | 56287993 | 10 | 50080 | oar3_OAR18_56237914 | oar3_OAR18_56287993 |
| 18 | 56531193 | 56592387 | 13 | 61195 | oar3_OAR18_56531193 | oar3_OAR18_56592387 |
| 18 | 57303783 | 57315672 | 6 | 11890 | oar3_OAR18_57303783 | oar3_OAR18_57315672 |
| 18 | 60142052 | 60150651 | 6 | 8600 | oar3_OAR18_60142052 | oar3_OAR18_60150651 |
| 18 | 63067268 | 63386051 | 78 | 318784 | oar3_OAR18_63067268 | oar3_OAR18_63386051 |
| 18 | 63169896 | 63299958 | 28 | 130063 | oar3_OAR18_63169896 | oar3_OAR18_63299958 |
| 18 | 63201548 | 63312624 | 24 | 111077 | oar3_OAR18_63201548 | oar3_OAR18_63312624 |
| 18 | 63287010 | 63374328 | 20 | 87319 | oar3_OAR18_63287010 | s55964.1 |
| 18 | 63702493 | 63769940 | 20 | 67448 | oar3_OAR18_63702493 | OAR18_67715847_X.1 |
| 18 | 63707987 | 63781279 | 20 | 73293 | oar3_OAR18_63707987 | oar3_OAR18_63781279 |
| 18 | 63722345 | 63769940 | 14 | 47596 | oar3_OAR18_63722345 | OAR18_67715847_X.1 |
| 18 | 63743861 | 63781279 | 10 | 37419 | oar3_OAR18_63743861 | oar3_OAR18_63781279 |
| 18 | 63765553 | 63865531 | 24 | 99979 | oar3_OAR18_63765553 | oar3_OAR18_63865531 |
| 18 | 63765553 | 63769940 | 4 | 4388 | oar3_OAR18_63765553 | OAR18_67715847_X.1 |
| 18 | 63823801 | 63848745 | 9 | 24945 | oar3_OAR18_63823801 | oar3_OAR18_63848745 |
| 18 | 63823801 | 63946456 | 27 | 122656 | oar3_OAR18_63823801 | oar3_OAR18_63946456 |
| 18 | 63823801 | 63941421 | 25 | 117621 | oar3_OAR18_63823801 | oar3_OAR18_63941421 |
| 18 | 63830156 | 63943777 | 24 | 113622 | s54088.1 | oar3_OAR18_63943777 |
| 18 | 63834719 | 63946456 | 22 | 111738 | oar3_OAR18_63834719 | oar3_OAR18_63946456 |
| 18 | 63859241 | 63943777 | 17 | 84537 | oar3_OAR18_63859241 | oar3_OAR18_63943777 |
| 18 | 63865531 | 63943777 | 15 | 78247 | oar3_OAR18_63865531 | oar3_OAR18_63943777 |
| 18 | 63865531 | 63946456 | 16 | 80926 | oar3_OAR18_63865531 | oar3_OAR18_63946456 |
| 18 | 64070746 | 64135407 | 13 | 64662 | oar3_OAR18_64070746 | oar3_OAR18_64135407 |
| 18 | 64070746 | 64162095 | 20 | 91350 | oar3_OAR18_64070746 | oar3_OAR18_64162095 |
| 18 | 64085426 | 64123422 | 10 | 37997 | oar3_OAR18_64085426 | oar3_OAR18_64123422 |
| 18 | 64089984 | 64123422 | 8 | 33439 | oar3_OAR18_64089984 | oar3_OAR18_64123422 |
| 18 | 64114754 | 64162095 | 13 | 47342 | oar3_OAR18_64114754 | oar3_OAR18_64162095 |
| 18 | 64135407 | 64168341 | 10 | 32935 | oar3_OAR18_64135407 | s38582.1 |
| 18 | 64475489 | 64481341 | 6 | 5853 | oar3_OAR18_64475489 | oar3_OAR18_64481341 |
| 18 | 64475489 | 64481341 | 6 | 5853 | oar3_OAR18_64475489 | oar3_OAR18_64481341 |
| 18 | 64623786 | 64673093 | 10 | 49308 | oar3_OAR18_64623786 | oar3_OAR18_64673093 |
| 18 | 64634064 | 64737895 | 20 | 103832 | oar3_OAR18_64634064 | oar3_OAR18_64737895 |
| 18 | 65096063 | 65191422 | 24 | 95360 | oar3_OAR18_65096063 | oar3_OAR18_65191422 |
| 18 | 65150773 | 65211766 | 15 | 60994 | oar3_OAR18_65150773 | OAR18_69099015.1 |
| 18 | 65189818 | 65211766 | 6 | 21949 | oar3_OAR18_65189818 | OAR18_69099015.1 |
| 18 | 65189818 | 65191422 | 3 | 1605 | oar3_OAR18_65189818 | oar3_OAR18_65191422 |
| 18 | 65189818 | 65191422 | 3 | 1605 | oar3_OAR18_65189818 | oar3_OAR18_65191422 |
| 18 | 65189818 | 65276464 | 17 | 86647 | oar3_OAR18_65189818 | oar3_OAR18_65276464 |
| 18 | 65189818 | 65191422 | 3 | 1605 | oar3_OAR18_65189818 | oar3_OAR18_65191422 |
| 18 | 65814031 | 66011558 | 37 | 197528 | oar3_OAR18_65814031 | oar3_OAR18_66011558 |
| 18 | 65893773 | 65905295 | 6 | 11523 | oar3_OAR18_65893773 | oar3_OAR18_65905295 |
| 18 | 65922104 | 66011558 | 13 | 89455 | oar3_OAR18_65922104 | oar3_OAR18_66011558 |
| 18 | 65936392 | 66011558 | 10 | 75167 | oar3_OAR18_65936392 | oar3_OAR18_66011558 |
| 18 | 65952807 | 66011558 | 8 | 58752 | oar3_OAR18_65952807 | oar3_OAR18_66011558 |
| 18 | 66381431 | 66467825 | 13 | 86395 | oar3_OAR18_66381431 | oar3_OAR18_66467825 |
| 18 | 66381431 | 66645322 | 32 | 263892 | oar3_OAR18_66381431 | oar3_OAR18_66645322 |
| 18 | 66398130 | 66607922 | 23 | 209793 | oar3_OAR18_66398130 | oar3_OAR18_66607922 |
| 18 | 66463737 | 66673992 | 25 | 210256 | oar3_OAR18_66463737 | oar3_OAR18_66673992 |
| 18 | 66463737 | 66592925 | 14 | 129189 | oar3_OAR18_66463737 | oar3_OAR18_66592925 |
| 18 | 66819611 | 66870885 | 11 | 51275 | oar3_OAR18_66819611 | oar3_OAR18_66870885 |
| 18 | 66834768 | 66911468 | 20 | 76701 | oar3_OAR18_66834768 | oar3_OAR18_66911468 |
| 18 | 66834768 | 66908201 | 18 | 73434 | oar3_OAR18_66834768 | oar3_OAR18_66908201 |
| 18 | 66850236 | 66870885 | 6 | 20650 | oar3_OAR18_66850236 | oar3_OAR18_66870885 |
| 18 | 66857743 | 66877195 | 7 | 19453 | oar3_OAR18_66857743 | oar3_OAR18_66877195 |
| 18 | 66899088 | 67030569 | 36 | 131482 | oar3_OAR18_66899088 | oar3_OAR18_67030569 |
| 18 | 66899088 | 66911468 | 5 | 12381 | oar3_OAR18_66899088 | oar3_OAR18_66911468 |
| 18 | 66908201 | 67014134 | 27 | 105934 | oar3_OAR18_66908201 | oar3_OAR18_67014134 |
| 18 | 66980508 | 67014134 | 9 | 33627 | oar3_OAR18_66980508 | oar3_OAR18_67014134 |
| 18 | 66980508 | 67005849 | 5 | 25342 | oar3_OAR18_66980508 | oar3_OAR18_67005849 |
| 18 | 66980508 | 67005849 | 5 | 25342 | oar3_OAR18_66980508 | oar3_OAR18_67005849 |
| 18 | 66980508 | 67026870 | 14 | 46363 | oar3_OAR18_66980508 | oar3_OAR18_67026870 |
| 18 | 66980508 | 67026870 | 14 | 46363 | oar3_OAR18_66980508 | oar3_OAR18_67026870 |
| 18 | 67075580 | 67118151 | 14 | 42572 | oar3_OAR18_67075580 | oar3_OAR18_67118151 |
| 18 | 67082273 | 67118151 | 13 | 35879 | oar3_OAR18_67082273 | oar3_OAR18_67118151 |
| 18 | 67106299 | 67118151 | 5 | 11853 | oar3_OAR18_67106299 | oar3_OAR18_67118151 |
| 18 | 67248769 | 67296526 | 6 | 47758 | oar3_OAR18_67248769 | oar3_OAR18_67296526 |
| 18 | 67248769 | 67296526 | 6 | 47758 | oar3_OAR18_67248769 | oar3_OAR18_67296526 |
| 18 | 67248769 | 67296526 | 6 | 47758 | oar3_OAR18_67248769 | oar3_OAR18_67296526 |
| 18 | 67248769 | 67296526 | 6 | 47758 | oar3_OAR18_67248769 | oar3_OAR18_67296526 |
| 18 | 67248769 | 67335156 | 10 | 86388 | oar3_OAR18_67248769 | oar3_OAR18_67335156 |
| 18 | 67289143 | 67296526 | 4 | 7384 | s48176.1 | oar3_OAR18_67296526 |
| 18 | 67289143 | 67345207 | 10 | 56065 | s48176.1 | oar3_OAR18_67345207 |
| 18 | 67355317 | 67373377 | 9 | 18061 | oar3_OAR18_67355317 | oar3_OAR18_67373377 |
| 18 | 67382206 | 67536427 | 28 | 154222 | oar3_OAR18_67382206 | oar3_OAR18_67536427 |
| 18 | 67450092 | 67536427 | 14 | 86336 | oar3_OAR18_67450092 | oar3_OAR18_67536427 |
| 18 | 67450092 | 67451842 | 3 | 1751 | oar3_OAR18_67450092 | oar3_OAR18_67451842 |
| 18 | 67511075 | 67628681 | 19 | 117607 | oar3_OAR18_67511075 | oar3_OAR18_67628681 |
| 18 | 67511075 | 67536427 | 7 | 25353 | oar3_OAR18_67511075 | oar3_OAR18_67536427 |
| 18 | 67589338 | 67628681 | 8 | 39344 | oar3_OAR18_67589338 | oar3_OAR18_67628681 |
| 18 | 67592275 | 67628681 | 7 | 36407 | oar3_OAR18_67592275 | oar3_OAR18_67628681 |
| 18 | 67677285 | 67696897 | 6 | 19613 | oar3_OAR18_67677285 | s09007.1 |
| 18 | 67677285 | 67706649 | 9 | 29365 | oar3_OAR18_67677285 | oar3_OAR18_67706649 |
| 18 | 67719519 | 67737127 | 7 | 17609 | oar3_OAR18_67719519 | oar3_OAR18_67737127 |
| 18 | 67728155 | 67857925 | 25 | 129771 | oar3_OAR18_67728155 | oar3_OAR18_67857925 |
| 18 | 67771953 | 67854662 | 15 | 82710 | oar3_OAR18_67771953 | oar3_OAR18_67854662 |
| 18 | 67791927 | 67842433 | 12 | 50507 | oar3_OAR18_67791927 | oar3_OAR18_67842433 |
| 18 | 67795911 | 67813578 | 4 | 17668 | oar3_OAR18_67795911 | oar3_OAR18_67813578 |
| 18 | 67795911 | 67877789 | 15 | 81879 | oar3_OAR18_67795911 | oar3_OAR18_67877789 |
| 18 | 67796502 | 67857925 | 13 | 61424 | oar3_OAR18_67796502 | oar3_OAR18_67857925 |
| 18 | 67796502 | 68009502 | 31 | 213001 | oar3_OAR18_67796502 | oar3_OAR18_68009502 |
| 18 | 67813578 | 67842433 | 8 | 28856 | oar3_OAR18_67813578 | oar3_OAR18_67842433 |
| 18 | 67813578 | 67842433 | 8 | 28856 | oar3_OAR18_67813578 | oar3_OAR18_67842433 |
| 18 | 67813578 | 67842433 | 8 | 28856 | oar3_OAR18_67813578 | oar3_OAR18_67842433 |
| 18 | 67813578 | 67842433 | 8 | 28856 | oar3_OAR18_67813578 | oar3_OAR18_67842433 |
| 18 | 67813578 | 67842433 | 8 | 28856 | oar3_OAR18_67813578 | oar3_OAR18_67842433 |
| 18 | 67813578 | 67832748 | 6 | 19171 | oar3_OAR18_67813578 | oar3_OAR18_67832748 |
| 18 | 67813578 | 67842433 | 8 | 28856 | oar3_OAR18_67813578 | oar3_OAR18_67842433 |
| 18 | 67813578 | 67842433 | 8 | 28856 | oar3_OAR18_67813578 | oar3_OAR18_67842433 |
| 18 | 67817610 | 67821799 | 4 | 4190 | s44952.1 | oar3_OAR18_67821799 |
| 18 | 67832748 | 67842433 | 3 | 9686 | oar3_OAR18_67832748 | oar3_OAR18_67842433 |
| 18 | 67854662 | 67990106 | 19 | 135445 | oar3_OAR18_67854662 | oar3_OAR18_67990106 |
| 18 | 67948508 | 67957856 | 4 | 9349 | oar3_OAR18_67948508 | oar3_OAR18_67957856 |
| 18 | 67948508 | 68009502 | 11 | 60995 | oar3_OAR18_67948508 | oar3_OAR18_68009502 |
| 18 | 67948508 | 68068956 | 18 | 120449 | oar3_OAR18_67948508 | oar3_OAR18_68068956 |
| 18 | 67986707 | 68078914 | 16 | 92208 | oar3_OAR18_67986707 | oar3_OAR18_68078914 |
| 18 | 68036923 | 68081384 | 9 | 44462 | oar3_OAR18_68036923 | oar3_OAR18_68081384 |
| 18 | 68074663 | 68257085 | 26 | 182423 | oar3_OAR18_68074663 | oar3_OAR18_68257085 |
| 18 | 68137231 | 68169675 | 5 | 32445 | s03219.1 | oar3_OAR18_68169675 |
| 18 | 68168043 | 68257085 | 11 | 89043 | oar3_OAR18_68168043 | oar3_OAR18_68257085 |
| 18 | 68168043 | 68557575 | 54 | 389533 | oar3_OAR18_68168043 | oar3_OAR18_68557575 |
| 18 | 68168043 | 68174999 | 5 | 6957 | oar3_OAR18_68168043 | oar3_OAR18_68174999 |
| 18 | 68168043 | 68257085 | 11 | 89043 | oar3_OAR18_68168043 | oar3_OAR18_68257085 |
| 18 | 68168043 | 68261569 | 12 | 93527 | oar3_OAR18_68168043 | oar3_OAR18_68261569 |
| 18 | 68169675 | 68257085 | 9 | 87411 | oar3_OAR18_68169675 | oar3_OAR18_68257085 |
| 18 | 68173648 | 68261569 | 9 | 87922 | oar3_OAR18_68173648 | oar3_OAR18_68261569 |
| 18 | 68227120 | 68257085 | 6 | 29966 | oar3_OAR18_68227120 | oar3_OAR18_68257085 |
| 18 | 68227120 | 68404225 | 24 | 177106 | oar3_OAR18_68227120 | oar3_OAR18_68404225 |
| 18 | 68227120 | 68257085 | 6 | 29966 | oar3_OAR18_68227120 | oar3_OAR18_68257085 |
| 18 | 68250265 | 68257085 | 5 | 6821 | oar3_OAR18_68250265 | oar3_OAR18_68257085 |
| 18 | 68250265 | 68378419 | 19 | 128155 | oar3_OAR18_68250265 | oar3_OAR18_68378419 |
| 18 | 68276652 | 68302689 | 4 | 26038 | oar3_OAR18_68276652 | oar3_OAR18_68302689 |
| 18 | 68340173 | 68386549 | 6 | 46377 | oar3_OAR18_68340173 | oar3_OAR18_68386549 |
| 18 | 68340173 | 68401733 | 8 | 61561 | oar3_OAR18_68340173 | oar3_OAR18_68401733 |
| 18 | 68378023 | 68401733 | 5 | 23711 | s13467.1 | oar3_OAR18_68401733 |
| 18 | 68378023 | 68450168 | 15 | 72146 | s13467.1 | s08970.1 |
| 18 | 68378023 | 68414625 | 7 | 36603 | s13467.1 | oar3_OAR18_68414625 |
| 18 | 68378023 | 68398710 | 4 | 20688 | s13467.1 | oar3_OAR18_68398710 |
| 18 | 68378023 | 68480722 | 21 | 102700 | s13467.1 | oar3_OAR18_68480722 |
| 18 | 68401733 | 68490423 | 18 | 88691 | oar3_OAR18_68401733 | oar3_OAR18_68490423 |
| 18 | 68446251 | 68478293 | 13 | 32043 | oar3_OAR18_68446251 | oar3_OAR18_68478293 |
| 19 | 6085329 | 6088550 | 6 | 3222 | oar3_OAR19_6085329 | oar3_OAR19_6088550 |
| 19 | 11385626 | 11414951 | 10 | 29326 | oar3_OAR19_11385626 | oar3_OAR19_11414951 |
| 19 | 11385626 | 11414951 | 10 | 29326 | oar3_OAR19_11385626 | oar3_OAR19_11414951 |
| 19 | 11488588 | 11524505 | 11 | 35918 | oar3_OAR19_11488588 | oar3_OAR19_11524505 |
| 19 | 11506428 | 11524505 | 8 | 18078 | oar3_OAR19_11506428 | oar3_OAR19_11524505 |
| 19 | 11760286 | 11790997 | 11 | 30712 | oar3_OAR19_11760286 | oar3_OAR19_11790997 |
| 19 | 11894194 | 11901045 | 5 | 6852 | oar3_OAR19_11894194 | OAR19_12320210.1 |
| 19 | 12377666 | 12428416 | 22 | 50751 | oar3_OAR19_12377666 | oar3_OAR19_12428416 |
| 19 | 12381568 | 12428416 | 19 | 46849 | oar3_OAR19_12381568 | oar3_OAR19_12428416 |
| 19 | 12395124 | 12428416 | 12 | 33293 | oar3_OAR19_12395124 | oar3_OAR19_12428416 |
| 19 | 12417748 | 12428416 | 8 | 10669 | oar3_OAR19_12417748 | oar3_OAR19_12428416 |
| 19 | 14581240 | 14650060 | 15 | 68821 | oar3_OAR19_14581240 | oar3_OAR19_14650060 |
| 19 | 14710638 | 14760733 | 9 | 50096 | oar3_OAR19_14710638 | oar3_OAR19_14760733 |
| 19 | 15217562 | 15229759 | 8 | 12198 | oar3_OAR19_15217562 | oar3_OAR19_15229759 |
| 19 | 15217562 | 15229759 | 8 | 12198 | oar3_OAR19_15217562 | oar3_OAR19_15229759 |
| 19 | 15217562 | 15242092 | 12 | 24531 | oar3_OAR19_15217562 | oar3_OAR19_15242092 |
| 19 | 15217562 | 15229759 | 8 | 12198 | oar3_OAR19_15217562 | oar3_OAR19_15229759 |
| 19 | 15217562 | 15229759 | 8 | 12198 | oar3_OAR19_15217562 | oar3_OAR19_15229759 |
| 19 | 16280980 | 16287754 | 5 | 6775 | oar3_OAR19_16280980 | oar3_OAR19_16287754 |
| 19 | 24623838 | 24626886 | 4 | 3049 | oar3_OAR19_24623838 | oar3_OAR19_24626886 |
| 19 | 24676674 | 24696438 | 6 | 19765 | oar3_OAR19_24676674 | oar3_OAR19_24696438 |
| 19 | 26393639 | 26394805 | 3 | 1167 | oar3_OAR19_26393639 | oar3_OAR19_26394805 |
| 19 | 26393639 | 26394805 | 3 | 1167 | oar3_OAR19_26393639 | oar3_OAR19_26394805 |
| 19 | 42815145 | 42859902 | 14 | 44758 | oar3_OAR19_42815145 | oar3_OAR19_42859902 |
| 19 | 47156464 | 47177337 | 8 | 20874 | oar3_OAR19_47156464 | oar3_OAR19_47177337 |
| 19 | 47156464 | 47177337 | 8 | 20874 | oar3_OAR19_47156464 | oar3_OAR19_47177337 |
| 19 | 47747884 | 47749401 | 3 | 1518 | oar3_OAR19_47747884 | oar3_OAR19_47749401 |
| 19 | 47747884 | 47749401 | 3 | 1518 | oar3_OAR19_47747884 | oar3_OAR19_47749401 |
| 19 | 48335315 | 48432080 | 31 | 96766 | s42197.1 | oar3_OAR19_48432080 |
| 19 | 48340190 | 48348120 | 4 | 7931 | oar3_OAR19_48340190 | oar3_OAR19_48348120 |
| 19 | 48389751 | 48414050 | 12 | 24300 | oar3_OAR19_48389751 | oar3_OAR19_48414050 |
| 19 | 48407386 | 48422940 | 11 | 15555 | oar3_OAR19_48407386 | oar3_OAR19_48422940 |
| 19 | 48469015 | 48489808 | 9 | 20794 | oar3_OAR19_48469015 | oar3_OAR19_48489808 |
| 19 | 48469015 | 48489808 | 9 | 20794 | oar3_OAR19_48469015 | oar3_OAR19_48489808 |
| 19 | 48469777 | 48573976 | 34 | 104200 | oar3_OAR19_48469777 | oar3_OAR19_48573976 |
| 19 | 48469777 | 48489808 | 8 | 20032 | oar3_OAR19_48469777 | oar3_OAR19_48489808 |
| 19 | 48475705 | 48481953 | 6 | 6249 | oar3_OAR19_48475705 | s09766.1 |
| 19 | 48479795 | 48481953 | 3 | 2159 | oar3_OAR19_48479795 | s09766.1 |
| 19 | 48503076 | 48567079 | 19 | 64004 | oar3_OAR19_48503076 | oar3_OAR19_48567079 |
| 19 | 48503076 | 48567079 | 19 | 64004 | oar3_OAR19_48503076 | oar3_OAR19_48567079 |
| 19 | 48798265 | 48812354 | 6 | 14090 | oar3_OAR19_48798265 | oar3_OAR19_48812354 |
| 19 | 48798265 | 48812354 | 6 | 14090 | oar3_OAR19_48798265 | oar3_OAR19_48812354 |
| 19 | 48798265 | 48812354 | 6 | 14090 | oar3_OAR19_48798265 | oar3_OAR19_48812354 |
| 19 | 48798265 | 48812354 | 6 | 14090 | oar3_OAR19_48798265 | oar3_OAR19_48812354 |
| 19 | 48798265 | 48812354 | 6 | 14090 | oar3_OAR19_48798265 | oar3_OAR19_48812354 |
| 19 | 48798265 | 48812354 | 6 | 14090 | oar3_OAR19_48798265 | oar3_OAR19_48812354 |
| 19 | 48798265 | 48812354 | 6 | 14090 | oar3_OAR19_48798265 | oar3_OAR19_48812354 |
| 19 | 48810874 | 48812354 | 3 | 1481 | OAR19_51317745.1 | oar3_OAR19_48812354 |
| 19 | 48810874 | 48812354 | 3 | 1481 | OAR19_51317745.1 | oar3_OAR19_48812354 |
| 19 | 48810874 | 48812354 | 3 | 1481 | OAR19_51317745.1 | oar3_OAR19_48812354 |
| 19 | 48810874 | 48812354 | 3 | 1481 | OAR19_51317745.1 | oar3_OAR19_48812354 |
| 19 | 48896365 | 48906539 | 6 | 10175 | oar3_OAR19_48896365 | oar3_OAR19_48906539 |
| 19 | 48896365 | 48902677 | 5 | 6313 | oar3_OAR19_48896365 | oar3_OAR19_48902677 |
| 19 | 48901438 | 48906539 | 4 | 5102 | oar3_OAR19_48901438 | oar3_OAR19_48906539 |
| 19 | 48901438 | 48931540 | 14 | 30103 | oar3_OAR19_48901438 | oar3_OAR19_48931540 |
| 19 | 48921822 | 48931540 | 6 | 9719 | oar3_OAR19_48921822 | oar3_OAR19_48931540 |
| 19 | 49694351 | 49796746 | 24 | 102396 | oar3_OAR19_49694351 | oar3_OAR19_49796746 |
| 19 | 49694351 | 49796746 | 24 | 102396 | oar3_OAR19_49694351 | oar3_OAR19_49796746 |
| 19 | 49706637 | 49796746 | 21 | 90110 | OAR19_52300573.1 | oar3_OAR19_49796746 |
| 19 | 49742993 | 49796746 | 13 | 53754 | oar3_OAR19_49742993 | oar3_OAR19_49796746 |
| 19 | 49770703 | 49796746 | 7 | 26044 | oar3_OAR19_49770703 | oar3_OAR19_49796746 |
| 19 | 49896600 | 49918951 | 6 | 22352 | oar3_OAR19_49896600 | oar3_OAR19_49918951 |
| 19 | 49954801 | 49990348 | 8 | 35548 | oar3_OAR19_49954801 | oar3_OAR19_49990348 |
| 19 | 49970286 | 49990348 | 6 | 20063 | oar3_OAR19_49970286 | oar3_OAR19_49990348 |
| 19 | 49970286 | 49990348 | 6 | 20063 | oar3_OAR19_49970286 | oar3_OAR19_49990348 |
| 19 | 49970286 | 49990348 | 6 | 20063 | oar3_OAR19_49970286 | oar3_OAR19_49990348 |
| 19 | 49976160 | 49990348 | 5 | 14189 | oar3_OAR19_49976160 | oar3_OAR19_49990348 |
| 19 | 50297805 | 50520625 | 49 | 222821 | oar3_OAR19_50297805 | oar3_OAR19_50520625 |
| 19 | 50297805 | 50504429 | 46 | 206625 | oar3_OAR19_50297805 | oar3_OAR19_50504429 |
| 19 | 50364845 | 50385732 | 9 | 20888 | oar3_OAR19_50364845 | oar3_OAR19_50385732 |
| 19 | 50364845 | 50386531 | 10 | 21687 | oar3_OAR19_50364845 | oar3_OAR19_50386531 |
| 19 | 50364845 | 50393546 | 13 | 28702 | oar3_OAR19_50364845 | oar3_OAR19_50393546 |
| 19 | 50367895 | 50393546 | 12 | 25652 | oar3_OAR19_50367895 | oar3_OAR19_50393546 |
| 19 | 50378578 | 50393546 | 11 | 14969 | s46131.1 | oar3_OAR19_50393546 |
| 19 | 50378578 | 50388106 | 9 | 9529 | s46131.1 | oar3_OAR19_50388106 |
| 19 | 50378578 | 50385732 | 7 | 7155 | s46131.1 | oar3_OAR19_50385732 |
| 19 | 50378578 | 50389381 | 10 | 10804 | s46131.1 | oar3_OAR19_50389381 |
| 19 | 50450129 | 50520625 | 20 | 70497 | oar3_OAR19_50450129 | oar3_OAR19_50520625 |
| 19 | 50450129 | 50520625 | 20 | 70497 | oar3_OAR19_50450129 | oar3_OAR19_50520625 |
| 19 | 50450129 | 50504429 | 17 | 54301 | oar3_OAR19_50450129 | oar3_OAR19_50504429 |
| 19 | 50450129 | 50520625 | 20 | 70497 | oar3_OAR19_50450129 | oar3_OAR19_50520625 |
| 19 | 50481082 | 50488756 | 6 | 7675 | oar3_OAR19_50481082 | oar3_OAR19_50488756 |
| 19 | 50481082 | 50520625 | 12 | 39544 | oar3_OAR19_50481082 | oar3_OAR19_50520625 |
| 19 | 50487803 | 50504429 | 7 | 16627 | oar3_OAR19_50487803 | oar3_OAR19_50504429 |
| 19 | 50792800 | 50865627 | 20 | 72828 | oar3_OAR19_50792800 | oar3_OAR19_50865627 |
| 19 | 50792800 | 50865627 | 20 | 72828 | oar3_OAR19_50792800 | oar3_OAR19_50865627 |
| 19 | 50792800 | 50849292 | 17 | 56493 | oar3_OAR19_50792800 | oar3_OAR19_50849292 |
| 19 | 50820652 | 50865627 | 11 | 44976 | oar3_OAR19_50820652 | oar3_OAR19_50865627 |
| 19 | 50951731 | 50982456 | 9 | 30726 | oar3_OAR19_50951731 | oar3_OAR19_50982456 |
| 19 | 51177448 | 51198280 | 10 | 20833 | oar3_OAR19_51177448 | oar3_OAR19_51198280 |
| 19 | 51240130 | 51259023 | 8 | 18894 | oar3_OAR19_51240130 | oar3_OAR19_51259023 |
| 19 | 51240130 | 51259023 | 8 | 18894 | oar3_OAR19_51240130 | oar3_OAR19_51259023 |
| 19 | 51240130 | 51259023 | 8 | 18894 | oar3_OAR19_51240130 | oar3_OAR19_51259023 |
| 19 | 51245397 | 51259023 | 6 | 13627 | oar3_OAR19_51245397 | oar3_OAR19_51259023 |
| 19 | 51245397 | 51398824 | 41 | 153428 | oar3_OAR19_51245397 | oar3_OAR19_51398824 |
| 19 | 51370010 | 51398824 | 8 | 28815 | oar3_OAR19_51370010 | oar3_OAR19_51398824 |
| 19 | 51370010 | 51398824 | 8 | 28815 | oar3_OAR19_51370010 | oar3_OAR19_51398824 |
| 19 | 51370010 | 51398824 | 8 | 28815 | oar3_OAR19_51370010 | oar3_OAR19_51398824 |
| 19 | 51370010 | 51398824 | 8 | 28815 | oar3_OAR19_51370010 | oar3_OAR19_51398824 |
| 19 | 51370010 | 51396572 | 7 | 26563 | oar3_OAR19_51370010 | oar3_OAR19_51396572 |
| 19 | 52140146 | 52188517 | 14 | 48372 | oar3_OAR19_52140146 | oar3_OAR19_52188517 |
| 19 | 52140146 | 52199852 | 16 | 59707 | oar3_OAR19_52140146 | oar3_OAR19_52199852 |
| 19 | 52140146 | 52188517 | 14 | 48372 | oar3_OAR19_52140146 | oar3_OAR19_52188517 |
| 19 | 52151885 | 52199852 | 14 | 47968 | oar3_OAR19_52151885 | oar3_OAR19_52199852 |
| 19 | 52179868 | 52188517 | 6 | 8650 | oar3_OAR19_52179868 | oar3_OAR19_52188517 |
| 19 | 52463044 | 52577036 | 24 | 113993 | oar3_OAR19_52463044 | oar3_OAR19_52577036 |
| 19 | 52474546 | 52577036 | 22 | 102491 | oar3_OAR19_52474546 | oar3_OAR19_52577036 |
| 19 | 52493095 | 52580066 | 18 | 86972 | oar3_OAR19_52493095 | oar3_OAR19_52580066 |
| 19 | 52493095 | 52577036 | 17 | 83942 | oar3_OAR19_52493095 | oar3_OAR19_52577036 |
| 19 | 52532591 | 52564527 | 10 | 31937 | oar3_OAR19_52532591 | oar3_OAR19_52564527 |
| 19 | 52532591 | 52580066 | 13 | 47476 | oar3_OAR19_52532591 | oar3_OAR19_52580066 |
| 19 | 52704734 | 52739754 | 19 | 35021 | oar3_OAR19_52704734 | oar3_OAR19_52739754 |
| 19 | 53263769 | 53339049 | 22 | 75281 | oar3_OAR19_53263769 | s52871.1 |
| 19 | 53979381 | 54044928 | 16 | 65548 | oar3_OAR19_53979381 | oar3_OAR19_54044928 |
| 19 | 54027443 | 54044928 | 8 | 17486 | oar3_OAR19_54027443 | oar3_OAR19_54044928 |
| 19 | 54027443 | 54044928 | 8 | 17486 | oar3_OAR19_54027443 | oar3_OAR19_54044928 |
| 19 | 54264944 | 54281976 | 9 | 17033 | oar3_OAR19_54264944 | OAR19_57487708.1 |
| 19 | 54368887 | 54408551 | 15 | 39665 | oar3_OAR19_54368887 | OAR19_57613919.1 |
| 19 | 54389612 | 54433864 | 14 | 44253 | oar3_OAR19_54389612 | oar3_OAR19_54433864 |
| 19 | 54400970 | 54454621 | 17 | 53652 | oar3_OAR19_54400970 | oar3_OAR19_54454621 |
| 19 | 54408551 | 54433864 | 6 | 25314 | OAR19_57613919.1 | oar3_OAR19_54433864 |
| 19 | 54420036 | 54454621 | 12 | 34586 | oar3_OAR19_54420036 | oar3_OAR19_54454621 |
| 19 | 54730970 | 54753839 | 8 | 22870 | oar3_OAR19_54730970 | oar3_OAR19_54753839 |
| 19 | 54730970 | 54753839 | 8 | 22870 | oar3_OAR19_54730970 | oar3_OAR19_54753839 |
| 19 | 55424935 | 55475359 | 9 | 50425 | oar3_OAR19_55424935 | oar3_OAR19_55475359 |
| 19 | 55424935 | 55475359 | 9 | 50425 | oar3_OAR19_55424935 | oar3_OAR19_55475359 |
| 19 | 55457899 | 55472003 | 3 | 14105 | oar3_OAR19_55457899 | oar3_OAR19_55472003 |
| 19 | 55968471 | 56053250 | 15 | 84780 | oar3_OAR19_55968471 | oar3_OAR19_56053250 |
| 19 | 55987367 | 56001079 | 5 | 13713 | oar3_OAR19_55987367 | oar3_OAR19_56001079 |
| 19 | 55987367 | 56108217 | 21 | 120851 | oar3_OAR19_55987367 | oar3_OAR19_56108217 |
| 19 | 55996864 | 56077475 | 12 | 80612 | oar3_OAR19_55996864 | oar3_OAR19_56077475 |
| 19 | 56016927 | 56077475 | 10 | 60549 | oar3_OAR19_56016927 | oar3_OAR19_56077475 |
| 19 | 56183921 | 56219539 | 13 | 35619 | oar3_OAR19_56183921 | oar3_OAR19_56219539 |
| 19 | 56398634 | 56432577 | 8 | 33944 | oar3_OAR19_56398634 | oar3_OAR19_56432577 |
| 19 | 57748802 | 57763854 | 7 | 15053 | oar3_OAR19_57748802 | oar3_OAR19_57763854 |
| 19 | 57785616 | 57833038 | 8 | 47423 | oar3_OAR19_57785616 | oar3_OAR19_57833038 |
| 19 | 57887581 | 57902166 | 5 | 14586 | oar3_OAR19_57887581 | oar3_OAR19_57902166 |
| 19 | 57895052 | 57927564 | 13 | 32513 | oar3_OAR19_57895052 | oar3_OAR19_57927564 |
| 19 | 57895052 | 57907622 | 6 | 12571 | oar3_OAR19_57895052 | oar3_OAR19_57907622 |
| 19 | 58017880 | 58045842 | 7 | 27963 | oar3_OAR19_58017880 | oar3_OAR19_58045842 |
| 19 | 58018388 | 58082617 | 13 | 64230 | oar3_OAR19_58018388 | oar3_OAR19_58082617 |
| 19 | 58142731 | 58152168 | 3 | 9438 | oar3_OAR19_58142731 | oar3_OAR19_58152168 |
| 19 | 58181019 | 58198367 | 8 | 17349 | oar3_OAR19_58181019 | oar3_OAR19_58198367 |
| 19 | 58192971 | 58264637 | 13 | 71667 | oar3_OAR19_58192971 | oar3_OAR19_58264637 |
| 19 | 58192971 | 58279723 | 15 | 86753 | oar3_OAR19_58192971 | oar3_OAR19_58279723 |
| 19 | 58192971 | 58279723 | 15 | 86753 | oar3_OAR19_58192971 | oar3_OAR19_58279723 |
| 19 | 58373894 | 58417228 | 18 | 43335 | oar3_OAR19_58373894 | oar3_OAR19_58417228 |
| 19 | 58373894 | 58417228 | 18 | 43335 | oar3_OAR19_58373894 | oar3_OAR19_58417228 |
| 19 | 58377868 | 58490329 | 29 | 112462 | oar3_OAR19_58377868 | oar3_OAR19_58490329 |
| 19 | 58377868 | 58437828 | 20 | 59961 | oar3_OAR19_58377868 | oar3_OAR19_58437828 |
| 19 | 58381431 | 58416464 | 14 | 35034 | oar3_OAR19_58381431 | oar3_OAR19_58416464 |
| 19 | 58381431 | 58393226 | 6 | 11796 | oar3_OAR19_58381431 | oar3_OAR19_58393226 |
| 19 | 58401973 | 58406046 | 5 | 4074 | oar3_OAR19_58401973 | oar3_OAR19_58406046 |
| 19 | 58401973 | 58433878 | 11 | 31906 | oar3_OAR19_58401973 | oar3_OAR19_58433878 |
| 19 | 58401973 | 58406046 | 5 | 4074 | oar3_OAR19_58401973 | oar3_OAR19_58406046 |
| 19 | 58401973 | 58416464 | 8 | 14492 | oar3_OAR19_58401973 | oar3_OAR19_58416464 |
| 19 | 58401973 | 58433878 | 11 | 31906 | oar3_OAR19_58401973 | oar3_OAR19_58433878 |
| 19 | 58401973 | 58406046 | 5 | 4074 | oar3_OAR19_58401973 | oar3_OAR19_58406046 |
| 19 | 58490190 | 58501364 | 4 | 11175 | oar3_OAR19_58490190 | s37983.1 |
| 19 | 58490190 | 58623411 | 27 | 133222 | oar3_OAR19_58490190 | oar3_OAR19_58623411 |
| 19 | 58530011 | 58567548 | 8 | 37538 | s35547.1 | oar3_OAR19_58567548 |
| 19 | 58530011 | 58572313 | 9 | 42303 | s35547.1 | oar3_OAR19_58572313 |
| 19 | 58556710 | 58586549 | 7 | 29840 | oar3_OAR19_58556710 | oar3_OAR19_58586549 |
| 19 | 58586549 | 58616952 | 6 | 30404 | oar3_OAR19_58586549 | oar3_OAR19_58616952 |
| 19 | 58639380 | 58838289 | 44 | 198910 | oar3_OAR19_58639380 | oar3_OAR19_58838289 |
| 19 | 58644590 | 58761160 | 18 | 116571 | oar3_OAR19_58644590 | oar3_OAR19_58761160 |
| 19 | 58644590 | 58694498 | 7 | 49909 | oar3_OAR19_58644590 | oar3_OAR19_58694498 |
| 19 | 58737796 | 58770870 | 10 | 33075 | oar3_OAR19_58737796 | oar3_OAR19_58770870 |
| 19 | 58737796 | 58874041 | 40 | 136246 | oar3_OAR19_58737796 | oar3_OAR19_58874041 |
| 19 | 58786121 | 58854038 | 21 | 67918 | oar3_OAR19_58786121 | oar3_OAR19_58854038 |
| 19 | 58790212 | 58838289 | 17 | 48078 | oar3_OAR19_58790212 | oar3_OAR19_58838289 |
| 19 | 58819205 | 58826850 | 6 | 7646 | oar3_OAR19_58819205 | oar3_OAR19_58826850 |
| 19 | 58926990 | 58976167 | 19 | 49178 | oar3_OAR19_58926990 | oar3_OAR19_58976167 |
| 19 | 58926990 | 59124733 | 51 | 197744 | oar3_OAR19_58926990 | oar3_OAR19_59124733 |
| 19 | 58926990 | 58976167 | 19 | 49178 | oar3_OAR19_58926990 | oar3_OAR19_58976167 |
| 19 | 58926990 | 59003474 | 25 | 76485 | oar3_OAR19_58926990 | oar3_OAR19_59003474 |
| 19 | 58926990 | 59037636 | 35 | 110647 | oar3_OAR19_58926990 | oar3_OAR19_59037636 |
| 19 | 58926990 | 58976167 | 19 | 49178 | oar3_OAR19_58926990 | oar3_OAR19_58976167 |
| 19 | 58926990 | 59037636 | 35 | 110647 | oar3_OAR19_58926990 | oar3_OAR19_59037636 |
| 19 | 58937997 | 58976167 | 17 | 38171 | oar3_OAR19_58937997 | oar3_OAR19_58976167 |
| 19 | 58961651 | 58976167 | 7 | 14517 | oar3_OAR19_58961651 | oar3_OAR19_58976167 |
| 19 | 59076351 | 59278405 | 31 | 202055 | oar3_OAR19_59076351 | oar3_OAR19_59278405 |
| 19 | 59076351 | 59104852 | 8 | 28502 | oar3_OAR19_59076351 | oar3_OAR19_59104852 |
| 19 | 59076351 | 59149792 | 13 | 73442 | oar3_OAR19_59076351 | oar3_OAR19_59149792 |
| 19 | 59076351 | 59378510 | 51 | 302160 | oar3_OAR19_59076351 | oar3_OAR19_59378510 |
| 19 | 59104852 | 59196968 | 9 | 92117 | oar3_OAR19_59104852 | oar3_OAR19_59196968 |
| 19 | 59145911 | 59196968 | 5 | 51058 | oar3_OAR19_59145911 | oar3_OAR19_59196968 |
| 19 | 59224781 | 59273799 | 11 | 49019 | oar3_OAR19_59224781 | oar3_OAR19_59273799 |
| 19 | 59224781 | 59297452 | 17 | 72672 | oar3_OAR19_59224781 | oar3_OAR19_59297452 |
| 19 | 59234751 | 59273799 | 10 | 39049 | oar3_OAR19_59234751 | oar3_OAR19_59273799 |
| 19 | 59247225 | 59282571 | 11 | 35347 | oar3_OAR19_59247225 | s04973.1 |
| 19 | 59252592 | 59343751 | 21 | 91160 | oar3_OAR19_59252592 | oar3_OAR19_59343751 |
| 19 | 59267010 | 59273799 | 4 | 6790 | oar3_OAR19_59267010 | oar3_OAR19_59273799 |
| 19 | 59297452 | 59343751 | 11 | 46300 | oar3_OAR19_59297452 | oar3_OAR19_59343751 |
| 19 | 59329196 | 59343751 | 6 | 14556 | oar3_OAR19_59329196 | oar3_OAR19_59343751 |
| 19 | 59411347 | 59448962 | 9 | 37616 | oar3_OAR19_59411347 | s06147.1 |
| 19 | 59506592 | 59573609 | 11 | 67018 | oar3_OAR19_59506592 | oar3_OAR19_59573609 |
| 19 | 59556779 | 59565465 | 4 | 8687 | oar3_OAR19_59556779 | oar3_OAR19_59565465 |
| 19 | 59556779 | 59621423 | 13 | 64645 | oar3_OAR19_59556779 | oar3_OAR19_59621423 |
| 19 | 59582675 | 59621423 | 8 | 38749 | oar3_OAR19_59582675 | oar3_OAR19_59621423 |
| 19 | 59601816 | 59901868 | 30 | 300053 | oar3_OAR19_59601816 | oar3_OAR19_59901868 |
| 19 | 59765291 | 59788902 | 5 | 23612 | oar3_OAR19_59765291 | oar3_OAR19_59788902 |
| 19 | 59765291 | 59788902 | 5 | 23612 | oar3_OAR19_59765291 | oar3_OAR19_59788902 |
| 19 | 59765291 | 59812546 | 10 | 47256 | oar3_OAR19_59765291 | oar3_OAR19_59812546 |
| 19 | 59805219 | 59846922 | 8 | 41704 | oar3_OAR19_59805219 | oar3_OAR19_59846922 |
| 19 | 59805219 | 59846922 | 8 | 41704 | oar3_OAR19_59805219 | oar3_OAR19_59846922 |
| 19 | 59805219 | 59846922 | 8 | 41704 | oar3_OAR19_59805219 | oar3_OAR19_59846922 |
| 19 | 59810486 | 59846922 | 7 | 36437 | oar3_OAR19_59810486 | oar3_OAR19_59846922 |
| 19 | 59936480 | 59964874 | 8 | 28395 | oar3_OAR19_59936480 | s32969.1 |
| 19 | 59961840 | 60009362 | 16 | 47523 | oar3_OAR19_59961840 | oar3_OAR19_60009362 |
| 19 | 60006024 | 60007961 | 3 | 1938 | oar3_OAR19_60006024 | oar3_OAR19_60007961 |
| 19 | 60050531 | 60071894 | 6 | 21364 | oar3_OAR19_60050531 | oar3_OAR19_60071894 |
| 19 | 60050531 | 60071894 | 6 | 21364 | oar3_OAR19_60050531 | oar3_OAR19_60071894 |
| 19 | 60070916 | 60071894 | 3 | 979 | oar3_OAR19_60070916 | oar3_OAR19_60071894 |
| 19 | 60070916 | 60109510 | 11 | 38595 | oar3_OAR19_60070916 | oar3_OAR19_60109510 |
| 19 | 60136466 | 60438098 | 51 | 301633 | oar3_OAR19_60136466 | oar3_OAR19_60438098 |
| 19 | 60136466 | 60184885 | 11 | 48420 | oar3_OAR19_60136466 | oar3_OAR19_60184885 |
| 19 | 60136466 | 60184885 | 11 | 48420 | oar3_OAR19_60136466 | oar3_OAR19_60184885 |
| 19 | 60144100 | 60231531 | 13 | 87432 | oar3_OAR19_60144100 | oar3_OAR19_60231531 |
| 19 | 60144100 | 60280045 | 24 | 135946 | oar3_OAR19_60144100 | oar3_OAR19_60280045 |
| 19 | 60144100 | 60220276 | 11 | 76177 | oar3_OAR19_60144100 | oar3_OAR19_60220276 |
| 19 | 60153795 | 60220276 | 10 | 66482 | oar3_OAR19_60153795 | oar3_OAR19_60220276 |
| 19 | 60161062 | 60197621 | 5 | 36560 | oar3_OAR19_60161062 | oar3_OAR19_60197621 |
| 19 | 60161062 | 60220276 | 8 | 59215 | oar3_OAR19_60161062 | oar3_OAR19_60220276 |
| 19 | 60197621 | 60220276 | 4 | 22656 | oar3_OAR19_60197621 | oar3_OAR19_60220276 |
| 19 | 60197621 | 60220276 | 4 | 22656 | oar3_OAR19_60197621 | oar3_OAR19_60220276 |
| 19 | 60210379 | 60229021 | 4 | 18643 | oar3_OAR19_60210379 | oar3_OAR19_60229021 |
| 19 | 60274685 | 60296207 | 8 | 21523 | oar3_OAR19_60274685 | oar3_OAR19_60296207 |
| 19 | 60359046 | 60438098 | 7 | 79053 | oar3_OAR19_60359046 | oar3_OAR19_60438098 |
| 20 | 987964 | 1010210 | 5 | 22247 | oar3_OAR20_987964 | oar3_OAR20_1010210 |
| 20 | 987964 | 1010210 | 5 | 22247 | oar3_OAR20_987964 | oar3_OAR20_1010210 |
| 20 | 1792320 | 1803350 | 4 | 11031 | oar3_OAR20_1792320 | oar3_OAR20_1803350 |
| 20 | 1792320 | 1814000 | 6 | 21681 | oar3_OAR20_1792320 | oar3_OAR20_1814000 |
| 20 | 1792320 | 1803350 | 4 | 11031 | oar3_OAR20_1792320 | oar3_OAR20_1803350 |
| 20 | 3373093 | 3398654 | 8 | 25562 | oar3_OAR20_3373093 | oar3_OAR20_3398654 |
| 20 | 3373093 | 3398654 | 8 | 25562 | oar3_OAR20_3373093 | oar3_OAR20_3398654 |
| 20 | 7127296 | 7134061 | 6 | 6766 | oar3_OAR20_7127296 | oar3_OAR20_7134061 |
| 20 | 7127296 | 7155553 | 11 | 28258 | oar3_OAR20_7127296 | oar3_OAR20_7155553 |
| 20 | 7127296 | 7134061 | 6 | 6766 | oar3_OAR20_7127296 | oar3_OAR20_7134061 |
| 20 | 7127296 | 7138669 | 8 | 11374 | oar3_OAR20_7127296 | oar3_OAR20_7138669 |
| 20 | 7127296 | 7141136 | 9 | 13841 | oar3_OAR20_7127296 | oar3_OAR20_7141136 |
| 20 | 7835350 | 7903573 | 22 | 68224 | oar3_OAR20_7835350 | oar3_OAR20_7903573 |
| 20 | 7859503 | 7903573 | 15 | 44071 | oar3_OAR20_7859503 | oar3_OAR20_7903573 |
| 20 | 7879194 | 7926523 | 10 | 47330 | oar3_OAR20_7879194 | oar3_OAR20_7926523 |
| 20 | 7879194 | 7926523 | 10 | 47330 | oar3_OAR20_7879194 | oar3_OAR20_7926523 |
| 20 | 7961681 | 8146895 | 36 | 185215 | OAR20_8148291.1 | oar3_OAR20_8146895 |
| 20 | 7961681 | 7977325 | 6 | 15645 | OAR20_8148291.1 | oar3_OAR20_7977325 |
| 20 | 8247650 | 8272487 | 7 | 24838 | oar3_OAR20_8247650 | oar3_OAR20_8272487 |
| 20 | 8247650 | 8272487 | 7 | 24838 | oar3_OAR20_8247650 | oar3_OAR20_8272487 |
| 20 | 8247650 | 8272487 | 7 | 24838 | oar3_OAR20_8247650 | oar3_OAR20_8272487 |
| 20 | 8247650 | 8272487 | 7 | 24838 | oar3_OAR20_8247650 | oar3_OAR20_8272487 |
| 20 | 8321229 | 8392855 | 15 | 71627 | oar3_OAR20_8321229 | oar3_OAR20_8392855 |
| 20 | 8344217 | 8446449 | 18 | 102233 | oar3_OAR20_8344217 | oar3_OAR20_8446449 |
| 20 | 8355727 | 8446449 | 15 | 90723 | oar3_OAR20_8355727 | oar3_OAR20_8446449 |
| 20 | 9141221 | 9168724 | 10 | 27504 | oar3_OAR20_9141221 | oar3_OAR20_9168724 |
| 20 | 9143131 | 9187744 | 12 | 44614 | oar3_OAR20_9143131 | oar3_OAR20_9187744 |
| 20 | 9143131 | 9187744 | 12 | 44614 | oar3_OAR20_9143131 | oar3_OAR20_9187744 |
| 20 | 9147904 | 9162259 | 6 | 14356 | oar3_OAR20_9147904 | s65814.1 |
| 20 | 9147904 | 9162259 | 6 | 14356 | oar3_OAR20_9147904 | s65814.1 |
| 20 | 9485067 | 9515974 | 10 | 30908 | oar3_OAR20_9485067 | oar3_OAR20_9515974 |
| 20 | 10713048 | 10748663 | 11 | 35616 | oar3_OAR20_10713048 | oar3_OAR20_10748663 |
| 20 | 10713048 | 10748663 | 11 | 35616 | oar3_OAR20_10713048 | oar3_OAR20_10748663 |
| 20 | 10717264 | 10722782 | 5 | 5519 | s22145.1 | oar3_OAR20_10722782 |
| 20 | 10973154 | 11080463 | 27 | 107310 | oar3_OAR20_10973154 | oar3_OAR20_11080463 |
| 20 | 14013746 | 14049020 | 9 | 35275 | oar3_OAR20_14013746 | oar3_OAR20_14049020 |
| 20 | 15339691 | 15566192 | 50 | 226502 | oar3_OAR20_15339691 | oar3_OAR20_15566192 |
| 20 | 15451966 | 15502770 | 11 | 50805 | oar3_OAR20_15451966 | oar3_OAR20_15502770 |
| 20 | 16856201 | 16946079 | 18 | 89879 | oar3_OAR20_16856201 | OAR20_17775022.1 |
| 20 | 16856201 | 16946079 | 18 | 89879 | oar3_OAR20_16856201 | OAR20_17775022.1 |
| 20 | 17076233 | 17138992 | 15 | 62760 | oar3_OAR20_17076233 | oar3_OAR20_17138992 |
| 20 | 17088108 | 17133442 | 12 | 45335 | oar3_OAR20_17088108 | oar3_OAR20_17133442 |
| 20 | 17088108 | 17091095 | 6 | 2988 | oar3_OAR20_17088108 | oar3_OAR20_17091095 |
| 20 | 17088108 | 17138992 | 14 | 50885 | oar3_OAR20_17088108 | oar3_OAR20_17138992 |
| 20 | 17395668 | 17588299 | 40 | 192632 | oar3_OAR20_17395668 | oar3_OAR20_17588299 |
| 20 | 17477494 | 17489382 | 5 | 11889 | oar3_OAR20_17477494 | oar3_OAR20_17489382 |
| 20 | 17816645 | 17886764 | 19 | 70120 | oar3_OAR20_17816645 | oar3_OAR20_17886764 |
| 20 | 17816645 | 17886764 | 19 | 70120 | oar3_OAR20_17816645 | oar3_OAR20_17886764 |
| 20 | 17870173 | 17886764 | 10 | 16592 | oar3_OAR20_17870173 | oar3_OAR20_17886764 |
| 20 | 17870173 | 17885738 | 9 | 15566 | oar3_OAR20_17870173 | oar3_OAR20_17885738 |
| 20 | 17878899 | 17886764 | 9 | 7866 | oar3_OAR20_17878899 | oar3_OAR20_17886764 |
| 20 | 17878899 | 17886764 | 9 | 7866 | oar3_OAR20_17878899 | oar3_OAR20_17886764 |
| 20 | 20094613 | 20099512 | 9 | 4900 | oar3_OAR20_20094613 | oar3_OAR20_20099512 |
| 20 | 20094613 | 20099512 | 9 | 4900 | oar3_OAR20_20094613 | oar3_OAR20_20099512 |
| 20 | 25255531 | 25257947 | 5 | 2417 | oar3_OAR20_25255531 | oar3_OAR20_25257947_dup |
| 20 | 25255531 | 25257947 | 5 | 2417 | oar3_OAR20_25255531 | oar3_OAR20_25257947_dup |
| 20 | 25255531 | 25257947 | 5 | 2417 | oar3_OAR20_25255531 | oar3_OAR20_25257947_dup |
| 20 | 25257945 | 25257947 | 4 | 3 | oar3_OAR20_25257945 | oar3_OAR20_25257947_dup |
| 20 | 25319392 | 25346061 | 6 | 26670 | oar3_OAR20_25319392 | oar3_OAR20_25346061 |
| 20 | 25669233 | 25685291 | 4 | 16059 | oar3_OAR20_25669233 | oar3_OAR20_25685291 |
| 20 | 25890124 | 25986430 | 16 | 96307 | oar3_OAR20_25890124 | oar3_OAR20_25986430 |
| 20 | 25890124 | 25908002 | 3 | 17879 | oar3_OAR20_25890124 | oar3_OAR20_25908002 |
| 20 | 26252684 | 26275625 | 8 | 22942 | oar3_OAR20_26252684 | oar3_OAR20_26275625 |
| 20 | 26415431 | 26451444 | 11 | 36014 | oar3_OAR20_26415431 | oar3_OAR20_26451444 |
| 20 | 26417540 | 26455214 | 11 | 37675 | oar3_OAR20_26417540 | oar3_OAR20_26455214 |
| 20 | 26451444 | 26552532 | 25 | 101089 | oar3_OAR20_26451444 | oar3_OAR20_26552532_dup |
| 20 | 26535105 | 26552532 | 15 | 17428 | oar3_OAR20_26535105 | oar3_OAR20_26552532_dup |
| 20 | 26535105 | 26552532 | 15 | 17428 | oar3_OAR20_26535105 | oar3_OAR20_26552532_dup |
| 20 | 26535105 | 26552532 | 15 | 17428 | oar3_OAR20_26535105 | oar3_OAR20_26552532_dup |
| 20 | 26551043 | 26552532 | 6 | 1490 | oar3_OAR20_26551043_dup | oar3_OAR20_26552532_dup |
| 20 | 26551043 | 26552532 | 6 | 1490 | oar3_OAR20_26551043_dup | oar3_OAR20_26552532_dup |
| 20 | 27024130 | 27037840 | 3 | 13711 | oar3_OAR20_27024130 | oar3_OAR20_27037840 |
| 20 | 27613000 | 27715553 | 9 | 102554 | oar3_OAR20_27613000 | oar3_OAR20_27715553 |
| 20 | 27628331 | 27762409 | 13 | 134079 | oar3_OAR20_27628331 | oar3_OAR20_27762409 |
| 20 | 27673907 | 27715553 | 6 | 41647 | oar3_OAR20_27673907 | oar3_OAR20_27715553 |
| 20 | 28094525 | 28100294 | 3 | 5770 | oar3_OAR20_28094525 | oar3_OAR20_28100294 |
| 20 | 28405966 | 28495064 | 10 | 89099 | oar3_OAR20_28405966 | oar3_OAR20_28495064 |
| 20 | 28405966 | 28523059 | 14 | 117094 | oar3_OAR20_28405966 | oar3_OAR20_28523059 |
| 20 | 28447039 | 28495064 | 9 | 48026 | oar3_OAR20_28447039 | oar3_OAR20_28495064 |
| 20 | 29338237 | 29345066 | 5 | 6830 | oar3_OAR20_29338237 | oar3_OAR20_29345066 |
| 20 | 29617590 | 29631541 | 4 | 13952 | oar3_OAR20_29617590 | oar3_OAR20_29631541 |
| 20 | 29617590 | 29631541 | 4 | 13952 | oar3_OAR20_29617590 | oar3_OAR20_29631541 |
| 20 | 33061919 | 33075804 | 6 | 13886 | oar3_OAR20_33061919 | oar3_OAR20_33075804 |
| 20 | 44596246 | 44636505 | 9 | 40260 | oar3_OAR20_44596246 | oar3_OAR20_44636505 |
| 20 | 47027548 | 47047485 | 6 | 19938 | oar3_OAR20_47027548 | oar3_OAR20_47047485 |
| 20 | 48535576 | 48586120 | 14 | 50545 | oar3_OAR20_48535576 | oar3_OAR20_48586120 |
| 20 | 48848459 | 48853637 | 4 | 5179 | oar3_OAR20_48848459 | oar3_OAR20_48853637 |
| 20 | 48848459 | 48853637 | 4 | 5179 | oar3_OAR20_48848459 | oar3_OAR20_48853637 |
| 20 | 48848459 | 48853637 | 4 | 5179 | oar3_OAR20_48848459 | oar3_OAR20_48853637 |
| 20 | 48876391 | 48884131 | 5 | 7741 | oar3_OAR20_48876391 | oar3_OAR20_48884131 |
| 20 | 48975686 | 48980286 | 4 | 4601 | oar3_OAR20_48975686 | oar3_OAR20_48980286 |
| 20 | 48975686 | 49034586 | 17 | 58901 | oar3_OAR20_48975686 | oar3_OAR20_49034586 |
| 20 | 49342949 | 49449119 | 18 | 106171 | oar3_OAR20_49342949 | oar3_OAR20_49449119 |
| 20 | 49342949 | 49407417 | 10 | 64469 | oar3_OAR20_49342949 | oar3_OAR20_49407417 |
| 20 | 49522132 | 49524087 | 3 | 1956 | oar3_OAR20_49522132 | oar3_OAR20_49524087 |
| 20 | 49699871 | 49722372 | 8 | 22502 | oar3_OAR20_49699871 | oar3_OAR20_49722372 |
| 20 | 49757530 | 49781280 | 12 | 23751 | oar3_OAR20_49757530 | oar3_OAR20_49781280 |
| 20 | 49873721 | 49930884 | 17 | 57164 | oar3_OAR20_49873721 | s61980.1 |
| 20 | 49913946 | 50511160 | 57 | 597215 | oar3_OAR20_49913946 | oar3_OAR20_50511160 |
| 20 | 49976469 | 50016278 | 6 | 39810 | oar3_OAR20_49976469 | s61798.1 |
| 20 | 50008071 | 50021678 | 6 | 13608 | oar3_OAR20_50008071 | oar3_OAR20_50021678 |
| 20 | 50008071 | 50240342 | 25 | 232272 | oar3_OAR20_50008071 | oar3_OAR20_50240342 |
| 20 | 50013976 | 50429755 | 38 | 415780 | oar3_OAR20_50013976 | oar3_OAR20_50429755 |
| 20 | 50013976 | 50206384 | 22 | 192409 | oar3_OAR20_50013976 | oar3_OAR20_50206384 |
| 20 | 50196027 | 50206384 | 3 | 10358 | oar3_OAR20_50196027 | oar3_OAR20_50206384 |
| 20 | 50196027 | 50206384 | 3 | 10358 | oar3_OAR20_50196027 | oar3_OAR20_50206384 |
| 20 | 50363061 | 50649611 | 23 | 286551 | oar3_OAR20_50363061 | oar3_OAR20_50649611 |
| 20 | 50363061 | 50429755 | 6 | 66695 | oar3_OAR20_50363061 | oar3_OAR20_50429755 |
| 20 | 50363061 | 50511160 | 7 | 148100 | oar3_OAR20_50363061 | oar3_OAR20_50511160 |
| 20 | 50587706 | 50604984 | 6 | 17279 | oar3_OAR20_50587706 | oar3_OAR20_50604984 |
| 20 | 50603609 | 50654197 | 15 | 50589 | oar3_OAR20_50603609 | oar3_OAR20_50654197 |
| 21 | 5082627 | 5091314 | 6 | 8688 | oar3_OAR21_5082627 | oar3_OAR21_5091314 |
| 21 | 6608968 | 6622726 | 6 | 13759 | oar3_OAR21_6608968 | oar3_OAR21_6622726 |
| 21 | 17172033 | 17213120 | 13 | 41088 | oar3_OAR21_17172033 | oar3_OAR21_17213120 |
| 21 | 17196608 | 17213120 | 8 | 16513 | oar3_OAR21_17196608 | oar3_OAR21_17213120 |
| 21 | 20397268 | 20423318 | 8 | 26051 | OAR21_22997043.1 | oar3_OAR21_20423318 |
| 21 | 26199619 | 26212808 | 5 | 13190 | oar3_OAR21_26199619 | oar3_OAR21_26212808 |
| 21 | 26830286 | 26837030 | 5 | 6745 | oar3_OAR21_26830286 | oar3_OAR21_26837030 |
| 21 | 31429601 | 31434862 | 4 | 5262 | OAR21_35021714.1 | oar3_OAR21_31434862 |
| 21 | 32316796 | 32371064 | 12 | 54269 | oar3_OAR21_32316796 | oar3_OAR21_32371064 |
| 21 | 32316796 | 32371064 | 12 | 54269 | oar3_OAR21_32316796 | oar3_OAR21_32371064 |
| 21 | 32349127 | 32371064 | 6 | 21938 | oar3_OAR21_32349127 | oar3_OAR21_32371064 |
| 21 | 32349127 | 32371064 | 6 | 21938 | oar3_OAR21_32349127 | oar3_OAR21_32371064 |
| 21 | 36455596 | 36477123 | 8 | 21528 | oar3_OAR21_36455596 | oar3_OAR21_36477123 |
| 21 | 36652772 | 36663416 | 7 | 10645 | s44626.1 | oar3_OAR21_36663416 |
| 21 | 36657509 | 36663416 | 6 | 5908 | oar3_OAR21_36657509 | oar3_OAR21_36663416 |
| 21 | 36657509 | 36681252 | 14 | 23744 | oar3_OAR21_36657509 | oar3_OAR21_36681252 |
| 21 | 36657509 | 36681252 | 14 | 23744 | oar3_OAR21_36657509 | oar3_OAR21_36681252 |
| 21 | 36657509 | 36663416 | 6 | 5908 | oar3_OAR21_36657509 | oar3_OAR21_36663416 |
| 21 | 39197391 | 39206714 | 5 | 9324 | oar3_OAR21_39197391 | oar3_OAR21_39206714 |
| 21 | 39197391 | 39260770 | 17 | 63380 | oar3_OAR21_39197391 | oar3_OAR21_39260770 |
| 21 | 39385496 | 39467264 | 15 | 81769 | oar3_OAR21_39385496 | oar3_OAR21_39467264 |
| 21 | 39385496 | 39405927 | 5 | 20432 | oar3_OAR21_39385496 | oar3_OAR21_39405927 |
| 21 | 39385496 | 39405927 | 5 | 20432 | oar3_OAR21_39385496 | oar3_OAR21_39405927 |
| 21 | 39522127 | 39638962 | 23 | 116836 | oar3_OAR21_39522127 | s33980.1 |
| 21 | 39533129 | 39612502 | 18 | 79374 | oar3_OAR21_39533129 | oar3_OAR21_39612502 |
| 21 | 39547065 | 39643146 | 17 | 96082 | oar3_OAR21_39547065 | oar3_OAR21_39643146 |
| 21 | 39547065 | 39572786 | 6 | 25722 | oar3_OAR21_39547065 | oar3_OAR21_39572786 |
| 21 | 39572786 | 39638962 | 10 | 66177 | oar3_OAR21_39572786 | s33980.1 |
| 21 | 39589560 | 39612502 | 6 | 22943 | oar3_OAR21_39589560 | oar3_OAR21_39612502 |
| 21 | 39589560 | 39638962 | 8 | 49403 | oar3_OAR21_39589560 | s33980.1 |
| 21 | 39777295 | 39814475 | 25 | 37181 | oar3_OAR21_39777295 | oar3_OAR21_39814475 |
| 21 | 39777295 | 39781722 | 6 | 4428 | oar3_OAR21_39777295 | oar3_OAR21_39781722 |
| 21 | 39808986 | 39828971 | 12 | 19986 | oar3_OAR21_39808986 | oar3_OAR21_39828971 |
| 21 | 39808986 | 39828971 | 12 | 19986 | oar3_OAR21_39808986 | oar3_OAR21_39828971 |
| 21 | 39808986 | 39811515 | 6 | 2530 | oar3_OAR21_39808986 | oar3_OAR21_39811515_dup |
| 21 | 39808986 | 39814475 | 7 | 5490 | oar3_OAR21_39808986 | oar3_OAR21_39814475 |
| 21 | 40006970 | 40037109 | 10 | 30140 | oar3_OAR21_40006970 | oar3_OAR21_40037109 |
| 21 | 40011974 | 40037109 | 8 | 25136 | oar3_OAR21_40011974 | oar3_OAR21_40037109 |
| 21 | 40011974 | 40037109 | 8 | 25136 | oar3_OAR21_40011974 | oar3_OAR21_40037109 |
| 21 | 40019018 | 40037109 | 7 | 18092 | oar3_OAR21_40019018 | oar3_OAR21_40037109 |
| 21 | 40333808 | 40417863 | 22 | 84056 | oar3_OAR21_40333808 | oar3_OAR21_40417863 |
| 21 | 40333808 | 40417863 | 22 | 84056 | oar3_OAR21_40333808 | oar3_OAR21_40417863 |
| 21 | 40361066 | 40417863 | 17 | 56798 | oar3_OAR21_40361066 | oar3_OAR21_40417863 |
| 21 | 40408018 | 40427706 | 7 | 19689 | oar3_OAR21_40408018 | oar3_OAR21_40427706 |
| 21 | 41476276 | 41597614 | 32 | 121339 | oar3_OAR21_41476276 | oar3_OAR21_41597614 |
| 21 | 41530055 | 41557926 | 10 | 27872 | oar3_OAR21_41530055 | oar3_OAR21_41557926 |
| 21 | 41543236 | 41569945 | 11 | 26710 | oar3_OAR21_41543236 | oar3_OAR21_41569945 |
| 21 | 41671459 | 41693263 | 8 | 21805 | oar3_OAR21_41671459 | oar3_OAR21_41693263 |
| 21 | 41671459 | 41702405 | 10 | 30947 | oar3_OAR21_41671459 | oar3_OAR21_41702405 |
| 21 | 41671459 | 41717747 | 14 | 46289 | oar3_OAR21_41671459 | s57567.1 |
| 21 | 42090959 | 42179795 | 23 | 88837 | oar3_OAR21_42090959 | oar3_OAR21_42179795 |
| 21 | 42150108 | 42279684 | 28 | 129577 | oar3_OAR21_42150108 | oar3_OAR21_42279684 |
| 21 | 42150108 | 42473948 | 49 | 323841 | oar3_OAR21_42150108 | oar3_OAR21_42473948 |
| 21 | 42150108 | 42179795 | 10 | 29688 | oar3_OAR21_42150108 | oar3_OAR21_42179795 |
| 21 | 42347326 | 42449754 | 12 | 102429 | oar3_OAR21_42347326 | oar3_OAR21_42449754 |
| 21 | 42376353 | 42449754 | 9 | 73402 | oar3_OAR21_42376353 | oar3_OAR21_42449754 |
| 21 | 42393680 | 42442018 | 6 | 48339 | oar3_OAR21_42393680 | OAR21_46936641.1 |
| 21 | 42430826 | 42449754 | 5 | 18929 | oar3_OAR21_42430826 | oar3_OAR21_42449754 |
| 21 | 42571993 | 42584061 | 7 | 12069 | oar3_OAR21_42571993 | oar3_OAR21_42584061 |
| 21 | 42578236 | 42582124 | 5 | 3889 | oar3_OAR21_42578236 | oar3_OAR21_42582124 |
| 21 | 42578236 | 42584061 | 6 | 5826 | oar3_OAR21_42578236 | oar3_OAR21_42584061 |
| 21 | 42578236 | 42584061 | 6 | 5826 | oar3_OAR21_42578236 | oar3_OAR21_42584061 |
| 21 | 43023084 | 43205792 | 29 | 182709 | oar3_OAR21_43023084 | oar3_OAR21_43205792 |
| 21 | 43023084 | 43213527 | 31 | 190444 | oar3_OAR21_43023084 | oar3_OAR21_43213527 |
| 21 | 43023084 | 43130551 | 14 | 107468 | oar3_OAR21_43023084 | oar3_OAR21_43130551 |
| 21 | 43023084 | 43130551 | 14 | 107468 | oar3_OAR21_43023084 | oar3_OAR21_43130551 |
| 21 | 43023084 | 43085610 | 8 | 62527 | oar3_OAR21_43023084 | oar3_OAR21_43085610 |
| 21 | 43023084 | 43130551 | 14 | 107468 | oar3_OAR21_43023084 | oar3_OAR21_43130551 |
| 21 | 43204043 | 43205792 | 6 | 1750 | oar3_OAR21_43204043 | oar3_OAR21_43205792 |
| 21 | 43204043 | 43205792 | 6 | 1750 | oar3_OAR21_43204043 | oar3_OAR21_43205792 |
| 21 | 43204043 | 43205792 | 6 | 1750 | oar3_OAR21_43204043 | oar3_OAR21_43205792 |
| 21 | 43204043 | 43205792 | 6 | 1750 | oar3_OAR21_43204043 | oar3_OAR21_43205792 |
| 21 | 43204043 | 43205792 | 6 | 1750 | oar3_OAR21_43204043 | oar3_OAR21_43205792 |
| 21 | 43365833 | 43399823 | 9 | 33991 | oar3_OAR21_43365833 | oar3_OAR21_43399823 |
| 21 | 43365833 | 43386880 | 8 | 21048 | oar3_OAR21_43365833 | oar3_OAR21_43386880 |
| 21 | 43365833 | 43386880 | 8 | 21048 | oar3_OAR21_43365833 | oar3_OAR21_43386880 |
| 21 | 43365833 | 43386880 | 8 | 21048 | oar3_OAR21_43365833 | oar3_OAR21_43386880 |
| 21 | 43365833 | 43386880 | 8 | 21048 | oar3_OAR21_43365833 | oar3_OAR21_43386880 |
| 21 | 43615509 | 43720543 | 20 | 105035 | oar3_OAR21_43615509 | oar3_OAR21_43720543 |
| 21 | 44152783 | 44180551 | 9 | 27769 | oar3_OAR21_44152783 | oar3_OAR21_44180551 |
| 21 | 44152783 | 44180551 | 9 | 27769 | oar3_OAR21_44152783 | oar3_OAR21_44180551 |
| 21 | 44164306 | 44180551 | 4 | 16246 | oar3_OAR21_44164306 | oar3_OAR21_44180551 |
| 21 | 44512435 | 44881157 | 75 | 368723 | oar3_OAR21_44512435 | oar3_OAR21_44881157 |
| 21 | 44551183 | 44685181 | 23 | 133999 | oar3_OAR21_44551183 | oar3_OAR21_44685181 |
| 21 | 44551183 | 44623988 | 13 | 72806 | oar3_OAR21_44551183 | oar3_OAR21_44623988 |
| 21 | 44551183 | 44685181 | 23 | 133999 | oar3_OAR21_44551183 | oar3_OAR21_44685181 |
| 21 | 44591551 | 44685181 | 18 | 93631 | oar3_OAR21_44591551 | oar3_OAR21_44685181 |
| 21 | 44602856 | 44673251 | 15 | 70396 | oar3_OAR21_44602856 | oar3_OAR21_44673251 |
| 21 | 44602856 | 44623988 | 7 | 21133 | oar3_OAR21_44602856 | oar3_OAR21_44623988 |
| 21 | 44602856 | 44685181 | 17 | 82326 | oar3_OAR21_44602856 | oar3_OAR21_44685181 |
| 21 | 44642044 | 44673251 | 8 | 31208 | oar3_OAR21_44642044 | oar3_OAR21_44673251 |
| 21 | 44744624 | 44881157 | 32 | 136534 | oar3_OAR21_44744624 | oar3_OAR21_44881157 |
| 21 | 44749997 | 44881157 | 28 | 131161 | oar3_OAR21_44749997 | oar3_OAR21_44881157 |
| 21 | 44822434 | 44881157 | 13 | 58724 | oar3_OAR21_44822434 | oar3_OAR21_44881157 |
| 21 | 45328757 | 45406377 | 25 | 77621 | oar3_OAR21_45328757 | oar3_OAR21_45406377 |
| 21 | 45340295 | 45412601 | 23 | 72307 | oar3_OAR21_45340295 | oar3_OAR21_45412601 |
| 21 | 45510724 | 45540787 | 8 | 30064 | oar3_OAR21_45510724 | oar3_OAR21_45540787 |
| 21 | 45636271 | 45890586 | 64 | 254316 | s25105.1 | oar3_OAR21_45890586 |
| 21 | 45658695 | 45755808 | 26 | 97114 | oar3_OAR21_45658695 | oar3_OAR21_45755808 |
| 21 | 45658695 | 45686671 | 9 | 27977 | oar3_OAR21_45658695 | oar3_OAR21_45686671 |
| 21 | 45665021 | 45755808 | 25 | 90788 | oar3_OAR21_45665021 | oar3_OAR21_45755808 |
| 21 | 45665021 | 45755808 | 25 | 90788 | oar3_OAR21_45665021 | oar3_OAR21_45755808 |
| 21 | 45717958 | 45755808 | 12 | 37851 | oar3_OAR21_45717958 | oar3_OAR21_45755808 |
| 21 | 45717958 | 45733273 | 9 | 15316 | oar3_OAR21_45717958 | s37663.1 |
| 21 | 45731327 | 45733273 | 6 | 1947 | oar3_OAR21_45731327 | s37663.1 |
| 21 | 45732643 | 45755808 | 7 | 23166 | oar3_OAR21_45732643 | oar3_OAR21_45755808 |
| 21 | 45732643 | 45755808 | 7 | 23166 | oar3_OAR21_45732643 | oar3_OAR21_45755808 |
| 21 | 45937350 | 45951795 | 6 | 14446 | oar3_OAR21_45937350 | oar3_OAR21_45951795 |
| 21 | 45948751 | 46008067 | 13 | 59317 | oar3_OAR21_45948751 | oar3_OAR21_46008067 |
| 21 | 46179662 | 46396466 | 58 | 216805 | oar3_OAR21_46179662 | oar3_OAR21_46396466 |
| 21 | 46179662 | 46275532 | 26 | 95871 | oar3_OAR21_46179662 | oar3_OAR21_46275532 |
| 21 | 46179662 | 46196348 | 7 | 16687 | oar3_OAR21_46179662 | oar3_OAR21_46196348 |
| 21 | 46179662 | 46196348 | 7 | 16687 | oar3_OAR21_46179662 | oar3_OAR21_46196348 |
| 21 | 46179662 | 46244936 | 18 | 65275 | oar3_OAR21_46179662 | oar3_OAR21_46244936 |
| 21 | 46244936 | 46269375 | 8 | 24440 | oar3_OAR21_46244936 | oar3_OAR21_46269375 |
| 21 | 46329996 | 46372743 | 13 | 42748 | oar3_OAR21_46329996 | oar3_OAR21_46372743 |
| 21 | 46329996 | 46372743 | 13 | 42748 | oar3_OAR21_46329996 | oar3_OAR21_46372743 |
| 21 | 46611608 | 46652632 | 12 | 41025 | oar3_OAR21_46611608 | oar3_OAR21_46652632 |
| 21 | 46637727 | 46666904 | 6 | 29178 | oar3_OAR21_46637727 | oar3_OAR21_46666904 |
| 21 | 46706957 | 46784200 | 16 | 77244 | oar3_OAR21_46706957 | oar3_OAR21_46784200 |
| 21 | 46836923 | 46884430 | 11 | 47508 | oar3_OAR21_46836923 | oar3_OAR21_46884430 |
| 21 | 46959138 | 47028834 | 22 | 69697 | oar3_OAR21_46959138 | oar3_OAR21_47028834 |
| 21 | 46959138 | 47045258 | 27 | 86121 | oar3_OAR21_46959138 | oar3_OAR21_47045258 |
| 21 | 46985441 | 47046811 | 19 | 61371 | oar3_OAR21_46985441 | oar3_OAR21_47046811 |
| 21 | 47003911 | 47046811 | 15 | 42901 | oar3_OAR21_47003911 | oar3_OAR21_47046811 |
| 21 | 47003911 | 47045258 | 14 | 41348 | oar3_OAR21_47003911 | oar3_OAR21_47045258 |
| 21 | 47096250 | 47153203 | 15 | 56954 | oar3_OAR21_47096250 | oar3_OAR21_47153203 |
| 21 | 47124771 | 47174767 | 11 | 49997 | oar3_OAR21_47124771 | oar3_OAR21_47174767 |
| 21 | 47124771 | 47141070 | 6 | 16300 | oar3_OAR21_47124771 | oar3_OAR21_47141070 |
| 21 | 47252276 | 47284494 | 12 | 32219 | oar3_OAR21_47252276 | oar3_OAR21_47284494 |
| 21 | 47283015 | 47284494 | 3 | 1480 | oar3_OAR21_47283015 | oar3_OAR21_47284494 |
| 21 | 47542127 | 47621684 | 20 | 79558 | oar3_OAR21_47542127 | oar3_OAR21_47621684 |
| 21 | 47570550 | 47621684 | 15 | 51135 | oar3_OAR21_47570550 | oar3_OAR21_47621684 |
| 21 | 47570550 | 47621684 | 15 | 51135 | oar3_OAR21_47570550 | oar3_OAR21_47621684 |
| 21 | 47578264 | 47621684 | 12 | 43421 | oar3_OAR21_47578264 | oar3_OAR21_47621684 |
| 21 | 47711116 | 47763150 | 11 | 52035 | oar3_OAR21_47711116 | oar3_OAR21_47763150 |
| 21 | 47711116 | 47835912 | 22 | 124797 | oar3_OAR21_47711116 | oar3_OAR21_47835912 |
| 21 | 47741095 | 47835334 | 18 | 94240 | oar3_OAR21_47741095 | oar3_OAR21_47835334 |
| 21 | 47741095 | 47836784 | 20 | 95690 | oar3_OAR21_47741095 | oar3_OAR21_47836784 |
| 21 | 47744758 | 47763150 | 6 | 18393 | oar3_OAR21_47744758 | oar3_OAR21_47763150 |
| 21 | 47753823 | 47823659 | 12 | 69837 | oar3_OAR21_47753823 | oar3_OAR21_47823659 |
| 21 | 47792637 | 47833940 | 7 | 41304 | oar3_OAR21_47792637 | oar3_OAR21_47833940 |
| 21 | 47896974 | 48001464 | 18 | 104491 | oar3_OAR21_47896974 | oar3_OAR21_48001464 |
| 21 | 47912658 | 48065729 | 24 | 153072 | oar3_OAR21_47912658 | oar3_OAR21_48065729 |
| 21 | 47975212 | 48006661 | 7 | 31450 | oar3_OAR21_47975212 | oar3_OAR21_48006661 |
| 21 | 47993094 | 48001464 | 3 | 8371 | oar3_OAR21_47993094 | oar3_OAR21_48001464 |
| 21 | 48039823 | 48122050 | 13 | 82228 | oar3_OAR21_48039823 | oar3_OAR21_48122050 |
| 21 | 48039823 | 48065729 | 6 | 25907 | oar3_OAR21_48039823 | oar3_OAR21_48065729 |
| 21 | 48039823 | 48065729 | 6 | 25907 | oar3_OAR21_48039823 | oar3_OAR21_48065729 |
| 21 | 48065729 | 48136693 | 11 | 70965 | oar3_OAR21_48065729 | oar3_OAR21_48136693 |
| 21 | 48065729 | 48132532 | 10 | 66804 | oar3_OAR21_48065729 | oar3_OAR21_48132532 |
| 21 | 48391133 | 48438703 | 7 | 47571 | oar3_OAR21_48391133 | oar3_OAR21_48438703 |
| 21 | 48391133 | 48399922 | 6 | 8790 | oar3_OAR21_48391133 | oar3_OAR21_48399922 |
| 21 | 48391133 | 48499178 | 18 | 108046 | oar3_OAR21_48391133 | oar3_OAR21_48499178 |
| 21 | 48397952 | 48499178 | 16 | 101227 | oar3_OAR21_48397952 | oar3_OAR21_48499178 |
| 21 | 48397952 | 48450239 | 9 | 52288 | oar3_OAR21_48397952 | oar3_OAR21_48450239 |
| 21 | 48397952 | 48438703 | 5 | 40752 | oar3_OAR21_48397952 | oar3_OAR21_48438703 |
| 21 | 48397952 | 48399922 | 4 | 1971 | oar3_OAR21_48397952 | oar3_OAR21_48399922 |
| 21 | 48397952 | 48399922 | 4 | 1971 | oar3_OAR21_48397952 | oar3_OAR21_48399922 |
| 21 | 48643800 | 48694234 | 7 | 50435 | oar3_OAR21_48643800 | oar3_OAR21_48694234 |
| 21 | 48763619 | 48827874 | 9 | 64256 | oar3_OAR21_48763619 | s64286.1 |
| 21 | 48863630 | 48994407 | 14 | 130778 | oar3_OAR21_48863630 | oar3_OAR21_48994407 |
| 21 | 48954915 | 49007031 | 7 | 52117 | oar3_OAR21_48954915 | oar3_OAR21_49007031 |
| 21 | 49054811 | 49104226 | 13 | 49416 | oar3_OAR21_49054811 | oar3_OAR21_49104226 |
| 21 | 49151077 | 49182610 | 8 | 31534 | oar3_OAR21_49151077 | oar3_OAR21_49182610 |
| 21 | 49220135 | 49318860 | 10 | 98726 | oar3_OAR21_49220135 | oar3_OAR21_49318860 |
| 21 | 49220135 | 49413987 | 18 | 193853 | oar3_OAR21_49220135 | oar3_OAR21_49413987 |
| 21 | 49220135 | 49256526 | 6 | 36392 | oar3_OAR21_49220135 | oar3_OAR21_49256526 |
| 21 | 49220135 | 49452769 | 23 | 232635 | oar3_OAR21_49220135 | oar3_OAR21_49452769 |
| 21 | 49220135 | 49362527 | 14 | 142393 | oar3_OAR21_49220135 | oar3_OAR21_49362527 |
| 21 | 49336701 | 49514645 | 20 | 177945 | oar3_OAR21_49336701 | oar3_OAR21_49514645 |
| 21 | 49351108 | 49362527 | 3 | 11420 | oar3_OAR21_49351108 | oar3_OAR21_49362527 |
| 21 | 49405978 | 49927360 | 37 | 521383 | oar3_OAR21_49405978 | oar3_OAR21_49927360 |
| 21 | 49405978 | 49599274 | 25 | 193297 | oar3_OAR21_49405978 | oar3_OAR21_49599274 |
| 21 | 49405978 | 49599274 | 25 | 193297 | oar3_OAR21_49405978 | oar3_OAR21_49599274 |
| 21 | 49405978 | 49599274 | 25 | 193297 | oar3_OAR21_49405978 | oar3_OAR21_49599274 |
| 21 | 49471252 | 49514645 | 5 | 43394 | oar3_OAR21_49471252 | oar3_OAR21_49514645 |
| 21 | 49471252 | 49599274 | 16 | 128023 | oar3_OAR21_49471252 | oar3_OAR21_49599274 |
| 21 | 49471252 | 49950631 | 31 | 479380 | oar3_OAR21_49471252 | oar3_OAR21_49950631 |
| 21 | 49492537 | 49599274 | 15 | 106738 | oar3_OAR21_49492537 | oar3_OAR21_49599274 |
| 21 | 49503951 | 49927360 | 26 | 423410 | oar3_OAR21_49503951 | oar3_OAR21_49927360 |
| 21 | 49581935 | 49599274 | 5 | 17340 | oar3_OAR21_49581935 | oar3_OAR21_49599274 |
| 21 | 49895023 | 49922145 | 5 | 27123 | OAR21_54563161.1 | oar3_OAR21_49922145 |
| 21 | 49895023 | 49922145 | 5 | 27123 | OAR21_54563161.1 | oar3_OAR21_49922145 |
| 21 | 49895023 | 49917169 | 4 | 22147 | OAR21_54563161.1 | oar3_OAR21_49917169 |
| 21 | 49895023 | 49922145 | 5 | 27123 | OAR21_54563161.1 | oar3_OAR21_49922145 |
| 21 | 49895023 | 49950631 | 9 | 55609 | OAR21_54563161.1 | oar3_OAR21_49950631 |
| 21 | 49895023 | 49950631 | 9 | 55609 | OAR21_54563161.1 | oar3_OAR21_49950631 |
| 21 | 49992498 | 50048181 | 10 | 55684 | oar3_OAR21_49992498 | oar3_OAR21_50048181 |
| 21 | 49992498 | 50001934 | 5 | 9437 | oar3_OAR21_49992498 | oar3_OAR21_50001934 |
| 21 | 49992498 | 50029788 | 8 | 37291 | oar3_OAR21_49992498 | s28122.1 |
| 21 | 49992498 | 50048181 | 10 | 55684 | oar3_OAR21_49992498 | oar3_OAR21_50048181 |
| 22 | 440384 | 441770 | 3 | 1387 | oar3_OAR22_440384 | oar3_OAR22_441770 |
| 22 | 1202652 | 1219518 | 4 | 16867 | oar3_OAR22_1202652 | oar3_OAR22_1219518 |
| 22 | 1596281 | 1726160 | 21 | 129880 | oar3_OAR22_1596281 | oar3_OAR22_1726160 |
| 22 | 1930252 | 1956245 | 8 | 25994 | oar3_OAR22_1930252 | oar3_OAR22_1956245 |
| 22 | 2144406 | 2192410 | 8 | 48005 | oar3_OAR22_2144406 | oar3_OAR22_2192410 |
| 22 | 17768584 | 17834115 | 18 | 65532 | oar3_OAR22_17768584 | oar3_OAR22_17834115 |
| 22 | 17821491 | 17834115 | 12 | 12625 | oar3_OAR22_17821491 | oar3_OAR22_17834115 |
| 22 | 18118139 | 18132645 | 9 | 14507 | oar3_OAR22_18118139 | oar3_OAR22_18132645 |
| 22 | 18118139 | 18132645 | 9 | 14507 | oar3_OAR22_18118139 | oar3_OAR22_18132645 |
| 22 | 18118139 | 18132645 | 9 | 14507 | oar3_OAR22_18118139 | oar3_OAR22_18132645 |
| 22 | 21108773 | 21228345 | 21 | 119573 | oar3_OAR22_21108773 | oar3_OAR22_21228345 |
| 22 | 23194863 | 23234132 | 10 | 39270 | s09141.1 | oar3_OAR22_23234132 |
| 22 | 23218970 | 23263482 | 15 | 44513 | oar3_OAR22_23218970 | oar3_OAR22_23263482 |
| 22 | 36793059 | 36816249 | 9 | 23191 | oar3_OAR22_36793059 | oar3_OAR22_36816249 |
| 22 | 36793059 | 36816249 | 9 | 23191 | oar3_OAR22_36793059 | oar3_OAR22_36816249 |
| 22 | 36793059 | 36816249 | 9 | 23191 | oar3_OAR22_36793059 | oar3_OAR22_36816249 |
| 22 | 40894056 | 40927912 | 13 | 33857 | oar3_OAR22_40894056 | s41885.1 |
| 22 | 42559348 | 42599791 | 14 | 40444 | oar3_OAR22_42559348 | oar3_OAR22_42599791 |
| 22 | 42967039 | 43121418 | 31 | 154380 | oar3_OAR22_42967039 | oar3_OAR22_43121418 |
| 22 | 42983433 | 43172058 | 38 | 188626 | oar3_OAR22_42983433 | oar3_OAR22_43172058 |
| 22 | 43121418 | 43182266 | 16 | 60849 | oar3_OAR22_43121418 | s31685.1 |
| 22 | 43121418 | 43189643 | 17 | 68226 | oar3_OAR22_43121418 | oar3_OAR22_43189643 |
| 22 | 43153949 | 43176722 | 9 | 22774 | oar3_OAR22_43153949 | oar3_OAR22_43176722 |
| 22 | 43153949 | 43172058 | 6 | 18110 | oar3_OAR22_43153949 | oar3_OAR22_43172058 |
| 22 | 43153949 | 43172058 | 6 | 18110 | oar3_OAR22_43153949 | oar3_OAR22_43172058 |
| 22 | 43449303 | 43453687 | 4 | 4385 | oar3_OAR22_43449303 | oar3_OAR22_43453687 |
| 22 | 46400719 | 46443024 | 15 | 42306 | oar3_OAR22_46400719 | oar3_OAR22_46443024 |
| 22 | 48097197 | 48152456 | 18 | 55260 | oar3_OAR22_48097197 | oar3_OAR22_48152456 |
| 22 | 48120705 | 48163517 | 18 | 42813 | oar3_OAR22_48120705 | s39518.1 |
| 22 | 48120705 | 48163517 | 18 | 42813 | oar3_OAR22_48120705 | s39518.1 |
| 22 | 48265735 | 48275217 | 6 | 9483 | oar3_OAR22_48265735 | oar3_OAR22_48275217 |
| 22 | 48487311 | 48503154 | 8 | 15844 | oar3_OAR22_48487311 | oar3_OAR22_48503154 |
| 22 | 48599090 | 48629208 | 17 | 30119 | oar3_OAR22_48599090 | oar3_OAR22_48629208 |
| 22 | 49006159 | 49024444 | 8 | 18286 | oar3_OAR22_49006159 | oar3_OAR22_49024444 |
| 22 | 49006159 | 49016281 | 5 | 10123 | oar3_OAR22_49006159 | oar3_OAR22_49016281 |
| 22 | 49113550 | 49165090 | 17 | 51541 | oar3_OAR22_49113550 | oar3_OAR22_49165090 |
| 22 | 49356110 | 49415842 | 18 | 59733 | oar3_OAR22_49356110 | oar3_OAR22_49415842 |
| 22 | 49384312 | 49415842 | 10 | 31531 | oar3_OAR22_49384312 | oar3_OAR22_49415842 |
| 22 | 49392660 | 49415842 | 8 | 23183 | oar3_OAR22_49392660 | oar3_OAR22_49415842 |
| 22 | 49409850 | 49415842 | 5 | 5993 | oar3_OAR22_49409850 | oar3_OAR22_49415842 |
| 22 | 49409850 | 49415842 | 5 | 5993 | oar3_OAR22_49409850 | oar3_OAR22_49415842 |
| 22 | 49409850 | 49429758 | 10 | 19909 | oar3_OAR22_49409850 | oar3_OAR22_49429758 |
| 22 | 49465938 | 49546682 | 17 | 80745 | oar3_OAR22_49465938 | oar3_OAR22_49546682 |
| 22 | 49570512 | 49577646 | 7 | 7135 | oar3_OAR22_49570512 | oar3_OAR22_49577646 |
| 22 | 49601512 | 49645486 | 13 | 43975 | oar3_OAR22_49601512 | s00052.1 |
| 22 | 49601512 | 49626803 | 10 | 25292 | oar3_OAR22_49601512 | oar3_OAR22_49626803 |
| 22 | 49620076 | 49678562 | 19 | 58487 | oar3_OAR22_49620076 | oar3_OAR22_49678562 |
| 22 | 49660523 | 49678562 | 10 | 18040 | oar3_OAR22_49660523 | oar3_OAR22_49678562 |
| 22 | 49661731 | 49744773 | 24 | 83043 | oar3_OAR22_49661731 | oar3_OAR22_49744773 |
| 22 | 49666607 | 49678562 | 8 | 11956 | oar3_OAR22_49666607 | oar3_OAR22_49678562 |
| 22 | 49730701 | 49992027 | 47 | 261327 | oar3_OAR22_49730701 | s26220.1 |
| 22 | 49740459 | 49760563 | 8 | 20105 | oar3_OAR22_49740459 | oar3_OAR22_49760563 |
| 22 | 49801999 | 50190070 | 63 | 388072 | oar3_OAR22_49801999 | oar3_OAR22_50190070 |
| 22 | 49801999 | 50045905 | 39 | 243907 | oar3_OAR22_49801999 | oar3_OAR22_50045905 |
| 22 | 49801999 | 49949686 | 26 | 147688 | oar3_OAR22_49801999 | oar3_OAR22_49949686 |
| 22 | 49803886 | 49851848 | 13 | 47963 | oar3_OAR22_49803886 | oar3_OAR22_49851848 |
| 22 | 49803886 | 50624440 | 132 | 820555 | oar3_OAR22_49803886 | oar3_OAR22_50624440 |
| 22 | 49806183 | 49851848 | 12 | 45666 | oar3_OAR22_49806183 | oar3_OAR22_49851848 |
| 22 | 49806183 | 49847105 | 11 | 40923 | oar3_OAR22_49806183 | oar3_OAR22_49847105 |
| 22 | 49905774 | 49970115 | 8 | 64342 | s58002.1 | oar3_OAR22_49970115 |
| 22 | 49905774 | 49970115 | 8 | 64342 | s58002.1 | oar3_OAR22_49970115 |
| 22 | 49905774 | 49970115 | 8 | 64342 | s58002.1 | oar3_OAR22_49970115 |
| 22 | 49905774 | 49992027 | 11 | 86254 | s58002.1 | s26220.1 |
| 22 | 49907858 | 49992027 | 10 | 84170 | oar3_OAR22_49907858 | s26220.1 |
| 22 | 49907858 | 49970115 | 7 | 62258 | oar3_OAR22_49907858 | oar3_OAR22_49970115 |
| 22 | 49936759 | 50016570 | 12 | 79812 | oar3_OAR22_49936759 | oar3_OAR22_50016570 |
| 22 | 49936759 | 49970115 | 5 | 33357 | oar3_OAR22_49936759 | oar3_OAR22_49970115 |
| 22 | 49951008 | 50003422 | 7 | 52415 | oar3_OAR22_49951008 | s49523.1 |
| 22 | 50002340 | 50055603 | 10 | 53264 | oar3_OAR22_50002340 | oar3_OAR22_50055603 |
| 22 | 50002340 | 50239761 | 38 | 237422 | oar3_OAR22_50002340 | oar3_OAR22_50239761 |
| 22 | 50005828 | 50033480 | 5 | 27653 | oar3_OAR22_50005828 | oar3_OAR22_50033480 |
| 22 | 50095720 | 50297216 | 31 | 201497 | oar3_OAR22_50095720 | oar3_OAR22_50297216 |
| 22 | 50143987 | 50239761 | 15 | 95775 | oar3_OAR22_50143987 | oar3_OAR22_50239761 |
| 22 | 50149478 | 50182593 | 6 | 33116 | oar3_OAR22_50149478 | oar3_OAR22_50182593 |
| 22 | 50149478 | 50624440 | 78 | 474963 | oar3_OAR22_50149478 | oar3_OAR22_50624440 |
| 22 | 50182593 | 50245707 | 10 | 63115 | oar3_OAR22_50182593 | oar3_OAR22_50245707 |
| 22 | 50214489 | 50258619 | 9 | 44131 | oar3_OAR22_50214489 | oar3_OAR22_50258619 |
| 22 | 50215847 | 50258619 | 8 | 42773 | oar3_OAR22_50215847 | oar3_OAR22_50258619 |
| 22 | 50326245 | 50544134 | 36 | 217890 | oar3_OAR22_50326245 | oar3_OAR22_50544134 |
| 22 | 50348506 | 50456263 | 17 | 107758 | oar3_OAR22_50348506 | oar3_OAR22_50456263 |
| 22 | 50364783 | 50508154 | 26 | 143372 | oar3_OAR22_50364783 | oar3_OAR22_50508154 |
| 22 | 50364783 | 50624440 | 46 | 259658 | oar3_OAR22_50364783 | oar3_OAR22_50624440 |
| 22 | 50395713 | 50476521 | 13 | 80809 | oar3_OAR22_50395713 | oar3_OAR22_50476521 |
| 22 | 50395713 | 50495631 | 18 | 99919 | oar3_OAR22_50395713 | oar3_OAR22_50495631 |
| 22 | 50419302 | 50569495 | 32 | 150194 | oar3_OAR22_50419302 | oar3_OAR22_50569495 |
| 22 | 50506778 | 50569495 | 18 | 62718 | oar3_OAR22_50506778 | oar3_OAR22_50569495 |
| 22 | 50506778 | 50624440 | 23 | 117663 | oar3_OAR22_50506778 | oar3_OAR22_50624440 |
| 22 | 50750467 | 50789329 | 6 | 38863 | oar3_OAR22_50750467 | oar3_OAR22_50789329 |
| 22 | 50750467 | 50828450 | 10 | 77984 | oar3_OAR22_50750467 | oar3_OAR22_50828450 |
| 22 | 50750467 | 50789329 | 6 | 38863 | oar3_OAR22_50750467 | oar3_OAR22_50789329 |
| 22 | 50750467 | 50767060 | 5 | 16594 | oar3_OAR22_50750467 | oar3_OAR22_50767060 |
| 22 | 50750467 | 50809926 | 8 | 59460 | oar3_OAR22_50750467 | oar3_OAR22_50809926 |
| 22 | 50750467 | 50828450 | 10 | 77984 | oar3_OAR22_50750467 | oar3_OAR22_50828450 |
| 22 | 50750467 | 50828450 | 10 | 77984 | oar3_OAR22_50750467 | oar3_OAR22_50828450 |
| 22 | 50750467 | 50767060 | 5 | 16594 | oar3_OAR22_50750467 | oar3_OAR22_50767060 |
| 22 | 50750467 | 50789329 | 6 | 38863 | oar3_OAR22_50750467 | oar3_OAR22_50789329 |
| 22 | 50750467 | 50789329 | 6 | 38863 | oar3_OAR22_50750467 | oar3_OAR22_50789329 |
| 22 | 50750467 | 50828450 | 10 | 77984 | oar3_OAR22_50750467 | oar3_OAR22_50828450 |
| 23 | 507057 | 532340 | 7 | 25284 | oar3_OAR23_507057 | oar3_OAR23_532340 |
| 23 | 507057 | 584268 | 17 | 77212 | oar3_OAR23_507057 | oar3_OAR23_584268 |
| 23 | 580390 | 656154 | 22 | 75765 | oar3_OAR23_580390 | s02518.1 |
| 23 | 580390 | 645827 | 16 | 65438 | oar3_OAR23_580390 | oar3_OAR23_645827 |
| 23 | 600212 | 679694 | 21 | 79483 | oar3_OAR23_600212 | oar3_OAR23_679694 |
| 23 | 678302 | 862651 | 33 | 184350 | oar3_OAR23_678302 | oar3_OAR23_862651 |
| 23 | 707354 | 783164 | 14 | 75811 | oar3_OAR23_707354 | oar3_OAR23_783164 |
| 23 | 818930 | 868836 | 10 | 49907 | oar3_OAR23_818930 | oar3_OAR23_868836 |
| 23 | 848215 | 868836 | 5 | 20622 | s10401.1 | oar3_OAR23_868836 |
| 23 | 988122 | 1057476 | 16 | 69355 | oar3_OAR23_988122 | oar3_OAR23_1057476 |
| 23 | 2185050 | 2195051 | 5 | 10002 | oar3_OAR23_2185050 | oar3_OAR23_2195051 |
| 23 | 2259370 | 2354138 | 23 | 94769 | oar3_OAR23_2259370 | oar3_OAR23_2354138 |
| 23 | 2727291 | 2734327 | 4 | 7037 | oar3_OAR23_2727291 | oar3_OAR23_2734327 |
| 23 | 4364845 | 4379405 | 5 | 14561 | oar3_OAR23_4364845 | oar3_OAR23_4379405 |
| 23 | 7097078 | 7131429 | 8 | 34352 | oar3_OAR23_7097078 | oar3_OAR23_7131429 |
| 23 | 7123081 | 7131429 | 5 | 8349 | oar3_OAR23_7123081 | oar3_OAR23_7131429 |
| 23 | 41537827 | 41580395 | 9 | 42569 | oar3_OAR23_41537827 | oar3_OAR23_41580395 |
| 23 | 41545145 | 41568655 | 6 | 23511 | oar3_OAR23_41545145 | oar3_OAR23_41568655 |
| 23 | 41545145 | 41580395 | 8 | 35251 | oar3_OAR23_41545145 | oar3_OAR23_41580395 |
| 23 | 41545145 | 41599098 | 12 | 53954 | oar3_OAR23_41545145 | oar3_OAR23_41599098 |
| 23 | 41545145 | 41580395 | 8 | 35251 | oar3_OAR23_41545145 | oar3_OAR23_41580395 |
| 23 | 41545145 | 41580395 | 8 | 35251 | oar3_OAR23_41545145 | oar3_OAR23_41580395 |
| 23 | 46398340 | 46421161 | 10 | 22822 | oar3_OAR23_46398340 | oar3_OAR23_46421161 |
| 23 | 48610690 | 48646573 | 10 | 35884 | s03405.1 | oar3_OAR23_48646573 |
| 23 | 56579398 | 56585941 | 9 | 6544 | oar3_OAR23_56579398 | oar3_OAR23_56585941 |
| 23 | 61007787 | 61035484 | 8 | 27698 | oar3_OAR23_61007787 | oar3_OAR23_61035484 |
| 23 | 61015186 | 61122299 | 21 | 107114 | oar3_OAR23_61015186 | oar3_OAR23_61122299 |
| 23 | 61015186 | 61037389 | 9 | 22204 | oar3_OAR23_61015186 | oar3_OAR23_61037389 |
| 23 | 61025543 | 61043741 | 7 | 18199 | oar3_OAR23_61025543 | oar3_OAR23_61043741 |
| 23 | 61091348 | 61198865 | 14 | 107518 | oar3_OAR23_61091348 | oar3_OAR23_61198865 |
| 23 | 61116580 | 61206404 | 13 | 89825 | oar3_OAR23_61116580 | oar3_OAR23_61206404 |
| 23 | 61513577 | 61517275 | 4 | 3699 | oar3_OAR23_61513577 | oar3_OAR23_61517275 |
| 23 | 61641766 | 61660282 | 8 | 18517 | oar3_OAR23_61641766 | s72313.1 |
| 23 | 61822015 | 61891760 | 20 | 69746 | oar3_OAR23_61822015 | oar3_OAR23_61891760 |
| 24 | 43498 | 94872 | 13 | 51375 | oar3_OAR24_43498 | oar3_OAR24_94872 |
| 24 | 43498 | 81079 | 9 | 37582 | oar3_OAR24_43498 | oar3_OAR24_81079 |
| 24 | 43498 | 1380176 | 271 | 1336679 | oar3_OAR24_43498 | oar3_OAR24_1380176 |
| 24 | 43498 | 94872 | 13 | 51375 | oar3_OAR24_43498 | oar3_OAR24_94872 |
| 24 | 64798 | 135005 | 19 | 70208 | oar3_OAR24_64798 | oar3_OAR24_135005 |
| 24 | 69274 | 90303 | 5 | 21030 | oar3_OAR24_69274 | oar3_OAR24_90303 |
| 24 | 69274 | 114775 | 12 | 45502 | oar3_OAR24_69274 | oar3_OAR24_114775 |
| 24 | 81079 | 114775 | 11 | 33697 | oar3_OAR24_81079 | oar3_OAR24_114775 |
| 24 | 81079 | 114775 | 11 | 33697 | oar3_OAR24_81079 | oar3_OAR24_114775 |
| 24 | 81079 | 114775 | 11 | 33697 | oar3_OAR24_81079 | oar3_OAR24_114775 |
| 24 | 105806 | 586007 | 91 | 480202 | oar3_OAR24_105806 | oar3_OAR24_586007 |
| 24 | 201064 | 289883 | 27 | 88820 | oar3_OAR24_201064 | oar3_OAR24_289883 |
| 24 | 201064 | 219096 | 5 | 18033 | oar3_OAR24_201064 | oar3_OAR24_219096 |
| 24 | 219096 | 247734 | 10 | 28639 | oar3_OAR24_219096 | oar3_OAR24_247734 |
| 24 | 219096 | 247734 | 10 | 28639 | oar3_OAR24_219096 | oar3_OAR24_247734 |
| 24 | 219096 | 247734 | 10 | 28639 | oar3_OAR24_219096 | oar3_OAR24_247734 |
| 24 | 223978 | 285964 | 20 | 61987 | oar3_OAR24_223978 | oar3_OAR24_285964 |
| 24 | 223978 | 247734 | 9 | 23757 | oar3_OAR24_223978 | oar3_OAR24_247734 |
| 24 | 279503 | 289883 | 10 | 10381 | oar3_OAR24_279503 | oar3_OAR24_289883 |
| 24 | 279503 | 289883 | 10 | 10381 | oar3_OAR24_279503 | oar3_OAR24_289883 |
| 24 | 279503 | 289883 | 10 | 10381 | oar3_OAR24_279503 | oar3_OAR24_289883 |
| 24 | 279503 | 289883 | 10 | 10381 | oar3_OAR24_279503 | oar3_OAR24_289883 |
| 24 | 279503 | 289883 | 10 | 10381 | oar3_OAR24_279503 | oar3_OAR24_289883 |
| 24 | 283338 | 289883 | 6 | 6546 | oar3_OAR24_283338 | oar3_OAR24_289883 |
| 24 | 367978 | 564975 | 30 | 196998 | oar3_OAR24_367978 | oar3_OAR24_564975 |
| 24 | 367978 | 591843 | 37 | 223866 | oar3_OAR24_367978 | oar3_OAR24_591843 |
| 24 | 367978 | 564975 | 30 | 196998 | oar3_OAR24_367978 | oar3_OAR24_564975 |
| 24 | 402305 | 564975 | 24 | 162671 | oar3_OAR24_402305 | oar3_OAR24_564975 |
| 24 | 402305 | 448251 | 9 | 45947 | oar3_OAR24_402305 | oar3_OAR24_448251 |
| 24 | 402305 | 448251 | 9 | 45947 | oar3_OAR24_402305 | oar3_OAR24_448251 |
| 24 | 402305 | 438011 | 8 | 35707 | oar3_OAR24_402305 | oar3_OAR24_438011 |
| 24 | 402305 | 428044 | 5 | 25740 | oar3_OAR24_402305 | oar3_OAR24_428044 |
| 24 | 406401 | 457915 | 10 | 51515 | oar3_OAR24_406401 | oar3_OAR24_457915 |
| 24 | 424911 | 438011 | 6 | 13101 | oar3_OAR24_424911 | oar3_OAR24_438011 |
| 24 | 424911 | 428044 | 3 | 3134 | oar3_OAR24_424911 | oar3_OAR24_428044 |
| 24 | 424911 | 428044 | 3 | 3134 | oar3_OAR24_424911 | oar3_OAR24_428044 |
| 24 | 454313 | 564975 | 15 | 110663 | oar3_OAR24_454313 | oar3_OAR24_564975 |
| 24 | 454313 | 586007 | 19 | 131695 | oar3_OAR24_454313 | oar3_OAR24_586007 |
| 24 | 542243 | 586007 | 9 | 43765 | oar3_OAR24_542243 | oar3_OAR24_586007 |
| 24 | 545620 | 573851 | 6 | 28232 | oar3_OAR24_545620 | oar3_OAR24_573851 |
| 24 | 545620 | 564975 | 4 | 19356 | oar3_OAR24_545620 | oar3_OAR24_564975 |
| 24 | 552557 | 609485 | 16 | 56929 | oar3_OAR24_552557 | oar3_OAR24_609485 |
| 24 | 603054 | 656676 | 10 | 53623 | oar3_OAR24_603054 | oar3_OAR24_656676 |
| 24 | 603054 | 850574 | 60 | 247521 | oar3_OAR24_603054 | oar3_OAR24_850574 |
| 24 | 603054 | 766586 | 44 | 163533 | oar3_OAR24_603054 | oar3_OAR24_766586 |
| 24 | 639830 | 693244 | 14 | 53415 | oar3_OAR24_639830 | oar3_OAR24_693244 |
| 24 | 676839 | 766586 | 30 | 89748 | oar3_OAR24_676839 | oar3_OAR24_766586 |
| 24 | 676839 | 794641 | 36 | 117803 | oar3_OAR24_676839 | oar3_OAR24_794641 |
| 24 | 676839 | 858907 | 48 | 182069 | oar3_OAR24_676839 | oar3_OAR24_858907 |
| 24 | 676839 | 834655 | 41 | 157817 | oar3_OAR24_676839 | oar3_OAR24_834655 |
| 24 | 676839 | 744416 | 26 | 67578 | oar3_OAR24_676839 | oar3_OAR24_744416 |
| 24 | 715738 | 834655 | 27 | 118918 | oar3_OAR24_715738 | oar3_OAR24_834655 |
| 24 | 727865 | 744416 | 9 | 16552 | oar3_OAR24_727865 | oar3_OAR24_744416 |
| 24 | 732565 | 744416 | 7 | 11852 | oar3_OAR24_732565 | oar3_OAR24_744416 |
| 24 | 732565 | 744416 | 7 | 11852 | oar3_OAR24_732565 | oar3_OAR24_744416 |
| 24 | 791618 | 927330 | 28 | 135713 | oar3_OAR24_791618 | oar3_OAR24_927330 |
| 24 | 812557 | 858907 | 11 | 46351 | oar3_OAR24_812557 | oar3_OAR24_858907 |
| 24 | 897190 | 914967 | 4 | 17778 | oar3_OAR24_897190 | oar3_OAR24_914967 |
| 24 | 897190 | 934033 | 8 | 36844 | oar3_OAR24_897190 | oar3_OAR24_934033 |
| 24 | 897190 | 914967 | 4 | 17778 | oar3_OAR24_897190 | oar3_OAR24_914967 |
| 24 | 911293 | 962868 | 12 | 51576 | oar3_OAR24_911293 | s34109.1 |
| 24 | 911293 | 961260 | 10 | 49968 | oar3_OAR24_911293 | oar3_OAR24_961260 |
| 24 | 911293 | 1086558 | 35 | 175266 | oar3_OAR24_911293 | oar3_OAR24_1086558 |
| 24 | 911293 | 961260 | 10 | 49968 | oar3_OAR24_911293 | oar3_OAR24_961260 |
| 24 | 978039 | 1065883 | 19 | 87845 | oar3_OAR24_978039 | oar3_OAR24_1065883 |
| 24 | 981413 | 982755 | 5 | 1343 | oar3_OAR24_981413 | oar3_OAR24_982755 |
| 24 | 981413 | 1117615 | 30 | 136203 | oar3_OAR24_981413 | oar3_OAR24_1117615 |
| 24 | 981413 | 982755 | 5 | 1343 | oar3_OAR24_981413 | oar3_OAR24_982755 |
| 24 | 1003199 | 1086558 | 14 | 83360 | oar3_OAR24_1003199 | oar3_OAR24_1086558 |
| 24 | 1003199 | 1092604 | 18 | 89406 | oar3_OAR24_1003199 | oar3_OAR24_1092604 |
| 24 | 1003199 | 1065883 | 10 | 62685 | oar3_OAR24_1003199 | oar3_OAR24_1065883 |
| 24 | 1003199 | 1022132 | 6 | 18934 | oar3_OAR24_1003199 | oar3_OAR24_1022132 |
[truncated: 450,024 more chars]
